# The burden of mitochondrial disease with associated seizures: systematic literature reviews of health-related quality of life, utilities, costs and healthcare resource use data

**DOI:** 10.1186/s13023-023-02945-6

**Published:** 2023-10-11

**Authors:** Enrico Bertini, Emily Gregg, Chris Bartlett, Vij Senthilnathan, Mick Arber, Deborah Watkins, Sara Graziadio, Ioannis Tomazos

**Affiliations:** 1 Unit of Neuromuscular and Neurodegenerative Disorders, Bambino Gesu’ Children’s Research Hospital, IRCCS, Rome, Italy; 2 York Health Economics Consortium, York, UK; 3 PTC Therapeutics, South Plainfield, NJ USA

**Keywords:** Mitochondrial diseases, Epilepsy, Seizures, Systematic literature reviews, Disease burden, Utilities, Health state utility values, Health-related quality of life, Costs, Healthcare resource use

## Abstract

**Background:**

Mitochondrial disease is a degenerative, progressive, heterogeneous group of genetic disorders affecting children and adults. Mitochondrial disease is associated with morbidity and mortality, with predominantly neurological and neuromuscular symptoms including dystonia, weakness, encephalopathy, developmental delay and seizures. Seizures are one of the most common and severe manifestations of mitochondrial disease. These seizures are typically refractory to common anti-seizure therapies. There are no approved disease-modifying treatments for mitochondrial disease. Our objective was to conduct two systematic literature reviews to identify health-related quality of life (HRQoL), utilities, costs and healthcare resource use data in mitochondrial disease with associated seizures.

**Methods:**

A range of databases and information sources were searched up to July 2022 to identify eligible studies. Search strategies included a range of variant terms for mitochondrial disease and HRQoL, utilities, cost and healthcare resource use outcomes. Two reviewers independently assessed articles against the eligibility criteria; studies were extracted by one reviewer and checked by a second. Risk of bias was assessed for studies reporting HRQoL data. Results were narratively assessed.

**Results:**

Seven studies were eligible for the HRQoL and utilities review. The studies used different tools to report data, and despite the variability in methods, HRQoL scores across the studies showed moderate/severe disease in patients with mitochondrial disease with associated seizures. Parents of patients with mitochondrial disease with associated seizures were characterised by high total parenting stress. No studies reported utilities data. Two case reports and one retrospective review of medical records of children who died in hospital were eligible for the costs and resource use review. These provided limited information on the duration of hospital stay, in an intensive care unit (ICU), on mechanical ventilation. No studies reported costs data.

**Conclusion:**

These reviews highlight the limited HRQoL, utilities, costs and resource use data and the variability of instruments used in mitochondrial disease with associated seizures. However, the data available indicate that mitochondrial disease with associated seizures affects patients’ and caregivers’ HRQoL alike. No robust conclusion can be drawn on the impact of mitochondrial disease with associated seizures on hospital or ICU length of stay.

*Trial registration* PROSPERO: CRD42022345005.

**Supplementary Information:**

The online version contains supplementary material available at 10.1186/s13023-023-02945-6.

## Background

Mitochondrial disease is a diverse group of rare disorders, resulting from mutations in both mitochondrial and nuclear DNA, causing deficiencies in mitochondrial function [1]. These serious and progressive diseases have an unpredictable disease course and occur in approximately 1 in 5,000 individuals [2, 3]. Mitochondrial diseases affect all organs in the body but especially those with high energy requirements including the brain and heart. Symptoms include muscular and neurological impairments alongside other complications, such as fatigue, behaviour and speech disturbances, impaired vision, and diabetes [4]. Seizures are a common neurological manifestation of mitochondrial disease, affecting up to 40% of adults and 60% of paediatric cases [5, 6].

The pathophysiology of seizures associated with mitochondrial disease is not fully understood, but this is mostly due to the disruption of the mitochondrial respiratory chain that results in cellular energy deficiency and promotes oxidative stress [5]. This can cause an accumulation of lipid peroxides and depletion of reduced glutathione, the cell’s natural antioxidant. These conditions trigger a feed-forward cycle promoting proinflammatory signalling, additional increases in oxidative stress, and, ultimately, cell death. The resultant cellular dysfunction and cell death drives the downstream pathologies observed with mitochondrial disease. In the brain, these events can create an excitatory/inhibitory imbalance that disrupts normal neuronal circuits and can cause seizures. Such seizures can increase morbidity and result in a poor prognosis or epileptic encephalopathy [5]. Children with mitochondrial disease that experience seizures are characterised by more severe disease than those without [7]. Seizures associated with mitochondrial disease are typically refractory to common anti-seizure therapies, as many of the approved seizure therapies are toxic to the mitochondria and exacerbate the underlying pathology of mitochondrial disease. There are no approved disease-modifying treatments for mitochondrial disease or the associated seizures. Currently there are very limited treatments for these patients. Standard of care includes multidisciplinary management and treatment of symptoms, including nutritional supplements and exercise. Furthermore, the diagnosis and management of mitochondrial disease with associated seizures is challenging because the diseases are biochemically and genetically heterogeneous and require multidisciplinary healthcare.

To the authors’ knowledge, there are no published reviews assessing the health-related quality of life (HRQoL) and economic impact of mitochondrial disease with associated seizures, which may present a burden for patients, caregivers and healthcare systems. To address this evidence gap, two systematic literature reviews (SLRs) were undertaken to identify HRQoL, utilities, costs and healthcare resource use data in these patients. Together, the SLRs aimed to provide a holistic understanding of the HRQoL, utility, cost and healthcare resource use burden of mitochondrial disease with associated seizures; therefore, they are both presented within this publication. The SLRs may be used to inform future health technology assessment (HTA) submissions of possible treatments.

## Methods

The SLRs were undertaken following the principles of systematic reviewing embodied in the Cochrane handbook [8] and the Preferred Reporting Items for Systematic Reviews and Meta-Analyses (PRISMA) guidelines [9]. The questions and methods of both SLRs were predefined in one protocol that was registered on the PROSPERO database (CRD42022345005) [10]. The completed PRISMA checklist is presented in Additional file 1.

### Eligibility criteria

Full details of the eligibility criteria for both SLRs are presented in Table 1. The eligibility criteria were consistent between the SLRs and only the eligible outcomes differed. Studies of patients with mitochondrial disease with associated seizures or studies reporting data for caregivers of these patients were eligible for inclusion. The specific eligible mitochondrial diseases (Table 1) were selected because of their increased likelihood of phenotypes of patients with seizures [5]. Studies reporting data from HRQoL and health state utility tools were eligible for the first SLR, and those reporting data on direct monetary costs, indirect monetary costs, and non-monetary healthcare resource use were eligible for the second SLR (Table 1). Eligible studies were limited to those in English language.

**Table 1:** Summary of the eligibility criteria for both SLRs

Criterion	Inclusion criteria	Exclusion criteria
Population	Studies of patients with mitochondrial disease with associated seizures were eligible. Studies reporting data for caregivers of these patients were also eligible Specifically, patients with the following mitochondrial diseases were eligible for inclusion in the SLR: Leigh’s syndrome POLG1 mutation related disorders: AHS MCHS MEMSA SCAE PEO MIRAS SANDO arPEO (includingC10orf2/Twinkle) adPEO MELAS MERRF RARS2 mutation related disorders: Pontocerebellar hypoplasia type 6 PDHC deficiency Studies with mixed populations (> 75% included in the list above) were also eligible In the original SLRs, the terminology used to describe the population was ‘mitochondrial disease with epilepsy’; however, for the update SLRs this was changed to ‘mitochondrial disease with associated seizures’. Although there was a change in terminology, the eligible patient population and study selection were consistent	Animal/in vitro studies Patients with other conditions Patients with mitochondrial diseases who did not have epilepsy
Interventions	No restrictions	
Comparators	No restrictions	
Outcomes	HRQoL and utilities SLR Studies reporting data from HRQoL tools were eligible for inclusion, including: Adult's Attitudes to Children with Epilepsy: Visual Analogue Scale DISABKIDS (Epilepsy Module) ELDQOL ECQ EFA GEOS-YP HARCES CHEQOL-25 Modified Impact of Epilepsy Schedule ICI IPES ICND NeuroQol Newcastle Mitochondrial Disease Questionnaire PedsQL PedsQL Epilepsy Module PESQ PROMIS Paediatric/Parent Proxy Profile: PROMIS Paediatric/Parent Proxy Profile 25 PROMIS Paediatric/Parent Proxy Profile 37 PROMIS Paediatric/Parent Proxy Profile 49 QOLCE: QOLCE 16 QOLCE 55 G-QOLCE QOLIE: QOLIE 31 QOLIE 89 QOLIE-AD-48 QOLPES SF-36 Studies reporting data from the following health state utility tools were eligible for inclusion: AQoL-6D for adolescents AQoL for adults: AQoL-4D AQoL-6D AQoL-8D CHU9D EQ-5D EQ-5D-Y: EQ-5D-Y Proxy Version 1 EQ-5D-Y Proxy Version 2 EQ-5D-Y Interviewer Administered Proxy Version 1 EQ-5D-Y Interviewer Administered Proxy Version 2 HUI HUI2 HUI3 SF-6D 15D 16D 17D AHUM HSCS-PS QWB Costs and healthcare resource use SLR Studies reporting data on the following types of costs and healthcare resource utilisation were eligible for inclusion: Direct monetary costs associated with mitochondrial disease with associated seizures specifically: Direct medical costs Direct non-medical costs Indirect monetary costs, including on caregivers, specifically: Impact on work or education Days lost, e.g. work or education Resource use: Any non-monetary resource use data	Economic evaluation outcomes, e.g. QALYs / ICERs, were ineligible
Study designs and categories of articles	No restrictions on study design providing eligible outcomes were reported	Categories of articles excluded: Opinion pieces Letters Editorials Systematic reviews Systematic reviews published since 2016 were used for reference checking only Studies published as abstracts or conference presentations were not eligible for inclusion
Limits	Limit to English language only	Non-English language articles

*15D* 15-dimensional, *16D* 16-dimensional, *17D* 17-dimensional, *adPEO* Autosomal dominant PEO, *AHS* Alpers–Huttenlocher syndrome, *AHUM* Adolescent Health Utility Measure, *arPEO* Autosomal recessive PEO, *AQoL* Assessment of Quality of Life, *CHEQOL-25* Health-Related Quality of Life Measure for Children with Epilepsy, *CHU9D* Child Health Utility instrument, *ECQ* Epilepsy and Children Questionnaire, *EFA* Epilepsy Foundations of America Concerns Index, *ELDQOL* Epilepsy and Learning Disability Quality of Life, *EQ-5D* EuroQol 5 dimensions, *EQ-5D-Y* EuroQol 5 dimensions (youth), *GEOS-YP* Glasgow Epilepsy Outcome Scale, *G-QOLCE* Global Quality of Life in Childhood Epilepsy, *HARCES* The Hague Restrictions in Childhood Epilepsy Scale, *HSCS-PS* Health Status Classification System-Preschool, *HUI* Health Utilities Index, *ICER* Incremental cost-effectiveness ratio, *ICI* Impact of Childhood Illness Scale, *ICND* Impact of Childhood Neurologic Disability, *IPES* Impact of Paediatric Epilepsy Scale, *MCHS* Childhood myocerebrohepatopathy spectrum, *MELAS* Mitochondrial encephalopathy, lactic acidosis, and stroke-like episode, *MEMSA* Myoclonic epilepsy myopathy sensory ataxia, *MERRF* Myoclonus epilepsy with ragged-red fibers, *MIRAS* Mitochondrial recessive ataxia syndrome, *NeuroQol* Neurology Quality of Life Measurement System, *PDHC* Pyruvate dehydrogenase complex, *PedsQL* Paediatric Quality of Life Inventory, *PEO* Progressive external ophthalmoplegia, *PESQ* Perceptual Evaluation of Speech Quality, *POLG* Polymerase gamma, *PROMIS* Patient-Reported Outcomes Measurement Information System, *QALY* Quality adjusted life year, *QOLCE* Quality of Life in Childhood Epilepsy, *QOLIE-AD-48* Quality of Life in Epilepsy Inventory for Adolescents, *QOLIE* Quality of Life in Epilepsy Inventory, *QOLPES* Quality of Life in Paediatric Epilepsy, *QWB* Quality of Well-Being Scale, *RARS2* Arginyl-tRNA synthetase 2, *SANDO* Sensory ataxia neuropathy dysarthria and ophthalmoplegia, *SCAE* Spinocerebellar ataxia with epilepsy, *SF-36* 36-Item Short Form Survey, *SF-6D* Short Form-6 dimensions, *SLR* Systematic literature review

### Searches

The original searches were conducted in August 2021 and then updated in July 2022. Two separate searches were conducted to inform the two SLRs. Table 2 shows the resources searched for each SLR. Date restrictions were not applied to searches; where appropriate, language restrictions reflecting the eligibility criteria (English language only) were applied. For both SLRs, reference lists of included studies and retrieved relevant SLRs published from 2016 were checked for additional eligible studies. Search methods for the original and update searches (including full search strategies for each database and information source) are presented in Additional file 1.

**Table 2:** Databases and information sources searched

Database / information source	Interface / URL	HRQoL / utilities review	Costs and healthcare resource use review
MEDLINE ALL	OvidSP	Y	Y
Embase	OvidSP	Y	Y
Cochrane Database of Systematic Reviews (CDSR)	Cochrane Library / Wiley	Y	Y
Cochrane Central Register of Controlled Trials (CENTRAL)	Cochrane Library / Wiley	Y	Y
HTA Database	https://database.inahta.org/	Y	Y
NHS Economic Evaluation Database (NHS EED)	https://www.crd.york.ac.uk/CRDWeb/	Y	Y
EconLit	OvidSP	Y	Y
Cost-Effectiveness Analysis (CEA) Registry	https://cevr.tuftsmedicalcenter.org/databases/cea-registry	Y	Y
Paediatric Economic Database Evaluation (PEDE)	http://pede.ccb.sickkids.ca/pede/	Y	Y
APA PsycInfo	OvidSP	Y	N
ScHARRHud	http://www.scharrhud.org/	Y	N
National Institute for Health and Care Excellence (NICE) webpages	https://www.nice.org.uk/	Y	Y
Canadian Agency for Drugs and Technologies in Health (CADTH) webpages	https://www.cadth.ca/	Y	Y
Institute for Clinical and Economic Review (ICER) webpages	https://icer.org/	Y	Y

### Study selection, data extraction and quality assessment

A single researcher assessed the search results according to their relevance in providing information for the reviews and removed the obviously irrelevant records such as those about animals. Two reviewers independently assessed the titles and abstracts then the full texts for relevance against the eligibility criteria, with any disagreements adjudicated by the third reviewer. The number of records included and removed at each selection stage was recorded in a PRISMA flow diagram.

Data extraction (into an Excel template) was conducted by one reviewer, and the second reviewer checked all the data points. Disagreements were adjudicated by the third reviewer. The Excel template was piloted on three studies before progressing to full data extraction. When different studies seemed to match recruitment dates and centres, and patient characteristics, we tried to reach the authors to identify whether the same patients were reported. We aimed to include the data from the same patient only once.

A quality assessment was performed for studies reporting HRQoL data [11]. Full details of the quality assessment are presented in Supplementary Table 5 (in Additional file 1). One reviewer assessed the quality of each study, and the second reviewer checked the assessment. Any disagreements were resolved through discussion or by consulting the third reviewer. No quality assessment was conducted for studies reporting resource use data because the transferability of these data for potential use in future HTAs would not be impacted by an assessment of the elicitation study’s conduct.

The included studies were summarised in tables and through a qualitative synthesis providing data on their methods and results.

## Results

### HRQoL studies identified and selected

**Fig. 1:**
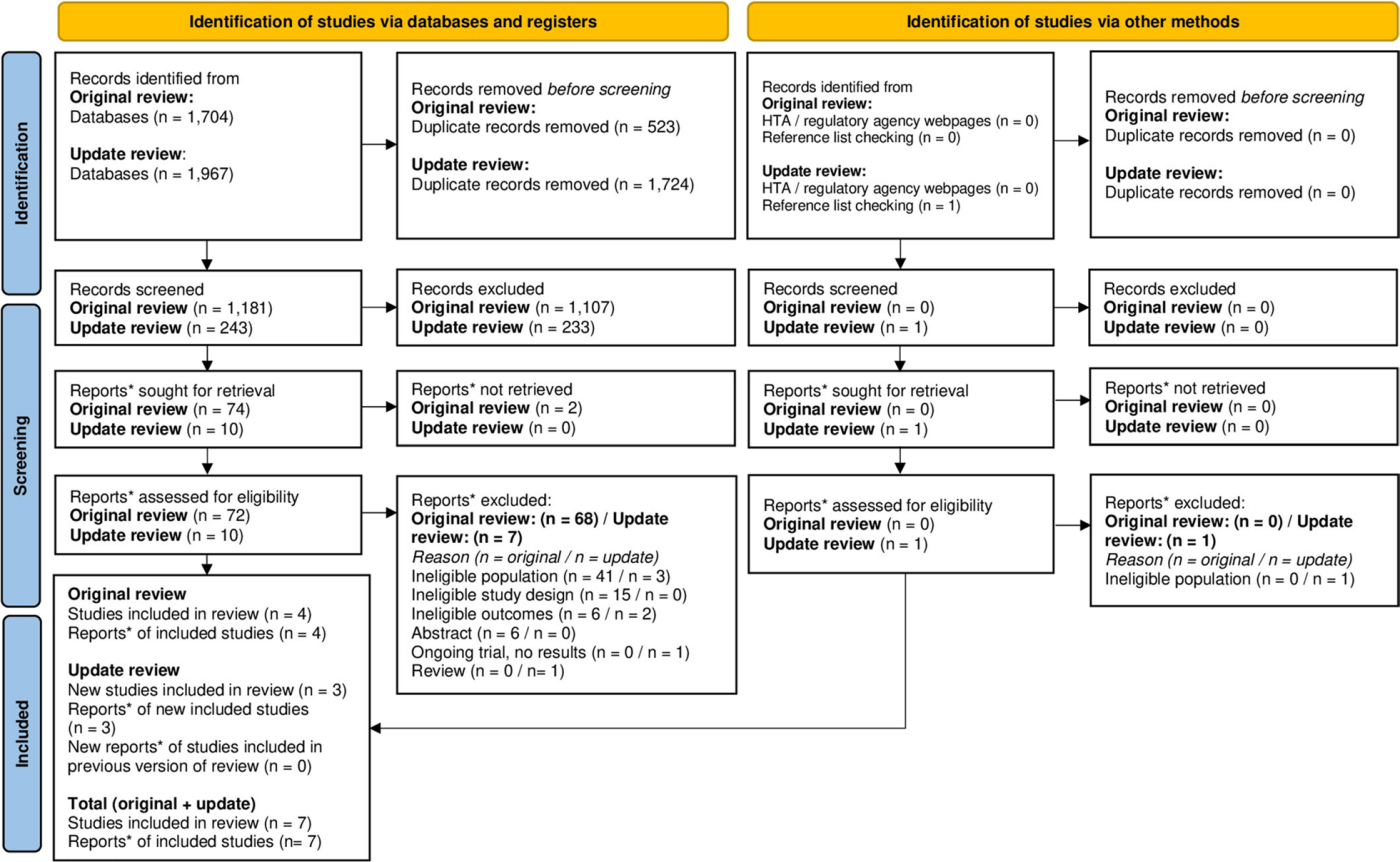
HRQoL and utilities SLR PRISMA. *“Note that a “report” could be a journal article, preprint, conference abstract, study register entry, clinical study report, dissertation, unpublished manuscript, government report or any other document providing relevant information”: https://www.bmj.com/content/372/bmj.n71. *Adapted from:* Page MJ, McKenzie JE, Bossuyt PM, Boutron I, Hoffmann TC, Mulrow CD, et al. The PRISMA 2020 statement: an updated guideline for reporting systematic reviews. BMJ 2021;372:n71. https://doi.org/10.1136/bmj.n71. For more information, visit: http://www.prisma-statement.org/

The original searches identified 1,704 records. Four studies (reported in four documents) were eligible for the SLR. The update searches identified 1,967 records. Three new studies (reported in three documents) were eligible for the SLR. The PRISMA diagram is presented in Fig. 1. Lists of included and excluded studies (with reasons for exclusion) are presented in Additional file 1.

### HRQoL study characteristics

Overall, seven studies (in seven publications) reporting HRQoL data were eligible for the SLR [12–18]. The characteristics of the included studies are presented in Table 3. Four of the studies reported eligible subgroup data (reported as individual-level patient data [IPD]) [13–16].

**Table 3:** Study and participant characteristics

Study	Study country	Study description and date of trial / study	Population	Intervention / Comparator(s)	Study / treatment duration	Number of participants	Age: minimum, maximum and mean (SD)	Sex
*HRQoL studies*								
Eom and Lee [12]	Korea	Cross-sectional study of patients recruited at the authors' institution March 2006 to February 2013	Paediatric patients with mitochondrial diseases and with the results of a neuropsychological evaluation	Intervention: NR Comparator: None	NR	70 whole sample 11 eligible patients 16 eligible mothers of these patients for total parenting stress 12 eligible mothers of these patients for maternal depression	0, 9.9 1.8 (2.5) mean age at the first symptom. Data not available for eligible population	40 boys (57%). Data not available for the eligible population
Hendrix et al. [18]	Netherlands	A retrospective cohort study including all mitochondrial disease patients who were referred to the Radboud Center for Mitochondrial Medicine between February 2014 and June 2020 All data were retrieved from the electronic patient record system from the natural history study of mitochondrial disease	Patients with mitochondrial diseases	Intervention: NR Comparator: NA	Not relevant	200 whole sample 17 eligible for current analysis	Minimum and maximum: NR Median (IQR) age: Whole sample: 45 (37–57) Eligible MERRF sample: 43 (39–52)	Whole sample: 72 males (36%) Eligible MERRF sample: 5 males (29.4%)
Koene et al. [15]	Multi-country: The Netherlands, South Africa, China (Hong Kong), Germany, USA	Multicentre study to test the feasibility, construct validity and reliability of the IPMDS Date NR	A clinically, biochemically, and genetically heterogeneous group of children and adolescents with mitochondrial diseases	Intervention: NR Comparator: None	Not relevant	17 whole sample 3 eligible for current analysis	1.6, 16 Mean age whole sample: 9.92 years Eligible sample: Male 1 was 2 years; male 2 was 12 years; and male 3 was 12 years	Whole sample: 9 males Eligible sample: 3 males
Koga et al. [14]	Japan	Pilot prospective, single-centre, exploratory, clinical study 2005–2015	Mitochondrial diseases	Intervention: SP Comparator: None	48 weeks	11 whole sample 3 eligible for current analysis	16, 62 Median age whole sample: 34.2 years Eligible sample: Age at start of the study: male was 18 years; female 1 was 22 years; and female 2 was 20 years	Whole sample: 6 males, 5 females Eligible: 2 females, 1 male
Li et al. [16]	China	A study (consecutive enrolment) of patients with PMDs and non-mitochondrial disorders enrolled at the neurology department of Children’s Hospital of Chongqing Medical University from 2015 to 2019	Patients with PMDs and non-mitochondrial disorders	Intervention: NR Comparator: NA	Not relevant	51 patients with PMDs 2 eligible patients	Minimum and maximum for patients with PMDs: 11 months, 96 months Median age Patients with PMDs: 36 months Eligible patient 1: 108 months Eligible patient 2: 101 months	Patients with PMDs M:F ratio = 1:0.7 Eligible MERRF sample: 2 females
van Kempen et al. [17]	Netherlands	A retrospective cohort study exploring the association between different mitochondrial diseases and hearing loss. Patients were recruited at the Radboud Center of Mitochondrial Medicine between 2015 and 2020	Patients with mitochondrial diseases	Intervention: NR Comparator: NA	Not relevant	62 whole sample 17 patients eligible for current analysis	Whole sample: 18, 66 Mean age: 43 years (SD: 13) Eligible MERRF sample: 7 patients (41.2%) aged 21–40; 9 patients (52.9%) aged 41–60); and 1 patient (5.9%) aged 61–80	Whole sample: 22 males (35.5%) and 40 females (64.5%) Eligible MERRF sample: 5 males (29.4%) and 12 females (70.6%)
Wang et al. [13]	China	Cohort of case series with 181 cases of genetically diagnosed Leigh/Leigh-like syndrome 2012–2019	Leigh/Leigh-like syndrome and HIBCH mutations	Intervention: Pharmacologic therapy: Antioxidants and OXPHOS complex cofactors including L-carnitine coenzyme Q10, thiamine and riboflavin, additionally including some symptomatic drugs, such as levetiracetam, or baclofen Adopting a valine-restricted diet Comparator: None	1 year	8 whole sample 3 eligible for current analysis	1, 5.7 Whole sample median: 2 years (age at diagnosis) Eligible sample: male 1 was 5 years 8 months; male 2 was 4 years; and male 3 was 1 year 2 months (age at diagnosis)	Whole sample: 4 males and 4 females Eligible: 3 males
*Healthcare resource use studies*								
Eom et al. [19]	Korea	Retrospective review of medical records of paediatric patients at the authors' hospital 2006–2015	Paediatric patients (aged less than 15 years) who were diagnosed with mitochondrial disease	Intervention: NR Comparator: None	Until patient discharge/death	31 patients with mitochondrial diseases and early death (included in the "cause of death analysis") of 221 eligible patients Leigh syndrome: 15 (48%) MELAS: 2 (7%) Non-specific mitochondrial disease: 14 (45%)	0, 7.9 Mean age at onset of symptoms: 1.8 (2.0)	17 boys (55%)
Shimizu et al. [20]	Japan	Case report Date NR	A woman diagnosed with MELAS admitted to a Japanese hospital with impaired consciousness and myoclonus	Intervention: Propofol, midazolam Comparator: None	Until patient discharge/death	1 (case report)	24	Female
Yesilbas et al. [21]	Turkey	Case report Date NR	A male patient who was referred to a paediatric intensive care unit with altered mental state, seizures, and vision loss. The patient was diagnosed with MELAS on the eighth day of hospitalisation	Intervention: The patient was extubated and non-invasive mechanical ventilatory support was initiated at the end of the third day after MELAS-specific therapies were initiated Comparator: None	Until patient discharge	1 (case report)	12	Male

*HIBCH* 3-hydroxyisobutryryl-CoA hydrolase, *HRQoL* Health-related quality of life, *IPMDS* International Paediatric Mitochondrial Disease Scale, *IQR* Interquartile range, *MELAS* Mitochondrial myopathy encephalopathy, lactic acidosis, and stroke-like episode, *NR* Not reported, *OXPHOS* Oxidative phosphorylation, *PMD* Primary mitochondrial disorder, *SP* Sodium pyruvate, *USA* United States of America

It must be noted that the patients included in van Kempen et al. [17] are likely the same as those included in Hendrix et al. [18] given the similarities in patient characteristics, study dates and recruitment. We contacted the study authors to clarify if this was the case, but we did not receive a response before completion of this SLR. Therefore, we have reported the results from both studies as separate sources of data because different eligible outcomes were reported in both studies.

Four studies were conducted in Asia (Japan, China and Korea) [12–14, 16], two in the Netherlands [17, 18], and one was a multi-country study [15]. There were two retrospective cohort studies [17, 18], one cross-sectional cohort study [12], one prospective pilot clinical study [14], one large case series cohort [13], a Delphi panel used to derive quality of life (QoL) data [15], and a retrospective age-matched cohort study [16].

Four studies enrolled paediatric patients [12, 13, 15, 16], and the other three studies recruited adult patients [14, 17, 18]; one study also recruited mothers of the paediatric patients [12]. None of the studies estimated utility data.

### HRQoL outcome data

Data for the HRQoL outcomes are shown in Table 4.

**Table 4:** HRQoL studies outcomes

Study	Population in which health effects were measured	Source of perspective of the values and population characteristics	Brief description of the tool and tool outcome grading	Total outcome data (SD)
Eom and Lee [12]	Paediatric patients with mitochondrial diseases and with the results of a neuropsychological evaluation	Patients’ perspective Eligible patients within the clinical range (IQ less than 80 and social quotient less than 70)	99 item K-CBCL for children aged 1.5–5 years and 118-item K-CBCL for children and adolescents aged 6–18 years Scores range from 0 to 100. The higher the score the more severe the disease. The clinical cut off for displaying a significant level of issues is 63	K-CBCL—total behavioural problems: 62.9 (17.1)
				K-CBCL—internalizing problems: 61.3 (16.9)
				K-CBCL—externalising problems: 57.5 (13.1)
				K-CBCL—externalising problems—withdrawn: 70.1 (17.1)
				K-CBCL—externalising problems—somatization: 56.9 (12.7)
				K-CBCL—externalising problems—anxiety/depression: 61.8 (16.7)
				K-CBCL—externalising problems—social problem: 67.8 (9.9)
				K-CBCL—externalising problems—thought problem: 58.8 (11.1)
				K-CBCL—externalising problems—attention problems: 66.0 (12.9)
				K-CBCL—externalising problems—delinquent behaviour: 55.4 (7.9)
				K-CBCL—externalising problems—aggressive behaviour: 57.9 (11.8)
				K-CBCL—externalising problems—emotional response: 63.3 (16.1)
				K-CBCL—externalising problems—sleep problems: 65.7 (15.1)
				K-CBCL—externalising problems—other problems: 59.8 (10.4)
		Parents’ perspective	A questionnaire that provides a total stress score and a score for 13 subscales across two broad domains: stress related to characteristics of the child (Child Domain) and stress related to characteristics of the parent (Parent Domain) Scores range from 0 to 100. The higher the score the more severe the disease. The clinical cut off for displaying a significant level of issues is 84	K-PSI—total parenting stress: 88.6 (9.4)
				K-PSI—child total stress: 90.1 (16.5)
				K-PSI—child stress—distractibility/hyperactivity: 68.1 (30.9)
				K-PSI—child stress—adaptability: 78.6 (31.4)
				K-PSI—child stress—reinforcement: 82.9 (17.1)
				K-PSI—child stress—demandingness: 93.2 (12.8)
				K-PSI—child stress—mood: 77.2 (28.1)
				K-PSI—child stress—acceptability: 91.4 (21.5)
				K-PSI—parent total stress: 84.1 (24.9)
				K-PSI—parent stress—competence: 87.8 (15.5)
				K-PSI—parent stress—isolation: 74.9 (28.3)
				K-PSI—parent stress—attachment: 88.0 (9.5)
				K-PSI—parent stress—health: 79.6 (20.5)
				K-PSI—parent stress—role restriction: 68.9 (29.8)
				K-PSI—parent stress—depression: 69.9 (30.3)
				K-PSI—parent stress—spouse: 65.8 (27.7)
		Mothers’ perspective	A 21-item measure of depression was used to evaluate the negative emotions experienced by the mothers Scores range from 0 to 63. The higher the score the more severe the disease. The clinical cut off for displaying a significant level of issues is 11	BDI (maternal depression): 14.6 (9.1)
	Subgroup of paediatric patients with mitochondrial diseases and intractable epilepsy	As above	As above	K-CBCL—total behavioural problems: 66.8 (16.8)
				K-CBCL—internalizing problems: 61.1 (16.4)
				K-CBCL—externalising problems: 62.6 (12.2)
		As above	As above	K-PSI—parent stress—total: 93.9 (9.5)
				K-PSI—parent stress—child total: 92.6 (10.6)
				K-PSI—parent stress—parent total: 90.1 (14.4)
		As above	As above	BDI (maternal depression): 14.7 (9.1)
Hendrix et al. [18]	Patients with mitochondrial diseases	Patients’ perspective All 17 eligible patients had MERRF	The NMDAS is a measure of disease severity. It is semi-quantitative clinical rating scale designed specifically for all forms of mitochondrial disease The NMDAS comprises four sections: current function (Section 1), system specific involvement ( Section 2), current clinical assessment ( Section 3), and QoL ( Section 4) An overall score was calculated for Sections 1–3 (maximum score 145). Section 4 is scored separately and was not reported in this study Participants were divided in subgroups based on total NMDAS: mild clinical manifestation (≤ 10), moderate disease severity (11 to 20), and severe disease severity (≥ 21)	NMDAS total: 20 (IQR: 12 to 34)
Koene et al. [15]	Patients with Leigh syndrome	Patients’ perspective Patient 1, 2 and 3 with epilepsy	The NPMDS is a semi-quantitative clinical rating scale designed specifically for all forms of mitochondrial disease The higher the score the more severe the disease (maximum score of 107 from Sections 1–3; total scores of 0 14 represent mild disease, 15–25 represent moderate disease, and > 25 represent severe disease)	NPMDS Sect. 1: 14 (NR)
				NPMDS Sect. 2: 3 (NR)
				NPMDS Sect. 3: 15 (NR)
				NPMDS total: 32 (NR)
				NPMDS Sect. 1: 6 (NR)
				NPMDS Sect. 2: 4 (NR)
				NPMDS Sect. 3: 9 (NR)
				NPMDS total: 19 (NR)
				NPMDS Sect. 1: 0 (NR)
				NPMDS Sect. 2: 0 (NR)
				NPMDS Sect. 3: 6 (NR)
				NPMDS total: 6 (NR)
Koga et al. [14]	Patients with a diagnosis of mitochondrial disease involving a known genetic abnormality and a plasma lactate concentration of > 2.5 mmol/L at rest	Patients’ perspective Of the 3 eligible patients: 1 had cardiomyopathy and 2 had end-stage MELAS	A Japanese version of the NMDAS a semi-quantitative clinical rating scale designed specifically for all forms of mitochondrial disease The higher the score the more severe the disease (maximum score 80)	JMDRS (CM patient): 65 (NR)
				JMDRS (MELAS patient 1): 70 (NR)
				JMDRS (MELAS patient 2): 70 (NR)
		Patients’ perspective Of the 3 eligible patients: 1 had cardiomyopathy and 2 had end-stage MELAS	See above Section 4 assesses QoL using the SF 12v2 Each question in the NMDAS has a possible score from 0 to 5. The higher the score the more severe the disease (maximum score 145)	NMDAS (CM patient): 108 (NR)
				NMDAS (MELAS patient 1): 134 (NR)
				NMDAS (MELAS patient 2): 141 (NR)
Li et al. [16]	Patients with primary mitochondrial disorders	Patients’ perspective Both eligible patients had MERRF	The IPMDS was used to assess the severity and natural history of patients with PMDs on the day after admission Higher scores indicate worse conditions (Koene et al., 2016) The total score is expressed as a percentage of items which were feasible to perform; therefore, the possible maximum score changes accordingly and can vary between patients [25]	IPMDS domain 1 raw score (MERRF patient 1): 9/103 (8.74%) (NR)
				IPMDS domain 2 raw score (MERRF patient 1): 4/61 (6.56%) (NR)
				IPMDS domain 3 raw score (MERRF patient 1): 28/44 (63.64%) (NR)
				IPMDS total score (MERRF patient 1): 41/208 (19.71%) (NR)
				IPMDS domain 1 raw score (MERRF patient 2): 29/103 (28.16%) (NR)
				IPMDS domain 2 raw score (MERRF patient 2): 14/63 (22.22%) (NR)
				IPMDS domain 3 raw score (MERRF patient 2): 8/59 (13.56%) (NR)
				IPMDS total score (MERRF patient 2): 51/225 (22.67%) (NR)
van Kempen et al. [17]	Patients with mitochondrial diseases	Patients’ perspective All 17 eligible patients had MERRF	See above Section 4 assess QoL using SF12v2 before 2012 and RAND SF-36 after 2012 The cognition tests performed were a Dutch equivalent of the reading test and a symbol test. The comprehension test was not used because of the absence of a Dutch equivalent Sections 1 to 3 of the NMDAS contain a total of 29 items and were scored from 0 (no involvement) to 5 (severe involvement). Maximum score 145. Scores ranged from 0 to 70 for Section 4. The sum of all four sections resulted in one mean NMDAS score Participants were divided into subgroups based on total NMDAS: mild (0–10), moderate (11–20), and severe (> 20)	Mild NMDAS score: 3 out of 15 patients (20%) (NR)
				Moderate NMDAS score: 5 out of 15 patients (33.3%) (NR)
				Severe NMDAS score: 7 out of 15 patients (46.7%) (NR)
				Mean NMDAS score for all 15 patients: 22 (NR)
Wang et al. [13]	Patients with Leigh/Leigh-like syndrome and HIBCH mutations	Patients’ perspective Of the 3 eligible patients: 1 had DD and 2 had encephalopathy	The NPMDS is a semi-quantitative clinical rating scale designed specifically for all forms of mitochondrial disease The higher the score the more severe the disease (maximum score 107 from Sections 1–3; total scores of 0–14 represent mild disease, 15–25 represent moderate disease, and > 25 represent severe disease)	NPMDS (DD patient) at peak phase: 36.2 (NR)
				NPMDS (DD patient) at last assessment: 53.1 (NR)
				NPMDS (encephalopathy patient 1) at peak disease phase (before medication): 48.8 (NR)
				NPMDS (encephalopathy patient 1) at last assessment (after medication): 38.7 (NR)
				NPMDS (encephalopathy patient 2) at peak disease phase (before medication): 44.8 (NR)
				NPMDS (encephalopathy patient 2) at last assessment (after medication): 48.4 (NR)

*BDI* Beck Depression Inventory, *CM* Cardiomyopathy, *DD* Developmental delay, *HIBCH* 3-hydroxyisobutryrly-CoA hydrolase, *HRQoL* Health-related quality of life, *IQ* Intelligence quotient, *IQR* Interquartile range, *JMDRS* Japanese Mitochondrial Disease Rating Scale, *K-CBCL* Korean Child Behaviour Check List, *K-PSI* Korean version of the Parenting Stress Index, *MELAS* Mitochondrial encephalopathy, lactic acidosis, and stroke-like episode, *NMDAS* Newcastle Mitochondrial Disease Adult Scale, *NPMDS* Newcastle Paediatric Mitochondrial Disease Scale, *NR* Not reported, *PMD* Primary mitochondrial disorder, *QoL* Quality of life, *SD* Standard deviation, *SF-12v2* Short Form 12-item version 2

### Newcastle Mitochondrial Disease Adult Scale (NMDAS)

The most reported score was the NMDAS (maximum score 145; higher scores reflected more severe disease) which was used in three studies [14, 17, 18].

In the first study, NMDAS scores ranged from 108 to 141 in three eligible patients (aged 18–22 years) [14].

In the second study, the NMDAS total score was 20 (interquartile range: 12–34) in 17 eligible patients with myoclonic epilepsy with ragged red fibres (MERRF; median age: 43 years) [18]. In this study, NMDAS scores ≤ 10 were defined as mild clinical manifestations, between 11 and 20 as moderate disease severity, and ≥ 21 as severe disease severity [18], so the MERRF subgroup was classified at the highest limit of moderate disease.

In the third study, the mean NMDAS score was 22 (standard deviation [SD]: 13.3) in 17 eligible patients with MERRF [17]. Of these 17 patients, 20% reported a mild NMDAS score (0–10), 33% reported a moderate score (11–20), and 47% reported a severe score (> 20) [17].

### NewcastlePaediatric Mitochondrial Disease Scale (NPMDS)

The NPMDS (maximum score 107) was used in two studies [13, 15]. Higher scores reflected more severe disease, with total scores > 25 indicating severe disease.

In the first study, NPMDS scores ranged from 36.2 to 48.8 at peak phase (before medication) and from 38.7 to 53.1 at last assessment in three eligible patients (age at diagnosis: 1 year 2 months to 5 years 8 months) [13].

In the second study, NPMDS total scores were 6, 20 and 32 for three eligible patients with Leigh syndrome and epilepsy (aged 2–12 years) [15]. In this study, total scores of 0–14 represented mild disease, 15–25 moderate disease, and > 25 severe disease, thus the three patients had different disease severity levels.

### Other HRQoL scores

The Japanese Mitochondrial Disease Rating Scale (JMDRS; maximum score 80; higher scores reflected more severe disease) was adopted in one study, and scores ranged from 65 to 70 in three eligible patients (aged 18–22 years) [14].

The International Paediatric Mitochondrial Disease Scale (IPMDS; higher percentage scores indicated worse conditions) was used in another study [16]. In this study, IPMDS total scores ranged between 41/208 (19.7%) and 51/225 (22.7%) for two eligible patients with MERRF (aged 101 and 108 months) [16].

The Korean Child Behaviour Check List (K-CBCL; maximum score 100; clinical cut-off score 63) was used in one study [12]. In this study, the mean K-CBCL score for total behavioural problems was 66.8 in children with mitochondrial diseases and intractable epilepsy (age not reported). The same study also reported data derived from mothers of children diagnosed with mitochondrial diseases and intractable epilepsy using the Korean version of the Parenting Stress Index (K-PSI; maximum score 100; clinical cut-off score 84) and the Beck Depression Inventory (BDI; maximum score 63; clinical cut-off score 11). For both tools, higher scores reflected more severe disease. In this study, the mean K-PSI score for total parenting stress was 93.9, and the mean BDI score for maternal depression was 14.7 [12].

### Costs and healthcare resource use studies identified and selected

**Fig. 2:**
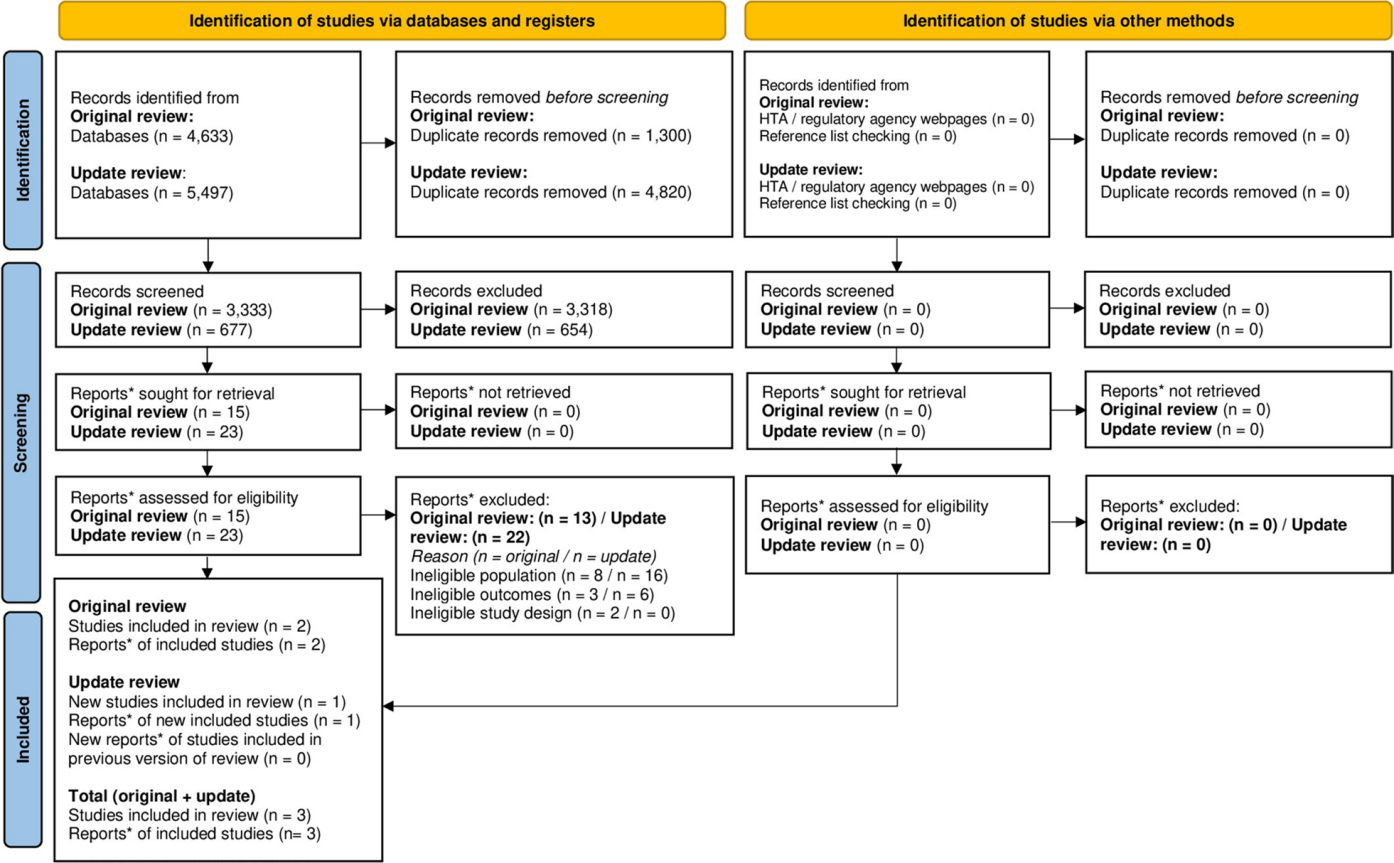
Costs and healthcare resource use data SLR PRISMA. *“Note that a “report” could be a journal article, preprint, conference abstract, study register entry, clinical study report, dissertation, unpublished manuscript, government report or any other document providing relevant information”: https://www.bmj.com/content/372/bmj.n71. *Adapted from:* Page MJ, McKenzie JE, Bossuyt PM, Boutron I, Hoffmann TC, Mulrow CD, et al. The PRISMA 2020 statement: an updated guideline for reporting systematic reviews. BMJ 2021;372:n71. https://doi.org/10.1136/bmj.n71. For more information, visit: http://www.prisma-statement.org/

The original searches identified 4,633 records. Two studies (reported in two documents) were eligible for the SLR. The update searches identified 5497 records. One study (reported in one document) was eligible for inclusion in the SLR. The PRISMA diagram is presented in Fig. 2. Lists of included and excluded studies (with reasons for exclusion) are presented in Additional file 1.

### Costs and healthcare resource use study characteristics

Three studies reporting healthcare resource use data were eligible for the SLR [19–21]. No studies reported costs data. The characteristics of the included studies are presented in Table 3. Two studies were carried out in Asia (Japan and Korea) [19, 20], and one was conducted in Turkey [21]. The studies included two case reports [20, 21] and one retrospective review of medical records [19]; all three were conducted in hospitals. Overall, limited information on healthcare resource use data (methods and results) was provided. Therefore, data from the two case reports are only presented in the narrative summary.

### Healthcare resource use outcome data

One study [19] analysed the clinical characteristics and cause of death in paediatric patients (aged < 15 years) with mitochondrial diseases to establish potential risk factors associated with mortality. For the 31 eligible patients (age: 1.8 ± 2.0 years) with mitochondrial diseases and death in hospital, the number of intensive care unit (ICU) admissions per year was 0.62 (SD: 0.86), and the mean duration of ICU stay was 14.59 days (SD: 23.68) [19]. Results by age subgroups are presented in Table 5. The mean number of causes of death per patient was 2.03 (SD: 0.91), with sepsis (17 patients), pneumonia (13 patients), disseminated intravascular coagulation (9 patients), and sudden unexpected death (9 patients) the most common causes. In addition, early death (children who died aged six years or younger) was associated with lesions in the thalamus, the number of organs involved, and Leigh syndrome [19].

**Table 5:** Healthcare resource use outcomes from Eom et al. [19]

Study	Country	Patient description	ICU usage	Source of data
Eom et al. [19]	Korea	31 Paediatric patients (aged less than 15 years) who were diagnosed with mitochondrial disease ("cause of death analysis")	Number of ICU admissions per year: Total: 0.62 ± 0.86 (range: 0–3) Patients < 6 years: 0.83 ± 1.06 Patients ≥ 6 Years: 0.38 ± 0.52	Severance Children’s Hospital, Yonsei University College of Medicine, Seoul
			Duration of ICU stay (days): Total: 14.59 ± 23.68 (range: 0–93.1) Patients < 6 years: 15.58 ± 23.1 Patients ≥ 6 years: 13.53 ± 25.03	

*ICU* Intensive care unit

Another study [20] presented a case report of a Japanese woman (aged 24 years) who was diagnosed with MELAS and admitted to hospital. The patient was transferred to ICU after 19 days of hospitalisation, and mechanical ventilation in ICU was required for 25 days.

A second case report [21] presented a 12-year-old male patient who was referred to a paediatric intensive care unit (PICU) in Turkey. The patient was diagnosed with MELAS on the eighth day of PICU hospitalisation and was intubated until three days after the start of MELAS-specific treatment, then non-invasive mechanical ventilation was initiated. The patient was discharged on the 36th day of admission. The total length of stay in PICU was not reported, but this was until at least day eight when MELAS was diagnosed.

## Discussion

The SLRs identified data on the HRQoL, utilities, costs and healthcare resource use burden in patients with mitochondrial disease with associated seizures. Overall, seven studies reporting HRQoL data and three studies reporting healthcare resource use data were identified. No studies reporting utilities or monetary costs data for this specific patient population were found in the literature. The studies included both children and adult patients, and one study also reported HRQoL data derived from mothers of children diagnosed with mitochondrial disease [12].

Mitochondrial disease is a progressive, heterogeneous group of genetic disorders, and seizures are one of the most common and severe manifestations of the disease. The presence of seizures is associated with increased morbidity and mortality and can result in a poor prognosis or epileptic encephalopathy [5] and severe disease in children [7], negatively impacting QoL and placing a burden on both patients and caregivers. The management of symptoms in these patients is challenging because seizures associated with mitochondrial disease are typically refractory to common anti-seizure therapies, and there are no approved disease-modifying treatments.

Across the included studies, HRQoL data were reported using different tools, thus limiting the comparability of results. However, despite this variability in study methods, a degree of consistency among the results was observed, with patients reporting HRQoL scores that indicate moderate to severe disease and parents scoring highly for total parenting stress.

Of the HRQoL tools used in the included studies, the NMDAS was the most commonly reported in adult patients [14, 17, 18]. In children, the NPMDS was reported in two studies [13, 15]. Data from other disease-specific tools were also reported, including the JMDRS in one study of adult patients [14] and the IPMDS in another study of paediatric patients [16]. In the final HRQoL study, Eom and Lee [12] used two other generic tools to capture behavioural data in children (K-CBCL) and its impact on mothers’ mental health (K-PSI).

Although the included studies provide insight into the HRQoL burden of patients with mitochondrial disease with associated seizures, they are limited in number and are generally based on small samples and retrospective or cross-sectional study designs. Further robust studies, with larger sample sizes and prospective study designs, are needed to fully understand the implications on HRQoL for patients and caregivers. Future work should use a common HRQoL tool to allow for consistent reporting and to enable further comparisons between studies. The NMDAS or NPMDS are the most commonly used currently in the literature.

In terms of the economic burden, only very limited non-monetary healthcare resource use data were identified, and the data available were in small and specific populations. Therefore, transferability of these data to other contexts and their usefulness for populating future economic models should be carefully assessed. For example, Eom et al. [19] investigated the reasons of early death in children with mitochondrial diseases, with only children who died included in the evaluation. This implies that the number of ICU admissions and the ICU length of stay data reported by Eom et al. [19] are likely to reflect the inherent disease severity of these patients and are unlikely to be representative of the average ICU admission and length of stay for children with mitochondrial disease with associated seizures.

Although no monetary costs data were identified for mitochondrial disease with associated seizures, previous studies have demonstrated that mitochondrial diseases more broadly (i.e. without specifying seizures) present an economic burden for both patients and healthcare systems [3, 22, 23]. Patients with mitochondrial diseases (and their families) may also experience substantial out-of-pocket expenses, as with other rare diseases, related to caregiver responsibilities, work productivity impairment, healthcare visits, and non-prescription medications [22]. The economic burden, including out-of-pocket expenses for patients and their caregivers, is potentially increased in mitochondrial disease with associated seizures. However, this is unknown given that no monetary costs data for this population were identified in the literature, and more work is needed to establish if this is the case.

### Strengths and limitations of the included studies

The strengths of the studies included the clear reporting of inclusion/exclusion criteria for patient enrolment and the explicit discussion of critical areas of the research. However, eight of the included studies were based on small samples, often as subgroups of already small cohorts with mitochondrial diseases, affecting the representativeness of the patient population and the transferability of the results to other settings. Furthermore, the epidemiological and clinical settings of some studies may not be directly applicable to populations in other areas, with potential differences in the management and treatment of these patients between countries, limiting the external validity. Further research is needed to explore the HRQoL and economic burden of mitochondrial disease with associated seizures across different countries.

The SLRs were focused on patients with mitochondrial disease with associated seizures; therefore, studies evaluating patients with mitochondrial disease, but without seizures, were not eligible and were excluded. With respect to study design, most studies were retrospective or cross-sectional, and two were single case reports. These designs are methodologically weak and lack rigour when compared with randomised controlled trials (RCTs).

### Strengths and limitations of the SLRs

Two SLRs were undertaken following systematic review guidance [8, 9], with extensive searches in several databases and information sources. The SLRs were originally conducted in 2021 and were updated in 2022.

Limitations of the SLRs include the restriction to English-language studies only and the exclusion of conference abstracts, both of which mean there is a risk of relevant research—either published in non-English language or as a conference abstract, including ongoing research—not being identified. Furthermore, although studies reporting on any non-monetary resource use data were eligible for the costs and healthcare resource use SLR, the search strategy was only designed to retrieve records that referred to a selection of non-specific healthcare resource use related terms or the following specific non-monetary resource use outcomes: hospitalisation, visits/appointments or length of stay. This approach was prospectively discussed and agreed within the research team at protocol stage, but did potentially increase the risk of not retrieving relevant data on additional specific non-monetary resource outcomes.

## Conclusions

Overall, the SLRs demonstrated the paucity of studies reporting HRQoL, utilities, costs, and healthcare resource use data in patients with mitochondrial disease with associated seizures. The HRQoL of patients and caregivers is affected with patients showing moderate to severe symptoms. No robust conclusions can be reached on resource use because of the limited data available in these patients. The limitations of the analysis are mainly related to the rarity of disease, but also to the lack of instruments that can capture the specific clinical condition that diminishes social support and family function, and presents cognitive challenges, medical and psychiatric comorbidities, and physical limitations in daily activities. Further prospective studies are needed to fully understand the HRQoL and economic burden of mitochondrial disease with associated seizures. Alongside seizures, future studies should also consider the indicators of increased severity and prognostic factors in these patients, including early age of disease onset; magnetic resonance imaging and clinical signs of brainstem involvement; and respiratory drive involvement [24].

### Supplementary Information

**Additional file 1**. Detailed search methods, supplementary tables, and PRISMA checklist.

## Data Availability

All data supporting the findings of this study are included in this published article and its supplementary information file.

## Abbreviations

ATP: Adenosine triphosphate
BDI: Beck Depression Inventory
CADTH: Canadian Agency for Drugs and Technologies in Health
HRQoL: Health-related quality of life
HTA: Health technology assessment
ICU: Intensive care unit
IPD: Individual-level patient data
IPMDS: International Paediatric Mitochondrial Disease Scale
JMDRS: Japanese Mitochondrial Disease Rating Scale
K-CBCL: Korean Child Behaviour Check List
K-PSI: Korean version of the Parenting Stress Index
MELAS: Mitochondrial encephalopathy, lactic acidosis, and stroke-like episode
MERRF: Myoclonic epilepsy with ragged red fibres
NICE: National Institute for Health and Care Excellence
NPMDS: Newcastle Paediatric Mitochondrial Disease Scale
NMDAS: Newcastle Mitochondrial Disease Adult Scale
PRISMA: Preferred Reporting Items for Systematic Reviews and Meta-Analyses
QoL: Quality of life
RCT: Randomised controlled trial
SD: Standard deviation
SLR: Systematic literature review
