# Supplementary material for: The burden of mitochondrial disease with associated seizures: systematic literature reviews of health-related quality of life, utilities, costs and healthcare resource use data
Source: Orphanet J Rare Dis. 2023 Oct 11;18:320. doi: 10.1186/s13023-023-02945-6 (PMC10568748; doi:10.1186/s13023-023-02945-6)
Supplement: Supplementary file 1 — Additional file 1. Detailed search methods, supplementary tables, and PRISMA checklist. [file 13023_2023_2945_MOESM1_ESM.docx]

**Additional file 1**

# Search methods

Two separate sets of searches were conducted to inform the two reviews. Searches were originally conducted in August 2021 and then updated in July 2022.

The original searches for the health-related quality of life (HRQoL) and utilities review were conducted between 10 August 2021 and 23 August 2021. The update searches were conducted between 25 July 2022 and 26 July 2022.

The original searches for the costs and healthcare resource use review were conducted between 11 August 2021 and 23 August 2021. The update searches were conducted between 25 July 2022 and 27 July 2022

## Search strategy

As far as possible, the search strategies used for the 2022 update searches reflected those used for the original searches, except for the addition in 2022 of the term *MDAS* to the search terms for the population concept.

### Search 1: HRQoL and utilities associated with the eligible population

The MEDLINE (OvidSP) search strategy was designed to identify studies reporting HRQoL and utility values associated with the eligible population. The final MEDLINE strategy used for the 2022 update search is presented in Supplementary Figure 1.

The strategy comprised four concepts:

- Non-specific mitochondrial disease (search lines 1 to 7).
- Specific eligible mitochondrial diseases (search lines 8 to 76).
- Health state utility values (HSUVs) (search lines 77 to 102).
- HRQoL (search lines 103 to 114).

The concepts were combined as follows: (non-specific mitochondrial disease OR specific eligible mitochondrial diseases) AND (HSUVs OR HRQoL).

The strategy was devised using a combination of subject indexing terms and free text search terms in the Title, Abstract and Keyword Heading Word fields.

The search terms for the population concepts were identified through discussions within the research team, scanning background literature, browsing database thesauri and use of the PubMed PubReMiner tool (<http://hgserver2.amc.nl/cgi-bin/miner/miner2.cgi>).

The performance of the population terms in the strategy was tested using records for the five studies included in the 2020 SLR by Chang et al. on Leigh’s disease [1] and the 72 studies included in the 2016 SLR by Anagnostou et al. on epilepsy due to mutations in the mitochondrial POLG gene [2] (77 records in total). The population terms successfully retrieved all 77 records.

The search terms for the HSUVs concept (search lines 77 to 102) included the YHEC sensitivity-maximising filter for identifying studies reporting HSUVs [3] (search lines 77 to 99). This filter was expanded by the inclusion of two additional terms the filter authors suggested may be potentially useful to enhance sensitivity (search line 100) and by additional terms for named eligible health state utility tools (search line 101).

The search terms for the HRQoL concept (search lines 103 to 114) included non-specific quality of life (QoL) terms and terms for named HRQoL tools, including those specified in the review protocol eligibility criteria.

The strategy excluded animal studies from MEDLINE using a standard algorithm (search lines 118 to 119).

Reflecting the eligibility criteria, the strategy was restricted to studies published in English language.

The final Ovid MEDLINE strategy was peer-reviewed by a second Information Specialist for errors in spelling, syntax and line combinations before it was executed.

**Supplementary Figure 1: Search strategy for MEDLINE ALL - HRQoL and utilities (2022 update search)**

1 mitochondrial diseases/ (6265)

2 mitochondrial myopathies/ or mitochondrial encephalomyopathies/ (2801)

3 (mitochondri$ adj6 (disease$ or disorder$ or syndrome$)).ti,ab,kf. (19516)

4 (mitochondri$ adj6 (cytopath$ or deficien$ or dysfunction$ or encephalomyopath$ or encephalopath$ or myopath$)).ti,ab,kf. (40843)

5 (mitochondriopath$ or (mdas not modified dental anxiety scale)).ti,ab,kf. (897)

6 (mitochondri$ adj6 (epilep$ or seizure$ or convuls$)).ti,ab,kf. (594)

7 or/1-6 (54633)

8 Leigh Disease/ (1199)

9 ((leigh$ or leigh-feigin$ or leigh-feigin-wolf$) adj6 (disease$ or disorder$ or syndrome$)).ti,ab,kf. (1740)

10 ((leigh$ or leigh-feigin$ or leigh-feigin-wolf$) adj6 (encephalopath$ or encephalomyelopath$)).ti,ab,kf. (317)

11 ((subacute or sub-acute) adj3 (necrot$ encephal$ or necrot$ juvenile encephal$ or necrot$ infantile encephal$)).ti,ab,kf. (265)

12 (leigh$ and mitochondri$).ti,ab,kf. (1261)

13 or/8-12 (2103)

14 DNA Polymerase gamma/ (847)

15 (polymerase$ adj3 gamma).ti,ab,kf. (1343)

16 (polg or polg1).ti,ab,kf. (918)

17 (polgone or polgi).ti,ab,kf. (0)

18 (mitochondri$ adj6 polymerase$).ti,ab,kf. (1911)

19 or/14-18 (3242)

20 "Diffuse Cerebral Sclerosis of Schilder"/ (2376)

21 ((alper$ or alper-huttenlocher$ or alpers-huttenlocher$) adj6 (disease$ or disorder$ or syndrome$)).ti,ab,kf. (250)

22 ((alper$ or alper-huttenlocher$ or alpers-huttenlocher$) adj6 (diffuse cerebral or diffuse degeneration or poliodystroph$)).ti,ab,kf. (19)

23 (schilder$ adj6 (disease$ or disorder$ or syndrome$)).ti,ab,kf. (182)

24 (schilder$ adj6 (diffuse cerebral or diffuse degeneration or poliodystroph$)).ti,ab,kf. (5)

25 (balo$ adj3 concentric scleros$).ti,ab,kf. (191)

26 (progressive adj3 poliodystroph$).ti,ab,kf. (18)

27 (diffuse cerebral degeneration$ or diffuse cerebral scleros$ or encephalitis periaxialis or myelinoclastic diffuse scleros$ or poliodystrophia cerebri or progressive neuronal degeneration$ or sudanophilic cerebral scleros$).ti,ab,kf. (337)

28 ahs.ti,ab,kf. (1683)

29 or/20-28 (4385)

30 myocerebrohepatopath$.ti,ab,kf. (13)

31 mchs.ti,ab,kf. (100)

32 or/30-31 (106)

33 Spinocerebellar Ataxias/ and exp Epilepsy/ (68)

34 (myoclon$ and epilep$ and sensory ataxia$).ti,ab,kf. (11)

35 memsa.ti,ab,kf. (14)

36 (spinocerebellar ataxia$ and epilep$).ti,ab,kf. (181)

37 scae.ti,ab,kf. (58)

38 or/33-37 (292)

39 Ophthalmoplegia, Chronic Progressive External/ (606)

40 external ophthalmoplegi$.ti,ab,kf. (1713)

41 ocular muscular dystroph$.ti,ab,kf. (10)

42 (peo or cpeo$ or arpeo or adpeo).ti,ab,kf. (6430)

43 ((graefe$ or graefe fuch$) adj6 (disease$ or disorder$ or syndrome$)).ti,ab,kf. (11)

44 ((graefe$ or graefe fuch$) adj6 myopath$).ti,ab,kf. (2)

45 or/39-44 (7805)

46 ataxia neuropath$.ti,ab,kf. (146)

47 ataxic neuropath$.ti,ab,kf. (261)

48 miras.ti,ab,kf. (134)

49 sando.ti,ab,kf. (73)

50 or/46-49 (572)

51 MELAS Syndrome/ (1440)

52 melas.ti,ab,kf. (2238)

53 myoencephalopath$.ti,ab,kf. (10)

54 or/51-53 (2565)

55 MERRF Syndrome/ (390)

56 merrf.ti,ab,kf. (536)

57 merff.ti,ab,kf. (13)

58 fukuhara$.ti,ab,kf. (42)

59 (epilep$ and ragged red).ti,ab,kf. (503)

60 or/55-59 (837)

61 Arginine-tRNA Ligase/ (201)

62 (mitochondri$ and arg$ and (tRNA or RNA or ribonucleic acid)).ti,ab,kf. (593)

63 (rars2 or rars-2).ti,ab,kf. (56)

64 (rarstwo or rars-two or rarsii or rars-ii or rars11 or rars-11).ti,ab,kf. (5)

65 or/61-64 (808)

66 Olivopontocerebellar Atrophies/ or (pontocerebellar adj6 hypoplasia$).ti,ab,kf. (940)

67 (pontocerebellum adj6 hypoplasia$).ti,ab,kf. (0)

68 (pch6 or pch-6).ti,ab,kf. (39)

69 (pchsix or pch-six or pchvi or pch-vi).ti,ab,kf. (1)

70 or/66-69 (958)

71 Pyruvate Dehydrogenase Complex Deficiency Disease/ (414)

72 (pyruvate dehydrogenase adj6 deficien$).ti,ab,kf. (522)

73 ((pdhc or pdh or pyruvate decarboxylase) adj6 deficien$).ti,ab,kf. (248)

74 ((ataxia or ataxic) adj6 (lactic acidosis or pyruvate or decarboxylase)).ti,ab,kf. (119)

75 or/71-74 (831)

76 7 or 13 or 19 or 29 or 32 or 38 or 45 or 50 or 54 or 60 or 65 or 70 or 75 (72991)

77 Quality-Adjusted Life Years/ (15049)

78 (quality adjusted or adjusted life year$).ti,ab,kf. (21378)

79 (qaly$ or qald$ or qale$ or qtime$).ti,ab,kf. (13479)

80 (illness state$1 or health state$1).ti,ab,kf. (7805)

81 (hui or hui1 or hui2 or hui3).ti,ab,kf. (1833)

82 (multiattribute$ or multi attribute$).ti,ab,kf. (1171)

83 (utility adj3 (score$1 or valu$ or health$ or cost$ or measur$ or disease$ or mean or gain or gains or index$)).ti,ab,kf. (18633)

84 utilities.ti,ab,kf. (8681)

85 (eq-5d or eq5d or eq-5 or eq5 or euro qual or euroqual or euro qual5d or euroqual5d or euro qol or euroqol or euro qol5d or euroqol5d or euro quol or euroquol or euro quol5d or euroquol5d or eur qol or eurqol or eur qol5d or eur qol5d or eur?qul or eur?qul5d or euro$ quality of life or european qol).ti,ab,kf. (15512)

86 (euro$ adj3 (5 d or 5d or 5 dimension$ or 5dimension$ or 5 domain$ or 5domain$)).ti,ab,kf. (5389)

87 (sf36$ or sf 36$ or sf thirtysix or sf thirty six).ti,ab,kf. (25319)

88 (time trade off$1 or time tradeoff$1 or tto or timetradeoff$1).ti,ab,kf. (2225)

89 quality of life/ and ((quality of life or qol) adj (score$1 or measure$1)).ti,ab,kf. (14571)

90 quality of life/ and ec.fs. (10872)

91 quality of life/ and (health adj3 status).ti,ab,kf. (11072)

92 (quality of life or qol).ti,ab,kf. and Cost-Benefit Analysis/ (15986)

93 ((qol or hrqol or quality of life).ti,kf. or *quality of life/) and ((qol or hrqol$ or quality of life) adj2 (increas$ or decrease$ or improv$ or declin$ or reduc$ or high$ or low$ or effect or effects or worse or score or scores or change$1 or impact$1 or impacted or deteriorat$)).ab. (48808)

94 Cost-Benefit Analysis/ and (cost-effectiveness ratio$ and (perspective$ or life expectanc$)).ti,ab,kf. (4812)

95 *quality of life/ and (quality of life or qol).ti. (62435)

96 quality of life/ and ((quality of life or qol) adj3 (improv$ or chang$)).ti,ab,kf. (37342)

97 quality of life/ and health-related quality of life.ti,ab,kf. (41448)

98 models,economic/ (11016)

99 or/77-98 (205171)

100 (utility loss$ or disutility$).ti,ab,kf. (563)

101 (ahum or aqol$ or chu9d or chu-9d or eq-5dy or eq5dy or health status classification system$ or hscs-ps or hscsps or hui or hui1 or hui2 or hui3 or quality of well-being or quality of wellbeing or qwb or 15d or 15-d or 15 dimension$ or 16d or 16-d or 16 dimension$ or 17d or 17-d or 17 dimension$).ti,ab,kf. (12577)

102 99 or 100 or 101 (214987)

103 "Quality of Life"/ (248211)

104 ((quality adj3 life) or qol).ti,ab,kf. (359744)

105 (hql or hqol or hrqol or hrql or hr-ql).ti,ab,kf. (25009)

106 (short form$ or shortform$).ti,ab,kf. (40947)

107 (sf6 or sf-6 or sf6d or sf-6d or sf-six or sfsix).ti,ab,kf. (3202)

108 (sf8 or sf-8 or sf-eight or sfeight).ti,ab,kf. (589)

109 (sf12 or sf-12 or sf-twelve or sftwelve).ti,ab,kf. (5847)

110 (sf16 or sf-16 or sf-sixteen or sfsixteen).ti,ab,kf. (32)

111 (sf20 or sf-20 or sf-twenty or sftwenty).ti,ab,kf. (352)

112 or/103-111 (444720)

113 (attitudes to children with epilepsy$ or aatcwe$ or disabkids$ or eldqol$ or "epilepsy and children questionnaire$" or ecq$ or "epilepsy foundation$ of america concern$ index$" or efa or efaci or glasgow epilepsy outcome scale$ or geos$ or hague restrictions in childhood epilepsy scale$ or harces$ or cheqol$ or modified impact of epilepsy schedule$ or mioes or impact of childhood illness scale$ or ici$ or impact of pediatric epilepsy scale$ or impact of paediatric epilepsy scale$ or ipes$ or impact of childhood neurologic disability$ or icnd$ or neuroqol$ or newcastle mitochondrial disease$ or nmds$ or newcastle adult mitochondrial disease$ or nmdas$ or nmdsa$ or newcastle pediatric mitochondrial disease$ or newcastle paediatric mitochondrial disease$ or npmds or pedsql$ or peds-ql$ or pedsqlem$ or peds-qlem$ or pediatric epilepsy side effects questionnaire$ or paediatric epilepsy side effects questionnaire$ or pesq$ or patient-reported outcomes measurement information system$ or promis or promis25 or promis37 or promis49 or qolce$ or gqolce$ or qolie$ or qolpes$).ti,ab,kf. (43983)

114 112 or 113 (482293)

115 76 and 102 (156)

116 76 and 114 (656)

117 115 or 116 (709)

118 exp Animals/ not Humans/ (5039010)

119 117 not 118 (644)

120 limit 119 to english language (589)

Key to Ovid symbols and commands:

$ Unlimited right-hand truncation symbol

$N Limited right-hand truncation - restricts the number of characters following the word to N

? Wildcard symbol

ti,ab,kf. Searches are restricted to the Title (ti), Abstract (ab), Keyword Heading Word (kf), fields

adj Retrieves records that contain terms next to each other (in the shown order)

adjN Retrieves records that contain terms (in any order) within a specified number (N) of words of each other

/ Searches are restricted to the Subject Heading field

exp The subject heading is exploded

* The subject heading is searched as a major descriptor only

pt. Search is restricted to the publication type field

or/1-6 Combines sets 1 to 6 using OR

.fs. Term is searched as a floating subheading

### Search 2: Costs and healthcare resource use associated with the eligible population

The MEDLINE (OvidSP) search strategy was designed to identify studies reporting costs and healthcare resource use associated with the eligible population. The final MEDLINE strategy used for the 2022 update search is presented in Supplementary Figure *2*.

The strategy comprised four concepts:

- Non-specific mitochondrial disease (search lines 1 to 7).
- Specific eligible mitochondrial diseases (search lines 8 to 76).
- Monetary costs (search lines 77 to 104).
- Non-monetary healthcare resource use (search lines 105 to 121).

The concepts were combined as follows: (non-specific mitochondrial disease OR specific eligible mitochondrial diseases) AND (monetary costs OR non-monetary healthcare resource use).

The strategy was devised using a combination of subject indexing terms and free text search terms in the Title, Abstract and Keyword Heading Word fields.

The search terms for the population concepts reflected those used in the HRQoL and utilities search strategy.

The search terms for the monetary costs concept (search lines 77 to 104) included the filter developed by the University of York Centre for Reviews and Dissemination (CRD) for identification of economic evaluations to include in NHS Economic Evaluation Database (NHS EED) (search lines 77 to 93) [4]. The CRD filter was specifically designed to identify economic evaluations, rather than all monetary cost studies. The CRD filter terms were, therefore, enriched with additional monetary cost-related terms (search lines 94 to 103).

The search terms for the non-monetary healthcare resource use concept (search lines 105 to 121) were designed to retrieve database records explicitly referring to a selection of non-specific healthcare resource use related terms (for example, ‘resource use’, 'healthcare utilisation', ‘burden of illness’) or the following specific non-monetary resource use outcomes: hospitalisation, visits/appointments or length of stay. This approach was discussed and agreed within the research team.

The strategy excluded animal studies from MEDLINE using a standard algorithm (search lines 125 to 126).

Reflecting the eligibility criteria, the strategy was restricted to studies published in English language.

The final Ovid MEDLINE strategy was peer-reviewed by a second Information Specialist for errors in spelling, syntax and line combinations before it was executed.

**Supplementary Figure 2: Search strategy for MEDLINE ALL - costs and healthcare resource use (2022 update search)**

1 mitochondrial diseases/ (6271)

2 mitochondrial myopathies/ or mitochondrial encephalomyopathies/ (2801)

3 (mitochondri$ adj6 (disease$ or disorder$ or syndrome$)).ti,ab,kf. (19539)

4 (mitochondri$ adj6 (cytopath$ or deficien$ or dysfunction$ or encephalomyopath$ or encephalopath$ or myopath$)).ti,ab,kf. (40895)

5 (mitochondriopath$ or (mdas not modified dental anxiety scale)).ti,ab,kf. (899)

6 (mitochondri$ adj6 (epilep$ or seizure$ or convuls$)).ti,ab,kf. (594)

7 or/1-6 (54693)

8 Leigh Disease/ (1200)

9 ((leigh$ or leigh-feigin$ or leigh-feigin-wolf$) adj6 (disease$ or disorder$ or syndrome$)).ti,ab,kf. (1741)

10 ((leigh$ or leigh-feigin$ or leigh-feigin-wolf$) adj6 (encephalopath$ or encephalomyelopath$)).ti,ab,kf. (317)

11 ((subacute or sub-acute) adj3 (necrot$ encephal$ or necrot$ juvenile encephal$ or necrot$ infantile encephal$)).ti,ab,kf. (265)

12 (leigh$ and mitochondri$).ti,ab,kf. (1262)

13 or/8-12 (2104)

14 DNA Polymerase gamma/ (847)

15 (polymerase$ adj3 gamma).ti,ab,kf. (1343)

16 (polg or polg1).ti,ab,kf. (918)

17 (polgone or polgi).ti,ab,kf. (0)

18 (mitochondri$ adj6 polymerase$).ti,ab,kf. (1912)

19 or/14-18 (3243)

20 "Diffuse Cerebral Sclerosis of Schilder"/ (2376)

21 ((alper$ or alper-huttenlocher$ or alpers-huttenlocher$) adj6 (disease$ or disorder$ or syndrome$)).ti,ab,kf. (250)

22 ((alper$ or alper-huttenlocher$ or alpers-huttenlocher$) adj6 (diffuse cerebral or diffuse degeneration or poliodystroph$)).ti,ab,kf. (19)

23 (schilder$ adj6 (disease$ or disorder$ or syndrome$)).ti,ab,kf. (182)

24 (schilder$ adj6 (diffuse cerebral or diffuse degeneration or poliodystroph$)).ti,ab,kf. (5)

25 (balo$ adj3 concentric scleros$).ti,ab,kf. (191)

26 (progressive adj3 poliodystroph$).ti,ab,kf. (18)

27 (diffuse cerebral degeneration$ or diffuse cerebral scleros$ or encephalitis periaxialis or myelinoclastic diffuse scleros$ or poliodystrophia cerebri or progressive neuronal degeneration$ or sudanophilic cerebral scleros$).ti,ab,kf. (337)

28 ahs.ti,ab,kf. (1684)

29 or/20-28 (4386)

30 myocerebrohepatopath$.ti,ab,kf. (13)

31 mchs.ti,ab,kf. (100)

32 or/30-31 (106)

33 Spinocerebellar Ataxias/ and exp Epilepsy/ (68)

34 (myoclon$ and epilep$ and sensory ataxia$).ti,ab,kf. (11)

35 memsa.ti,ab,kf. (14)

36 (spinocerebellar ataxia$ and epilep$).ti,ab,kf. (181)

37 scae.ti,ab,kf. (58)

38 or/33-37 (292)

39 Ophthalmoplegia, Chronic Progressive External/ (606)

40 external ophthalmoplegi$.ti,ab,kf. (1713)

41 ocular muscular dystroph$.ti,ab,kf. (10)

42 (peo or cpeo$ or arpeo or adpeo).ti,ab,kf. (6436)

43 ((graefe$ or graefe fuch$) adj6 (disease$ or disorder$ or syndrome$)).ti,ab,kf. (11)

44 ((graefe$ or graefe fuch$) adj6 myopath$).ti,ab,kf. (2)

45 or/39-44 (7811)

46 ataxia neuropath$.ti,ab,kf. (146)

47 ataxic neuropath$.ti,ab,kf. (261)

48 miras.ti,ab,kf. (134)

49 sando.ti,ab,kf. (73)

50 or/46-49 (572)

51 MELAS Syndrome/ (1440)

52 melas.ti,ab,kf. (2239)

53 myoencephalopath$.ti,ab,kf. (10)

54 or/51-53 (2566)

55 MERRF Syndrome/ (390)

56 merrf.ti,ab,kf. (537)

57 merff.ti,ab,kf. (13)

58 fukuhara$.ti,ab,kf. (42)

59 (epilep$ and ragged red).ti,ab,kf. (504)

60 or/55-59 (838)

61 Arginine-tRNA Ligase/ (201)

62 (mitochondri$ and arg$ and (tRNA or RNA or ribonucleic acid)).ti,ab,kf. (593)

63 (rars2 or rars-2).ti,ab,kf. (56)

64 (rarstwo or rars-two or rarsii or rars-ii or rars11 or rars-11).ti,ab,kf. (5)

65 or/61-64 (808)

66 Olivopontocerebellar Atrophies/ or (pontocerebellar adj6 hypoplasia$).ti,ab,kf. (940)

67 (pontocerebellum adj6 hypoplasia$).ti,ab,kf. (0)

68 (pch6 or pch-6).ti,ab,kf. (39)

69 (pchsix or pch-six or pchvi or pch-vi).ti,ab,kf. (1)

70 or/66-69 (958)

71 Pyruvate Dehydrogenase Complex Deficiency Disease/ (415)

72 (pyruvate dehydrogenase adj6 deficien$).ti,ab,kf. (522)

73 ((pdhc or pdh or pyruvate decarboxylase) adj6 deficien$).ti,ab,kf. (248)

74 ((ataxia or ataxic) adj6 (lactic acidosis or pyruvate or decarboxylase)).ti,ab,kf. (119)

75 or/71-74 (831)

76 7 or 13 or 19 or 29 or 32 or 38 or 45 or 50 or 54 or 60 or 65 or 70 or 75 (73057)

77 Economics/ (27457)

78 exp "costs and cost analysis"/ (259838)

79 Economics, Dental/ (1920)

80 exp economics, hospital/ (25616)

81 Economics, Medical/ (9229)

82 Economics, Nursing/ (4013)

83 Economics, Pharmaceutical/ (3077)

84 (economic$ or cost or costs or costly or costing or price or prices or pricing or pharmacoeconomic$).ti,ab. (964965)

85 (expenditure$ not energy).ti,ab. (34826)

86 value for money.ti,ab. (1999)

87 budget$.ti,ab. (33632)

88 or/77-87 (1126780)

89 ((energy or oxygen) adj cost).ti,ab. (4586)

90 (metabolic adj cost).ti,ab. (1622)

91 ((energy or oxygen) adj expenditure).ti,ab. (27954)

92 or/89-91 (33122)

93 88 not 92 (1119153)

94 exp Budgets/ (14042)

95 exp models, economic/ (16135)

96 "Value of Life"/ (5794)

97 ec.fs. (442237)

98 Income/ (33346)

99 Remuneration/ (345)

100 "Salaries and Fringe Benefits"/ (16176)

101 exp "Fees and Charges"/ (31185)

102 (earn$ or expens$ or fee or fees or financ$ or fiscal$ or income$1 or money$ or monetary or paid or pay or pays or paying or payment$1 or remunerat$ or salar$ or wage$1).ti,ab,kf. (585663)

103 or/94-102 (946825)

104 93 or 103 (1688243)

105 Health Resources/ or "Supply & Distribution".fs. or exp Resource Allocation/ (100032)

106 (burden$ or resource$1).ti. (90306)

107 (burden$ adj6 (care or caring or disease$ or healthcare or illness$ or sickness$ or therap$ or treatment$)).ti,ab,kf. (87926)

108 (((resource$1 or healthcare or health-care) adj6 (allocat$ or consum$ or ration$ or usage$ or use$1 or utilis$ or utiliz$)) or hcru).ti,ab,kf. (163800)

109 Office Visits/sn, td or "Facilities and Services Utilization"/ or "Equipment and Supplies Utilization"/ or "Procedures and Techniques Utilization"/ (5292)

110 (visit or visits or visited or visiting).ti,ab,kf. (270156)

111 appointment$.ti,ab,kf. (29961)

112 Hospitalization/ (129601)

113 (hospitalization$1 or hospitalisation$1 or hospitalised or hospitalized).ti,ab,kf. (307527)

114 (admission$1 or readmission$1 or admitted or readmitted).ti,ab,kf. (468932)

115 "length of stay"/ (100209)

116 hospital stay$1.ti,ab,kf. (99943)

117 (bed adj3 day$1).ti,ab,kf. (4014)

118 ((days or time or length or duration$1) adj3 hospital$).ti,ab,kf. (111854)

119 ((days or time or length or duration$1) adj3 (stay or stays or stayed)).ti,ab,kf. (125981)

120 ((days or time or length or duration$1) adj3 (discharge or discharged or home or homes)).ti,ab,kf. (29378)

121 or/105-120 (1464376)

122 76 and 104 (1219)

123 76 and 121 (1312)

124 122 or 123 (2404)

125 exp Animals/ not Humans/ (5040469)

126 124 not 125 (2189)

127 limit 126 to english language (1913)

Key to Ovid symbols and commands:

$ Unlimited right-hand truncation symbol

$N Limited right-hand truncation - restricts the number of characters following the word to N

? Wildcard symbol

ti,ab,kf. Searches are restricted to the Title (ti), Abstract (ab), Keyword Heading Word (kf), fields

adj Retrieves records that contain terms next to each other (in the shown order)

adjN Retrieves records that contain terms (in any order) within a specified number (N) of words of each other

/ Searches are restricted to the Subject Heading field

exp The subject heading is exploded

pt. Search is restricted to the publication type field

or/1-6 Combines sets 1 to 6 using OR

.fs. Term is searched as a floating subheading

## Resources searched

We conducted the literature search in the databases and information resources shown in Table 2 of the manuscript. The same resources were searched for both the original 2021 searches and the 2022 update searches, with the exception of NHS EED which was not searched for the 2022 update. No update search of NHS EED was required for the 2022 search (NHS EED is a closed database to which new records were last added in 2015).

In addition to searching the HTA Database, targeted searches of the listed technology assessment and regulatory agency websites were conducted:

- National Institute for Health and Care Excellence (NICE) webpages for company submissions to NICE, Final Appraisal Determination documents, Evidence Review Group reports (for single technology assessments), and assessment reports (for multiple technology appraisals).
- CADTH webpages for CADTH Economic Guidance and CADTH Final Recommendations associated with Reimbursement Reviews.
- ICER webpages for Evidence Reports.

Reflecting the eligibility criteria, records in Embase indexed as conference abstracts were excluded.

We also checked the reference lists of any included studies and retrieved relevant SLRs published from 2016 for eligible studies that may have been missed by the database searches.

## Running the search strategies and downloading results

For each review, we conducted searches using each database or resource listed above, translating the agreed Ovid MEDLINE strategy appropriately. Translation included consideration of differences in database interfaces and functionality, in addition to variation in indexing languages and thesauri. As far as possible the translations for the 2022 update searches reflected those used for the original 2021 searches.

For the 2022 update searches, the database searches were re-run in full. The searches of agency websites were restricted to the identification of documents published since the date of the original 2021 search.

The full search strategies used for both the original 2021 searches and the 2022 update searches are presented below (Section 1.4).

The searches for each review were conducted separately. Where possible, we downloaded the results of searches in a tagged format and loaded them into bibliographic software (EndNote) [5] for deduplication. Results for each review were processed separately, using separate EndNote libraries, with two separate sets of results produced for screening.

For the original searches, results were downloaded into an empty EndNote library and deduplicated using several algorithms (duplicate references were held in a separate EndNote database for checking if required). For the update searches, results were downloaded into an EndNote Library containing the results retrieved for the original searches and deduplicated within-set and against the original search results using several algorithms (duplicate references were held in a separate EndNote database for checking if required). Results from resources that did not allow export in a format compatible with EndNote were saved in Word or Excel documents as appropriate and manually deduplicated.

## Full search strategies

### HRQoL / Utilities Full Search Strategies

#### Original searches: August 2021

1. **Source: MEDLINE ALL**

Interface / URL: OvidSP

Database coverage dates: 1946 to August 06, 2021

Search date: 10/08/2021

Retrieved records: 483

Search strategy:

1 mitochondrial diseases/ (5731)

2 mitochondrial myopathies/ or mitochondrial encephalomyopathies/ (2704)

3 (mitochondri$ adj6 (disease$ or disorder$ or syndrome$)).ti,ab,kf. (17738)

4 (mitochondri$ adj6 (cytopath$ or deficien$ or dysfunction$ or encephalomyopath$ or encephalopath$ or myopath$)).ti,ab,kf. (36633)

5 mitochondriopath$.ti,ab,kf. (347)

6 (mitochondri$ adj6 (epilep$ or seizure$ or convuls$)).ti,ab,kf. (559)

7 or/1-6 (48878)

8 Leigh Disease/ (1121)

9 ((leigh$ or leigh-feigin$ or leigh-feigin-wolf$) adj6 (disease$ or disorder$ or syndrome$)).ti,ab,kf. (1624)

10 ((leigh$ or leigh-feigin$ or leigh-feigin-wolf$) adj6 (encephalopath$ or encephalomyelopath$)).ti,ab,kf. (304)

11 ((subacute or sub-acute) adj3 (necrot$ encephal$ or necrot$ juvenile encephal$ or necrot$ infantile encephal$)).ti,ab,kf. (262)

12 (leigh$ and mitochondri$).ti,ab,kf. (1159)

13 or/8-12 (1984)

14 DNA Polymerase gamma/ (803)

15 (polymerase$ adj3 gamma).ti,ab,kf. (1310)

16 (polg or polg1).ti,ab,kf. (857)

17 (polgone or polgi).ti,ab,kf. (0)

18 (mitochondri$ adj6 polymerase$).ti,ab,kf. (1838)

19 or/14-18 (3108)

20 "Diffuse Cerebral Sclerosis of Schilder"/ (2365)

21 ((alper$ or alper-huttenlocher$ or alpers-huttenlocher$) adj6 (disease$ or disorder$ or syndrome$)).ti,ab,kf. (244)

22 ((alper$ or alper-huttenlocher$ or alpers-huttenlocher$) adj6 (diffuse cerebral or diffuse degeneration or poliodystroph$)).ti,ab,kf. (19)

23 (schilder$ adj6 (disease$ or disorder$ or syndrome$)).ti,ab,kf. (180)

24 (schilder$ adj6 (diffuse cerebral or diffuse degeneration or poliodystroph$)).ti,ab,kf. (5)

25 (balo$ adj3 concentric scleros$).ti,ab,kf. (185)

26 (progressive adj3 poliodystroph$).ti,ab,kf. (18)

27 (diffuse cerebral degeneration$ or diffuse cerebral scleros$ or encephalitis periaxialis or myelinoclastic diffuse scleros$ or poliodystrophia cerebri or progressive neuronal degeneration$ or sudanophilic cerebral scleros$).ti,ab,kf. (335)

28 ahs.ti,ab,kf. (1571)

29 or/20-28 (4256)

30 myocerebrohepatopath$.ti,ab,kf. (11)

31 mchs.ti,ab,kf. (94)

32 or/30-31 (98)

33 Spinocerebellar Ataxias/ and exp Epilepsy/ (67)

34 (myoclon$ and epilep$ and sensory ataxia$).ti,ab,kf. (10)

35 memsa.ti,ab,kf. (14)

36 (spinocerebellar ataxia$ and epilep$).ti,ab,kf. (164)

37 scae.ti,ab,kf. (54)

38 or/33-37 (270)

39 Ophthalmoplegia, Chronic Progressive External/ (583)

40 external ophthalmoplegi$.ti,ab,kf. (1667)

41 ocular muscular dystroph$.ti,ab,kf. (10)

42 (peo or cpeo$ or arpeo or adpeo).ti,ab,kf. (5882)

43 ((graefe$ or graefe fuch$) adj6 (disease$ or disorder$ or syndrome$)).ti,ab,kf. (11)

44 ((graefe$ or graefe fuch$) adj6 myopath$).ti,ab,kf. (2)

45 or/39-44 (7219)

46 ataxia neuropath$.ti,ab,kf. (128)

47 ataxic neuropath$.ti,ab,kf. (260)

48 miras.ti,ab,kf. (131)

49 sando.ti,ab,kf. (73)

50 or/46-49 (550)

51 MELAS Syndrome/ (1368)

52 melas.ti,ab,kf. (2140)

53 myoencephalopath$.ti,ab,kf. (10)

54 or/51-53 (2461)

55 MERRF Syndrome/ (384)

56 merrf.ti,ab,kf. (528)

57 merff.ti,ab,kf. (13)

58 fukuhara$.ti,ab,kf. (40)

59 (epilep$ and ragged red).ti,ab,kf. (498)

60 or/55-59 (823)

61 Arginine-tRNA Ligase/ (192)

62 (mitochondri$ and arg$ and (tRNA or RNA or ribonucleic acid)).ti,ab,kf. (553)

63 (rars2 or rars-2).ti,ab,kf. (51)

64 (rarstwo or rars-two or rarsii or rars-ii or rars11 or rars-11).ti,ab,kf. (5)

65 or/61-64 (763)

66 Olivopontocerebellar Atrophies/ or (pontocerebellar adj6 hypoplasia$).ti,ab,kf. (901)

67 (pontocerebellum adj6 hypoplasia$).ti,ab,kf. (0)

68 (pch6 or pch-6).ti,ab,kf. (35)

69 (pchsix or pch-six or pchvi or pch-vi).ti,ab,kf. (1)

70 or/66-69 (918)

71 Pyruvate Dehydrogenase Complex Deficiency Disease/ (406)

72 (pyruvate dehydrogenase adj6 deficien$).ti,ab,kf. (509)

73 ((pdhc or pdh or pyruvate decarboxylase) adj6 deficien$).ti,ab,kf. (244)

74 ((ataxia or ataxic) adj6 (lactic acidosis or pyruvate or decarboxylase)).ti,ab,kf. (113)

75 or/71-74 (811)

76 7 or 13 or 19 or 29 or 32 or 38 or 45 or 50 or 54 or 60 or 65 or 70 or 75 (66276)

77 Quality-Adjusted Life Years/ (13563)

78 (quality adjusted or adjusted life year$).ti,ab,kf. (19090)

79 (qaly$ or qald$ or qale$ or qtime$).ti,ab,kf. (12140)

80 (illness state$1 or health state$1).ti,ab,kf. (7189)

81 (hui or hui1 or hui2 or hui3).ti,ab,kf. (1700)

82 (multiattribute$ or multi attribute$).ti,ab,kf. (1019)

83 (utility adj3 (score$1 or valu$ or health$ or cost$ or measur$ or disease$ or mean or gain or gains or index$)).ti,ab,kf. (16962)

84 utilities.ti,ab,kf. (7925)

85 (eq-5d or eq5d or eq-5 or eq5 or euro qual or euroqual or euro qual5d or euroqual5d or euro qol or euroqol or euro qol5d or euroqol5d or euro quol or euroquol or euro quol5d or euroquol5d or eur qol or eurqol or eur qol5d or eur qol5d or eur?qul or eur?qul5d or euro$ quality of life or european qol).ti,ab,kf. (13549)

86 (euro$ adj3 (5 d or 5d or 5 dimension$ or 5dimension$ or 5 domain$ or 5domain$)).ti,ab,kf. (4734)

87 (sf36$ or sf 36$ or sf thirtysix or sf thirty six).ti,ab,kf. (23797)

88 (time trade off$1 or time tradeoff$1 or tto or timetradeoff$1).ti,ab,kf. (2054)

89 quality of life/ and ((quality of life or qol) adj (score$1 or measure$1)).ti,ab,kf. (12952)

90 quality of life/ and ec.fs. (10652)

91 quality of life/ and (health adj3 status).ti,ab,kf. (9919)

92 (quality of life or qol).ti,ab,kf. and Cost-Benefit Analysis/ (14220)

93 ((qol or hrqol or quality of life).ti,kf. or *quality of life/) and ((qol or hrqol$ or quality of life) adj2 (increas$ or decrease$ or improv$ or declin$ or reduc$ or high$ or low$ or effect or effects or worse or score or scores or change$1 or impact$1 or impacted or deteriorat$)).ab. (43223)

94 Cost-Benefit Analysis/ and (cost-effectiveness ratio$ and (perspective$ or life expectanc$)).ti,ab,kf. (4101)

95 *quality of life/ and (quality of life or qol).ti. (58447)

96 quality of life/ and ((quality of life or qol) adj3 (improv$ or chang$)).ti,ab,kf. (31686)

97 quality of life/ and health-related quality of life.ti,ab,kf. (36387)

98 models,economic/ (10667)

99 or/77-98 (184879)

100 (utility loss$ or disutility$).ti,ab,kf. (517)

101 (ahum or aqol$ or chu9d or chu-9d or eq-5dy or eq5dy or health status classification system$ or hscs-ps or hscsps or hui or hui1 or hui2 or hui3 or quality of well-being or quality of wellbeing or qwb or 15d or 15-d or 15 dimension$ or 16d or 16-d or 16 dimension$ or 17d or 17-d or 17 dimension$).ti,ab,kf. (11954)

102 99 or 100 or 101 (194278)

103 "Quality of Life"/ (218063)

104 ((quality adj3 life) or qol).ti,ab,kf. (325509)

105 (hql or hqol or hrqol or hrql or hr-ql).ti,ab,kf. (22631)

106 (short form$ or shortform$).ti,ab,kf. (37362)

107 (sf6 or sf-6 or sf6d or sf-6d or sf-six or sfsix).ti,ab,kf. (3006)

108 (sf8 or sf-8 or sf-eight or sfeight).ti,ab,kf. (543)

109 (sf12 or sf-12 or sf-twelve or sftwelve).ti,ab,kf. (5331)

110 (sf16 or sf-16 or sf-sixteen or sfsixteen).ti,ab,kf. (30)

111 (sf20 or sf-20 or sf-twenty or sftwenty).ti,ab,kf. (344)

112 or/103-111 (406010)

113 (attitudes to children with epilepsy$ or aatcwe$ or disabkids$ or eldqol$ or "epilepsy and children questionnaire$" or ecq$ or "epilepsy foundation$ of america concern$ index$" or efa or efaci or glasgow epilepsy outcome scale$ or geos$ or hague restrictions in childhood epilepsy scale$ or harces$ or cheqol$ or modified impact of epilepsy schedule$ or mioes or impact of childhood illness scale$ or ici$ or impact of Paediatric epilepsy scale$ or impact of paediatric epilepsy scale$ or ipes$ or impact of childhood neurologic disability$ or icnd$ or neuroqol$ or newcastle mitochondrial disease$ or nmds$ or newcastle adult mitochondrial disease$ or nmdas$ or nmdsa$ or newcastle Paediatric mitochondrial disease$ or newcastle paediatric mitochondrial disease$ or npmds or pedsql$ or peds-ql$ or pedsqlem$ or peds-qlem$ or Paediatric epilepsy side effects questionnaire$ or paediatric epilepsy side effects questionnaire$ or pesq$ or patient-reported outcomes measurement information system$ or promis or promis25 or promis37 or promis49 or qolce$ or gqolce$ or qolie$ or qolpes$).ti,ab,kf. (37098)

114 112 or 113 (437617)

115 76 and 102 (124)

116 76 and 114 (541)

117 115 or 116 (588)

118 exp Animals/ not Humans/ (4870600)

119 117 not 118 (531)

120 limit 119 to english language (483)

1. **Source: Embase**

Interface / URL: OvidSP

Database coverage dates: 1974 to 2021 August 10

Search date: 10/08/2021

Retrieved records: 805

Search strategy:

1 "disorders of mitochondrial functions"/ (23247)

2 mitochondrial dna disorder/ or mitochondrial encephalomyopathy/ or mitochondrial encephalopathy/ or mitochondrial myopathy/ (4913)

3 (mitochondri$ adj6 (disease$ or disorder$ or syndrome$)).ti,ab,kw,dq. (25044)

4 (mitochondri$ adj6 (cytopath$ or deficien$ or dysfunction$ or encephalomyopath$ or encephalopath$ or myopath$)).ti,ab,kw,dq. (49421)

5 mitochondriopath$.ti,ab,kw,dq. (558)

6 (mitochondri$ adj6 (epilep$ or seizure$ or convuls$)).ti,ab,kw,dq. (822)

7 or/1-6 (71703)

8 Leigh disease/ (2684)

9 ((leigh$ or leigh-feigin$ or leigh-feigin-wolf$) adj6 (disease$ or disorder$ or syndrome$)).ti,ab,kw,dq. (2293)

10 ((leigh$ or leigh-feigin$ or leigh-feigin-wolf$) adj6 (encephalopath$ or encephalomyelopath$)).ti,ab,kw,dq. (377)

11 ((subacute or sub-acute) adj3 (necrot$ encephal$ or necrot$ juvenile encephal$ or necrot$ infantile encephal$)).ti,ab,kw,dq. (260)

12 (leigh$ and mitochondri$).ti,ab,kw,dq. (1756)

13 or/8-12 (3248)

14 DNA directed DNA polymerase gamma/ (2214)

15 (polymerase$ adj3 gamma).ti,ab,kw,dq. (1610)

16 (polg or polg1).ti,ab,kw,dq. (1533)

17 (polgone or polgi).ti,ab,kw,dq. (4)

18 (mitochondri$ adj6 polymerase$).ti,ab,kw,dq. (2205)

19 or/14-18 (5211)

20 Alpers disease/ or Schilder disease/ (710)

21 ((alper$ or alper-huttenlocher$ or alpers-huttenlocher$) adj6 (disease$ or disorder$ or syndrome$)).ti,ab,kw,dq. (369)

22 ((alper$ or alper-huttenlocher$ or alpers-huttenlocher$) adj6 (diffuse cerebral or diffuse degeneration or poliodystroph$)).ti,ab,kw,dq. (17)

23 (schilder$ adj6 (disease$ or disorder$ or syndrome$)).ti,ab,kw,dq. (176)

24 (schilder$ adj6 (diffuse cerebral or diffuse degeneration or poliodystroph$)).ti,ab,kw,dq. (12)

25 (balo$ adj3 concentric scleros$).ti,ab,kw,dq. (270)

26 (progressive adj3 poliodystroph$).ti,ab,kw,dq. (15)

27 (diffuse cerebral degeneration$ or diffuse cerebral scleros$ or encephalitis periaxialis or myelinoclastic diffuse scleros$ or poliodystrophia cerebri or progressive neuronal degeneration$ or sudanophilic cerebral scleros$).ti,ab,kw,dq. (227)

28 ahs.ti,ab,kw,dq. (2151)

29 or/20-28 (3412)

30 myocerebrohepatopath$.ti,ab,kw,dq. (23)

31 mchs.ti,ab,kw,dq. (125)

32 or/30-31 (140)

33 spinocerebellar degeneration/ and exp epilepsy/ (464)

34 (myoclon$ and epilep$ and sensory ataxia$).ti,ab,kw,dq. (23)

35 memsa.ti,ab,kw,dq. (19)

36 (spinocerebellar ataxia$ and epilep$).ti,ab,kw,dq. (262)

37 scae.ti,ab,kw,dq. (86)

38 or/33-37 (696)

39 chronic progressive external ophthalmoplegia/ (1017)

40 external ophthalmoplegi$.ti,ab,kw,dq. (2113)

41 ocular muscular dystroph$.ti,ab,kw,dq. (6)

42 (peo or cpeo$ or arpeo or adpeo).ti,ab,kw,dq. (5632)

43 ((graefe$ or graefe fuch$) adj6 (disease$ or disorder$ or syndrome$)).ti,ab,kw,dq. (11)

44 ((graefe$ or graefe fuch$) adj6 myopath$).ti,ab,kw,dq. (3)

45 or/39-44 (7419)

46 ataxia neuropath$.ti,ab,kw,dq. (216)

47 ataxic neuropath$.ti,ab,kw,dq. (399)

48 miras.ti,ab,kw,dq. (127)

49 sando.ti,ab,kw,dq. (119)

50 or/46-49 (786)

51 MELAS syndrome/ (2958)

52 melas.ti,ab,kw,dq. (2933)

53 myoencephalopath$.ti,ab,kw,dq. (12)

54 or/51-53 (3979)

55 MERRF syndrome/ (723)

56 merrf.ti,ab,kw,dq. (707)

57 merff.ti,ab,kw,dq. (35)

58 fukuhara$.ti,ab,kw,dq. (64)

59 (epilep$ and ragged red).ti,ab,kw,dq. (624)

60 or/55-59 (1282)

61 arginine transfer RNA ligase/ (224)

62 (mitochondri$ and arg$ and (tRNA or RNA or ribonucleic acid)).ti,ab,kw,dq. (651)

63 (rars2 or rars-2).ti,ab,kw,dq. (130)

64 (rarstwo or rars-two or rarsii or rars-ii or rars11 or rars-11).ti,ab,kw,dq. (7)

65 or/61-64 (949)

66 cerebellum hypoplasia/ or olivopontocerebellar atrophy/ or (pontocerebellar adj6 hypoplasia$).ti,ab,kw,dq. (3265)

67 (pontocerebellum adj6 hypoplasia$).ti,ab,kw,dq. (5)

68 (pch6 or pch-6).ti,ab,kw,dq. (42)

69 (pchsix or pch-six or pchvi or pch-vi).ti,ab,kw,dq. (1)

70 or/66-69 (3281)

71 pyruvate dehydrogenase complex deficiency/ (467)

72 (pyruvate dehydrogenase adj6 deficien$).ti,ab,kw,dq. (691)

73 ((pdhc or pdh or pyruvate decarboxylase) adj6 deficien$).ti,ab,kw,dq. (351)

74 ((ataxia or ataxic) adj6 (lactic acidosis or pyruvate or decarboxylase)).ti,ab,kw,dq. (146)

75 or/71-74 (1104)

76 7 or 13 or 19 or 29 or 32 or 38 or 45 or 50 or 54 or 60 or 65 or 70 or 75 (92930)

77 quality adjusted life year/ (29540)

78 (quality adjusted or adjusted life year$).ti,ab,kw,dq. (28216)

79 (qaly$ or qald$ or qale$ or qtime$).ti,ab,kw,dq. (22787)

80 (illness state$1 or health state$1).ti,ab,kw,dq. (12557)

81 (hui or hui1 or hui2 or hui3).ti,ab,kw,dq. (2623)

82 (multiattribute$ or multi attribute$).ti,ab,kw,dq. (1303)

83 (utility adj3 (score$1 or valu$ or health$ or cost$ or measur$ or disease$ or mean or gain or gains or index$)).ti,ab,kw,dq. (27156)

84 utilities.ti,ab,kw,dq. (13018)

85 exp "european quality of life 5 dimensions questionnaire"/ (7365)

86 (eq-5d or eq5d or eq-5 or eq5 or euro qual or euroqual or euro qual5d or euroqual5d or euro qol or euroqol or euro qol5d or euroqol5d or euro quol or euroquol or euro quol5d or euroquol5d or eur qol or eurqol or eur qol5d or eur qol5d or eur?qul or eur?qul5d or euro$ quality of life or european qol).ti,ab,kw,dq. (25123)

87 (euro$ adj3 (5 d or 5d or 5 dimension$ or 5dimension$ or 5 domain$ or 5domain$)).ti,ab,kw,dq. (7508)

88 short form 36/ (32451)

89 (sf36$ or sf 36$ or sf thirtysix or sf thirty six).ti,ab,kw,dq. (40958)

90 (time trade off$1 or time tradeoff$1 or tto or timetradeoff$1).ti,ab,kw,dq. (3046)

91 quality of life/ and ((quality of life or qol) adj (score$1 or measure$1)).ti,ab,kw,dq. (28153)

92 quality of life/ and (pe or de).fs. (9225)

93 quality of life/ and (health adj3 status).ti,ab,kw,dq. (17875)

94 (quality of life or qol).ti,ab,kw,dq. and cost benefit analysis/ (5979)

95 ((qol or hrqol or quality of life).ti,kw,dq. or *quality of life/) and ((qol or hrqol$ or quality of life) adj2 (increas$ or decrease$ or improv$ or declin$ or reduc$ or high$ or low$ or effect or effects or worse or score or scores or change$1 or impact$1 or impacted or deteriorat$)).ab. (64886)

96 cost benefit analysis/ and (cost-effectiveness ratio$ and (perspective$ or life expectanc$)).ti,ab,kw,dq. (937)

97 *quality of life/ and (quality of life or qol).ti. (94443)

98 quality of life/ and ((quality of life or qol) adj3 (improv$ or chang$)).ti,ab,kw,dq. (84382)

99 quality of life/ and health-related quality of life.ti,ab,kw,dq. (65264)

100 economic model/ (2496)

101 or/77-100 (322753)

102 (utility loss$ or disutility$).ti,ab,kw,dq. (924)

103 (ahum or aqol$ or chu9d or chu-9d or eq-5dy or eq5dy or health status classification system$ or hscs-ps or hscsps or hui or hui1 or hui2 or hui3 or quality of well-being or quality of wellbeing or qwb or 15d or 15-d or 15 dimension$ or 16d or 16-d or 16 dimension$ or 17d or 17-d or 17 dimension$).ti,ab,kw,dq. (15410)

104 101 or 102 or 103 (334297)

105 "quality of life"/ (518163)

106 "quality of life assessment"/ (11246)

107 ((quality adj3 life) or qol).ti,ab,kw,dq. (524395)

108 (hql or hqol or hrqol or hrql or hr-ql).ti,ab,kw,dq. (37114)

109 (short form$ or shortform$).ti,ab,kw,dq. (51948)

110 short form 12/ or short form 20/ or short form 8/ (8110)

111 (sf6 or sf-6 or sf6d or sf-6d or sf-six or sfsix).ti,ab,kw,dq. (3980)

112 (sf8 or sf-8 or sf-eight or sfeight).ti,ab,kw,dq. (904)

113 (sf12 or sf-12 or sf-twelve or sftwelve).ti,ab,kw,dq. (9078)

114 (sf16 or sf-16 or sf-sixteen or sfsixteen).ti,ab,kw,dq. (59)

115 (sf20 or sf-20 or sf-twenty or sftwenty).ti,ab,kw,dq. (353)

116 or/105-115 (694123)

117 (attitudes to children with epilepsy$ or aatcwe$ or disabkids$ or eldqol$ or "epilepsy and children questionnaire$" or ecq$ or "epilepsy foundation$ of america concern$ index$" or efa or efaci or glasgow epilepsy outcome scale$ or geos$ or hague restrictions in childhood epilepsy scale$ or harces$ or cheqol$ or modified impact of epilepsy schedule$ or mioes or impact of childhood illness scale$ or ici$ or impact of Paediatric epilepsy scale$ or impact of paediatric epilepsy scale$ or ipes$ or impact of childhood neurologic disability$ or icnd$ or neuroqol$ or newcastle mitochondrial disease$ or nmds$ or newcastle adult mitochondrial disease$ or nmdas$ or nmdsa$ or newcastle Paediatric mitochondrial disease$ or newcastle paediatric mitochondrial disease$ or npmds or pedsql$ or peds-ql$ or pedsqlem$ or peds-qlem$ or Paediatric epilepsy side effects questionnaire$ or paediatric epilepsy side effects questionnaire$ or pesq$ or patient-reported outcomes measurement information system$ or promis or promis25 or promis37 or promis49 or qolce$ or gqolce$ or qolie$ or qolpes$).ti,ab,kw,dq. or "Paediatric quality of life inventory"/ (50604)

118 116 or 117 (733409)

119 76 and 104 (330)

120 76 and 118 (1153)

121 119 or 120 (1244)

122 (animal/ or animal experiment/ or animal model/ or animal tissue/ or nonhuman/) not exp human/ (6273181)

123 conference abstract.pt. (4151130)

124 121 not (122 or 123) (836)

125 limit 124 to english language (805)

1. **Source: Cochrane Database of Systematic Reviews (CDSR)**

Interface / URL: Cochrane Library / Wiley

Database coverage dates: Information not found. Issue searched: Issue 8 of 12, August 2021

Search date: 11/08/2021

Retrieved records: 8

Search strategy:

#1 MeSH descriptor: [Mitochondrial Diseases] this term only 52

#2 MeSH descriptor: [Mitochondrial Myopathies] this term only 31

#3 MeSH descriptor: [Mitochondrial Encephalomyopathies] this term only 10

#4 (mitochondri* NEAR/6 (disease* OR disorder* OR syndrome*)):ti,ab,kw 340

#5 (mitochondri* NEAR/6 (cytopath* OR deficien* OR dysfunction* OR encephalomyopath* OR encephalopath* OR myopath*)):ti,ab,kw 479

#6 mitochondriopath*:ti,ab,kw 8

#7 (mitochondri* NEAR/6 (epilep* OR seizure* OR convuls*)):ti,ab,kw 9

#8 #1 OR #2 OR #3 OR #4 OR #5 OR #6 OR #7 695

#9 MeSH descriptor: [Leigh Disease] this term only 4

#10 ((leigh* OR leigh-feigin* OR leigh-feigin-wolf*) NEAR/6 (disease* OR disorder* OR syndrome*)):ti,ab,kw 11

#11 ((leigh* OR leigh-feigin* OR leigh-feigin-wolf*) NEAR/6 (encephalopath* OR encephalomyelopath*)):ti,ab,kw 0

#12 ((subacute OR sub-acute) NEAR/3 (necrot* NEXT encephal* OR necrot* NEXT juvenile NEXT encephal* OR necrot* NEXT infantile NEXT encephal*)):ti,ab,kw 0

#13 (leigh* AND mitochondri*):ti,ab,kw 8

#14 #9 OR #10 OR #11 OR #12 OR #13 11

#15 MeSH descriptor: [DNA Polymerase gamma] this term only 1

#16 (polymerase* NEAR/3 gamma):ti,ab,kw 12

#17 (polg OR polg1):ti,ab,kw 3

#18 (polgone OR polgi):ti,ab,kw 0

#19 (mitochondri* NEAR/6 polymerase*):ti,ab,kw 19

#20 #15 or #16 or #17 or #18 or #19 22

#21 MeSH descriptor: [Diffuse Cerebral Sclerosis of Schilder] this term only 7

#22 ((alper* OR alper-huttenlocher* OR alpers-huttenlocher*) NEAR/6 (disease* OR disorder* OR syndrome*)):ti,ab,kw 3

#23 ((alper* OR alper-huttenlocher* OR alpers-huttenlocher*) NEAR/6 ("diffuse cerebral" OR "diffuse degeneration" OR poliodystroph*)):ti,ab,kw 0

#24 (schilder* NEAR/6 (disease* OR disorder* OR syndrome*)):ti,ab,kw 1

#25 (schilder* NEAR/6 ("diffuse cerebral" OR "diffuse degeneration" OR poliodystroph*)):ti,ab,kw 7

#26 (balo* NEAR/3 (concentric NEXT scleros*)):ti,ab,kw 0

#27 (progressive NEAR/3 poliodystroph*):ti,ab,kw 0

#28 (diffuse NEXT cerebral NEXT degeneration* OR diffuse NEXT cerebral NEXT scleros* OR "encephalitis periaxialis" OR myelinoclastic NEXT diffuse NEXT scleros* OR "poliodystrophia cerebri" OR progressive NEXT neuronal NEXT degeneration* OR sudanophilic NEXT cerebral NEXT scleros*):ti,ab,kw 10

#29 ahs:ti,ab,kw 104

#30 #21 OR #22 OR #23 OR #24 OR #25 OR #26 OR #27 OR #28 OR #29 118

#31 myocerebrohepatopath*:ti,ab,kw 0

#32 mchs:ti,ab,kw 2

#33 #31 OR #32 2

#34 MeSH descriptor: [Spinocerebellar Ataxias] this term only 51

#35 MeSH descriptor: [Epilepsy] explode all trees 2454

#36 #34 AND #35 0

#37 (myoclon* AND epilep* AND sensory NEXT ataxia*):ti,ab,kw 0

#38 memsa:ti,ab,kw 8

#39 (spinocerebellar NEXT ataxia* AND epilep*):ti,ab,kw 1

#40 scae:ti,ab,kw 5

#41 #36 OR #37 OR #38 OR #39 OR #40 14

#42 MeSH descriptor: [Ophthalmoplegia, Chronic Progressive External] this term only 6

#43 external NEXT ophthalmoplegi*:ti,ab,kw 16

#44 ocular NEXT muscular NEXT dystroph*:ti,ab,kw 0

#45 (peo OR cpeo* OR arpeo OR adpeo):ti,ab,kw 32

#46 ((graefe* OR graefe NEXT fuch*) NEAR/6 (disease* OR disorder* OR syndrome*)):ti,ab,kw 1

#47 ((graefe* OR graefe NEXT fuch*) NEAR/6 myopath*):ti,ab,kw 0

#48 #42 OR #43 OR #44 OR #45 OR #46 OR #47 45

#49 ataxia NEXT neuropath*:ti,ab,kw 2

#50 ataxic NEXT neuropath*:ti,ab,kw 8

#51 miras:ti,ab,kw 11

#52 sando:ti,ab,kw 1

#53 #49 OR #50 OR #51 OR #52 22

#54 MeSH descriptor: [MELAS Syndrome] this term only 12

#55 melas:ti,ab,kw 57

#56 myoencephalopath*:ti,ab,kw 0

#57 #54 OR #55 OR #56 57

#58 MeSH descriptor: [MERRF Syndrome] this term only 0

#59 merrf:ti,ab,kw 2

#60 merff:ti,ab,kw 0

#61 fukuhara*:ti,ab,kw 1

#62 (epilep* AND "ragged red"):ti,ab,kw 1

#63 #58 OR #59 OR #60 OR #61 OR #62 4

#64 MeSH descriptor: [Arginine-tRNA Ligase] this term only 0

#65 (mitochondri* AND arg* AND (tRNA OR RNA OR "ribonucleic acid")):ti,ab,kw 3

#66 (rars2 OR rars-2):ti,ab,kw 5

#67 (rarstwo OR rars-two OR rarsii OR rars-ii OR rars11 OR rars-11):ti,ab,kw 0

#68 #64 OR #65 OR #66 OR #67 8

#69 MeSH descriptor: [Olivopontocerebellar Atrophies] this term only 10

#70 (pontocerebellar NEAR/6 hypoplasia*):ti,ab,kw 0

#71 (pontocerebellum NEAR/6 hypoplasia*):ti,ab,kw 0

#72 (pch6 OR pch-6):ti,ab,kw 0

#73 (pchsix OR pch-six OR pchvi OR pch-vi):ti,ab,kw 0

#74 #69 OR #70 OR #71 OR #72 OR #73 10

#75 MeSH descriptor: [Pyruvate Dehydrogenase Complex Deficiency Disease] this term only 4

#76 ("pyruvate dehydrogenase" NEAR/6 deficien*):ti,ab,kw 9

#77 ((pdhc OR pdh OR "pyruvate decarboxylase") NEAR/6 deficien*):ti,ab,kw 3

#78 ((ataxia OR ataxic) NEAR/6 ("lactic acidosis" OR pyruvate OR decarboxylase)):ti,ab,kw 1

#79 #75 OR #76 OR #77 OR #78 13

#80 #8 OR #14 OR #20 OR #30 OR #33 OR #41 OR #48 OR #53 OR #57 OR #63 OR #68 OR #74 OR #79 958

#81 #80 in Cochrane Reviews, Cochrane Protocols 8

1. **Source: Cochrane Central Register of Controlled Trials (CENTRAL)**

Interface / URL: Cochrane / Wiley

Database coverage dates: Information not found. Issue searched: Issue 8 of 12, August 2021

Search date: 12/08/2021

Retrieved records: 189

Search strategy:

ID Search Hits

#1 MeSH descriptor: [Mitochondrial Diseases] this term only 52

#2 MeSH descriptor: [Mitochondrial Myopathies] this term only 31

#3 MeSH descriptor: [Mitochondrial Encephalomyopathies] this term only 10

#4 mitochondri* NEAR/6 (disease* OR disorder* OR syndrome*) 412

#5 mitochondri* NEAR/6 (cytopath* OR deficien* OR dysfunction* OR encephalomyopath* OR encephalopath* OR myopath*) 522

#6 mitochondriopath* 9

#7 mitochondri* NEAR/6 (epilep* OR seizure* OR convuls*) 9

#8 #1 OR #2 OR #3 OR #4 OR #5 OR #6 OR #7 787

#9 MeSH descriptor: [Leigh Disease] this term only 4

#10 (leigh* OR leigh-feigin* OR leigh-feigin-wolf*) NEAR/6 (disease* OR disorder* OR syndrome*) 19

#11 (leigh* OR leigh-feigin* OR leigh-feigin-wolf*) NEAR/6 (encephalopath* OR encephalomyelopath*) 1

#12 (subacute OR sub-acute) NEAR/3 (necrot* NEXT encephal* OR necrot* NEXT juvenile NEXT encephal* OR necrot* NEXT infantile NEXT encephal*) 0

#13 leigh* AND mitochondri* 17

#14 #9 OR #10 OR #11 OR #12 OR #13 22

#15 MeSH descriptor: [DNA Polymerase gamma] this term only 1

#16 polymerase* NEAR/3 gamma 25

#17 polg OR polg1 4

#18 polgone OR polgi 0

#19 mitochondri* NEAR/6 polymerase* 27

#20 #15 or #16 or #17 or #18 or #19 41

#21 MeSH descriptor: [Diffuse Cerebral Sclerosis of Schilder] this term only 7

#22 (alper* OR alper-huttenlocher* OR alpers-huttenlocher*) NEAR/6 (disease* OR disorder* OR syndrome*) 4

#23 (alper* OR alper-huttenlocher* OR alpers-huttenlocher*) NEAR/6 ("diffuse cerebral" OR "diffuse degeneration" OR poliodystroph*) 0

#24 schilder* NEAR/6 (disease* OR disorder* OR syndrome*) 8

#25 schilder* NEAR/6 ("diffuse cerebral" OR "diffuse degeneration" OR poliodystroph*) 7

#26 balo* NEAR/3 (concentric NEXT scleros*) 0

#27 progressive NEAR/3 poliodystroph* 0

#28 diffuse NEXT cerebral NEXT degeneration* OR diffuse NEXT cerebral NEXT scleros* OR "encephalitis periaxialis" OR myelinoclastic NEXT diffuse NEXT scleros* OR "poliodystrophia cerebri" OR progressive NEXT neuronal NEXT degeneration* OR sudanophilic NEXT cerebral NEXT scleros* 10

#29 ahs 324

#30 #21 OR #22 OR #23 OR #24 OR #25 OR #26 OR #27 OR #28 OR #29 346

#31 myocerebrohepatopath* 0

#32 mchs 2

#33 #31 OR #32 2

#34 MeSH descriptor: [Spinocerebellar Ataxias] this term only 51

#35 MeSH descriptor: [Epilepsy] explode all trees 2454

#36 #34 AND #35 0

#37 myoclon* AND epilep* AND sensory NEXT ataxia* 0

#38 memsa 29

#39 spinocerebellar NEXT ataxia* AND epilep* 3

#40 scae 5

#41 #36 OR #37 OR #38 OR #39 OR #40 37

#42 MeSH descriptor: [Ophthalmoplegia, Chronic Progressive External] this term only 6

#43 external NEXT ophthalmoplegi* 18

#44 ocular NEXT muscular NEXT dystroph* 0

#45 peo OR cpeo* OR arpeo OR adpeo 46

#46 (graefe* OR graefe NEXT fuch*) NEAR/6 (disease* OR disorder* OR syndrome*) 1

#47 (graefe* OR graefe NEXT fuch*) NEAR/6 myopath* 0

#48 #42 OR #43 OR #44 OR #45 OR #46 OR #47 60

#49 ataxia NEXT neuropath* 3

#50 ataxic NEXT neuropath* 10

#51 miras 87

#52 sando 82

#53 #49 OR #50 OR #51 OR #52 182

#54 MeSH descriptor: [MELAS Syndrome] this term only 12

#55 melas 94

#56 myoencephalopath* 0

#57 #54 OR #55 OR #56 94

#58 MeSH descriptor: [MERRF Syndrome] this term only 0

#59 merrf 4

#60 merff 1

#61 fukuhara* 204

#62 epilep* AND "ragged red" 2

#63 #58 OR #59 OR #60 OR #61 OR #62 209

#64 MeSH descriptor: [Arginine-tRNA Ligase] this term only 0

#65 mitochondri* AND arg* AND (tRNA OR RNA OR "ribonucleic acid") 9

#66 rars2 OR rars-2 5

#67 rarstwo OR rars-two OR rarsii OR rars-ii OR rars11 OR rars-11 0

#68 #64 OR #65 OR #66 OR #67 14

#69 MeSH descriptor: [Olivopontocerebellar Atrophies] this term only 10

#70 pontocerebellar NEAR/6 hypoplasia* 0

#71 pontocerebellum NEAR/6 hypoplasia* 0

#72 pch6 OR pch-6 0

#73 pchsix OR pch-six OR pchvi OR pch-vi 0

#74 #69 OR #70 OR #71 OR #72 OR #73 10

#75 MeSH descriptor: [Pyruvate Dehydrogenase Complex Deficiency Disease] this term only 4

#76 "pyruvate dehydrogenase" NEAR/6 deficien* 12

#77 (pdhc OR pdh OR "pyruvate decarboxylase") NEAR/6 deficien* 3

#78 (ataxia OR ataxic) NEAR/6 ("lactic acidosis" OR pyruvate OR decarboxylase) 1

#79 #75 OR #76 OR #77 OR #78 16

#80 #8 OR #14 OR #20 OR #30 OR #33 OR #41 OR #48 OR #53 OR #57 OR #63 OR #68 OR #74 OR #79 1737

#81 MeSH descriptor: [Quality-Adjusted Life Years] this term only 1339

#82 "quality adjusted" OR adjusted NEXT life NEXT year* 6195

#83 qaly* OR qald* OR qale* OR qtime* 4179

#84 illness NEXT state* OR health NEXT state* 1384

#85 hui OR hui1 OR hui2 OR hui3 2056

#86 multiattribute* OR multi NEXT attribute* 87

#87 utility NEAR/3 (score* OR valu* OR health* OR cost* OR measur* OR disease* OR mean OR gain OR gains OR index*) 4461

#88 utilities 1232

#89 eq-5d OR eq5d OR eq-5 OR eq5 OR "euro qual" OR euroqual OR "euro qual5d" OR euroqual5d OR "euro qol" OR euroqol OR "euro qol5d" OR euroqol5d OR "euro quol" OR euroquol OR "euro quol5d" OR euroquol5d OR "eur qol" OR eurqol OR "eur qol5d" OR "eur qol5d" OR eur?qul OR eur?qul5d OR euro* NEXT "quality of life" OR "european qol" 11428

#90 euro* NEAR/3 ("5 d" OR 5d OR 5 NEXT dimension* OR 5dimension* OR 5 NEXT domain* OR 5domain*) 3407

#91 sf36* OR sf-36* OR "sf thirtysix" OR "sf thirty six" 13962

#92 time NEXT trade NEXT off* OR time NEXT tradeoff* OR tto OR timetradeoff* 301

#93 MeSH descriptor: [Cost-Benefit Analysis] this term only 7285

#94 cost-effectiveness NEXT ratio* AND (perspective* OR life NEXT expectanc*) 1723

#95 #93 AND #94 594

#96 MeSH descriptor: [Models, Economic] this term only 251

#97 #81 OR #82 OR #83 OR #84 OR #85 OR #86 OR #87 OR #88 OR #89 OR #90 OR #91 OR #92 OR #95 OR #96 33062

#98 utility NEXT loss* OR disutility* 62

#99 ahum OR aqol* OR chu9d OR chu-9d OR eq-5dy OR eq5dy OR "health status classification" NEXT system* OR hscs-ps OR hscsps OR hui OR hui1 OR hui2 OR hui3 OR "quality of well-being" OR "quality of wellbeing" OR qwb OR 15d OR "15 d" OR 15 NEXT dimension* OR 16d OR "16 d" OR 16 NEXT dimension* OR 17d OR "17 d" OR 17 NEXT dimension* 8990

#100 #97 OR #98 OR #99 38575

#101 MeSH descriptor: [Quality of Life] this term only 25960

#102 (quality NEAR/3 life) OR qol 131638

#103 hql OR hqol OR hrqol OR hrql OR hr-ql 7547

#104 short NEXT form* OR shortform* 16463

#105 sf6 OR sf-6 OR sf6d OR sf-6d OR sf-six OR sfsix 531

#106 sf8 OR sf-8 OR sf-eight OR sfeight 257

#107 sf12 OR sf-12 OR sf-twelve OR sftwelve 2735

#108 sf16 OR sf-16 OR sf-sixteen OR sfsixteen 11

#109 sf20 OR sf-20 OR sf-twenty OR sftwenty 87

#110 #101 OR #102 OR #103 OR #104 OR #105 OR #106 OR #107 OR #108 OR #109 138303

#111 "attitudes to children with" NEXT epilepsy* OR aatcwe* OR disabkids* OR eldqol* OR (epilepsy AND (children NEXT questionnaire*)) OR ecq* OR epilepsy NEXT foundation* NEXT of NEXT america NEXT concern* NEXT index* OR efa OR efaci OR "glasgow epilepsy outcome" NEXT scale* OR geos* OR "hague restrictions in childhood epilepsy" NEXT scale* OR harces* OR cheqol* OR "modified impact of epilepsy" NEXT schedule* OR mioes OR "impact of childhood illness" NEXT scale* OR ici* OR "impact of Paediatric epilepsy" NEXT scale* OR "impact of paediatric epilepsy" NEXT scale* OR ipes* OR "impact of childhood neurologic" NEXT disability* OR icnd* OR neuroqol* OR newcastle NEXT mitochondrial NEXT disease* OR nmds* OR "newcastle adult mitochondrial" NEXT disease* OR nmdas* OR nmdsa* OR "newcastle Paediatric mitochondrial" NEXT disease* OR "newcastle paediatric mitochondrial" NEXT disease* OR npmds OR pedsql* OR peds-ql* OR pedsqlem* OR peds-qlem* OR "Paediatric epilepsy side effects" NEXT questionnaire* OR "paediatric epilepsy side effects" NEXT questionnaire* OR pesq* OR "patient-reported outcomes measurement information" NEXT system* OR promis OR promis25 OR promis37 OR promis49 OR qolce* OR gqolce* OR qolie* OR qolpes* 6165

#112 #110 OR #111 141604

#113 #80 AND #100 209

#114 #80 AND #112 355

#115 #113 OR #114 405

#116 #115 in Trials 189

1. **Source: HTA Database**

Interface / URL: https://database.inahta.org/

Database coverage dates: Information not found. The former database was produced by the CRD until March 2018, at which time the addition of records was stopped as INAHTA was in the process of rebuilding the new database platform. In July 2019, the database records were exported from the CRD platform and imported into the new platform that was developed by INAHTA. The rebuild of the new platform was launched in June 2020.

Search date: 12/08/2021

Retrieved records: 23

Search strategy:

54 #53 OR #52 OR #51 OR #50 OR #49 OR #48 OR #47 OR #46 OR #45 OR #44 OR #43 OR #42 OR #41 OR #40 OR #39 OR #38 OR #37 OR #36 OR #35 OR #34 OR #33 OR #32 OR #31 OR #30 OR #29 OR #28 OR #27 OR #26 OR #25 OR #24 OR #23 OR #22 OR #21 OR #20 OR #19 OR #18 OR #17 OR #16 OR #15 OR #14 OR #13 OR #12 OR #11 OR #10 OR #9 OR #8 OR #7 OR #6 OR #5 OR #4 OR #3 OR #2 OR #1 23

53 (ataxia OR ataxic) AND ("lactic acidosis" OR pyruvate OR decarboxylase) 0

52 (pdhc OR pdh OR "pyruvate decarboxylase") AND deficien* 0

51 ("pyruvate dehydrogenase" AND deficien*) 0

50 "Pyruvate Dehydrogenase Complex Deficiency Disease"[mh] 0

49 (pchsix OR "pch-six" OR pchvi OR "pch-vi") 0

48 (pch6 OR "pch-6") 0

47 (pontocerebellum AND hypoplasia*) 0

46 (pontocerebellar AND hypoplasia*) 0

45 "Olivopontocerebellar Atrophies"[mh] 0

44 (rarstwo OR "rars-two" OR rarsii OR "rars-ii" OR rars11 OR "rars-11") 0

43 (rars2 OR "rars-2") 0

42 "Arginine-tRNA Ligase"[mh] 0

41 epilep* AND "ragged red" 0

40 fukuhara* 0

39 merff 0

38 merrf 0

37 "MERRF Syndrome"[mh] 0

36 myoencephalopath* 0

35 melas 0

34 "MELAS Syndrome"[mh] 0

33 sando 0

32 miras 0

31 ataxic AND neuropath* 0

30 ataxia AND neuropath* 4

29 graefe* 0

28 (peo OR cpeo* OR arpeo OR adpeo) 1

27 "ocular muscular" 0

26 ophthalmoplegi* 2

25 "Ophthalmoplegia, Chronic Progressive External"[mh] 0

24 scae 0

23 spinocerebellar AND ataxia* AND epilep* 1

22 memsa 1

21 myoclon* AND epilep* AND ataxia* 3

20 "Spinocerebellar Ataxias"[mh] AND "Epilepsy"[mhe] 0

19 mchs 1

18 myocerebrohepatopath* 1

17 ahs 1

16 ("diffuse cerebral" OR "encephalitis periaxialis" OR "myelinoclastic diffuse" OR "poliodystrophia cerebri" OR "progressive neuronal" OR "sudanophilic cerebral") 0

15 poliodystroph* 0

14 balo* AND concentric 0

13 schilder* 3

12 alper* 1

11 "Diffuse Cerebral Sclerosis of Schilder"[mh] 0

10 (polgone OR polgi) 0

9 (polg OR polg1) 1

8 (polymerase* AND gamma) 1

7 "DNA Polymerase gamma"[mh] 0

6 (subacute OR "sub-acute") AND encephal* 0

5 leigh* 3

4 "Leigh Disease"[mh] 0

3 mitochondri* 11

2 "Mitochondrial Myopathies"[mh] OR "Mitochondrial Encephalomyopathies"[mh] 1

1 "Mitochondrial Diseases"[mh] 1

1. **Source: Cost-Effectiveness Analysis Registry**

Interface / URL: http://healtheconomics.tuftsmedicalcenter.org/cear4/home.aspx

Database coverage dates: The webpage at the above URL states that the database includes studies published from 1976 to 2019. However, a check of the actual content indicates that records with 2020 and 2021 publication dates are available to be found in the database on the date of search.

Search date: 12/08/2021

Retrieved records: 0

Search strategy:

The basic search interface at the following URL was used: <http://healtheconomics.tuftsmedicalcenter.org/cear2n/search/search.aspx>. Search settings were left as default.

Separate searches were conducted on each of the following terms. Returned results were screened by the information specialist for potential relevance to the populations of interest. Potentially relevant results were cross-checked against results from other search sources to see if already retrieved. Any potentially relevant results not identified via another source were retrieved for further assessment.

mitochondri = 0 (1 result returned, excluded)

leigh = 0 (23 results returned, excluded)

subacute = 0 (15 results returned, excluded)

sub-acute = 0 (4 results returned, excluded)

polymerase = 0 (37 results returned, excluded)

polg = 0 (0 results returned)

alper = 0 (25 results returned, excluded)

schilder = 0 (1 result returned, excluded)

concentric = 0 (0 results returned)

poliodystroph = 0 (0 results returned)

diffuse cerebral = 0 (0 results returned)

encephalitis periaxialis = 0 (0 results returned)

myelinoclastic diffuse = 0 (0 results returned)

progressive neuronal = 0 (0 results returned)

sudanophilic cerebral = 0 (0 results returned)

ahs = 0 (9 results returned, excluded)

myocerebrohepatopath = 0 (0 results returned)

mchs = 0 (0 results returned)

sensory ataxia = 0 (0 results returned)

memsa = 0 (0 results returned)

spinocerebellar ataxia = 0 (0 results returned)

scae = 0 (0 results returned)

external ophthalmoplegi = 0 (0 results returned)

ocular muscular dystroph = 0 (0 results returned)

peo[space] = 0 (0 results returned) *

cpeo = 0 (0 results returned)

arpeo = 0 (0 results returned)

adpeo = 0 (0 results returned)

graefe = 0 (3 results returned, excluded)

ataxia neuropath = 0 (0 results returned)

ataxic neuropath = 0 (0 results returned)

miras = 0 (1 result returned, excluded)

sando = 0 (2 results returned, excluded)

melas = 0 (2 results returned, excluded)

myoencephalopath = 0 (0 results returned)

merrf = 0 (0 results returned)

merff = 0 (0 results returned)

fukuhara = 0 (4 results returned, excluded)

ragged red = 0 (0 results returned)

rars = 0 (0 results returned)

pontocerebellar = 0 (0 results returned)

pontocerebellum = 0 (0 results returned)

pch = 0 (4 results returned, excluded)

pdh = 0 (0 results returned)

pyruvate = 0 (0 results returned)

lactic acidosis = 0 (0 results returned)

decarboxylase = 0 (0 results returned)

Search notes:

* The search term *peo* when entered was followed by a space.

1. **Source: NHS Economic Evaluation Database (NHS EED)**

Interface / URL: https://www.crd.york.ac.uk/CRDWeb

Database coverage dates: Information not found. Bibliographic records were published on NHS EED until 31st March 2015. Searches of MEDLINE, Embase, CINAHL, PsycINFO and PubMed were continued until the end of the 2014.

Search date: 13/08/2021

Retrieved records: 27

Search strategy:

1 MeSH DESCRIPTOR mitochondrial diseases 3

2 MeSH DESCRIPTOR mitochondrial myopathies 0

3 MeSH DESCRIPTOR mitochondrial encephalomyopathies 0

4 (mitochondri*) 29

5 MeSH DESCRIPTOR Leigh Disease 0

6 ((leigh* AND (disease* OR disorder* OR syndrome* OR encephalopath* OR encephalomyelopath*))) 16

7 (((subacute OR sub-acute) AND encephal*)) 3

8 MeSH DESCRIPTOR DNA Polymerase gamma 0

9 ((polymerase* AND gamma)) 1

10 (polg*) 2

11 MeSH DESCRIPTOR Diffuse Cerebral Sclerosis of Schilder 0

12 ((alper* AND (disease* OR disorder* OR syndrome* ))) 14

13 (diffuse cerebral OR diffuse degeneration OR poliodystroph*) 0

14 ((schilder* AND (disease* OR disorder* OR syndrome* ))) 12

15 (concentric scleros*) 0

16 (encephalitis periaxialis OR myelinoclastic diffuse scleros* OR progressive neuronal degeneration* OR sudanophilic cerebral scleros*) 0

17 (ahs) 5

18 (myocerebrohepatopath*) 1

19 (mchs) 1

20 MeSH DESCRIPTOR Spinocerebellar Ataxias 9

21 ((myoclon* AND epilep* AND sensory ataxia*)) 1

22 (memsa) 1

23 ((spinocerebellar ataxia* AND epilep*)) 1

24 (scae) 0

25 MeSH DESCRIPTOR Ophthalmoplegia, Chronic Progressive External 0

26 (external ophthalmoplegi*) 1

27 (ocular muscular dystroph*) 0

28 ((peo OR cpeo* OR arpeo OR adpeo)) 3

29 ((graefe* AND (disease* OR disorder* OR syndrome* OR myopath*))) 3

30 ((ataxia neuropath* OR ataxic neuropath* OR miras OR sando)) 1

31 MeSH DESCRIPTOR MELAS Syndrome EXPLODE ALL TREES 0

32 ((melas OR myoencephalopath*)) 0

33 MeSH DESCRIPTOR MERRF Syndrome 0

34 ((merrf OR merff OR fukuhara* OR ragged red)) 12

35 MeSH DESCRIPTOR Arginine-tRNA Ligase 0

36 (rars*) 0

37 MeSH DESCRIPTOR Olivopontocerebellar Atrophies 0

38 ((pontocerebellar ADJ6 hypoplasia*)) OR ((hypoplasia* ADJ6 pontocerebellar)) 0

39 ((pontocerebellum ADJ6 hypoplasia*)) OR ((hypoplasia* ADJ6 pontocerebellum)) 0

40 (pch*) 8

41 MeSH DESCRIPTOR Pyruvate Dehydrogenase Complex Deficiency Disease 0

42 ((pyruvate dehydrogenase ADJ6 deficien*)) OR ((deficien* ADJ6 pyruvate dehydrogenase)) 0

43 (((pdhc OR pdh OR pyruvate decarboxylase) ADJ6 deficien*)) OR (deficien* ADJ6 (pdhc OR pdh OR pyruvate decarboxylase)) 0

44 (((ataxia OR ataxic) ADJ6 (lactic acidosis OR pyruvate OR decarboxylase))) OR ((lactic acidosis OR pyruvate OR decarboxylase) ADJ6 (ataxia OR ataxic)) 0

45 #1 OR #2 OR #3 OR #4 OR #5 OR #6 OR #7 OR #8 OR #9 OR #10 OR #11 OR #12 OR #13 OR #14 OR #15 OR #16 OR #17 OR #18 OR #19 OR #20 OR #21 OR #22 OR #23 OR #24 OR #25 OR #26 OR #27 OR #28 OR #29 OR #30 OR #31 OR #32 OR #33 OR #34 OR #35 OR #36 OR #37 OR #38 OR #39 OR #40 OR #41 OR #42 OR #43 OR #44 112

46 (#45) IN NHSEED 27

1. **Source: EconLit**

Interface / URL: OvidSP

Database coverage dates: 1886 to August 05, 2021

Search date: 12/08/2021

Retrieved records: 79

Search strategy:

1 (mitochondri$ adj6 (disease$ or disorder$ or syndrome$)).af. (0)

2 (mitochondri$ adj6 (cytopath$ or deficien$ or dysfunction$ or encephalomyopath$ or encephalopath$ or myopath$)).af. (0)

3 mitochondriopath$.af. (0)

4 (mitochondri$ adj6 (epilep$ or seizure$ or convuls$)).af. (0)

5 or/1-4 (0)

6 ((leigh$ or leigh-feigin$ or leigh-feigin-wolf$) adj6 (disease$ or disorder$ or syndrome$)).af. (0)

7 ((leigh$ or leigh-feigin$ or leigh-feigin-wolf$) adj6 (encephalopath$ or encephalomyelopath$)).af. (0)

8 ((subacute or sub-acute) adj3 (necrot$ encephal$ or necrot$ juvenile encephal$ or necrot$ infantile encephal$)).af. (0)

9 (leigh$ and mitochondri$).af. (0)

10 or/6-9 (0)

11 (polymerase$ adj3 gamma).af. (0)

12 (polg or polg1).af. (0)

13 (polgone or polgi).af. (0)

14 (mitochondri$ adj6 polymerase$).af. (0)

15 or/11-14 (0)

16 ((alper$ or alper-huttenlocher$ or alpers-huttenlocher$) adj6 (disease$ or disorder$ or syndrome$)).af. (1)

17 ((alper$ or alper-huttenlocher$ or alpers-huttenlocher$) adj6 (diffuse cerebral or diffuse degeneration or poliodystroph$)).af. (0)

18 (schilder$ adj6 (disease$ or disorder$ or syndrome$)).af. (0)

19 (schilder$ adj6 (diffuse cerebral or diffuse degeneration or poliodystroph$)).af. (0)

20 (balo$ adj3 concentric scleros$).af. (0)

21 (progressive adj3 poliodystroph$).af. (0)

22 (diffuse cerebral degeneration$ or diffuse cerebral scleros$ or encephalitis periaxialis or myelinoclastic diffuse scleros$ or poliodystrophia cerebri or progressive neuronal degeneration$ or sudanophilic cerebral scleros$).af. (0)

23 ahs.af. (50)

24 or/16-23 (51)

25 myocerebrohepatopath$.af. (0)

26 mchs.af. (0)

27 25 or 26 (0)

28 (myoclon$ and epilep$ and sensory ataxia$).af. (0)

29 memsa.af. (0)

30 (spinocerebellar ataxia$ and epilep$).af. (0)

31 scae.af. (0)

32 or/28-31 (0)

33 external ophthalmoplegi$.af. (0)

34 ocular muscular dystroph$.af. (0)

35 (peo or cpeo$ or arpeo or adpeo).af. (6)

36 ((graefe$ or graefe fuch$) adj6 (disease$ or disorder$ or syndrome$)).af. (0)

37 ((graefe$ or graefe fuch$) adj6 myopath$).af. (0)

38 or/33-37 (6)

39 ataxia neuropath$.af. (0)

40 ataxic neuropath$.af. (0)

41 miras.af. (10)

42 sando.af. (0)

43 or/39-42 (10)

44 melas.af. (2)

45 myoencephalopath$.af. (0)

46 44 or 45 (2)

47 merrf.af. (0)

48 merff.af. (0)

49 fukuhara$.af. (10)

50 (epilep$ and ragged red).af. (0)

51 or/47-50 (10)

52 (mitochondri$ and arg$ and (tRNA or RNA or ribonucleic acid)).af. (0)

53 (rars2 or rars-2).af. (0)

54 (rarstwo or rars-two or rarsii or rars-ii or rars11 or rars-11).af. (0)

55 or/52-54 (0)

56 (pontocerebellar adj6 hypoplasia$).af. (0)

57 (pontocerebellum adj6 hypoplasia$).af. (0)

58 (pch6 or pch-6).af. (0)

59 (pchsix or pch-six or pchvi or pch-vi).af. (0)

60 or/56-59 (0)

61 (pyruvate dehydrogenase adj6 deficien$).af. (0)

62 ((pdhc or pdh or pyruvate decarboxylase) adj6 deficien$).af. (0)

63 ((ataxia or ataxic) adj6 (lactic acidosis or pyruvate or decarboxylase)).af. (0)

64 or/61-63 (0)

65 5 or 10 or 15 or 24 or 27 or 32 or 38 or 43 or 46 or 51 or 55 or 60 or 64 (79)

1. **Source: Paediatric Economic Database Evaluation**

Interface / URL: http://pede.ccb.sickkids.ca/pede/

Database coverage dates: The information at the following URL states that the database contains records for studies published from January 1, 1980 to December 31, 2019 - http://pede.ccb.sickkids.ca/pede/database.jsp.

Search date: 13/08/2021

Retrieved records: 0

Search strategy:

The basic search interface at the following URL was used: <http://pede.ccb.sickkids.ca/pede/search.jsp>. All search settings were left as default. In the default setting, terms are searched across the 'Title, abstract, or Keywords'.

The basic search interface allows the searcher to enter up to three terms combined with AND using the separate search line functionality built into the interface. Where AND is shown between terms in the syntax below, this indicates that this functionality was used. All other terms were searched for individually and not combined with any other term.

Separate searches were conducted on each of the following terms. Returned results were screened by the information specialist for potential relevance to the populations of interest. Potentially relevant results were cross-checked against results from other search sources to see if already retrieved. Any potentially relevant results not identified via another source were retrieved for further assessment.

mitochondri = 0 (0 results returned)

leigh = 0 (0 results returned)

subacute = 0 (0 results returned)

sub-acute = 0 (0 results returned)

polymerase AND gamma = 0 (0 results returned)

polg = 0 (0 results returned)

alper = 0 (0 results returned)

schilder = 0 (0 results returned)

concentric = 0 (0 results returned)

poliodystroph = 0 (0 results returned)

diffuse cerebral = 0 (0 results returned)

encephalitis periaxialis = 0 (0 results returned)

myelinoclastic diffuse = 0 (0 results returned)

progressive neuronal = 0 (0 results returned)

sudanophilic cerebral = 0 (0 results returned)

ahs = 0 (2 results returned, excluded)

myocerebrohepatopath = 0 (0 results returned)

mchs = 0 (0 results returned)

sensory ataxia = 0 (0 results returned)

memsa = 0 (0 results returned)

spinocerebellar ataxia = 0 (0 results returned)

scae = 0 (0 results returned)

external ophthalmoplegi = 0 (0 results returned)

ocular muscular dystroph = 0 (0 results returned)

peo = 0 (91 results returned, excluded)

cpeo = 0 (0 results returned)

arpeo = 0 (0 results returned)

adpeo = 0 (0 results returned)

graefe = 0 (0 results returned)

ataxia neuropath = 0 (0 results returned)

ataxic neuropath = 0 (0 results returned)

miras = 0 (0 result returned)

sando = 0 (0 results returned)

melas = 0 (0 results returned)

myoencephalopath = 0 (0 results returned)

merrf = 0 (0 results returned)

merff = 0 (0 results returned)

fukuhara = 0 (0 results returned)

ragged red = 0 (0 results returned)

rars = 0 (0 results returned)

pontocerebellar = 0 (0 results returned)

pontocerebellum = 0 (0 results returned)

pch = 0 (1 result returned, excluded)

pdh = 0 (0 results returned)

pyruvate = 0 (0 results returned)

lactic acidosis = 0 (0 results returned)

decarboxylase = 0 (0 results returned)

1. **Source: APA PsycInfo**

Interface / URL: OvidSP

Database coverage dates: 1806 to August Week 2 2021

Search date: 12/08/2021

Retrieved records: 90

Search strategy:

1 mitochondria/ (4047)

2 (mitochondri$ adj6 (disease$ or disorder$ or syndrome$)).ti,ab,hw,id. (1958)

3 (mitochondri$ adj6 (cytopath$ or deficien$ or dysfunction$ or encephalomyopath$ or encephalopath$ or myopath$)).ti,ab,hw,id. (2785)

4 mitochondriopath$.ti,ab,hw,id. (31)

5 (mitochondri$ adj6 (epilep$ or seizure$ or convuls$)).ti,ab,hw,id. (129)

6 or/1-5 (5574)

7 ((leigh$ or leigh-feigin$ or leigh-feigin-wolf$) adj6 (disease$ or disorder$ or syndrome$)).ti,ab,hw,id. (161)

8 ((leigh$ or leigh-feigin$ or leigh-feigin-wolf$) adj6 (encephalopath$ or encephalomyelopath$)).ti,ab,hw,id. (17)

9 ((subacute or sub-acute) adj3 (necrot$ encephal$ or necrot$ juvenile encephal$ or necrot$ infantile encephal$)).ti,ab,hw,id. (8)

10 (leigh$ and mitochondri$).ti,ab,hw,id. (135)

11 or/7-10 (169)

12 (polymerase$ adj3 gamma).ti,ab,hw,id. (70)

13 (polg or polg1).ti,ab,hw,id. (138)

14 (polgone or polgi).ti,ab,hw,id. (1)

15 (mitochondri$ adj6 polymerase$).ti,ab,hw,id. (65)

16 or/12-15 (176)

17 ((alper$ or alper-huttenlocher$ or alpers-huttenlocher$) adj6 (disease$ or disorder$ or syndrome$)).ti,ab,hw,id. (27)

18 ((alper$ or alper-huttenlocher$ or alpers-huttenlocher$) adj6 (diffuse cerebral or diffuse degeneration or poliodystroph$)).ti,ab,hw,id. (1)

19 (schilder$ adj6 (disease$ or disorder$ or syndrome$)).ti,ab,hw,id. (16)

20 (schilder$ adj6 (diffuse cerebral or diffuse degeneration or poliodystroph$)).ti,ab,hw,id. (0)

21 (balo$ adj3 concentric scleros$).ti,ab,hw,id. (22)

22 (progressive adj3 poliodystroph$).ti,ab,hw,id. (1)

23 (diffuse cerebral degeneration$ or diffuse cerebral scleros$ or encephalitis periaxialis or myelinoclastic diffuse scleros$ or poliodystrophia cerebri or progressive neuronal degeneration$ or sudanophilic cerebral scleros$).ti,ab,hw,id. (33)

24 ahs.ti,ab,hw,id. (292)

25 or/17-24 (384)

26 myocerebrohepatopath$.ti,ab,hw,id. (1)

27 mchs.ti,ab,hw,id. (14)

28 26 or 27 (14)

29 (myoclon$ and epilep$ and sensory ataxia$).ti,ab,hw,id. (1)

30 memsa.ti,ab,hw,id. (4)

31 (spinocerebellar ataxia$ and epilep$).ti,ab,hw,id. (33)

32 scae.ti,ab,hw,id. (5)

33 or/29-32 (38)

34 external ophthalmoplegi$.ti,ab,hw,id. (128)

35 ocular muscular dystroph$.ti,ab,hw,id. (0)

36 (peo or cpeo$ or arpeo or adpeo).ti,ab,hw,id. (116)

37 ((graefe$ or graefe fuch$) adj6 (disease$ or disorder$ or syndrome$)).ti,ab,hw,id. (0)

38 ((graefe$ or graefe fuch$) adj6 myopath$).ti,ab,hw,id. (1)

39 or/34-38 (194)

40 ataxia neuropath$.ti,ab,hw,id. (28)

41 ataxic neuropath$.ti,ab,hw,id. (35)

42 miras.ti,ab,hw,id. (52)

43 sando.ti,ab,hw,id. (15)

44 or/40-43 (119)

45 melas/ (142)

46 melas.ti,ab,hw,id. (250)

47 myoencephalopath$.ti,ab,hw,id. (1)

48 or/45-47 (251)

49 merrf.ti,ab,hw,id. (46)

50 merff.ti,ab,hw,id. (2)

51 fukuhara$.ti,ab,hw,id. (7)

52 (epilep$ and ragged red).ti,ab,hw,id. (37)

53 or/49-52 (66)

54 (mitochondri$ and arg$ and (tRNA or RNA or ribonucleic acid)).ti,ab,hw,id. (8)

55 (rars2 or rars-2).ti,ab,hw,id. (7)

56 (rarstwo or rars-two or rarsii or rars-ii or rars11 or rars-11).ti,ab,hw,id. (1)

57 or/54-56 (13)

58 ((pontocerebellar adj6 hypoplasia$) or (pontocerebellum adj6 hypoplasia$)).ti,ab,hw,id. (48)

59 (pch6 or pch-6).ti,ab,hw,id. (3)

60 (pchsix or pch-six or pchvi or pch-vi).ti,ab,hw,id. (0)

61 or/58-60 (49)

62 (pyruvate dehydrogenase adj6 deficien$).ti,ab,hw,id. (24)

63 ((pdhc or pdh or pyruvate decarboxylase) adj6 deficien$).ti,ab,hw,id. (8)

64 ((ataxia or ataxic) adj6 (lactic acidosis or pyruvate or decarboxylase)).ti,ab,hw,id. (16)

65 or/62-64 (41)

66 6 or 11 or 16 or 25 or 28 or 33 or 39 or 44 or 48 or 53 or 57 or 61 or 65 (6383)

67 exp "Quality of Life"/ (46078)

68 (quality adjusted or adjusted life year$).ti,ab,hw,id. (2031)

69 (qaly$ or qald$ or qale$ or qtime$).ti,ab,hw,id. (1306)

70 (illness state$1 or health state$1).ti,ab,hw,id. (1747)

71 (hui or hui1 or hui2 or hui3).ti,ab,hw,id. (617)

72 (multiattribute$ or multi attribute$).ti,ab,hw,id. (1194)

73 (utility adj3 (score$1 or valu$ or health$ or cost$ or measur$ or disease$ or mean or gain or gains or index$)).ti,ab,hw,id. (4901)

74 utilities.ti,ab,hw,id. (2181)

75 (eq-5d or eq5d or eq-5 or eq5 or euro qual or euroqual or euro qual5d or euroqual5d or euro qol or euroqol or euro qol5d or euroqol5d or euro quol or euroquol or euro quol5d or euroquol5d or eur qol or eurqol or eur qol5d or eur qol5d or eur?qul or eur?qul5d or euro$ quality of life or european qol).ti,ab,hw,id. (2631)

76 (euro$ adj3 (5 d or 5d or 5 dimension$ or 5dimension$ or 5 domain$ or 5domain$)).ti,ab,hw,id. (754)

77 (sf36$ or sf 36$ or sf thirtysix or sf thirty six).ti,ab,hw,id. (4929)

78 (time trade off$1 or time tradeoff$1 or tto or timetradeoff$1).ti,ab,hw,id. (453)

79 ("Costs and Cost Analysis"/ or exp Health Care Costs/) and (cost-effectiveness ratio$ and (perspective$ or life expectanc$)).ti,ab,hw,id. (289)

80 (economics/ or health care economics/ or pharmacoeconomics/ or "costs and cost analysis"/) and models/ (1067)

81 or/67-80 (58897)

82 (utility loss$ or disutility$).ti,ab,hw,id. (255)

83 (ahum or aqol$ or chu9d or chu-9d or eq-5dy or eq5dy or health status classification system$ or hscs-ps or hscsps or hui or hui1 or hui2 or hui3 or quality of well-being or quality of wellbeing or qwb or 15d or 15-d or 15 dimension$ or 16d or 16-d or 16 dimension$ or 17d or 17-d or 17 dimension$).ti,ab,hw,id. (1467)

84 or/81-83 (59571)

85 ((quality adj3 life) or qol).ti,ab,hw,id. (82199)

86 (hql or hqol or hrqol or hrql or hr-ql).ti,ab,hw,id. (6322)

87 (short form$ or shortform$).ti,ab,hw,id. (14788)

88 (sf6 or sf-6 or sf6d or sf-6d or sf-six or sfsix).ti,ab,hw,id. (341)

89 (sf8 or sf-8 or sf-eight or sfeight).ti,ab,hw,id. (134)

90 (sf12 or sf-12 or sf-twelve or sftwelve).ti,ab,hw,id. (1324)

91 (sf16 or sf-16 or sf-sixteen or sfsixteen).ti,ab,hw,id. (2)

92 (sf20 or sf-20 or sf-twenty or sftwenty).ti,ab,hw,id. (47)

93 or/85-92 (93401)

94 (attitudes to children with epilepsy$ or aatcwe$ or disabkids$ or eldqol$ or "epilepsy and children questionnaire$" or ecq$ or "epilepsy foundation$ of america concern$ index$" or efa or efaci or glasgow epilepsy outcome scale$ or geos$ or hague restrictions in childhood epilepsy scale$ or harces$ or cheqol$ or modified impact of epilepsy schedule$ or mioes or impact of childhood illness scale$ or ici$ or impact of Paediatric epilepsy scale$ or impact of paediatric epilepsy scale$ or ipes$ or impact of childhood neurologic disability$ or icnd$ or neuroqol$ or newcastle mitochondrial disease$ or nmds$ or newcastle adult mitochondrial disease$ or nmdas$ or nmdsa$ or newcastle Paediatric mitochondrial disease$ or newcastle paediatric mitochondrial disease$ or npmds or pedsql$ or peds-ql$ or pedsqlem$ or peds-qlem$ or Paediatric epilepsy side effects questionnaire$ or paediatric epilepsy side effects questionnaire$ or pesq$ or patient-reported outcomes measurement information system$ or promis or promis25 or promis37 or promis49 or qolce$ or gqolce$ or qolie$ or qolpes$).ti,ab,hw,id. (8521)

95 "Quality of Life Measures"/ (690)

96 or/93-95 (100130)

97 66 and 84 (21)

98 66 and 96 (86)

99 97 or 98 (90)

1. **Source: ScHARRHud**

Interface / URL: http://www.scharrhud.org/

Database coverage dates: Information not found

Search date: 13/08/2021

Retrieved records: 0

Search strategy:

The basic search interface at the following URL was used: https://www.scharrhud.org/index.php?recordsN1&m=search. All search settings were left as default. In the default setting, terms are searched across 'Any field'.

Separate searches were conducted on each of the following terms. Returned results were screened by the information specialist for potential relevance to the populations of interest. Potentially relevant results were cross-checked against results from other search sources to see if already retrieved. Any potentially relevant results not identified via another source were retrieved for further assessment.

mitochondri* = 0 (0 results returned)

leigh* OR encephal* = 0 (2 results returned, excluded)

polymerase* AND gamma = 0 (0 results returned)

polg* = 0 (1 result returned, excluded)

alper* = 0 (0 results returned)

schilder* = 0 (0 results returned)

concentric scleros* OR poliodystroph* OR diffuse cerebral OR encephalitis periaxialis OR myelinoclastic diffuse OR progressive neuronal OR sudanophilic cerebral = 0 (0 results returned)

ahs = 0 (0 results returned)

myocerebrohepatopath* OR mchs = 0 (0 results returned)

myoclon* AND epilep* AND sensory ataxia* = 0 (0 results returned)

memsa = 0 (1 result returned, excluded)

spinocerebellar ataxia* = 0 (0 results returned)

scae = 0 (0 results returned)

external ophthalmoplegi* = 0 (0 results returned)

ocular muscular dystroph* = 0 (0 results returned)

peo OR cpeo* OR arpeo OR adpeo OR graefe* OR ataxia neuropath* OR ataxic neuropath* OR miras OR sando OR melas OR myoencephalopath* OR merrf OR merff OR fukuhara* OR ragged red = 0 (0 results returned)

rars* = 0 (0 results returned)

pontocerebellar OR pontocerebellum OR pch* = 0 (0 results returned)

pdhc OR pdh OR decarboxylase OR lactic acidosis OR pyruvate = 0 (1 result returned, excluded)

1. **Source: National Institute for Health and Care Excellence (NICE) webpages**

Interface / URL: https://www.nice.org.uk/

Database coverage dates: n/a

Search date: 13/08/2021

Retrieved records: 0

Search strategy:

The following documents were sought: Company Submissions, Final Appraisal Determination Documents, Assessment Reports for multiple technology appraisals, Evidence Review Group (ERG) reports for single technology appraisals.

The site-wide search interface was used at: <https://www.nice.org.uk/>. Separate searches were conducted on each term shown below.

Results were filtered by 'Document Type' to 'Guidance', then by 'Guidance Programme' to 'Technology appraisal guidance.'

Results were scanned to identify technology appraisals on the eligible mitochondrial diseases. For any identified, associated documents under the 'History' tab were viewed to check for relevant documents.

PDFs for relevant documents were retrieved for further assessment. Duplicate documents were not retrieved.

mitochondri* = 0 (1 result returned, excluded)

leigh* = 0 (8 results returned, excluded)

subacute = 0 (1 result returned, excluded)

"sub-acute" = 0 (1 result returned, excluded)

polymerase* AND gamma = 0 (1 result returned, excluded)

polg* = 0 (0 results returned)

alper* OR schilder* OR concentric OR poliodystroph* = 0 (0 results returned)

"diffuse cerebral" OR "encephalitis periaxialis" OR "myelinoclastic diffuse" OR "progressive neuronal" OR "sudanophilic cerebral" = 0 (0 results returned)

ahs = 0 (0 results returned)

myocerebrohepatopath* OR mchs = 0 (0 results returned)

myoclon* AND epilep* AND ataxia* = 0 (0 results returned)

memsa = 0 (0 results returned)

spinocerebellar AND epilep* = 0 (0 results returned)

scae = 0 (0 results returned)

external AND ophthalmoplegi* = 0 (0 results returned)

"ocular muscular" = 0 (0 results returned)

peo OR cpeo* OR arpeo OR adpeo = 0 (0 results returned)

graefe* OR ataxia OR ataxic OR miras OR sando OR melas OR myoencephalopath* OR merrf OR merff OR fukuhara* OR "ragged red" OR rars* OR pontocerebellar OR pontocerebellum OR pch* OR "pyruvate dehydrogenase" OR pdhc OR pdh OR decarboxylase = 0 (1 result returned, excluded)

0 documents were retrieved

1. **Source: Institute for Clinical and Economic Review webpages**

Interface / URL: https://icer-review.org/

Database coverage dates: n/a

Search date: 23/08/2021

Retrieved records: 0

Search strategy:

The following were sought: Final Evidence Report (or Draft Evidence Report if Final was not available) and the Evidence Presentation (if available).

Assessments were located at the following URL: <https://icer.org/explore-our-research/assessments/>.

The drop-down options were used to view all documents with status 'completed' and research type 'assessment'. 84 results were returned.

The descriptive text was checked for relevance to the eligible mitochondrial diseases. No documents were retrieved for further assessment.

1. **Source: Canadian Agency for Drugs and Technologies in Health (CADTH) webpages**

Interface / URL: https://www.cadth.ca/

Database coverage dates: n/a

Search date: 23/08/2021

Retrieved records: 0

Search strategy:

The following documents were sought: economic guidance and final recommendations associated with reimbursement reviews.

The site-wide search interface was used at: <https://www.cadth.ca/>. 'Advanced Search' was selected. Separate searches were conducted on each term shown below.

Results were filtered by 'Project Line' to 'Reimbursement Review'.

Results were assessed for relevance to the eligible mitochondrial diseases. Relevant results were checked for document types of interest.

PDFs of relevant documents were retrieved for further assessment. Duplicate documents were not retrieved.

mitochondri* = 0 (7 results returned, excluded)

leigh* = 0 (13 results returned, excluded)

subacute AND encephal* = 0 (0 results returned)

"sub-acute" AND encephal* = 0 (0 results returned)

polymerase* AND gamma = 0 (2 results returned, excluded)

polg* = 0 (0 results returned)

alper* OR schilder* OR concentric OR poliodystroph* = 0 (2 results returned, excluded)

"diffuse cerebral" OR "encephalitis periaxialis" OR "myelinoclastic diffuse" OR "progressive neuronal" = 0 (0 results returned)

"sudanophilic cerebral" = 0 (0 results returned)

ahs = 0 (1 result returned, excluded)

myocerebrohepatopath* OR mchs = 0 (0 results returned)

myoclon* AND epilep* AND ataxia* = 0 (3 results returned, excluded)

memsa = 0 (0 results returned)

spinocerebellar AND epilep* = 0 (0 results returned)

scae = 0 (0 results returned)

external AND ophthalmoplegi* = 0 (1 result returned, excluded)

"ocular muscular" = 0 (0 results returned)

peo OR cpeo* OR arpeo OR adpeo = 0 (0 results returned)

graefe* OR ataxia OR ataxic OR miras OR sando = 0 (11 results returned, excluded)

melas OR myoencephalopath* OR merrf OR merff OR fukuhara* OR "ragged red" = 0 (0 results returned)

rars* OR pontocerebellar OR pontocerebellum OR pch* = 0 (3 results returned, excluded)

"pyruvate dehydrogenase" OR pdhc OR pdh OR decarboxylase = 0 (0 results returned)

0 documents were retrieved

#### Update searches: July 2022

1. **Source: MEDLINE ALL**

Interface / URL: OvidSP

Database coverage dates: 1946 to July 22, 2022

Search date: 25/07/2022

Retrieved records: 589

Search strategy:

1 mitochondrial diseases/ (6265)

2 mitochondrial myopathies/ or mitochondrial encephalomyopathies/ (2801)

3 (mitochondri$ adj6 (disease$ or disorder$ or syndrome$)).ti,ab,kf. (19516)

4 (mitochondri$ adj6 (cytopath$ or deficien$ or dysfunction$ or encephalomyopath$ or encephalopath$ or myopath$)).ti,ab,kf. (40843)

5 (mitochondriopath$ or (mdas not modified dental anxiety scale)).ti,ab,kf. (897)

6 (mitochondri$ adj6 (epilep$ or seizure$ or convuls$)).ti,ab,kf. (594)

7 or/1-6 (54633)

8 Leigh Disease/ (1199)

9 ((leigh$ or leigh-feigin$ or leigh-feigin-wolf$) adj6 (disease$ or disorder$ or syndrome$)).ti,ab,kf. (1740)

10 ((leigh$ or leigh-feigin$ or leigh-feigin-wolf$) adj6 (encephalopath$ or encephalomyelopath$)).ti,ab,kf. (317)

11 ((subacute or sub-acute) adj3 (necrot$ encephal$ or necrot$ juvenile encephal$ or necrot$ infantile encephal$)).ti,ab,kf. (265)

12 (leigh$ and mitochondri$).ti,ab,kf. (1261)

13 or/8-12 (2103)

14 DNA Polymerase gamma/ (847)

15 (polymerase$ adj3 gamma).ti,ab,kf. (1343)

16 (polg or polg1).ti,ab,kf. (918)

17 (polgone or polgi).ti,ab,kf. (0)

18 (mitochondri$ adj6 polymerase$).ti,ab,kf. (1911)

19 or/14-18 (3242)

20 "Diffuse Cerebral Sclerosis of Schilder"/ (2376)

21 ((alper$ or alper-huttenlocher$ or alpers-huttenlocher$) adj6 (disease$ or disorder$ or syndrome$)).ti,ab,kf. (250)

22 ((alper$ or alper-huttenlocher$ or alpers-huttenlocher$) adj6 (diffuse cerebral or diffuse degeneration or poliodystroph$)).ti,ab,kf. (19)

23 (schilder$ adj6 (disease$ or disorder$ or syndrome$)).ti,ab,kf. (182)

24 (schilder$ adj6 (diffuse cerebral or diffuse degeneration or poliodystroph$)).ti,ab,kf. (5)

25 (balo$ adj3 concentric scleros$).ti,ab,kf. (191)

26 (progressive adj3 poliodystroph$).ti,ab,kf. (18)

27 (diffuse cerebral degeneration$ or diffuse cerebral scleros$ or encephalitis periaxialis or myelinoclastic diffuse scleros$ or poliodystrophia cerebri or progressive neuronal degeneration$ or sudanophilic cerebral scleros$).ti,ab,kf. (337)

28 ahs.ti,ab,kf. (1683)

29 or/20-28 (4385)

30 myocerebrohepatopath$.ti,ab,kf. (13)

31 mchs.ti,ab,kf. (100)

32 or/30-31 (106)

33 Spinocerebellar Ataxias/ and exp Epilepsy/ (68)

34 (myoclon$ and epilep$ and sensory ataxia$).ti,ab,kf. (11)

35 memsa.ti,ab,kf. (14)

36 (spinocerebellar ataxia$ and epilep$).ti,ab,kf. (181)

37 scae.ti,ab,kf. (58)

38 or/33-37 (292)

39 Ophthalmoplegia, Chronic Progressive External/ (606)

40 external ophthalmoplegi$.ti,ab,kf. (1713)

41 ocular muscular dystroph$.ti,ab,kf. (10)

42 (peo or cpeo$ or arpeo or adpeo).ti,ab,kf. (6430)

43 ((graefe$ or graefe fuch$) adj6 (disease$ or disorder$ or syndrome$)).ti,ab,kf. (11)

44 ((graefe$ or graefe fuch$) adj6 myopath$).ti,ab,kf. (2)

45 or/39-44 (7805)

46 ataxia neuropath$.ti,ab,kf. (146)

47 ataxic neuropath$.ti,ab,kf. (261)

48 miras.ti,ab,kf. (134)

49 sando.ti,ab,kf. (73)

50 or/46-49 (572)

51 MELAS Syndrome/ (1440)

52 melas.ti,ab,kf. (2238)

53 myoencephalopath$.ti,ab,kf. (10)

54 or/51-53 (2565)

55 MERRF Syndrome/ (390)

56 merrf.ti,ab,kf. (536)

57 merff.ti,ab,kf. (13)

58 fukuhara$.ti,ab,kf. (42)

59 (epilep$ and ragged red).ti,ab,kf. (503)

60 or/55-59 (837)

61 Arginine-tRNA Ligase/ (201)

62 (mitochondri$ and arg$ and (tRNA or RNA or ribonucleic acid)).ti,ab,kf. (593)

63 (rars2 or rars-2).ti,ab,kf. (56)

64 (rarstwo or rars-two or rarsii or rars-ii or rars11 or rars-11).ti,ab,kf. (5)

65 or/61-64 (808)

66 Olivopontocerebellar Atrophies/ or (pontocerebellar adj6 hypoplasia$).ti,ab,kf. (940)

67 (pontocerebellum adj6 hypoplasia$).ti,ab,kf. (0)

68 (pch6 or pch-6).ti,ab,kf. (39)

69 (pchsix or pch-six or pchvi or pch-vi).ti,ab,kf. (1)

70 or/66-69 (958)

71 Pyruvate Dehydrogenase Complex Deficiency Disease/ (414)

72 (pyruvate dehydrogenase adj6 deficien$).ti,ab,kf. (522)

73 ((pdhc or pdh or pyruvate decarboxylase) adj6 deficien$).ti,ab,kf. (248)

74 ((ataxia or ataxic) adj6 (lactic acidosis or pyruvate or decarboxylase)).ti,ab,kf. (119)

75 or/71-74 (831)

76 7 or 13 or 19 or 29 or 32 or 38 or 45 or 50 or 54 or 60 or 65 or 70 or 75 (72991)

77 Quality-Adjusted Life Years/ (15049)

78 (quality adjusted or adjusted life year$).ti,ab,kf. (21378)

79 (qaly$ or qald$ or qale$ or qtime$).ti,ab,kf. (13479)

80 (illness state$1 or health state$1).ti,ab,kf. (7805)

81 (hui or hui1 or hui2 or hui3).ti,ab,kf. (1833)

82 (multiattribute$ or multi attribute$).ti,ab,kf. (1171)

83 (utility adj3 (score$1 or valu$ or health$ or cost$ or measur$ or disease$ or mean or gain or gains or index$)).ti,ab,kf. (18633)

84 utilities.ti,ab,kf. (8681)

85 (eq-5d or eq5d or eq-5 or eq5 or euro qual or euroqual or euro qual5d or euroqual5d or euro qol or euroqol or euro qol5d or euroqol5d or euro quol or euroquol or euro quol5d or euroquol5d or eur qol or eurqol or eur qol5d or eur qol5d or eur?qul or eur?qul5d or euro$ quality of life or european qol).ti,ab,kf. (15512)

86 (euro$ adj3 (5 d or 5d or 5 dimension$ or 5dimension$ or 5 domain$ or 5domain$)).ti,ab,kf. (5389)

87 (sf36$ or sf 36$ or sf thirtysix or sf thirty six).ti,ab,kf. (25319)

88 (time trade off$1 or time tradeoff$1 or tto or timetradeoff$1).ti,ab,kf. (2225)

89 quality of life/ and ((quality of life or qol) adj (score$1 or measure$1)).ti,ab,kf. (14571)

90 quality of life/ and ec.fs. (10872)

91 quality of life/ and (health adj3 status).ti,ab,kf. (11072)

92 (quality of life or qol).ti,ab,kf. and Cost-Benefit Analysis/ (15986)

93 ((qol or hrqol or quality of life).ti,kf. or *quality of life/) and ((qol or hrqol$ or quality of life) adj2 (increas$ or decrease$ or improv$ or declin$ or reduc$ or high$ or low$ or effect or effects or worse or score or scores or change$1 or impact$1 or impacted or deteriorat$)).ab. (48808)

94 Cost-Benefit Analysis/ and (cost-effectiveness ratio$ and (perspective$ or life expectanc$)).ti,ab,kf. (4812)

95 *quality of life/ and (quality of life or qol).ti. (62435)

96 quality of life/ and ((quality of life or qol) adj3 (improv$ or chang$)).ti,ab,kf. (37342)

97 quality of life/ and health-related quality of life.ti,ab,kf. (41448)

98 models,economic/ (11016)

99 or/77-98 (205171)

100 (utility loss$ or disutility$).ti,ab,kf. (563)

101 (ahum or aqol$ or chu9d or chu-9d or eq-5dy or eq5dy or health status classification system$ or hscs-ps or hscsps or hui or hui1 or hui2 or hui3 or quality of well-being or quality of wellbeing or qwb or 15d or 15-d or 15 dimension$ or 16d or 16-d or 16 dimension$ or 17d or 17-d or 17 dimension$).ti,ab,kf. (12577)

102 99 or 100 or 101 (214987)

103 "Quality of Life"/ (248211)

104 ((quality adj3 life) or qol).ti,ab,kf. (359744)

105 (hql or hqol or hrqol or hrql or hr-ql).ti,ab,kf. (25009)

106 (short form$ or shortform$).ti,ab,kf. (40947)

107 (sf6 or sf-6 or sf6d or sf-6d or sf-six or sfsix).ti,ab,kf. (3202)

108 (sf8 or sf-8 or sf-eight or sfeight).ti,ab,kf. (589)

109 (sf12 or sf-12 or sf-twelve or sftwelve).ti,ab,kf. (5847)

110 (sf16 or sf-16 or sf-sixteen or sfsixteen).ti,ab,kf. (32)

111 (sf20 or sf-20 or sf-twenty or sftwenty).ti,ab,kf. (352)

112 or/103-111 (444720)

113 (attitudes to children with epilepsy$ or aatcwe$ or disabkids$ or eldqol$ or "epilepsy and children questionnaire$" or ecq$ or "epilepsy foundation$ of america concern$ index$" or efa or efaci or glasgow epilepsy outcome scale$ or geos$ or hague restrictions in childhood epilepsy scale$ or harces$ or cheqol$ or modified impact of epilepsy schedule$ or mioes or impact of childhood illness scale$ or ici$ or impact of pediatric epilepsy scale$ or impact of paediatric epilepsy scale$ or ipes$ or impact of childhood neurologic disability$ or icnd$ or neuroqol$ or newcastle mitochondrial disease$ or nmds$ or newcastle adult mitochondrial disease$ or nmdas$ or nmdsa$ or newcastle pediatric mitochondrial disease$ or newcastle paediatric mitochondrial disease$ or npmds or pedsql$ or peds-ql$ or pedsqlem$ or peds-qlem$ or pediatric epilepsy side effects questionnaire$ or paediatric epilepsy side effects questionnaire$ or pesq$ or patient-reported outcomes measurement information system$ or promis or promis25 or promis37 or promis49 or qolce$ or gqolce$ or qolie$ or qolpes$).ti,ab,kf. (43983)

114 112 or 113 (482293)

115 76 and 102 (156)

116 76 and 114 (656)

117 115 or 116 (709)

118 exp Animals/ not Humans/ (5039010)

119 117 not 118 (644)

120 limit 119 to english language (589)

1. **Source: Embase**

Interface / URL: OvidSP

Database coverage dates: 1974 to 2022 July 22

Search date: 25/07/2022

Retrieved records: 914

Search strategy:

1 "disorders of mitochondrial functions"/ (25423)

2 mitochondrial dna disorder/ or mitochondrial encephalomyopathy/ or mitochondrial encephalopathy/ or mitochondrial myopathy/ (5106)

3 (mitochondri$ adj6 (disease$ or disorder$ or syndrome$)).ti,ab,kw,dq. (24689)

4 (mitochondri$ adj6 (cytopath$ or deficien$ or dysfunction$ or encephalomyopath$ or encephalopath$ or myopath$)).ti,ab,kw,dq. (52533)

5 (mitochondriopath$ or (mdas not modified dental anxiety scale)).ti,ab,kw,dq. (1364)

6 (mitochondri$ adj6 (epilep$ or seizure$ or convuls$)).ti,ab,kw,dq. (1014)

7 or/1-6 (76517)

8 Leigh disease/ (2850)

9 ((leigh$ or leigh-feigin$ or leigh-feigin-wolf$) adj6 (disease$ or disorder$ or syndrome$)).ti,ab,kw,dq. (2331)

10 ((leigh$ or leigh-feigin$ or leigh-feigin-wolf$) adj6 (encephalopath$ or encephalomyelopath$)).ti,ab,kw,dq. (395)

11 ((subacute or sub-acute) adj3 (necrot$ encephal$ or necrot$ juvenile encephal$ or necrot$ infantile encephal$)).ti,ab,kw,dq. (254)

12 (leigh$ and mitochondri$).ti,ab,kw,dq. (1888)

13 or/8-12 (3431)

14 DNA directed DNA polymerase gamma/ (2326)

15 (polymerase$ adj3 gamma).ti,ab,kw,dq. (1610)

16 (polg or polg1).ti,ab,kw,dq. (1644)

17 (polgone or polgi).ti,ab,kw,dq. (4)

18 (mitochondri$ adj6 polymerase$).ti,ab,kw,dq. (2326)

19 or/14-18 (5487)

20 Alpers disease/ or Schilder disease/ (726)

21 ((alper$ or alper-huttenlocher$ or alpers-huttenlocher$) adj6 (disease$ or disorder$ or syndrome$)).ti,ab,kw,dq. (357)

22 ((alper$ or alper-huttenlocher$ or alpers-huttenlocher$) adj6 (diffuse cerebral or diffuse degeneration or poliodystroph$)).ti,ab,kw,dq. (18)

23 (schilder$ adj6 (disease$ or disorder$ or syndrome$)).ti,ab,kw,dq. (168)

24 (schilder$ adj6 (diffuse cerebral or diffuse degeneration or poliodystroph$)).ti,ab,kw,dq. (8)

25 (balo$ adj3 concentric scleros$).ti,ab,kw,dq. (274)

26 (progressive adj3 poliodystroph$).ti,ab,kw,dq. (12)

27 (diffuse cerebral degeneration$ or diffuse cerebral scleros$ or encephalitis periaxialis or myelinoclastic diffuse scleros$ or poliodystrophia cerebri or progressive neuronal degeneration$ or sudanophilic cerebral scleros$).ti,ab,kw,dq. (231)

28 ahs.ti,ab,kw,dq. (2306)

29 or/20-28 (3574)

30 myocerebrohepatopath$.ti,ab,kw,dq. (25)

31 mchs.ti,ab,kw,dq. (131)

32 or/30-31 (148)

33 spinocerebellar degeneration/ and exp epilepsy/ (518)

34 (myoclon$ and epilep$ and sensory ataxia$).ti,ab,kw,dq. (24)

35 memsa.ti,ab,kw,dq. (19)

36 (spinocerebellar ataxia$ and epilep$).ti,ab,kw,dq. (287)

37 scae.ti,ab,kw,dq. (88)

38 or/33-37 (763)

39 chronic progressive external ophthalmoplegia/ (1061)

40 external ophthalmoplegi$.ti,ab,kw,dq. (2134)

41 ocular muscular dystroph$.ti,ab,kw,dq. (6)

42 (peo or cpeo$ or arpeo or adpeo).ti,ab,kw,dq. (5914)

43 ((graefe$ or graefe fuch$) adj6 (disease$ or disorder$ or syndrome$)).ti,ab,kw,dq. (12)

44 ((graefe$ or graefe fuch$) adj6 myopath$).ti,ab,kw,dq. (3)

45 or/39-44 (7745)

46 ataxia neuropath$.ti,ab,kw,dq. (258)

47 ataxic neuropath$.ti,ab,kw,dq. (400)

48 miras.ti,ab,kw,dq. (131)

49 sando.ti,ab,kw,dq. (119)

50 or/46-49 (831)

51 MELAS syndrome/ (3103)

52 melas.ti,ab,kw,dq. (3018)

53 myoencephalopath$.ti,ab,kw,dq. (12)

54 or/51-53 (4137)

55 MERRF syndrome/ (750)

56 merrf.ti,ab,kw,dq. (706)

57 merff.ti,ab,kw,dq. (36)

58 fukuhara$.ti,ab,kw,dq. (72)

59 (epilep$ and ragged red).ti,ab,kw,dq. (623)

60 or/55-59 (1315)

61 arginine transfer RNA ligase/ (236)

62 (mitochondri$ and arg$ and (tRNA or RNA or ribonucleic acid)).ti,ab,kw,dq. (685)

63 (rars2 or rars-2).ti,ab,kw,dq. (136)

64 (rarstwo or rars-two or rarsii or rars-ii or rars11 or rars-11).ti,ab,kw,dq. (7)

65 or/61-64 (997)

66 cerebellum hypoplasia/ or olivopontocerebellar atrophy/ or (pontocerebellar adj6 hypoplasia$).ti,ab,kw,dq. (3482)

67 (pontocerebellum adj6 hypoplasia$).ti,ab,kw,dq. (5)

68 (pch6 or pch-6).ti,ab,kw,dq. (46)

69 (pchsix or pch-six or pchvi or pch-vi).ti,ab,kw,dq. (1)

70 or/66-69 (3498)

71 pyruvate dehydrogenase complex deficiency/ (489)

72 (pyruvate dehydrogenase adj6 deficien$).ti,ab,kw,dq. (696)

73 ((pdhc or pdh or pyruvate decarboxylase) adj6 deficien$).ti,ab,kw,dq. (349)

74 ((ataxia or ataxic) adj6 (lactic acidosis or pyruvate or decarboxylase)).ti,ab,kw,dq. (152)

75 or/71-74 (1122)

76 7 or 13 or 19 or 29 or 32 or 38 or 45 or 50 or 54 or 60 or 65 or 70 or 75 (99085)

77 quality adjusted life year/ (32012)

78 (quality adjusted or adjusted life year$).ti,ab,kw,dq. (30550)

79 (qaly$ or qald$ or qale$ or qtime$).ti,ab,kw,dq. (24651)

80 (illness state$1 or health state$1).ti,ab,kw,dq. (13394)

81 (hui or hui1 or hui2 or hui3).ti,ab,kw,dq. (2838)

82 (multiattribute$ or multi attribute$).ti,ab,kw,dq. (1401)

83 (utility adj3 (score$1 or valu$ or health$ or cost$ or measur$ or disease$ or mean or gain or gains or index$)).ti,ab,kw,dq. (28747)

84 utilities.ti,ab,kw,dq. (13874)

85 exp "european quality of life 5 dimensions questionnaire"/ (10365)

86 (eq-5d or eq5d or eq-5 or eq5 or euro qual or euroqual or euro qual5d or euroqual5d or euro qol or euroqol or euro qol5d or euroqol5d or euro quol or euroquol or euro quol5d or euroquol5d or eur qol or eurqol or eur qol5d or eur qol5d or eur?qul or eur?qul5d or euro$ quality of life or european qol).ti,ab,kw,dq. (27867)

87 (euro$ adj3 (5 d or 5d or 5 dimension$ or 5dimension$ or 5 domain$ or 5domain$)).ti,ab,kw,dq. (8254)

88 short form 36/ (35410)

89 (sf36$ or sf 36$ or sf thirtysix or sf thirty six).ti,ab,kw,dq. (43071)

90 (time trade off$1 or time tradeoff$1 or tto or timetradeoff$1).ti,ab,kw,dq. (3254)

91 quality of life/ and ((quality of life or qol) adj (score$1 or measure$1)).ti,ab,kw,dq. (30358)

92 quality of life/ and (pe or de).fs. (9726)

93 quality of life/ and (health adj3 status).ti,ab,kw,dq. (18454)

94 (quality of life or qol).ti,ab,kw,dq. and cost benefit analysis/ (6459)

95 ((qol or hrqol or quality of life).ti,kw,dq. or *quality of life/) and ((qol or hrqol$ or quality of life) adj2 (increas$ or decrease$ or improv$ or declin$ or reduc$ or high$ or low$ or effect or effects or worse or score or scores or change$1 or impact$1 or impacted or deteriorat$)).ab. (69697)

96 cost benefit analysis/ and (cost-effectiveness ratio$ and (perspective$ or life expectanc$)).ti,ab,kw,dq. (1103)

97 *quality of life/ and (quality of life or qol).ti. (101356)

98 quality of life/ and ((quality of life or qol) adj3 (improv$ or chang$)).ti,ab,kw,dq. (91799)

99 quality of life/ and health-related quality of life.ti,ab,kw,dq. (70962)

100 economic model/ (2846)

101 or/77-100 (348988)

102 (utility loss$ or disutility$).ti,ab,kw,dq. (991)

103 (ahum or aqol$ or chu9d or chu-9d or eq-5dy or eq5dy or health status classification system$ or hscs-ps or hscsps or hui or hui1 or hui2 or hui3 or quality of well-being or quality of wellbeing or qwb or 15d or 15-d or 15 dimension$ or 16d or 16-d or 16 dimension$ or 17d or 17-d or 17 dimension$).ti,ab,kw,dq. (16138)

104 101 or 102 or 103 (360988)

105 "quality of life"/ (563639)

106 "quality of life assessment"/ (12914)

107 ((quality adj3 life) or qol).ti,ab,kw,dq. (556752)

108 (hql or hqol or hrqol or hrql or hr-ql).ti,ab,kw,dq. (40257)

109 (short form$ or shortform$).ti,ab,kw,dq. (56140)

110 short form 12/ or short form 20/ or short form 8/ (9147)

111 (sf6 or sf-6 or sf6d or sf-6d or sf-six or sfsix).ti,ab,kw,dq. (4204)

112 (sf8 or sf-8 or sf-eight or sfeight).ti,ab,kw,dq. (958)

113 (sf12 or sf-12 or sf-twelve or sftwelve).ti,ab,kw,dq. (9760)

114 (sf16 or sf-16 or sf-sixteen or sfsixteen).ti,ab,kw,dq. (61)

115 (sf20 or sf-20 or sf-twenty or sftwenty).ti,ab,kw,dq. (366)

116 or/105-115 (748863)

117 (attitudes to children with epilepsy$ or aatcwe$ or disabkids$ or eldqol$ or "epilepsy and children questionnaire$" or ecq$ or "epilepsy foundation$ of america concern$ index$" or efa or efaci or glasgow epilepsy outcome scale$ or geos$ or hague restrictions in childhood epilepsy scale$ or harces$ or cheqol$ or modified impact of epilepsy schedule$ or mioes or impact of childhood illness scale$ or ici$ or impact of pediatric epilepsy scale$ or impact of paediatric epilepsy scale$ or ipes$ or impact of childhood neurologic disability$ or icnd$ or neuroqol$ or newcastle mitochondrial disease$ or nmds$ or newcastle adult mitochondrial disease$ or nmdas$ or nmdsa$ or newcastle pediatric mitochondrial disease$ or newcastle paediatric mitochondrial disease$ or npmds or pedsql$ or peds-ql$ or pedsqlem$ or peds-qlem$ or pediatric epilepsy side effects questionnaire$ or paediatric epilepsy side effects questionnaire$ or pesq$ or patient-reported outcomes measurement information system$ or promis or promis25 or promis37 or promis49 or qolce$ or gqolce$ or qolie$ or qolpes$).ti,ab,kw,dq. or "pediatric quality of life inventory"/ (58836)

118 116 or 117 (794836)

119 76 and 104 (364)

120 76 and 118 (1303)

121 119 or 120 (1402)

122 (animal/ or animal experiment/ or animal model/ or animal tissue/ or nonhuman/) not exp human/ (6498357)

123 conference abstract.pt. (4456034)

124 121 not (122 or 123) (946)

125 limit 124 to english language (914)

1. **Source: Cochrane Database of Systematic Reviews (CDSR)**

Interface / URL: Cochrane Library / Wiley

Database coverage dates: Information not found. Issue searched: Issue 7 of 12, July 2022

Search date: 26/07/2022

Retrieved records: 8

Search strategy:

#1 MeSH descriptor: [Mitochondrial Diseases] this term only 56

#2 MeSH descriptor: [Mitochondrial Myopathies] this term only 34

#3 MeSH descriptor: [Mitochondrial Encephalomyopathies] this term only 10

#4 (mitochondri* NEAR/6 (disease* OR disorder* OR syndrome*)):ti,ab,kw 358

#5 (mitochondri* NEAR/6 (cytopath* OR deficien* OR dysfunction* OR encephalomyopath* OR encephalopath* OR myopath*)):ti,ab,kw 522

#6 (mitochondriopath* OR (mdas not "modified dental anxiety scale")):ti,ab,kw 91

#7 (mitochondri* NEAR/6 (epilep* OR seizure* OR convuls*)):ti,ab,kw 10

#8 #1 OR #2 OR #3 OR #4 OR #5 OR #6 OR #7 831

#9 MeSH descriptor: [Leigh Disease] this term only 4

#10 ((leigh* OR leigh-feigin* OR leigh-feigin-wolf*) NEAR/6 (disease* OR disorder* OR syndrome*)):ti,ab,kw 11

#11 ((leigh* OR leigh-feigin* OR leigh-feigin-wolf*) NEAR/6 (encephalopath* OR encephalomyelopath*)):ti,ab,kw 0

#12 ((subacute OR sub-acute) NEAR/3 (necrot* NEXT encephal* OR necrot* NEXT juvenile NEXT encephal* OR necrot* NEXT infantile NEXT encephal*)):ti,ab,kw 0

#13 (leigh* AND mitochondri*):ti,ab,kw 8

#14 #9 OR #10 OR #11 OR #12 OR #13 11

#15 MeSH descriptor: [DNA Polymerase gamma] this term only 1

#16 (polymerase* NEAR/3 gamma):ti,ab,kw 12

#17 (polg OR polg1):ti,ab,kw 5

#18 (polgone OR polgi):ti,ab,kw 0

#19 (mitochondri* NEAR/6 polymerase*):ti,ab,kw 21

#20 #15 or #16 or #17 or #18 or #19 26

#21 MeSH descriptor: [Diffuse Cerebral Sclerosis of Schilder] this term only 7

#22 ((alper* OR alper-huttenlocher* OR alpers-huttenlocher*) NEAR/6 (disease* OR disorder* OR syndrome*)):ti,ab,kw 3

#23 ((alper* OR alper-huttenlocher* OR alpers-huttenlocher*) NEAR/6 ("diffuse cerebral" OR "diffuse degeneration" OR poliodystroph*)):ti,ab,kw 0

#24 (schilder* NEAR/6 (disease* OR disorder* OR syndrome*)):ti,ab,kw 1

#25 (schilder* NEAR/6 ("diffuse cerebral" OR "diffuse degeneration" OR poliodystroph*)):ti,ab,kw 7

#26 (balo* NEAR/3 (concentric NEXT scleros*)):ti,ab,kw 1

#27 (progressive NEAR/3 poliodystroph*):ti,ab,kw 0

#28 (diffuse NEXT cerebral NEXT degeneration* OR diffuse NEXT cerebral NEXT scleros* OR "encephalitis periaxialis" OR myelinoclastic NEXT diffuse NEXT scleros* OR "poliodystrophia cerebri" OR progressive NEXT neuronal NEXT degeneration* OR sudanophilic NEXT cerebral NEXT scleros*):ti,ab,kw 10

#29 ahs:ti,ab,kw 112

#30 #21 OR #22 OR #23 OR #24 OR #25 OR #26 OR #27 OR #28 OR #29 127

#31 myocerebrohepatopath*:ti,ab,kw 0

#32 mchs:ti,ab,kw 4

#33 #31 OR #32 4

#34 MeSH descriptor: [Spinocerebellar Ataxias] this term only 59

#35 MeSH descriptor: [Epilepsy] explode all trees 2597

#36 #34 AND #35 0

#37 (myoclon* AND epilep* AND sensory NEXT ataxia*):ti,ab,kw 0

#38 memsa:ti,ab,kw 8

#39 (spinocerebellar NEXT ataxia* AND epilep*):ti,ab,kw 1

#40 scae:ti,ab,kw 5

#41 #36 OR #37 OR #38 OR #39 OR #40 14

#42 MeSH descriptor: [Ophthalmoplegia, Chronic Progressive External] this term only 6

#43 external NEXT ophthalmoplegi*:ti,ab,kw 17

#44 ocular NEXT muscular NEXT dystroph*:ti,ab,kw 0

#45 (peo OR cpeo* OR arpeo OR adpeo):ti,ab,kw 35

#46 ((graefe* OR graefe NEXT fuch*) NEAR/6 (disease* OR disorder* OR syndrome*)):ti,ab,kw 1

#47 ((graefe* OR graefe NEXT fuch*) NEAR/6 myopath*):ti,ab,kw 0

#48 #42 OR #43 OR #44 OR #45 OR #46 OR #47 48

#49 ataxia NEXT neuropath*:ti,ab,kw 2

#50 ataxic NEXT neuropath*:ti,ab,kw 8

#51 miras:ti,ab,kw 11

#52 sando:ti,ab,kw 1

#53 #49 OR #50 OR #51 OR #52 22

#54 MeSH descriptor: [MELAS Syndrome] this term only 12

#55 melas:ti,ab,kw 58

#56 myoencephalopath*:ti,ab,kw 0

#57 #54 OR #55 OR #56 58

#58 MeSH descriptor: [MERRF Syndrome] this term only 0

#59 merrf:ti,ab,kw 2

#60 merff:ti,ab,kw 0

#61 fukuhara*:ti,ab,kw 1

#62 (epilep* AND "ragged red"):ti,ab,kw 1

#63 #58 OR #59 OR #60 OR #61 OR #62 4

#64 MeSH descriptor: [Arginine-tRNA Ligase] this term only 0

#65 (mitochondri* AND arg* AND (tRNA OR RNA OR "ribonucleic acid")):ti,ab,kw 4

#66 (rars2 OR rars-2):ti,ab,kw 5

#67 (rarstwo OR rars-two OR rarsii OR rars-ii OR rars11 OR rars-11):ti,ab,kw 0

#68 #64 OR #65 OR #66 OR #67 9

#69 MeSH descriptor: [Olivopontocerebellar Atrophies] this term only 11

#70 (pontocerebellar NEAR/6 hypoplasia*):ti,ab,kw 0

#71 (pontocerebellum NEAR/6 hypoplasia*):ti,ab,kw 0

#72 (pch6 OR pch-6):ti,ab,kw 0

#73 (pchsix OR pch-six OR pchvi OR pch-vi):ti,ab,kw 0

#74 #69 OR #70 OR #71 OR #72 OR #73 11

#75 MeSH descriptor: [Pyruvate Dehydrogenase Complex Deficiency Disease] this term only 4

#76 ("pyruvate dehydrogenase" NEAR/6 deficien*):ti,ab,kw 8

#77 ((pdhc OR pdh OR "pyruvate decarboxylase") NEAR/6 deficien*):ti,ab,kw 3

#78 ((ataxia OR ataxic) NEAR/6 ("lactic acidosis" OR pyruvate OR decarboxylase)):ti,ab,kw 1

#79 #75 OR #76 OR #77 OR #78 12

#80 #8 OR #14 OR #20 OR #30 OR #33 OR #41 OR #48 OR #53 OR #57 OR #63 OR #68 OR #74 OR #79 1111

#81 #80 in Cochrane Reviews, Cochrane Protocols 8

1. **Source: Cochrane Central Register of Controlled Trials (CENTRAL)**

Interface / URL: Cochrane / Wiley

Database coverage dates: Information not found. Issue searched: Issue 7 of 12, July 2022

Search date: 26/07/2022

Retrieved records: 225

Search strategy:

#1 MeSH descriptor: [Mitochondrial Diseases] this term only 56

#2 MeSH descriptor: [Mitochondrial Myopathies] this term only 34

#3 MeSH descriptor: [Mitochondrial Encephalomyopathies] this term only 10

#4 mitochondri* NEAR/6 (disease* OR disorder* OR syndrome*) 434

#5 mitochondri* NEAR/6 (cytopath* OR deficien* OR dysfunction* OR encephalomyopath* OR encephalopath* OR myopath*) 565

#6 (mitochondriopath* OR (mdas not "modified dental anxiety scale")) 109

#7 mitochondri* NEAR/6 (epilep* OR seizure* OR convuls*) 10

#8 #1 OR #2 OR #3 OR #4 OR #5 OR #6 OR #7 943

#9 MeSH descriptor: [Leigh Disease] this term only 4

#10 (leigh* OR leigh-feigin* OR leigh-feigin-wolf*) NEAR/6 (disease* OR disorder* OR syndrome*) 19

#11 (leigh* OR leigh-feigin* OR leigh-feigin-wolf*) NEAR/6 (encephalopath* OR encephalomyelopath*) 1

#12 (subacute OR sub-acute) NEAR/3 (necrot* NEXT encephal* OR necrot* NEXT juvenile NEXT encephal* OR necrot* NEXT infantile NEXT encephal*) 0

#13 leigh* AND mitochondri* 17

#14 #9 OR #10 OR #11 OR #12 OR #13 22

#15 MeSH descriptor: [DNA Polymerase gamma] this term only 1

#16 polymerase* NEAR/3 gamma 25

#17 polg OR polg1 6

#18 polgone OR polgi 0

#19 mitochondri* NEAR/6 polymerase* 29

#20 #15 or #16 or #17 or #18 or #19 45

#21 MeSH descriptor: [Diffuse Cerebral Sclerosis of Schilder] this term only 7

#22 (alper* OR alper-huttenlocher* OR alpers-huttenlocher*) NEAR/6 (disease* OR disorder* OR syndrome*) 4

#23 (alper* OR alper-huttenlocher* OR alpers-huttenlocher*) NEAR/6 ("diffuse cerebral" OR "diffuse degeneration" OR poliodystroph*) 0

#24 schilder* NEAR/6 (disease* OR disorder* OR syndrome*) 8

#25 schilder* NEAR/6 ("diffuse cerebral" OR "diffuse degeneration" OR poliodystroph*) 7

#26 balo* NEAR/3 (concentric NEXT scleros*) 1

#27 progressive NEAR/3 poliodystroph* 0

#28 diffuse NEXT cerebral NEXT degeneration* OR diffuse NEXT cerebral NEXT scleros* OR "encephalitis periaxialis" OR myelinoclastic NEXT diffuse NEXT scleros* OR "poliodystrophia cerebri" OR progressive NEXT neuronal NEXT degeneration* OR sudanophilic NEXT cerebral NEXT scleros* 10

#29 ahs 393

#30 #21 OR #22 OR #23 OR #24 OR #25 OR #26 OR #27 OR #28 OR #29 416

#31 myocerebrohepatopath* 0

#32 mchs 4

#33 #31 OR #32 4

#34 MeSH descriptor: [Spinocerebellar Ataxias] this term only 59

#35 MeSH descriptor: [Epilepsy] explode all trees 2597

#36 #34 AND #35 0

#37 myoclon* AND epilep* AND sensory NEXT ataxia* 0

#38 memsa 30

#39 spinocerebellar NEXT ataxia* AND epilep* 3

#40 scae 5

#41 #36 OR #37 OR #38 OR #39 OR #40 38

#42 MeSH descriptor: [Ophthalmoplegia, Chronic Progressive External] this term only 6

#43 external NEXT ophthalmoplegi* 19

#44 ocular NEXT muscular NEXT dystroph* 0

#45 peo OR cpeo* OR arpeo OR adpeo 48

#46 (graefe* OR graefe NEXT fuch*) NEAR/6 (disease* OR disorder* OR syndrome*) 1

#47 (graefe* OR graefe NEXT fuch*) NEAR/6 myopath* 0

#48 #42 OR #43 OR #44 OR #45 OR #46 OR #47 62

#49 ataxia NEXT neuropath* 3

#50 ataxic NEXT neuropath* 10

#51 miras 92

#52 sando 90

#53 #49 OR #50 OR #51 OR #52 195

#54 MeSH descriptor: [MELAS Syndrome] this term only 12

#55 melas 100

#56 myoencephalopath* 0

#57 #54 OR #55 OR #56 100

#58 MeSH descriptor: [MERRF Syndrome] this term only 0

#59 merrf 4

#60 merff 1

#61 fukuhara* 227

#62 epilep* AND "ragged red" 2

#63 #58 OR #59 OR #60 OR #61 OR #62 232

#64 MeSH descriptor: [Arginine-tRNA Ligase] this term only 0

#65 mitochondri* AND arg* AND (tRNA OR RNA OR "ribonucleic acid") 10

#66 rars2 OR rars-2 5

#67 rarstwo OR rars-two OR rarsii OR rars-ii OR rars11 OR rars-11 0

#68 #64 OR #65 OR #66 OR #67 15

#69 MeSH descriptor: [Olivopontocerebellar Atrophies] this term only 11

#70 pontocerebellar NEAR/6 hypoplasia* 0

#71 pontocerebellum NEAR/6 hypoplasia* 0

#72 pch6 OR pch-6 0

#73 pchsix OR pch-six OR pchvi OR pch-vi 0

#74 #69 OR #70 OR #71 OR #72 OR #73 11

#75 MeSH descriptor: [Pyruvate Dehydrogenase Complex Deficiency Disease] this term only 4

#76 "pyruvate dehydrogenase" NEAR/6 deficien* 11

#77 (pdhc OR pdh OR "pyruvate decarboxylase") NEAR/6 deficien* 3

#78 (ataxia OR ataxic) NEAR/6 ("lactic acidosis" OR pyruvate OR decarboxylase) 1

#79 #75 OR #76 OR #77 OR #78 15

#80 #8 OR #14 OR #20 OR #30 OR #33 OR #41 OR #48 OR #53 OR #57 OR #63 OR #68 OR #74 OR #79 2011

#81 MeSH descriptor: [Quality-Adjusted Life Years] this term only 1485

#82 "quality adjusted" OR adjusted NEXT life NEXT year* 6665

#83 qaly* OR qald* OR qale* OR qtime* 4537

#84 illness NEXT state* OR health NEXT state* 1507

#85 hui OR hui1 OR hui2 OR hui3 2223

#86 multiattribute* OR multi NEXT attribute* 92

#87 utility NEAR/3 (score* OR valu* OR health* OR cost* OR measur* OR disease* OR mean OR gain OR gains OR index*) 4806

#88 utilities 1321

#89 eq-5d OR eq5d OR eq-5 OR eq5 OR "euro qual" OR euroqual OR "euro qual5d" OR euroqual5d OR "euro qol" OR euroqol OR "euro qol5d" OR euroqol5d OR "euro quol" OR euroquol OR "euro quol5d" OR euroquol5d OR "eur qol" OR eurqol OR "eur qol5d" OR "eur qol5d" OR eur?qul OR eur?qul5d OR euro* NEXT "quality of life" OR "european qol" 12629

#90 euro* NEAR/3 ("5 d" OR 5d OR 5 NEXT dimension* OR 5dimension* OR 5 NEXT domain* OR 5domain*) 3733

#91 sf36* OR sf-36* OR "sf thirtysix" OR "sf thirty six" 14715

#92 time NEXT trade NEXT off* OR time NEXT tradeoff* OR tto OR timetradeoff* 307

#93 MeSH descriptor: [Cost-Benefit Analysis] this term only 7762

#94 cost-effectiveness NEXT ratio* AND (perspective* OR life NEXT expectanc*) 1906

#95 #93 AND #94 695

#96 MeSH descriptor: [Models, Economic] this term only 259

#97 #81 OR #82 OR #83 OR #84 OR #85 OR #86 OR #87 OR #88 OR #89 OR #90 OR #91 OR #92 OR #95 OR #96 35574

#98 utility NEXT loss* OR disutility* 72

#99 ahum OR aqol* OR chu9d OR chu-9d OR eq-5dy OR eq5dy OR "health status classification" NEXT system* OR hscs-ps OR hscsps OR hui OR hui1 OR hui2 OR hui3 OR "quality of well-being" OR "quality of wellbeing" OR qwb OR 15d OR "15 d" OR 15 NEXT dimension* OR 16d OR "16 d" OR 16 NEXT dimension* OR 17d OR "17 d" OR 17 NEXT dimension* 9559

#100 #97 OR #98 OR #99 41361

#101 MeSH descriptor: [Quality of Life] this term only 29130

#102 (quality NEAR/3 life) OR qol 143332

#103 hql OR hqol OR hrqol OR hrql OR hr-ql 8253

#104 short NEXT form* OR shortform* 17894

#105 sf6 OR sf-6 OR sf6d OR sf-6d OR sf-six OR sfsix 562

#106 sf8 OR sf-8 OR sf-eight OR sfeight 272

#107 sf12 OR sf-12 OR sf-twelve OR sftwelve 2981

#108 sf16 OR sf-16 OR sf-sixteen OR sfsixteen 11

#109 sf20 OR sf-20 OR sf-twenty OR sftwenty 91

#110 #101 OR #102 OR #103 OR #104 OR #105 OR #106 OR #107 OR #108 OR #109 150596

#111 "attitudes to children with" NEXT epilepsy* OR aatcwe* OR disabkids* OR eldqol* OR (epilepsy AND (children NEXT questionnaire*)) OR ecq* OR epilepsy NEXT foundation* NEXT of NEXT america NEXT concern* NEXT index* OR efa OR efaci OR "glasgow epilepsy outcome" NEXT scale* OR geos* OR "hague restrictions in childhood epilepsy" NEXT scale* OR harces* OR cheqol* OR "modified impact of epilepsy" NEXT schedule* OR mioes OR "impact of childhood illness" NEXT scale* OR ici* OR "impact of Paediatric epilepsy" NEXT scale* OR "impact of paediatric epilepsy" NEXT scale* OR ipes* OR "impact of childhood neurologic" NEXT disability* OR icnd* OR neuroqol* OR newcastle NEXT mitochondrial NEXT disease* OR nmds* OR "newcastle adult mitochondrial" NEXT disease* OR nmdas* OR nmdsa* OR "newcastle Paediatric mitochondrial" NEXT disease* OR "newcastle paediatric mitochondrial" NEXT disease* OR npmds OR pedsql* OR peds-ql* OR pedsqlem* OR peds-qlem* OR "Paediatric epilepsy side effects" NEXT questionnaire* OR "paediatric epilepsy side effects" NEXT questionnaire* OR pesq* OR "patient-reported outcomes measurement information" NEXT system* OR promis OR promis25 OR promis37 OR promis49 OR qolce* OR gqolce* OR qolie* OR qolpes* 6948

#112 #110 OR #111 154295

#113 #80 AND #100 242

#114 #80 AND #112 409

#115 #113 OR #114 465

#116 #115 in Trials 225

1. **Source: HTA Database**

Interface / URL: https://database.inahta.org/

Database coverage dates: Information not found. The former database was produced by the CRD until March 2018, at which time the addition of records was stopped as INAHTA was in the process of rebuilding the new database platform. In July 2019, the database records were exported from the CRD platform and imported into the new platform that was developed by INAHTA. The rebuild of the new platform was launched in June 2020.

Search date: 26/07/2022

Retrieved records: 27

Search strategy:

54 #53 OR #52 OR #51 OR #50 OR #49 OR #48 OR #47 OR #46 OR #45 OR #44 OR #43 OR #42 OR #41 OR #40 OR #39 OR #38 OR #37 OR #36 OR #35 OR #34 OR #33 OR #32 OR #31 OR #30 OR #29 OR #28 OR #27 OR #26 OR #25 OR #24 OR #23 OR #22 OR #21 OR #20 OR #19 OR #18 OR #17 OR #16 OR #15 OR #14 OR #13 OR #12 OR #11 OR #10 OR #9 OR #8 OR #7 OR #6 OR #5 OR #4 OR #3 OR #2 OR #1 27

53 (ataxia OR ataxic) AND ("lactic acidosis" OR pyruvate OR decarboxylase) 0

52 (pdhc OR pdh OR "pyruvate decarboxylase") AND deficien* 0

51 ("pyruvate dehydrogenase" AND deficien*) 0

50 "Pyruvate Dehydrogenase Complex Deficiency Disease"[mh] 0

49 (pchsix OR "pch-six" OR pchvi OR "pch-vi") 0

48 (pch6 OR "pch-6") 0

47 (pontocerebellum AND hypoplasia*) 0

46 (pontocerebellar AND hypoplasia*) 0

45 "Olivopontocerebellar Atrophies"[mh] 0

44 (rarstwo OR "rars-two" OR rarsii OR "rars-ii" OR rars11 OR "rars-11") 0

43 (rars2 OR "rars-2") 0

42 "Arginine-tRNA Ligase"[mh] 0

41 epilep* AND "ragged red" 0

40 fukuhara* 0

39 merff 0

38 merrf 0

37 "MERRF Syndrome"[mh] 0

36 myoencephalopath* 0

35 melas 0

34 "MELAS Syndrome"[mh] 0

33 sando 0

32 miras 1

31 ataxic AND neuropath* 0

30 ataxia AND neuropath* 4

29 graefe* 0

28 (peo OR cpeo* OR arpeo OR adpeo) 1

27 "ocular muscular" 0

26 ophthalmoplegi* 2

25 "Ophthalmoplegia, Chronic Progressive External"[mh] 0

24 scae 0

23 spinocerebellar AND ataxia* AND epilep* 1

22 memsa 1

21 myoclon* AND epilep* AND ataxia* 3

20 "Spinocerebellar Ataxias"[mh] AND "Epilepsy"[mhe] 0

19 mchs 1

18 myocerebrohepatopath* 1

17 ahs OR mdas 1

16 ("diffuse cerebral" OR "encephalitis periaxialis" OR "myelinoclastic diffuse" OR "poliodystrophia cerebri" OR "progressive neuronal" OR "sudanophilic cerebral") 0

15 poliodystroph* 0

14 balo* AND concentric 0

13 schilder* 4

12 alper* 1

11 "Diffuse Cerebral Sclerosis of Schilder"[mh] 0

10 (polgone OR polgi) 0

9 (polg OR polg1) 1

8 (polymerase* AND gamma) 1

7 "DNA Polymerase gamma"[mh] 0

6 (subacute OR "sub-acute") AND encephal* 0

5 leigh* 5

4 "Leigh Disease"[mh] 0

3 mitochondri* 11

2 "Mitochondrial Myopathies"[mh] OR "Mitochondrial Encephalomyopathies"[mh] 1

1 "Mitochondrial Diseases"[mh] 1

1. **Source: Cost-Effectiveness Analysis (CEA) Registry**

Interface / URL: https://cevr.tuftsmedicalcenter.org/databases/cea-registry

Database coverage dates: The webpage at the URL states that the database includes studies published from 1976 to the present

Search date: 26/07/2022

Retrieved records: 0

Search strategy:

The advanced search interface at the following URL was used: <https://cear.tuftsmedicalcenter.org/>

The following searches were conducted separately. Search settings were left at default ('Methods' was left selected, drop-down search options were left as 'Keyword' 'Is'). Where AND or OR is shown below, this indicates that Boolean AND / OR was selected using the interface '+AND' / '+OR' options.

Returned results were screened by the information specialist for potential relevance to the populations of interest. Potentially relevant results were cross-checked against results from other search sources to see if already retrieved. Any potentially relevant results not identified via another source were retrieved for further assessment.

mitochondrial OR mitochondria OR mitochondrias OR mitochondriopathy OR mitochondriopathies OR mitochondriopathic OR mdas = 0 (1 result returned, excluded)

leigh OR leighs OR leigh's = 0 (3 results returned, excluded)

necrotizing OR necrotising OR necrotic = 0 (6 results returned, excluded)

polymerase AND gamma = 0 (0 results returned)

polymerases OR polg OR polg1 OR polgone OR polgi = 0 (0 results returned)

alper OR alpers OR alper's = 0 (0 results returned)

schilder OR schilders OR schilder's = 0 (0 results returned)

concentric OR poliodystrophy OR poliodystrophies OR poliodystrophia OR poliodystrophias OR poliodystrophic = 0 (0 results returned)

diffuse AND cerebral = 0 (1 result returned, excluded)

encephalitis AND periaxialis = 0 (0 results returned)

myelinoclastic OR sudanophilic = 0 (0 results returned)

progressive AND neuronal = 0 (0 results returned)

ahs = 0 (1 result returned, excluded)

myocerebrohepatopathy OR myocerebrohepatopathies OR myocerebrohepatopathic OR mchs = 0 (0 results returned)

sensory OR memsa OR spinocerebellar OR scae OR ophthalmoplegia OR ophthalmoplegias OR ophthalmoplegic = 0 (3 results returned, excluded)

ocular AND muscular = 0 (0 results returned)

peo OR cpeo OR arpeo OR adpeo = 0 (0 results returned)

graefe OR graefes OR graefe's = 0 (0 results returned)

ataxia AND neuropathy = 0 (0 results returned)

ataxia AND neuropathies = 0 (0 results returned)

ataxia AND neuropathic = 0 (0 results returned)

ataxic = 0 (0 results returned)

miras OR sando OR melas = 0 (1 result returned, excluded)

myoencephalopathy OR myoencephalopathies OR myoencephalopathic = 0 (0 results returned)

merrf OR merff = 0 (0 results returned)

fukuhara OR fukuharas OR fukuhara's = 0 (0 results returned)

ragged = 0 (0 results returned)

rars2 OR rars OR rarstwo OR rarsii OR rars11 = 0 (0 results returned)

pontocerebellar OR pontocerebellum = 0 (0 results returned)

pch6 OR pch OR pchsix OR pchvi OR pyruvate OR pdhc OR pdh OR decarboxylase = 0 (2 results returned, excluded)

lactic AND acidosis = 0 (0 results returned)

Search note: The CEA Registry search interface had been updated since the date of the original searches. As a result, the search terms used for this search differ from the original search.

1. **Source: NHS Economic Evaluation Database (NHS EED)**

Interface / URL: https://www.crd.york.ac.uk/CRDWeb

Database coverage dates: Information not found. Bibliographic records were published on NHS EED until 31st March 2015. Searches of MEDLINE, Embase, CINAHL, PsycINFO and PubMed were continued until the end of the 2014.

Search date: na – no search conducted – see below

Retrieved records: na

Search strategy: na

Search note:

Although it can still be accessed, NHS EED is a closed database. Records were last added to NHS EED in 2015. This means that no new records will have been added since the date of the original search (13/08/2021). No search of NHS EED was therefore required for this update.

1. **Source: Econlit**

Interface / URL: OvidSP

Database coverage dates: 1886 to July 14, 2022

Search date: 26/07/2022

Retrieved records: 99

Search strategy:

1 (mitochondri$ adj6 (disease$ or disorder$ or syndrome$)).af. (0)

2 (mitochondri$ adj6 (cytopath$ or deficien$ or dysfunction$ or encephalomyopath$ or encephalopath$ or myopath$)).af. (0)

3 (mitochondriopath$ or (mdas not modified dental anxiety scale)).af. (13)

4 (mitochondri$ adj6 (epilep$ or seizure$ or convuls$)).af. (0)

5 or/1-4 (13)

6 ((leigh$ or leigh-feigin$ or leigh-feigin-wolf$) adj6 (disease$ or disorder$ or syndrome$)).af. (0)

7 ((leigh$ or leigh-feigin$ or leigh-feigin-wolf$) adj6 (encephalopath$ or encephalomyelopath$)).af. (0)

8 ((subacute or sub-acute) adj3 (necrot$ encephal$ or necrot$ juvenile encephal$ or necrot$ infantile encephal$)).af. (0)

9 (leigh$ and mitochondri$).af. (0)

10 or/6-9 (0)

11 (polymerase$ adj3 gamma).af. (0)

12 (polg or polg1).af. (0)

13 (polgone or polgi).af. (0)

14 (mitochondri$ adj6 polymerase$).af. (0)

15 or/11-14 (0)

16 ((alper$ or alper-huttenlocher$ or alpers-huttenlocher$) adj6 (disease$ or disorder$ or syndrome$)).af. (1)

17 ((alper$ or alper-huttenlocher$ or alpers-huttenlocher$) adj6 (diffuse cerebral or diffuse degeneration or poliodystroph$)).af. (0)

18 (schilder$ adj6 (disease$ or disorder$ or syndrome$)).af. (0)

19 (schilder$ adj6 (diffuse cerebral or diffuse degeneration or poliodystroph$)).af. (0)

20 (balo$ adj3 concentric scleros$).af. (0)

21 (progressive adj3 poliodystroph$).af. (0)

22 (diffuse cerebral degeneration$ or diffuse cerebral scleros$ or encephalitis periaxialis or myelinoclastic diffuse scleros$ or poliodystrophia cerebri or progressive neuronal degeneration$ or sudanophilic cerebral scleros$).af. (0)

23 ahs.af. (53)

24 or/16-23 (54)

25 myocerebrohepatopath$.af. (0)

26 mchs.af. (0)

27 25 or 26 (0)

28 (myoclon$ and epilep$ and sensory ataxia$).af. (0)

29 memsa.af. (0)

30 (spinocerebellar ataxia$ and epilep$).af. (0)

31 scae.af. (0)

32 or/28-31 (0)

33 external ophthalmoplegi$.af. (0)

34 ocular muscular dystroph$.af. (0)

35 (peo or cpeo$ or arpeo or adpeo).af. (9)

36 ((graefe$ or graefe fuch$) adj6 (disease$ or disorder$ or syndrome$)).af. (0)

37 ((graefe$ or graefe fuch$) adj6 myopath$).af. (0)

38 or/33-37 (9)

39 ataxia neuropath$.af. (0)

40 ataxic neuropath$.af. (0)

41 miras.af. (10)

42 sando.af. (0)

43 or/39-42 (10)

44 melas.af. (3)

45 myoencephalopath$.af. (0)

46 44 or 45 (3)

47 merrf.af. (0)

48 merff.af. (0)

49 fukuhara$.af. (10)

50 (epilep$ and ragged red).af. (0)

51 or/47-50 (10)

52 (mitochondri$ and arg$ and (tRNA or RNA or ribonucleic acid)).af. (0)

53 (rars2 or rars-2).af. (0)

54 (rarstwo or rars-two or rarsii or rars-ii or rars11 or rars-11).af. (0)

55 or/52-54 (0)

56 (pontocerebellar adj6 hypoplasia$).af. (0)

57 (pontocerebellum adj6 hypoplasia$).af. (0)

58 (pch6 or pch-6).af. (0)

59 (pchsix or pch-six or pchvi or pch-vi).af. (0)

60 or/56-59 (0)

61 (pyruvate dehydrogenase adj6 deficien$).af. (0)

62 ((pdhc or pdh or pyruvate decarboxylase) adj6 deficien$).af. (0)

63 ((ataxia or ataxic) adj6 (lactic acidosis or pyruvate or decarboxylase)).af. (0)

64 or/61-63 (0)

65 5 or 10 or 15 or 24 or 27 or 32 or 38 or 43 or 46 or 51 or 55 or 60 or 64 (99)

1. **Source: Paediatric Economic Database Evaluation**

Interface / URL: http://pede.ccb.sickkids.ca/pede/

Database coverage dates: The information at the following URL states that the database contains records for studies published from January 1, 1980 to December 31, 2020 - http://pede.ccb.sickkids.ca/pede/database.jsp

Search date: 26/07/2022

Retrieved records: 0

Search strategy:

The basic search interface at the following URL was used: http://pede.ccb.sickkids.ca/pede/search.jsp. All search settings were left as default. In the default setting, terms are searched across the 'Title, abstract, or Keywords'.

The basic search interface allows the searcher to enter up to three terms combined with AND using the separate search line functionality built into the interface. Where AND is shown between terms in the syntax below, this indicates that this functionality was used. All other terms were searched for individually and not combined with any other term.

Separate searches were conducted on each of the following terms. Returned results were screened by the information specialist for potential relevance to the populations of interest. Potentially relevant results were cross-checked against results from other search sources to see if already retrieved. Any potentially relevant results not identified via another source were retrieved for further assessment.

mitochondri = 0 (0 results returned)

mdas = 0 (0 results returned)

leigh = 0 (0 results returned)

subacute = 0 (0 results returned)

sub-acute = 0 (0 results returned)

polymerase AND gamma = 0 (0 results returned)

polg = 0 (0 results returned)

alper = 0 (0 results returned)

schilder = 0 (0 results returned)

concentric = 0 (0 results returned)

poliodystroph = 0 (0 results returned)

diffuse cerebral = 0 (0 results returned)

encephalitis periaxialis = 0 (0 results returned)

myelinoclastic diffuse = 0 (0 results returned)

progressive neuronal = 0 (0 results returned)

sudanophilic cerebral = 0 (0 results returned)

ahs = 0 (2 results returned, excluded)

myocerebrohepatopath = 0 (0 results returned)

mchs = 0 (0 results returned)

sensory ataxia = 0 (0 results returned)

memsa = 0 (0 results returned)

spinocerebellar ataxia = 0 (0 results returned)

scae = 0 (0 results returned)

external ophthalmoplegi = 0 (0 results returned)

ocular muscular dystroph = 0 (0 results returned)

peo = 0 (102 results returned, excluded)

cpeo = 0 (0 results returned)

arpeo = 0 (0 results returned)

adpeo = 0 (0 results returned)

graefe = 0 (0 results returned)

ataxia neuropath = 0 (0 results returned)

ataxic neuropath = 0 (0 results returned)

miras = 0 (0 result returned)

sando = 0 (1 result returned, excluded)

melas = 0 (0 results returned)

myoencephalopath = 0 (0 results returned)

merrf = 0 (0 results returned)

merff = 0 (0 results returned)

fukuhara = 0 (0 results returned)

ragged red = 0 (0 results returned)

rars = 0 (0 results returned)

pontocerebellar = 0 (0 results returned)

pontocerebellum = 0 (0 results returned)

pch = 0 (1 result returned, excluded)

pdh = 0 (0 results returned)

pyruvate = 0 (0 results returned)

lactic acidosis = 0 (0 results returned)

decarboxylase = 0 (0 results returned)

1. **Source: APA PsycInfo**

Interface / URL: OvidSP

Database coverage dates: 1806 to July Week 3 2022

Search date: 26/07/2022

Retrieved records: 105

Search strategy:

1 mitochondria/ (4312)

2 (mitochondri$ adj6 (disease$ or disorder$ or syndrome$)).ti,ab,hw,id. (2055)

3 (mitochondri$ adj6 (cytopath$ or deficien$ or dysfunction$ or encephalomyopath$ or encephalopath$ or myopath$)).ti,ab,hw,id. (2960)

4 (mitochondriopath$ or (mdas not modified dental anxiety scale)).ti,ab,hw,id. (124)

5 (mitochondri$ adj6 (epilep$ or seizure$ or convuls$)).ti,ab,hw,id. (130)

6 or/1-5 (5994)

7 ((leigh$ or leigh-feigin$ or leigh-feigin-wolf$) adj6 (disease$ or disorder$ or syndrome$)).ti,ab,hw,id. (174)

8 ((leigh$ or leigh-feigin$ or leigh-feigin-wolf$) adj6 (encephalopath$ or encephalomyelopath$)).ti,ab,hw,id. (22)

9 ((subacute or sub-acute) adj3 (necrot$ encephal$ or necrot$ juvenile encephal$ or necrot$ infantile encephal$)).ti,ab,hw,id. (9)

10 (leigh$ and mitochondri$).ti,ab,hw,id. (144)

11 or/7-10 (182)

12 (polymerase$ adj3 gamma).ti,ab,hw,id. (71)

13 (polg or polg1).ti,ab,hw,id. (140)

14 (polgone or polgi).ti,ab,hw,id. (1)

15 (mitochondri$ adj6 polymerase$).ti,ab,hw,id. (67)

16 or/12-15 (181)

17 ((alper$ or alper-huttenlocher$ or alpers-huttenlocher$) adj6 (disease$ or disorder$ or syndrome$)).ti,ab,hw,id. (27)

18 ((alper$ or alper-huttenlocher$ or alpers-huttenlocher$) adj6 (diffuse cerebral or diffuse degeneration or poliodystroph$)).ti,ab,hw,id. (1)

19 (schilder$ adj6 (disease$ or disorder$ or syndrome$)).ti,ab,hw,id. (16)

20 (schilder$ adj6 (diffuse cerebral or diffuse degeneration or poliodystroph$)).ti,ab,hw,id. (0)

21 (balo$ adj3 concentric scleros$).ti,ab,hw,id. (22)

22 (progressive adj3 poliodystroph$).ti,ab,hw,id. (1)

23 (diffuse cerebral degeneration$ or diffuse cerebral scleros$ or encephalitis periaxialis or myelinoclastic diffuse scleros$ or poliodystrophia cerebri or progressive neuronal degeneration$ or sudanophilic cerebral scleros$).ti,ab,hw,id. (33)

24 ahs.ti,ab,hw,id. (304)

25 or/17-24 (396)

26 myocerebrohepatopath$.ti,ab,hw,id. (1)

27 mchs.ti,ab,hw,id. (15)

28 26 or 27 (15)

29 (myoclon$ and epilep$ and sensory ataxia$).ti,ab,hw,id. (1)

30 memsa.ti,ab,hw,id. (4)

31 (spinocerebellar ataxia$ and epilep$).ti,ab,hw,id. (36)

32 scae.ti,ab,hw,id. (5)

33 or/29-32 (41)

34 external ophthalmoplegi$.ti,ab,hw,id. (128)

35 ocular muscular dystroph$.ti,ab,hw,id. (0)

36 (peo or cpeo$ or arpeo or adpeo).ti,ab,hw,id. (120)

37 ((graefe$ or graefe fuch$) adj6 (disease$ or disorder$ or syndrome$)).ti,ab,hw,id. (0)

38 ((graefe$ or graefe fuch$) adj6 myopath$).ti,ab,hw,id. (1)

39 or/34-38 (198)

40 ataxia neuropath$.ti,ab,hw,id. (29)

41 ataxic neuropath$.ti,ab,hw,id. (35)

42 miras.ti,ab,hw,id. (53)

43 sando.ti,ab,hw,id. (15)

44 or/40-43 (121)

45 melas/ (146)

46 melas.ti,ab,hw,id. (257)

47 myoencephalopath$.ti,ab,hw,id. (1)

48 or/45-47 (258)

49 merrf.ti,ab,hw,id. (47)

50 merff.ti,ab,hw,id. (2)

51 fukuhara$.ti,ab,hw,id. (7)

52 (epilep$ and ragged red).ti,ab,hw,id. (37)

53 or/49-52 (67)

54 (mitochondri$ and arg$ and (tRNA or RNA or ribonucleic acid)).ti,ab,hw,id. (9)

55 (rars2 or rars-2).ti,ab,hw,id. (7)

56 (rarstwo or rars-two or rarsii or rars-ii or rars11 or rars-11).ti,ab,hw,id. (1)

57 or/54-56 (14)

58 ((pontocerebellar adj6 hypoplasia$) or (pontocerebellum adj6 hypoplasia$)).ti,ab,hw,id. (50)

59 (pch6 or pch-6).ti,ab,hw,id. (3)

60 (pchsix or pch-six or pchvi or pch-vi).ti,ab,hw,id. (0)

61 or/58-60 (51)

62 (pyruvate dehydrogenase adj6 deficien$).ti,ab,hw,id. (28)

63 ((pdhc or pdh or pyruvate decarboxylase) adj6 deficien$).ti,ab,hw,id. (9)

64 ((ataxia or ataxic) adj6 (lactic acidosis or pyruvate or decarboxylase)).ti,ab,hw,id. (16)

65 or/62-64 (45)

66 6 or 11 or 16 or 25 or 28 or 33 or 39 or 44 or 48 or 53 or 57 or 61 or 65 (6833)

67 exp "Quality of Life"/ (48808)

68 (quality adjusted or adjusted life year$).ti,ab,hw,id. (2178)

69 (qaly$ or qald$ or qale$ or qtime$).ti,ab,hw,id. (1397)

70 (illness state$1 or health state$1).ti,ab,hw,id. (1838)

71 (hui or hui1 or hui2 or hui3).ti,ab,hw,id. (641)

72 (multiattribute$ or multi attribute$).ti,ab,hw,id. (1245)

73 (utility adj3 (score$1 or valu$ or health$ or cost$ or measur$ or disease$ or mean or gain or gains or index$)).ti,ab,hw,id. (5156)

74 utilities.ti,ab,hw,id. (2259)

75 (eq-5d or eq5d or eq-5 or eq5 or euro qual or euroqual or euro qual5d or euroqual5d or euro qol or euroqol or euro qol5d or euroqol5d or euro quol or euroquol or euro quol5d or euroquol5d or eur qol or eurqol or eur qol5d or eur qol5d or eur?qul or eur?qul5d or euro$ quality of life or european qol).ti,ab,hw,id. (2840)

76 (euro$ adj3 (5 d or 5d or 5 dimension$ or 5dimension$ or 5 domain$ or 5domain$)).ti,ab,hw,id. (795)

77 (sf36$ or sf 36$ or sf thirtysix or sf thirty six).ti,ab,hw,id. (5103)

78 (time trade off$1 or time tradeoff$1 or tto or timetradeoff$1).ti,ab,hw,id. (475)

79 ("Costs and Cost Analysis"/ or exp Health Care Costs/) and (cost-effectiveness ratio$ and perspective$ or life expectanc$)).ti,ab,hw,id. (318)

80 (economics/ or health care economics/ or pharmacoeconomics/ or "costs and cost analysis"/) and models/ (1147)

81 or/67-80 (62235)

82 (utility loss$ or disutility$).ti,ab,hw,id. (272)

83 (ahum or aqol$ or chu9d or chu-9d or eq-5dy or eq5dy or health status classification system$ or hscs-ps or hscsps or hui or hui1 or hui2 or hui3 or quality of well-being or quality of wellbeing or qwb or 15d or 15-d or 15 dimension$ or 16d or 16-d or 16 dimension$ or 17d or 17-d or 17 dimension$).ti,ab,hw,id. (1539)

84 or/81-83 (62947)

85 ((quality adj3 life) or qol).ti,ab,hw,id. (87269)

86 (hql or hqol or hrqol or hrql or hr-ql).ti,ab,hw,id. (6704)

87 (short form$ or shortform$).ti,ab,hw,id. (15705)

88 (sf6 or sf-6 or sf6d or sf-6d or sf-six or sfsix).ti,ab,hw,id. (355)

89 (sf8 or sf-8 or sf-eight or sfeight).ti,ab,hw,id. (143)

90 (sf12 or sf-12 or sf-twelve or sftwelve).ti,ab,hw,id. (1386)

91 (sf16 or sf-16 or sf-sixteen or sfsixteen).ti,ab,hw,id. (3)

92 (sf20 or sf-20 or sf-twenty or sftwenty).ti,ab,hw,id. (48)

93 or/85-92 (99185)

94 (attitudes to children with epilepsy$ or aatcwe$ or disabkids$ or eldqol$ or "epilepsy and children questionnaire$" or ecq$ or "epilepsy foundation$ of america concern$ index$" or efa or efaci or glasgow epilepsy outcome scale$ or geos$ or hague restrictions in childhood epilepsy scale$ or harces$ or cheqol$ or modified impact of epilepsy schedule$ or mioes or impact of childhood illness scale$ or ici$ or impact of pediatric epilepsy scale$ or impact of paediatric epilepsy scale$ or ipes$ or impact of childhood neurologic disability$ or icnd$ or neuroqol$ or newcastle mitochondrial disease$ or nmds$ or newcastle adult mitochondrial disease$ or nmdas$ or nmdsa$ or newcastle pediatric mitochondrial disease$ or newcastle paediatric mitochondrial disease$ or npmds or pedsql$ or peds-ql$ or pedsqlem$ or peds-qlem$ or pediatric epilepsy side effects questionnaire$ or paediatric epilepsy side effects questionnaire$ or pesq$ or patient-reported outcomes measurement information system$ or promis or promis25 or promis37 or promis49 or qolce$ or gqolce$ or qolie$ or qolpes$).ti,ab,hw,id. (9516)

95 "Quality of Life Measures"/ (752)

96 or/93-95 (106736)

97 66 and 84 (23)

98 66 and 96 (100)

99 97 or 98 (105)

1. **Source: ScHARRHud**

Interface / URL: http://www.scharrhud.org/

Database coverage dates: Information not found

Search date: 26/07/2022

Retrieved records: 0

Search strategy:

The basic search interface at the following URL was used: https://www.scharrhud.org/index.php?recordsN1&m=search. All search settings were left as default. In the default setting, terms are searched across 'Any field'.

Separate searches were conducted on each of the following terms. Returned results were screened by the information specialist for potential relevance to the populations of interest. Potentially relevant results were cross-checked against results from other search sources to see if already retrieved. Any potentially relevant results not identified via another source were retrieved for further assessment.

mitochondri* = 0 (0 results returned)

mdas = 0 (0 results returned)

leigh* OR encephal* = 0 (2 results returned, excluded)

polymerase* AND gamma = 0 (0 results returned)

polg* = 0 (1 result returned, excluded)

alper* = 0 (0 results returned)

schilder* = 0 (0 results returned)

concentric scleros* OR poliodystroph* OR diffuse cerebral OR encephalitis periaxialis OR myelinoclastic diffuse OR progressive neuronal OR sudanophilic cerebral = 0 (0 results returned)

ahs = 0 (0 results returned)

myocerebrohepatopath* OR mchs = 0 (0 results returned)

myoclon* AND epilep* AND sensory ataxia* = 0 (0 results returned)

memsa = 0 (1 result returned, excluded)

spinocerebellar ataxia* = 0 (0 results returned)

scae = 0 (0 results returned)

external ophthalmoplegi* = 0 (0 results returned)

ocular muscular dystroph* = 0 (0 results returned)

peo OR cpeo* OR arpeo OR adpeo OR graefe* OR ataxia neuropath* OR ataxic neuropath* OR miras OR sando OR melas OR myoencephalopath* OR merrf OR merff OR fukuhara* OR ragged red = 0 (0 results returned)

rars* = 0 (0 results returned)

pontocerebellar OR pontocerebellum OR pch* = 0 (0 results returned)

pdhc OR pdh OR decarboxylase OR lactic acidosis OR pyruvate = 0 (1 result returned, excluded)

1. **Source: National Institute for Health and Care Excellence (NICE) webpages**

Interface / URL: https://www.nice.org.uk/

Database coverage dates: n/a

Search date: 26/07/2022

Retrieved records: 0

Search strategy:

The following documents were sought: Company Submissions, Final Appraisal Determination Documents, Assessment Reports for multiple technology appraisals, Evidence Review Group (ERG) reports for single technology appraisals – published or updated since the date of the original search (13/08/2021).

The site-wide search interface was used at: https://www.nice.org.uk/. Separate searches were conducted on each term shown below.

Results were filtered by 'Document Type' to 'Guidance', then by 'Guidance Programme' to 'Technology appraisal guidance' (if these were available as options).

Results were scanned to identify published technology appraisals on the eligible mitochondrial diseases. For any identified, associated documents under the 'History' tab were viewed to check for relevant documents. PDFs for relevant documents were retrieved for further assessment. Duplicate documents were not retrieved.

mitochondri* = 0 (1 technology appraisal result returned, excluded)

mdas = 0 (0 results returned)

leigh* = 0 (0 technology appraisal results returned)

subacute = 0 (1 technology appraisal result returned, excluded)

"sub-acute" = 0 (1 technology appraisal result returned, excluded)

polymerase* AND gamma = 0 (0 technology appraisal results returned)

polg* = 0 (0 results returned)

alper* OR schilder* OR concentric OR poliodystroph* = (0 technology appraisal results returned)

"diffuse cerebral" OR "encephalitis periaxialis" OR "myelinoclastic diffuse" OR "progressive neuronal" OR "sudanophilic cerebral" = 0 (0 results returned)

ahs = 0 (0 technology appraisal results returned)

myocerebrohepatopath* OR mchs = 0 (0 results returned)

myoclon* AND epilep* AND ataxia* = 0 (0 results returned)

memsa = 0 (0 results returned)

spinocerebellar AND epilep* = 0 (0 results returned)

scae = 0 (0 results returned)

external AND ophthalmoplegi* = 0 (0 results returned)

"ocular muscular" = 0 (0 results returned)

peo OR cpeo* OR arpeo OR adpeo = 0 (0 results returned)

graefe* OR ataxia OR ataxic OR miras OR sando OR melas OR myoencephalopath* OR merrf OR merff OR fukuhara* OR "ragged red" OR rars* OR pontocerebellar OR pontocerebellum OR pch* OR "pyruvate dehydrogenase" OR pdhc OR pdh OR decarboxylase = 0 (0 technology appraisal results returned)

0 documents were retrieved

1. **Source: Institute for Clinical and Economic Review webpages**

Interface / URL: https://icer-review.org/

Database coverage dates: n/a

Search date: 25/07/2022

Retrieved records: 0

Search strategy:

The following were sought: Final Evidence Report (or Draft Evidence Report if Final was not available) and the Evidence Presentation (if available) – published since the date of the previous search (23/08/2021).

Assessments were located at the following URL: <https://icer.org/explore-our-research/assessments/>.

The drop-down options were used to view all documents with status 'completed' and research type 'assessment'. 92 results were returned.

The descriptive text was checked for relevance to the eligible mitochondrial diseases. No documents were retrieved for further assessment.

1. **Source: Canadian Agency for Drugs and Technologies in Health (CADTH) webpages**

Interface / URL: https://www.cadth.ca/

Database coverage dates: n/a

Search date: 26/07/2022

Retrieved records: 0

Search strategy:

The following documents were sought: economic guidance and final recommendations associated with reimbursement reviews – published or updated from the date of the previous search (23/08/2021).

The site-wide search interface was used at: <https://www.cadth.ca/search?s>=. 'Advanced search was selected'.

Results were filtered by 'Project Line' to 'Reimbursement Review'.

Results were assessed for relevance to the eligible mitochondrial diseases. Relevant results were checked for document types of interest.

PDFs of relevant documents were retrieved for further assessment. Duplicate documents were not retrieved.

mitochondrial = 0 (7 results returned, excluded)

mitochondria = 0 (3 results returned, excluded)

mitochondrias = 0 (3 results returned, excluded)

mitochondriopathy = 0 (0 results returned)

mitochondriopathies = 0 (0 results returned)

mitochondriopathic = 0 (0 results returned)

mdas = 0 (0 results returned)

leigh = 0 (11 results returned, excluded)

leighs = 0 (11 results returned, excluded)

leigh's = 0 (11 results returned, excluded)

necrotizing = 0 (7 results returned, excluded)

necrotising = 0 (7 results returned, excluded)

necrotic = 0 (6 results returned, excluded)

polymerase = 0 (49 results returned, excluded)

polymerases = 0 (49 results returned, excluded)

polg = 0 (0 results returned)

polg1 = 0 (0 results returned

polgone = 0 (0 results returned

polgi = 0 (0 results returned)

alper = 0 (1 result returned, excluded)

alpers = 0 (1 result returned, excluded)

alper's = 0 (1 result returned, excluded)

schilder = 0 (0 results returned)

schilders = 0 (0 results returned)

schilder's = 0 (0 results returned)

balo = 0 (0 results returned)

balos = 0 (0 results returned)

balo's = 0 (0 results returned)

poliodystrophy = 0 (0 results returned)

poliodystrophies = 0 (0 results returned)

poliodystrophia = 0 (0 results returned)

poliodystrophias = 0 (0 results returned)

poliodystrophic = 0 (0 results returned)

diffuse AND cerebral = 0 (8 results returned, excluded)

periaxialis = 0 (0 results returned)

myelinoclastic = 0 (0 results returned)

sudanophilic = 0 (0 results returned)

neuronal = 0 (31 results returned, excluded)

ahs = 0 (93 results returned, excluded)

myocerebrohepatopathy = 0 (0 results returned)

myocerebrohepatopathies = 0 (0 results returned)

myocerebrohepatopathic = 0 (0 results returned)

mchs = 0 (0 results returned)

ataxia = 0 (14 results returned, excluded)

ataxias = 0 (14 results returned, excluded)

memsa = 0 (0 results returned)

scae = 0 (0 results returned)

ophthalmoplegia = 0 (0 results returned)

ophthalmoplegias = 0 (0 results returned)

ophthalmoplegic = 0 (2 results returned, excluded)

ocular AND muscular = 0 (7 results returned, excluded)

peo = 0 (12 results returned, excluded)

cpeo = 0 (0 results returned)

arpeo = 0 (0 results returned)

adpeo = 0 (0 results returned)

graefe = 0 (8 results returned, excluded)

graefes = 0 (8 results returned, excluded)

graefe's = 0 (8 results returned, excluded)

ataxic = 0 (1 result returned, excluded)

miras = 0 (0 results returned)

sando = 0 (1 result returned, excluded)

melas = 0 (0 results returned)

myoencephalopathy = 0 (0 results returned)

myoencephalopathies = 0 (0 results returned)

myoencephalopathic = 0 (0 results returned)

merrf = 0 (0 results returned)

merff = 0 (0 results returned)

fukuhara = 0 (0 results returned)

fukuharas = 0 (0 results returned)

fukuhara's = 0 (0 results returned)

ragged = 0 (3 results returned, excluded)

rars2 = 0 (0 results returned)

rars = 0 (2 results returned, excluded)

rarstwo = 0 (0 results returned)

rarsii = 0 (0 results returned)

rars11 = 0 (0 results returned)

pontocerebellar = 0 (0 results returned)

pontocerebellum = 0 (0 results returned)

pch6 = 0 (0 results returned)

pch = 0 (8 results returned, excluded)

pchsix = 0 (0 results returned)

pchvi = 0 (0 results returned)

pyruvate = 0 (2 results returned, excluded)

pdhc = 0 (0 results returned)

pdh = 0 (1 result returned, excluded)

decarboxylase = 0 (7 results returned, excluded)

lactic AND acidosis = 0 (23 results returned, excluded)

Search note: The CADTH search interface had been updated since the date of the original searches. As a result, the search terms used for this search differ from the original search.

### Costs / Healthcare Resource Use Full Search Strategies

#### Original searches: August 2021

1. **Source: MEDLINE ALL**

Interface / URL: OvidSP

Database coverage dates: 1946 to August 10, 2021

Search date: 11/08/2021

Retrieved records: 1538

Search strategy:

1 mitochondrial diseases/ (5734)

2 mitochondrial myopathies/ or mitochondrial encephalomyopathies/ (2704)

3 (mitochondri$ adj6 (disease$ or disorder$ or syndrome$)).ti,ab,kf. (17759)

4 (mitochondri$ adj6 (cytopath$ or deficien$ or dysfunction$ or encephalomyopath$ or encephalopath$ or myopath$)).ti,ab,kf. (36686)

5 mitochondriopath$.ti,ab,kf. (347)

6 (mitochondri$ adj6 (epilep$ or seizure$ or convuls$)).ti,ab,kf. (560)

7 or/1-6 (48941)

8 Leigh Disease/ (1123)

9 ((leigh$ or leigh-feigin$ or leigh-feigin-wolf$) adj6 (disease$ or disorder$ or syndrome$)).ti,ab,kf. (1624)

10 ((leigh$ or leigh-feigin$ or leigh-feigin-wolf$) adj6 (encephalopath$ or encephalomyelopath$)).ti,ab,kf. (304)

11 ((subacute or sub-acute) adj3 (necrot$ encephal$ or necrot$ juvenile encephal$ or necrot$ infantile encephal$)).ti,ab,kf. (262)

12 (leigh$ and mitochondri$).ti,ab,kf. (1159)

13 or/8-12 (1984)

14 DNA Polymerase gamma/ (805)

15 (polymerase$ adj3 gamma).ti,ab,kf. (1310)

16 (polg or polg1).ti,ab,kf. (857)

17 (polgone or polgi).ti,ab,kf. (0)

18 (mitochondri$ adj6 polymerase$).ti,ab,kf. (1839)

19 or/14-18 (3109)

20 "Diffuse Cerebral Sclerosis of Schilder"/ (2366)

21 ((alper$ or alper-huttenlocher$ or alpers-huttenlocher$) adj6 (disease$ or disorder$ or syndrome$)).ti,ab,kf. (244)

22 ((alper$ or alper-huttenlocher$ or alpers-huttenlocher$) adj6 (diffuse cerebral or diffuse degeneration or poliodystroph$)).ti,ab,kf. (19)

23 (schilder$ adj6 (disease$ or disorder$ or syndrome$)).ti,ab,kf. (180)

24 (schilder$ adj6 (diffuse cerebral or diffuse degeneration or poliodystroph$)).ti,ab,kf. (5)

25 (balo$ adj3 concentric scleros$).ti,ab,kf. (185)

26 (progressive adj3 poliodystroph$).ti,ab,kf. (18)

27 (diffuse cerebral degeneration$ or diffuse cerebral scleros$ or encephalitis periaxialis or myelinoclastic diffuse scleros$ or poliodystrophia cerebri or progressive neuronal degeneration$ or sudanophilic cerebral scleros$).ti,ab,kf. (335)

28 ahs.ti,ab,kf. (1573)

29 or/20-28 (4258)

30 myocerebrohepatopath$.ti,ab,kf. (11)

31 mchs.ti,ab,kf. (94)

32 or/30-31 (98)

33 Spinocerebellar Ataxias/ and exp Epilepsy/ (67)

34 (myoclon$ and epilep$ and sensory ataxia$).ti,ab,kf. (10)

35 memsa.ti,ab,kf. (14)

36 (spinocerebellar ataxia$ and epilep$).ti,ab,kf. (164)

37 scae.ti,ab,kf. (55)

38 or/33-37 (271)

39 Ophthalmoplegia, Chronic Progressive External/ (583)

40 external ophthalmoplegi$.ti,ab,kf. (1667)

41 ocular muscular dystroph$.ti,ab,kf. (10)

42 (peo or cpeo$ or arpeo or adpeo).ti,ab,kf. (5887)

43 ((graefe$ or graefe fuch$) adj6 (disease$ or disorder$ or syndrome$)).ti,ab,kf. (11)

44 ((graefe$ or graefe fuch$) adj6 myopath$).ti,ab,kf. (2)

45 or/39-44 (7224)

46 ataxia neuropath$.ti,ab,kf. (128)

47 ataxic neuropath$.ti,ab,kf. (260)

48 miras.ti,ab,kf. (131)

49 sando.ti,ab,kf. (73)

50 or/46-49 (550)

51 MELAS Syndrome/ (1368)

52 melas.ti,ab,kf. (2140)

53 myoencephalopath$.ti,ab,kf. (10)

54 or/51-53 (2461)

55 MERRF Syndrome/ (384)

56 merrf.ti,ab,kf. (528)

57 merff.ti,ab,kf. (13)

58 fukuhara$.ti,ab,kf. (41)

59 (epilep$ and ragged red).ti,ab,kf. (498)

60 or/55-59 (824)

61 Arginine-tRNA Ligase/ (192)

62 (mitochondri$ and arg$ and (tRNA or RNA or ribonucleic acid)).ti,ab,kf. (554)

63 (rars2 or rars-2).ti,ab,kf. (51)

64 (rarstwo or rars-two or rarsii or rars-ii or rars11 or rars-11).ti,ab,kf. (5)

65 or/61-64 (764)

66 Olivopontocerebellar Atrophies/ or (pontocerebellar adj6 hypoplasia$).ti,ab,kf. (901)

67 (pontocerebellum adj6 hypoplasia$).ti,ab,kf. (0)

68 (pch6 or pch-6).ti,ab,kf. (35)

69 (pchsix or pch-six or pchvi or pch-vi).ti,ab,kf. (1)

70 or/66-69 (918)

71 Pyruvate Dehydrogenase Complex Deficiency Disease/ (406)

72 (pyruvate dehydrogenase adj6 deficien$).ti,ab,kf. (509)

73 ((pdhc or pdh or pyruvate decarboxylase) adj6 deficien$).ti,ab,kf. (244)

74 ((ataxia or ataxic) adj6 (lactic acidosis or pyruvate or decarboxylase)).ti,ab,kf. (113)

75 or/71-74 (811)

76 7 or 13 or 19 or 29 or 32 or 38 or 45 or 50 or 54 or 60 or 65 or 70 or 75 (66349)

77 Economics/ (27356)

78 exp "costs and cost analysis"/ (248056)

79 Economics, Dental/ (1919)

80 exp economics, hospital/ (25254)

81 Economics, Medical/ (9146)

82 Economics, Nursing/ (4005)

83 Economics, Pharmaceutical/ (3009)

84 (economic$ or cost or costs or costly or costing or price or prices or pricing or pharmacoeconomic$).ti,ab. (880356)

85 (expenditure$ not energy).ti,ab. (32380)

86 value for money.ti,ab. (1854)

87 budget$.ti,ab. (31485)

88 or/77-87 (1037117)

89 ((energy or oxygen) adj cost).ti,ab. (4340)

90 (metabolic adj cost).ti,ab. (1524)

91 ((energy or oxygen) adj expenditure).ti,ab. (26543)

92 or/89-91 (31393)

93 88 not 92 (1029899)

94 exp Budgets/ (13869)

95 exp models, economic/ (15736)

96 "Value of Life"/ (5756)

97 ec.fs. (436585)

98 Income/ (31189)

99 Remuneration/ (334)

100 "Salaries and Fringe Benefits"/ (15866)

101 exp "Fees and Charges"/ (30836)

102 (earn$ or expens$ or fee or fees or financ$ or fiscal$ or income$1 or money$ or monetary or paid or pay or pays or paying or payment$1 or remunerat$ or salar$ or wage$1).ti,ab,kf. (534225)

103 or/94-102 (891404)

104 93 or 103 (1561429)

105 Health Resources/ or "Supply & Distribution".fs. or exp Resource Allocation/ (98589)

106 (burden$ or resource$1).ti. (81611)

107 (burden$ adj6 (care or caring or disease$ or healthcare or illness$ or sickness$ or therap$ or treatment$)).ti,ab,kf. (76773)

108 (((resource$1 or healthcare or health-care) adj6 (allocat$ or consum$ or ration$ or usage$ or use$1 or utilis$ or utiliz$)) or hcru).ti,ab,kf. (147453)

109 Office Visits/sn, td or "Facilities and Services Utilization"/ or "Equipment and Supplies Utilization"/ or "Procedures and Techniques Utilization"/ (4925)

110 (visit or visits or visited or visiting).ti,ab,kf. (246573)

111 appointment$.ti,ab,kf. (26896)

112 Hospitalization/ (119120)

113 (hospitalization$1 or hospitalisation$1 or hospitalised or hospitalized).ti,ab,kf. (276521)

114 (admission$1 or readmission$1 or admitted or readmitted).ti,ab,kf. (429405)

115 "length of stay"/ (94918)

116 hospital stay$1.ti,ab,kf. (91572)

117 (bed adj3 day$1).ti,ab,kf. (3797)

118 ((days or time or length or duration$1) adj3 hospital$).ti,ab,kf. (101115)

119 ((days or time or length or duration$1) adj3 (stay or stays or stayed)).ti,ab,kf. (114020)

120 ((days or time or length or duration$1) adj3 (discharge or discharged or home or homes)).ti,ab,kf. (26570)

121 or/105-120 (1343705)

122 76 and 104 (1004)

123 76 and 121 (1068)

124 122 or 123 (1974)

125 exp Animals/ not Humans/ (4871784)

126 124 not 125 (1788)

127 limit 126 to english language (1538)

1. **Source: Embase**

Interface / URL: OvidSP

Database coverage dates: 1974 to 2021 August 10

Search date: 11/08/21

Retrieved records: 2728

Search strategy:

1 "disorders of mitochondrial functions"/ (23247)

2 mitochondrial dna disorder/ or mitochondrial encephalomyopathy/ or mitochondrial encephalopathy/ or mitochondrial myopathy/ (4913)

3 (mitochondri$ adj6 (disease$ or disorder$ or syndrome$)).ti,ab,kw,dq. (25044)

4 (mitochondri$ adj6 (cytopath$ or deficien$ or dysfunction$ or encephalomyopath$ or encephalopath$ or myopath$)).ti,ab,kw,dq. (49421)

5 mitochondriopath$.ti,ab,kw,dq. (558)

6 (mitochondri$ adj6 (epilep$ or seizure$ or convuls$)).ti,ab,kw,dq. (822)

7 or/1-6 (71703)

8 Leigh disease/ (2684)

9 ((leigh$ or leigh-feigin$ or leigh-feigin-wolf$) adj6 (disease$ or disorder$ or syndrome$)).ti,ab,kw,dq. (2293)

10 ((leigh$ or leigh-feigin$ or leigh-feigin-wolf$) adj6 (encephalopath$ or encephalomyelopath$)).ti,ab,kw,dq. (377)

11 ((subacute or sub-acute) adj3 (necrot$ encephal$ or necrot$ juvenile encephal$ or necrot$ infantile encephal$)).ti,ab,kw,dq. (260)

12 (leigh$ and mitochondri$).ti,ab,kw,dq. (1756)

13 or/8-12 (3248)

14 DNA directed DNA polymerase gamma/ (2214)

15 (polymerase$ adj3 gamma).ti,ab,kw,dq. (1610)

16 (polg or polg1).ti,ab,kw,dq. (1533)

17 (polgone or polgi).ti,ab,kw,dq. (4)

18 (mitochondri$ adj6 polymerase$).ti,ab,kw,dq. (2205)

19 or/14-18 (5211)

20 Alpers disease/ or Schilder disease/ (710)

21 ((alper$ or alper-huttenlocher$ or alpers-huttenlocher$) adj6 (disease$ or disorder$ or syndrome$)).ti,ab,kw,dq. (369)

22 ((alper$ or alper-huttenlocher$ or alpers-huttenlocher$) adj6 (diffuse cerebral or diffuse degeneration or poliodystroph$)).ti,ab,kw,dq. (17)

23 (schilder$ adj6 (disease$ or disorder$ or syndrome$)).ti,ab,kw,dq. (176)

24 (schilder$ adj6 (diffuse cerebral or diffuse degeneration or poliodystroph$)).ti,ab,kw,dq. (12)

25 (balo$ adj3 concentric scleros$).ti,ab,kw,dq. (270)

26 (progressive adj3 poliodystroph$).ti,ab,kw,dq. (15)

27 (diffuse cerebral degeneration$ or diffuse cerebral scleros$ or encephalitis periaxialis or myelinoclastic diffuse scleros$ or poliodystrophia cerebri or progressive neuronal degeneration$ or sudanophilic cerebral scleros$).ti,ab,kw,dq. (227)

28 ahs.ti,ab,kw,dq. (2151)

29 or/20-28 (3412)

30 myocerebrohepatopath$.ti,ab,kw,dq. (23)

31 mchs.ti,ab,kw,dq. (125)

32 or/30-31 (140)

33 spinocerebellar degeneration/ and exp epilepsy/ (464)

34 (myoclon$ and epilep$ and sensory ataxia$).ti,ab,kw,dq. (23)

35 memsa.ti,ab,kw,dq. (19)

36 (spinocerebellar ataxia$ and epilep$).ti,ab,kw,dq. (262)

37 scae.ti,ab,kw,dq. (86)

38 or/33-37 (696)

39 chronic progressive external ophthalmoplegia/ (1017)

40 external ophthalmoplegi$.ti,ab,kw,dq. (2113)

41 ocular muscular dystroph$.ti,ab,kw,dq. (6)

42 (peo or cpeo$ or arpeo or adpeo).ti,ab,kw,dq. (5632)

43 ((graefe$ or graefe fuch$) adj6 (disease$ or disorder$ or syndrome$)).ti,ab,kw,dq. (11)

44 ((graefe$ or graefe fuch$) adj6 myopath$).ti,ab,kw,dq. (3)

45 or/39-44 (7419)

46 ataxia neuropath$.ti,ab,kw,dq. (216)

47 ataxic neuropath$.ti,ab,kw,dq. (399)

48 miras.ti,ab,kw,dq. (127)

49 sando.ti,ab,kw,dq. (119)

50 or/46-49 (786)

51 MELAS syndrome/ (2958)

52 melas.ti,ab,kw,dq. (2933)

53 myoencephalopath$.ti,ab,kw,dq. (12)

54 or/51-53 (3979)

55 MERRF syndrome/ (723)

56 merrf.ti,ab,kw,dq. (707)

57 merff.ti,ab,kw,dq. (35)

58 fukuhara$.ti,ab,kw,dq. (64)

59 (epilep$ and ragged red).ti,ab,kw,dq. (624)

60 or/55-59 (1282)

61 arginine transfer RNA ligase/ (224)

62 (mitochondri$ and arg$ and (tRNA or RNA or ribonucleic acid)).ti,ab,kw,dq. (651)

63 (rars2 or rars-2).ti,ab,kw,dq. (130)

64 (rarstwo or rars-two or rarsii or rars-ii or rars11 or rars-11).ti,ab,kw,dq. (7)

65 or/61-64 (949)

66 cerebellum hypoplasia/ or olivopontocerebellar atrophy/ or (pontocerebellar adj6 hypoplasia$).ti,ab,kw,dq. (3265)

67 (pontocerebellum adj6 hypoplasia$).ti,ab,kw,dq. (5)

68 (pch6 or pch-6).ti,ab,kw,dq. (42)

69 (pchsix or pch-six or pchvi or pch-vi).ti,ab,kw,dq. (1)

70 or/66-69 (3281)

71 pyruvate dehydrogenase complex deficiency/ (467)

72 (pyruvate dehydrogenase adj6 deficien$).ti,ab,kw,dq. (691)

73 ((pdhc or pdh or pyruvate decarboxylase) adj6 deficien$).ti,ab,kw,dq. (351)

74 ((ataxia or ataxic) adj6 (lactic acidosis or pyruvate or decarboxylase)).ti,ab,kw,dq. (146)

75 or/71-74 (1104)

76 7 or 13 or 19 or 29 or 32 or 38 or 45 or 50 or 54 or 60 or 65 or 70 or 75 (92930)

77 Health Economics/ (33584)

78 exp Economic Evaluation/ (322108)

79 exp Health Care Cost/ (306327)

80 pharmacoeconomics/ (8666)

81 (econom$ or cost or costs or costly or costing or price or prices or pricing or pharmacoeconomic$).ti,ab. (1177113)

82 (expenditure$ not energy).ti,ab. (44000)

83 (value adj2 money).ti,ab. (2624)

84 budget$.ti,ab. (41576)

85 or/77-84 (1447621)

86 (metabolic adj cost).ti,ab. (1633)

87 ((energy or oxygen) adj cost).ti,ab. (4587)

88 ((energy or oxygen) adj expenditure).ti,ab. (33647)

89 or/86-88 (38725)

90 85 not 89 (1439673)

91 economics/ (241753)

92 cost/ (59605)

93 budget/ (30765)

94 device economics/ (57)

95 exp economic model/ (2668)

96 socioeconomics/ (146996)

97 (pe or de).fs. (85165)

98 income/ (62871)

99 remuneration/ (1646)

100 "salary and fringe benefit"/ or salary/ (19708)

101 exp fee/ (41166)

102 (expens$ or earning$ or salar$ or wage$1 or pay or pays or paid or paying or payment$1 or income$1 or remunerat$ or financ$ or money or monetary or fee or fees or charg$).ti,ab,kw,dq. (931821)

103 or/91-102 (1381138)

104 90 or 103 (2374458)

105 exp health care utilization/ (78896)

106 drug utilization/ (21076)

107 exp "utilization review"/ (66700)

108 health care planning/ or resource management/ or resource allocation/ (131513)

109 disease burden/ (26611)

110 hospital utilization/ (2395)

111 hospital bed utilization/ (3861)

112 (burden$ or resource$1).ti. (109377)

113 (burden$ adj6 (care or caring or disease$ or healthcare or illness$ or sickness$ or therap$ or treatment$)).ti,ab,kw,dq. (119389)

114 (((resource$1 or healthcare or health-care) adj6 (allocat$ or consum$ or ration$ or usage$ or use$1 or utilis$ or utiliz$)) or hcru).ti,ab,kw,dq. (204993)

115 (visit or visits or visited or visiting).ti,ab,kw,dq. (401988)

116 appointment$.ti,ab,kw,dq. (47168)

117 hospitalization/ or child hospitalization/ or hospital admission/ (617313)

118 (hospitalization$1 or hospitalisation$1 or hospitalised or hospitalized).ti,ab,kw,dq. (448175)

119 (admission$1 or readmission$1 or admitted or readmitted).ti,ab,kw,dq. (733501)

120 "length of stay"/ (214004)

121 hospital stay$1.ti,ab,kw,dq. (151371)

122 (bed adj3 day$1).ti,ab,kw,dq. (6269)

123 ((days or time or length or duration$1) adj3 hospital$).ti,ab,kw,dq. (170485)

124 ((days or time or length or duration$1) adj3 (stay or stays or stayed)).ti,ab,kw,dq. (202796)

125 ((days or time or length or duration$1) adj3 (discharge or discharged or home or homes)).ti,ab,kw,dq. (48120)

126 or/105-125 (2322199)

127 76 and 104 (2449)

128 76 and 126 (2575)

129 127 or 128 (4802)

130 (animal/ or animal experiment/ or animal model/ or animal tissue/ or nonhuman/) not exp human/ (6273181)

131 conference abstract.pt. (4151130)

132 129 not (130 or 131) (2990)

133 limit 132 to english language (2728)

1. **Source: Cochrane Database of Systematic Reviews (CDSR)**

Interface / URL: Cochrane Library / Wiley

Database coverage dates: Information not found. Issue searched: Issue 8 of 12, August 2021

Search date: 11/08/2021

Retrieved records: 8

Search strategy:

#1 MeSH descriptor: [Mitochondrial Diseases] this term only 52

#2 MeSH descriptor: [Mitochondrial Myopathies] this term only 31

#3 MeSH descriptor: [Mitochondrial Encephalomyopathies] this term only 10

#4 (mitochondri* NEAR/6 (disease* OR disorder* OR syndrome*)):ti,ab,kw 340

#5 (mitochondri* NEAR/6 (cytopath* OR deficien* OR dysfunction* OR encephalomyopath* OR encephalopath* OR myopath*)):ti,ab,kw 479

#6 mitochondriopath*:ti,ab,kw 8

#7 (mitochondri* NEAR/6 (epilep* OR seizure* OR convuls*)):ti,ab,kw 9

#8 #1 OR #2 OR #3 OR #4 OR #5 OR #6 OR #7 695

#9 MeSH descriptor: [Leigh Disease] this term only 4

#10 ((leigh* OR leigh-feigin* OR leigh-feigin-wolf*) NEAR/6 (disease* OR disorder* OR syndrome*)):ti,ab,kw 11

#11 ((leigh* OR leigh-feigin* OR leigh-feigin-wolf*) NEAR/6 (encephalopath* OR encephalomyelopath*)):ti,ab,kw 0

#12 ((subacute OR sub-acute) NEAR/3 (necrot* NEXT encephal* OR necrot* NEXT juvenile NEXT encephal* OR necrot* NEXT infantile NEXT encephal*)):ti,ab,kw 0

#13 (leigh* AND mitochondri*):ti,ab,kw 8

#14 #9 OR #10 OR #11 OR #12 OR #13 11

#15 MeSH descriptor: [DNA Polymerase gamma] this term only 1

#16 (polymerase* NEAR/3 gamma):ti,ab,kw 12

#17 (polg OR polg1):ti,ab,kw 3

#18 (polgone OR polgi):ti,ab,kw 0

#19 (mitochondri* NEAR/6 polymerase*):ti,ab,kw 19

#20 #15 or #16 or #17 or #18 or #19 22

#21 MeSH descriptor: [Diffuse Cerebral Sclerosis of Schilder] this term only 7

#22 ((alper* OR alper-huttenlocher* OR alpers-huttenlocher*) NEAR/6 (disease* OR disorder* OR syndrome*)):ti,ab,kw 3

#23 ((alper* OR alper-huttenlocher* OR alpers-huttenlocher*) NEAR/6 ("diffuse cerebral" OR "diffuse degeneration" OR poliodystroph*)):ti,ab,kw 0

#24 (schilder* NEAR/6 (disease* OR disorder* OR syndrome*)):ti,ab,kw 1

#25 (schilder* NEAR/6 ("diffuse cerebral" OR "diffuse degeneration" OR poliodystroph*)):ti,ab,kw 7

#26 (balo* NEAR/3 (concentric NEXT scleros*)):ti,ab,kw 0

#27 (progressive NEAR/3 poliodystroph*):ti,ab,kw 0

#28 (diffuse NEXT cerebral NEXT degeneration* OR diffuse NEXT cerebral NEXT scleros* OR "encephalitis periaxialis" OR myelinoclastic NEXT diffuse NEXT scleros* OR "poliodystrophia cerebri" OR progressive NEXT neuronal NEXT degeneration* OR sudanophilic NEXT cerebral NEXT scleros*):ti,ab,kw 10

#29 ahs:ti,ab,kw 104

#30 #21 OR #22 OR #23 OR #24 OR #25 OR #26 OR #27 OR #28 OR #29 118

#31 myocerebrohepatopath*:ti,ab,kw 0

#32 mchs:ti,ab,kw 2

#33 #31 OR #32 2

#34 MeSH descriptor: [Spinocerebellar Ataxias] this term only 51

#35 MeSH descriptor: [Epilepsy] explode all trees 2454

#36 #34 AND #35 0

#37 (myoclon* AND epilep* AND sensory NEXT ataxia*):ti,ab,kw 0

#38 memsa:ti,ab,kw 8

#39 (spinocerebellar NEXT ataxia* AND epilep*):ti,ab,kw 1

#40 scae:ti,ab,kw 5

#41 #36 OR #37 OR #38 OR #39 OR #40 14

#42 MeSH descriptor: [Ophthalmoplegia, Chronic Progressive External] this term only 6

#43 external NEXT ophthalmoplegi*:ti,ab,kw 16

#44 ocular NEXT muscular NEXT dystroph*:ti,ab,kw 0

#45 (peo OR cpeo* OR arpeo OR adpeo):ti,ab,kw 32

#46 ((graefe* OR graefe NEXT fuch*) NEAR/6 (disease* OR disorder* OR syndrome*)):ti,ab,kw 1

#47 ((graefe* OR graefe NEXT fuch*) NEAR/6 myopath*):ti,ab,kw 0

#48 #42 OR #43 OR #44 OR #45 OR #46 OR #47 45

#49 ataxia NEXT neuropath*:ti,ab,kw 2

#50 ataxic NEXT neuropath*:ti,ab,kw 8

#51 miras:ti,ab,kw 11

#52 sando:ti,ab,kw 1

#53 #49 OR #50 OR #51 OR #52 22

#54 MeSH descriptor: [MELAS Syndrome] this term only 12

#55 melas:ti,ab,kw 57

#56 myoencephalopath*:ti,ab,kw 0

#57 #54 OR #55 OR #56 57

#58 MeSH descriptor: [MERRF Syndrome] this term only 0

#59 merrf:ti,ab,kw 2

#60 merff:ti,ab,kw 0

#61 fukuhara*:ti,ab,kw 1

#62 (epilep* AND "ragged red"):ti,ab,kw 1

#63 #58 OR #59 OR #60 OR #61 OR #62 4

#64 MeSH descriptor: [Arginine-tRNA Ligase] this term only 0

#65 (mitochondri* AND arg* AND (tRNA OR RNA OR "ribonucleic acid")):ti,ab,kw 3

#66 (rars2 OR rars-2):ti,ab,kw 5

#67 (rarstwo OR rars-two OR rarsii OR rars-ii OR rars11 OR rars-11):ti,ab,kw 0

#68 #64 OR #65 OR #66 OR #67 8

#69 MeSH descriptor: [Olivopontocerebellar Atrophies] this term only 10

#70 (pontocerebellar NEAR/6 hypoplasia*):ti,ab,kw 0

#71 (pontocerebellum NEAR/6 hypoplasia*):ti,ab,kw 0

#72 (pch6 OR pch-6):ti,ab,kw 0

#73 (pchsix OR pch-six OR pchvi OR pch-vi):ti,ab,kw 0

#74 #69 OR #70 OR #71 OR #72 OR #73 10

#75 MeSH descriptor: [Pyruvate Dehydrogenase Complex Deficiency Disease] this term only 4

#76 ("pyruvate dehydrogenase" NEAR/6 deficien*):ti,ab,kw 9

#77 ((pdhc OR pdh OR "pyruvate decarboxylase") NEAR/6 deficien*):ti,ab,kw 3

#78 ((ataxia OR ataxic) NEAR/6 ("lactic acidosis" OR pyruvate OR decarboxylase)):ti,ab,kw 1

#79 #75 OR #76 OR #77 OR #78 13

#80 #8 OR #14 OR #20 OR #30 OR #33 OR #41 OR #48 OR #53 OR #57 OR #63 OR #68 OR #74 OR #79 958

#81 #80 in Cochrane Reviews, Cochrane Protocols 8

1. **Source: Cochrane Central Register of Controlled Trials (CENTRAL)**

Interface / URL: Cochrane Library / Wiley

Database coverage dates: Information not found. Issue searched: Issue 8 of 12, August 2021

Search date: 12/08/2021

Retrieved records: 230

Search strategy:

#1 MeSH descriptor: [Mitochondrial Diseases] this term only 52

#2 MeSH descriptor: [Mitochondrial Myopathies] this term only 31

#3 MeSH descriptor: [Mitochondrial Encephalomyopathies] this term only 10

#4 mitochondri* NEAR/6 (disease* OR disorder* OR syndrome*) 412

#5 mitochondri* NEAR/6 (cytopath* OR deficien* OR dysfunction* OR encephalomyopath* OR encephalopath* OR myopath*) 522

#6 mitochondriopath* 9

#7 mitochondri* NEAR/6 (epilep* OR seizure* OR convuls*) 9

#8 #1 OR #2 OR #3 OR #4 OR #5 OR #6 OR #7 787

#9 MeSH descriptor: [Leigh Disease] this term only 4

#10 (leigh* OR leigh-feigin* OR leigh-feigin-wolf*) NEAR/6 (disease* OR disorder* OR syndrome*) 19

#11 (leigh* OR leigh-feigin* OR leigh-feigin-wolf*) NEAR/6 (encephalopath* OR encephalomyelopath*) 1

#12 (subacute OR sub-acute) NEAR/3 (necrot* NEXT encephal* OR necrot* NEXT juvenile NEXT encephal* OR necrot* NEXT infantile NEXT encephal*) 0

#13 leigh* AND mitochondri* 17

#14 #9 OR #10 OR #11 OR #12 OR #13 22

#15 MeSH descriptor: [DNA Polymerase gamma] this term only 1

#16 polymerase* NEAR/3 gamma 25

#17 polg OR polg1 4

#18 polgone OR polgi 0

#19 mitochondri* NEAR/6 polymerase* 27

#20 #15 or #16 or #17 or #18 or #19 41

#21 MeSH descriptor: [Diffuse Cerebral Sclerosis of Schilder] this term only 7

#22 (alper* OR alper-huttenlocher* OR alpers-huttenlocher*) NEAR/6 (disease* OR disorder* OR syndrome*) 4

#23 (alper* OR alper-huttenlocher* OR alpers-huttenlocher*) NEAR/6 ("diffuse cerebral" OR "diffuse degeneration" OR poliodystroph*) 0

#24 schilder* NEAR/6 (disease* OR disorder* OR syndrome*) 8

#25 schilder* NEAR/6 ("diffuse cerebral" OR "diffuse degeneration" OR poliodystroph*) 7

#26 balo* NEAR/3 (concentric NEXT scleros*) 0

#27 progressive NEAR/3 poliodystroph* 0

#28 diffuse NEXT cerebral NEXT degeneration* OR diffuse NEXT cerebral NEXT scleros* OR "encephalitis periaxialis" OR myelinoclastic NEXT diffuse NEXT scleros* OR "poliodystrophia cerebri" OR progressive NEXT neuronal NEXT degeneration* OR sudanophilic NEXT cerebral NEXT scleros* 10

#29 ahs 324

#30 #21 OR #22 OR #23 OR #24 OR #25 OR #26 OR #27 OR #28 OR #29 346

#31 myocerebrohepatopath* 0

#32 mchs 2

#33 #31 OR #32 2

#34 MeSH descriptor: [Spinocerebellar Ataxias] this term only 51

#35 MeSH descriptor: [Epilepsy] explode all trees 2454

#36 #34 AND #35 0

#37 myoclon* AND epilep* AND sensory NEXT ataxia* 0

#38 memsa 29

#39 spinocerebellar NEXT ataxia* AND epilep* 3

#40 scae 5

#41 #36 OR #37 OR #38 OR #39 OR #40 37

#42 MeSH descriptor: [Ophthalmoplegia, Chronic Progressive External] this term only 6

#43 external NEXT ophthalmoplegi* 18

#44 ocular NEXT muscular NEXT dystroph* 0

#45 peo OR cpeo* OR arpeo OR adpeo 46

#46 (graefe* OR graefe NEXT fuch*) NEAR/6 (disease* OR disorder* OR syndrome*) 1

#47 (graefe* OR graefe NEXT fuch*) NEAR/6 myopath* 0

#48 #42 OR #43 OR #44 OR #45 OR #46 OR #47 60

#49 ataxia NEXT neuropath* 3

#50 ataxic NEXT neuropath* 10

#51 miras 87

#52 sando 82

#53 #49 OR #50 OR #51 OR #52 182

#54 MeSH descriptor: [MELAS Syndrome] this term only 12

#55 melas 94

#56 myoencephalopath* 0

#57 #54 OR #55 OR #56 94

#58 MeSH descriptor: [MERRF Syndrome] this term only 0

#59 merrf 4

#60 merff 1

#61 fukuhara* 204

#62 epilep* AND "ragged red" 2

#63 #58 OR #59 OR #60 OR #61 OR #62 209

#64 MeSH descriptor: [Arginine-tRNA Ligase] this term only 0

#65 mitochondri* AND arg* AND (tRNA OR RNA OR "ribonucleic acid") 9

#66 rars2 OR rars-2 5

#67 rarstwo OR rars-two OR rarsii OR rars-ii OR rars11 OR rars-11 0

#68 #64 OR #65 OR #66 OR #67 14

#69 MeSH descriptor: [Olivopontocerebellar Atrophies] this term only 10

#70 pontocerebellar NEAR/6 hypoplasia* 0

#71 pontocerebellum NEAR/6 hypoplasia* 0

#72 pch6 OR pch-6 0

#73 pchsix OR pch-six OR pchvi OR pch-vi 0

#74 #69 OR #70 OR #71 OR #72 OR #73 10

#75 MeSH descriptor: [Pyruvate Dehydrogenase Complex Deficiency Disease] this term only 4

#76 "pyruvate dehydrogenase" NEAR/6 deficien* 12

#77 (pdhc OR pdh OR "pyruvate decarboxylase") NEAR/6 deficien* 3

#78 (ataxia OR ataxic) NEAR/6 ("lactic acidosis" OR pyruvate OR decarboxylase) 1

#79 #75 OR #76 OR #77 OR #78 16

#80 #8 OR #14 OR #20 OR #30 OR #33 OR #41 OR #48 OR #53 OR #57 OR #63 OR #68 OR #74 OR #79 1737

#81 MeSH descriptor: [Economics] this term only 41

#82 MeSH descriptor: [Costs and Cost Analysis] explode all trees 10920

#83 MeSH descriptor: [Economics, Dental] this term only 2

#84 MeSH descriptor: [Economics, Hospital] explode all trees 728

#85 MeSH descriptor: [Economics, Medical] this term only 26

#86 MeSH descriptor: [Economics, Nursing] this term only 12

#87 MeSH descriptor: [Economics, Pharmaceutical] this term only 65

#88 economic* OR cost OR costs OR costly OR costing OR price OR prices OR pricing OR pharmacoeconomic* 94949

#89 expenditure* NOT energy 2299

#90 "value for money" 318

#91 budget* 1504

#92 #81 OR #82 OR #83 OR #84 OR #85 OR #86 OR #87 OR #88 OR #89 OR #90 OR #91 96036

#93 "energy cost" OR "oxygen cost" 492

#94 "metabolic cost" 136

#95 "energy expenditure" OR "oxygen expenditure" 4945

#96 #93 OR #94 OR #95 5423

#97 #92 NOT #96 95107

#98 MeSH descriptor: [Budgets] explode all trees 28

#99 MeSH descriptor: [Models, Economic] explode all trees 362

#100 MeSH descriptor: [Value of Life] this term only 33

#101 MeSH descriptor: [] explode all trees and with qualifier(s): [economics - EC] 11818

#102 MeSH descriptor: [Income] this term only 331

#103 MeSH descriptor: [Remuneration] this term only 11

#104 MeSH descriptor: [Salaries and Fringe Benefits] this term only 51

#105 MeSH descriptor: [Fees and Charges] explode all trees 258

#106 earn* OR expens* OR fee OR fees OR financ* OR fiscal* OR income* OR money* OR monetary OR paid OR pay OR pays OR paying OR payment* OR remunerat* OR salar* OR wage* 43117

#107 #98 OR #99 OR #100 OR #101 OR #102 OR #103 OR #104 OR #105 OR #106 51698

#108 #97 OR #107 119991

#109 MeSH descriptor: [Health Resources] this term only 428

#110 MeSH descriptor: [] explode all trees and with qualifier(s): [supply & distribution - SD] 386

#111 MeSH descriptor: [Resource Allocation] explode all trees 74

#112 (burden* OR resource*):ti 3796

#113 burden* adj6 (care OR caring OR disease* OR healthcare OR illness* OR sickness* OR therap* OR treatment*) 146

#114 (resource* OR healthcare OR health-care) NEAR/6 (allocat* OR consum* OR ration* OR usage* OR use* OR utilis* OR utiliz*) 19738

#115 HCRU 83

#116 MeSH descriptor: [Office Visits] this term only and with qualifier(s): [statistics & numerical data - SN] 135

#117 MeSH descriptor: [Office Visits] this term only and with qualifier(s): [trends - TD] 4

#118 MeSH descriptor: [Facilities and Services Utilization] this term only 35

#119 MeSH descriptor: [Equipment and Supplies Utilization] this term only 0

#120 MeSH descriptor: [Procedures and Techniques Utilization] this term only 8

#121 visit OR visits OR visited OR visiting 88528

#122 appointment* 8665

#123 MeSH descriptor: [Hospitalization] this term only 5452

#124 hospitalization* OR hospitalisation* OR hospitalised OR hospitalized 60357

#125 admission* OR readmission* OR admitted OR readmitted 57237

#126 MeSH descriptor: [Length of Stay] this term only 7471

#127 hospital stay* 37523

#128 bed NEAR/3 day* 853

#129 (days OR time OR length OR duration*) NEAR/3 hospital* 30296

#130 (days OR time OR length OR duration*) NEAR/3 (stay OR stays OR stayed) 32011

#131 (days OR time OR length OR duration*) NEAR/3 (discharge OR discharged OR home OR homes) 9109

#132 #109 OR #110 OR #111 OR #112 OR #113 OR #114 OR #115 OR #116 OR #117 OR #118 OR #119 OR #120 OR #121 OR #122 OR #123 OR #124 OR #125 OR #126 OR #127 OR #128 OR #129 OR #130 OR #131 224715

#133 #80 AND #108 279

#134 #80 AND #132 364

#135 #133 OR #134 454

#136 #135 in Trials 230

1. **Source: HTA Database**

Interface / URL: https://database.inahta.org/

Database coverage dates: Information not found. The former database was produced by the CRD until March 2018, at which time the addition of records was stopped because INAHTA was in the process of rebuilding the new database platform. In July 2019, the database records were exported from the CRD platform and imported into the new platform that was developed by INAHTA. The rebuild of the new platform was launched in June 2020.

Search date: 12/08/2021

Retrieved records: 23

Search strategy:

54 #53 OR #52 OR #51 OR #50 OR #49 OR #48 OR #47 OR #46 OR #45 OR #44 OR #43 OR #42 OR #41 OR #40 OR #39 OR #38 OR #37 OR #36 OR #35 OR #34 OR #33 OR #32 OR #31 OR #30 OR #29 OR #28 OR #27 OR #26 OR #25 OR #24 OR #23 OR #22 OR #21 OR #20 OR #19 OR #18 OR #17 OR #16 OR #15 OR #14 OR #13 OR #12 OR #11 OR #10 OR #9 OR #8 OR #7 OR #6 OR #5 OR #4 OR #3 OR #2 OR #1 23

53 (ataxia OR ataxic) AND ("lactic acidosis" OR pyruvate OR decarboxylase) 0

52 (pdhc OR pdh OR "pyruvate decarboxylase") AND deficien* 0

51 ("pyruvate dehydrogenase" AND deficien*) 0

50 "Pyruvate Dehydrogenase Complex Deficiency Disease"[mh] 0

49 (pchsix OR "pch-six" OR pchvi OR "pch-vi") 0

48 (pch6 OR "pch-6") 0

47 (pontocerebellum AND hypoplasia*) 0

46 (pontocerebellar AND hypoplasia*) 0

45 "Olivopontocerebellar Atrophies"[mh] 0

44 (rarstwo OR "rars-two" OR rarsii OR "rars-ii" OR rars11 OR "rars-11") 0

43 (rars2 OR "rars-2") 0

42 "Arginine-tRNA Ligase"[mh] 0

41 epilep* AND "ragged red" 0

40 fukuhara* 0

39 merff 0

38 merrf 0

37 "MERRF Syndrome"[mh] 0

36 myoencephalopath* 0

35 melas 0

34 "MELAS Syndrome"[mh] 0

33 sando 0

32 miras 0

31 ataxic AND neuropath* 0

30 ataxia AND neuropath* 4

29 graefe* 0

28 (peo OR cpeo* OR arpeo OR adpeo) 1

27 "ocular muscular" 0

26 ophthalmoplegi* 2

25 "Ophthalmoplegia, Chronic Progressive External"[mh] 0

24 scae 0

23 spinocerebellar AND ataxia* AND epilep* 1

22 memsa 1

21 myoclon* AND epilep* AND ataxia* 3

20 "Spinocerebellar Ataxias"[mh] AND "Epilepsy"[mhe] 0

19 mchs 1

18 myocerebrohepatopath* 1

17 ahs 1

16 ("diffuse cerebral" OR "encephalitis periaxialis" OR "myelinoclastic diffuse" OR "poliodystrophia cerebri" OR "progressive neuronal" OR "sudanophilic cerebral") 0

15 poliodystroph* 0

14 balo* AND concentric 0

13 schilder* 3

12 alper* 1

11 "Diffuse Cerebral Sclerosis of Schilder"[mh] 0

10 (polgone OR polgi) 0

9 (polg OR polg1) 1

8 (polymerase* AND gamma) 1

7 "DNA Polymerase gamma"[mh] 0

6 (subacute OR "sub-acute") AND encephal* 0

5 leigh* 3

4 "Leigh Disease"[mh] 0

3 mitochondri* 11

2 "Mitochondrial Myopathies"[mh] OR "Mitochondrial Encephalomyopathies"[mh] 1

1 "Mitochondrial Diseases"[mh] 1

1. **Source: Cost-Effectiveness Analysis Registry**

Interface / URL: http://healtheconomics.tuftsmedicalcenter.org/cear4/home.aspx

Database coverage dates: The webpage at the above URL states that the database includes studies published from 1976 to 2019. However, a check of the actual content indicates that records with 2020 and 2021 publication dates are available to be found in the database on the date of search.

Search date: 12/08/2021

Retrieved records: 0

Search strategy:

The basic search interface at the following URL was used: <http://healtheconomics.tuftsmedicalcenter.org/cear2n/search/search.aspx>. Search settings were left as default.

Separate searches were conducted on each of the following terms. Returned results were screened by the information specialist for potential relevance to the populations of interest. Potentially relevant results were cross-checked against results from other search sources to see if already retrieved. Any potentially relevant results not identified via another source were retrieved for further assessment.

mitochondri = 0 (1 result returned, excluded)

leigh = 0 (23 results returned, excluded)

subacute = 0 (15 results returned, excluded)

sub-acute = 0 (4 results returned, excluded)

polymerase = 0 (37 results returned, excluded)

polg = 0 (0 results returned)

alper = 0 (25 results returned, excluded)

schilder = 0 (1 result returned, excluded)

concentric = 0 (0 results returned)

poliodystroph = 0 (0 results returned)

diffuse cerebral = 0 (0 results returned)

encephalitis periaxialis = 0 (0 results returned)

myelinoclastic diffuse = 0 (0 results returned)

progressive neuronal = 0 (0 results returned)

sudanophilic cerebral = 0 (0 results returned)

ahs = 0 (9 results returned, excluded)

myocerebrohepatopath = 0 (0 results returned)

mchs = 0 (0 results returned)

sensory ataxia = 0 (0 results returned)

memsa = 0 (0 results returned)

spinocerebellar ataxia = 0 (0 results returned)

scae = 0 (0 results returned)

external ophthalmoplegi = 0 (0 results returned)

ocular muscular dystroph = 0 (0 results returned)

peo[space] = 0 (0 results returned) *

cpeo = 0 (0 results returned)

arpeo = 0 (0 results returned)

adpeo = 0 (0 results returned)

graefe = 0 (3 results returned, excluded)

ataxia neuropath = 0 (0 results returned)

ataxic neuropath = 0 (0 results returned)

miras = 0 (1 result returned, excluded)

sando = 0 (2 results returned, excluded)

melas = 0 (2 results returned, excluded)

myoencephalopath = 0 (0 results returned)

merrf = 0 (0 results returned)

merff = 0 (0 results returned)

fukuhara = 0 (4 results returned, excluded)

ragged red = 0 (0 results returned)

rars = 0 (0 results returned)

pontocerebellar = 0 (0 results returned)

pontocerebellum = 0 (0 results returned)

pch = 0 (4 results returned, excluded)

pdh = 0 (0 results returned)

pyruvate = 0 (0 results returned)

lactic acidosis = 0 (0 results returned)

decarboxylase = 0 (0 results returned)

Search notes:

* The search term *peo* when entered was followed by a space.

1. **Source: NHS Economic Evaluation Database (NHS EED)**

Interface / URL: https://www.crd.york.ac.uk/CRDWeb

Database coverage dates: Information not found. Bibliographic records were published on NHS EED until 31st March 2015. Searches of MEDLINE, Embase, CINAHL, PsycINFO and PubMed were continued until the end of the 2014.

Search date: 13/08/2021

Retrieved records: 27

Search strategy:

1 MeSH DESCRIPTOR mitochondrial diseases 3

2 MeSH DESCRIPTOR mitochondrial myopathies 0

3 MeSH DESCRIPTOR mitochondrial encephalomyopathies 0

4 (mitochondri*) 29

5 MeSH DESCRIPTOR Leigh Disease 0

6 ((leigh* AND (disease* OR disorder* OR syndrome* OR encephalopath* OR encephalomyelopath*))) 16

7 (((subacute OR sub-acute) AND encephal*)) 3

8 MeSH DESCRIPTOR DNA Polymerase gamma 0

9 ((polymerase* AND gamma)) 1

10 (polg*) 2

11 MeSH DESCRIPTOR Diffuse Cerebral Sclerosis of Schilder 0

12 ((alper* AND (disease* OR disorder* OR syndrome* ))) 14

13 (diffuse cerebral OR diffuse degeneration OR poliodystroph*) 0

14 ((schilder* AND (disease* OR disorder* OR syndrome* ))) 12

15 (concentric scleros*) 0

16 (encephalitis periaxialis OR myelinoclastic diffuse scleros* OR progressive neuronal degeneration* OR sudanophilic cerebral scleros*) 0

17 (ahs) 5

18 (myocerebrohepatopath*) 1

19 (mchs) 1

20 MeSH DESCRIPTOR Spinocerebellar Ataxias 9

21 ((myoclon* AND epilep* AND sensory ataxia*)) 1

22 (memsa) 1

23 ((spinocerebellar ataxia* AND epilep*)) 1

24 (scae) 0

25 MeSH DESCRIPTOR Ophthalmoplegia, Chronic Progressive External 0

26 (external ophthalmoplegi*) 1

27 (ocular muscular dystroph*) 0

28 ((peo OR cpeo* OR arpeo OR adpeo)) 3

29 ((graefe* AND (disease* OR disorder* OR syndrome* OR myopath*))) 3

30 ((ataxia neuropath* OR ataxic neuropath* OR miras OR sando)) 1

31 MeSH DESCRIPTOR MELAS Syndrome EXPLODE ALL TREES 0

32 ((melas OR myoencephalopath*)) 0

33 MeSH DESCRIPTOR MERRF Syndrome 0

34 ((merrf OR merff OR fukuhara* OR ragged red)) 12

35 MeSH DESCRIPTOR Arginine-tRNA Ligase 0

36 (rars*) 0

37 MeSH DESCRIPTOR Olivopontocerebellar Atrophies 0

38 ((pontocerebellar ADJ6 hypoplasia*)) OR ((hypoplasia* ADJ6 pontocerebellar)) 0

39 ((pontocerebellum ADJ6 hypoplasia*)) OR ((hypoplasia* ADJ6 pontocerebellum)) 0

40 (pch*) 8

41 MeSH DESCRIPTOR Pyruvate Dehydrogenase Complex Deficiency Disease 0

42 ((pyruvate dehydrogenase ADJ6 deficien*)) OR ((deficien* ADJ6 pyruvate dehydrogenase)) 0

43 (((pdhc OR pdh OR pyruvate decarboxylase) ADJ6 deficien*)) OR (deficien* ADJ6 (pdhc OR pdh OR pyruvate decarboxylase)) 0

44 (((ataxia OR ataxic) ADJ6 (lactic acidosis OR pyruvate OR decarboxylase))) OR ((lactic acidosis OR pyruvate OR decarboxylase) ADJ6 (ataxia OR ataxic)) 0

45 #1 OR #2 OR #3 OR #4 OR #5 OR #6 OR #7 OR #8 OR #9 OR #10 OR #11 OR #12 OR #13 OR #14 OR #15 OR #16 OR #17 OR #18 OR #19 OR #20 OR #21 OR #22 OR #23 OR #24 OR #25 OR #26 OR #27 OR #28 OR #29 OR #30 OR #31 OR #32 OR #33 OR #34 OR #35 OR #36 OR #37 OR #38 OR #39 OR #40 OR #41 OR #42 OR #43 OR #44 112

46 (#45) IN NHSEED 27

1. **Source: EconLit**

Interface / URL: OvidSP

Database coverage dates: 1886 to August 05, 2021

Search date: 12/08/2021

Retrieved records: 79

Search strategy:

1 (mitochondri$ adj6 (disease$ or disorder$ or syndrome$)).af. (0)

2 (mitochondri$ adj6 (cytopath$ or deficien$ or dysfunction$ or encephalomyopath$ or encephalopath$ or myopath$)).af. (0)

3 mitochondriopath$.af. (0)

4 (mitochondri$ adj6 (epilep$ or seizure$ or convuls$)).af. (0)

5 or/1-4 (0)

6 ((leigh$ or leigh-feigin$ or leigh-feigin-wolf$) adj6 (disease$ or disorder$ or syndrome$)).af. (0)

7 ((leigh$ or leigh-feigin$ or leigh-feigin-wolf$) adj6 (encephalopath$ or encephalomyelopath$)).af. (0)

8 ((subacute or sub-acute) adj3 (necrot$ encephal$ or necrot$ juvenile encephal$ or necrot$ infantile encephal$)).af. (0)

9 (leigh$ and mitochondri$).af. (0)

10 or/6-9 (0)

11 (polymerase$ adj3 gamma).af. (0)

12 (polg or polg1).af. (0)

13 (polgone or polgi).af. (0)

14 (mitochondri$ adj6 polymerase$).af. (0)

15 or/11-14 (0)

16 ((alper$ or alper-huttenlocher$ or alpers-huttenlocher$) adj6 (disease$ or disorder$ or syndrome$)).af. (1)

17 ((alper$ or alper-huttenlocher$ or alpers-huttenlocher$) adj6 (diffuse cerebral or diffuse degeneration or poliodystroph$)).af. (0)

18 (schilder$ adj6 (disease$ or disorder$ or syndrome$)).af. (0)

19 (schilder$ adj6 (diffuse cerebral or diffuse degeneration or poliodystroph$)).af. (0)

20 (balo$ adj3 concentric scleros$).af. (0)

21 (progressive adj3 poliodystroph$).af. (0)

22 (diffuse cerebral degeneration$ or diffuse cerebral scleros$ or encephalitis periaxialis or myelinoclastic diffuse scleros$ or poliodystrophia cerebri or progressive neuronal degeneration$ or sudanophilic cerebral scleros$).af. (0)

23 ahs.af. (50)

24 or/16-23 (51)

25 myocerebrohepatopath$.af. (0)

26 mchs.af. (0)

27 25 or 26 (0)

28 (myoclon$ and epilep$ and sensory ataxia$).af. (0)

29 memsa.af. (0)

30 (spinocerebellar ataxia$ and epilep$).af. (0)

31 scae.af. (0)

32 or/28-31 (0)

33 external ophthalmoplegi$.af. (0)

34 ocular muscular dystroph$.af. (0)

35 (peo or cpeo$ or arpeo or adpeo).af. (6)

36 ((graefe$ or graefe fuch$) adj6 (disease$ or disorder$ or syndrome$)).af. (0)

37 ((graefe$ or graefe fuch$) adj6 myopath$).af. (0)

38 or/33-37 (6)

39 ataxia neuropath$.af. (0)

40 ataxic neuropath$.af. (0)

41 miras.af. (10)

42 sando.af. (0)

43 or/39-42 (10)

44 melas.af. (2)

45 myoencephalopath$.af. (0)

46 44 or 45 (2)

47 merrf.af. (0)

48 merff.af. (0)

49 fukuhara$.af. (10)

50 (epilep$ and ragged red).af. (0)

51 or/47-50 (10)

52 (mitochondri$ and arg$ and (tRNA or RNA or ribonucleic acid)).af. (0)

53 (rars2 or rars-2).af. (0)

54 (rarstwo or rars-two or rarsii or rars-ii or rars11 or rars-11).af. (0)

55 or/52-54 (0)

56 (pontocerebellar adj6 hypoplasia$).af. (0)

57 (pontocerebellum adj6 hypoplasia$).af. (0)

58 (pch6 or pch-6).af. (0)

59 (pchsix or pch-six or pchvi or pch-vi).af. (0)

60 or/56-59 (0)

61 (pyruvate dehydrogenase adj6 deficien$).af. (0)

62 ((pdhc or pdh or pyruvate decarboxylase) adj6 deficien$).af. (0)

63 ((ataxia or ataxic) adj6 (lactic acidosis or pyruvate or decarboxylase)).af. (0)

64 or/61-63 (0)

65 5 or 10 or 15 or 24 or 27 or 32 or 38 or 43 or 46 or 51 or 55 or 60 or 64 (79)

1. **Source: Paediatric Economic Database Evaluation**

Interface / URL: http://pede.ccb.sickkids.ca/pede/

Database coverage dates: The information at the following URL states that the database contains records for studies published from January 1, 1980 to December 31, 2019 - http://pede.ccb.sickkids.ca/pede/database.jsp.

Search date: 13/08/2021

Retrieved records: 0

Search strategy:

The basic search interface at the following URL was used: <http://pede.ccb.sickkids.ca/pede/search.jsp>. All search settings were left as default. In the default setting, terms are searched across the 'Title, abstract, or Keywords'.

The basic search interface allows the searcher to enter up to three terms combined with AND using the separate search line functionality built into the interface. Where AND is shown between terms in the syntax below, this indicates that this functionality was used. All other terms were searched for individually and not combined with any other term.

Separate searches were conducted on each of the following terms. Returned results were screened by the information specialist for potential relevance to the populations of interest. Potentially relevant results were cross-checked against results from other search sources to see if already retrieved. Any potentially relevant results not identified via another source were retrieved for further assessment.

mitochondri = 0 (0 results returned)

leigh = 0 (0 results returned)

subacute = 0 (0 results returned)

sub-acute = 0 (0 results returned)

polymerase AND gamma = 0 (0 results returned)

polg = 0 (0 results returned)

alper = 0 (0 results returned)

schilder = 0 (0 results returned)

concentric = 0 (0 results returned)

poliodystroph = 0 (0 results returned)

diffuse cerebral = 0 (0 results returned)

encephalitis periaxialis = 0 (0 results returned)

myelinoclastic diffuse = 0 (0 results returned)

progressive neuronal = 0 (0 results returned)

sudanophilic cerebral = 0 (0 results returned)

ahs = 0 (2 results returned, excluded)

myocerebrohepatopath = 0 (0 results returned)

mchs = 0 (0 results returned)

sensory ataxia = 0 (0 results returned)

memsa = 0 (0 results returned)

spinocerebellar ataxia = 0 (0 results returned)

scae = 0 (0 results returned)

external ophthalmoplegi = 0 (0 results returned)

ocular muscular dystroph = 0 (0 results returned)

peo = 0 (91 results returned, excluded)

cpeo = 0 (0 results returned)

arpeo = 0 (0 results returned)

adpeo = 0 (0 results returned)

graefe = 0 (0 results returned)

ataxia neuropath = 0 (0 results returned)

ataxic neuropath = 0 (0 results returned)

miras = 0 (0 result returned)

sando = 0 (0 results returned)

melas = 0 (0 results returned)

myoencephalopath = 0 (0 results returned)

merrf = 0 (0 results returned)

merff = 0 (0 results returned)

fukuhara = 0 (0 results returned)

ragged red = 0 (0 results returned)

rars = 0 (0 results returned)

pontocerebellar = 0 (0 results returned)

pontocerebellum = 0 (0 results returned)

pch = 0 (1 result returned, excluded)

pdh = 0 (0 results returned)

pyruvate = 0 (0 results returned)

lactic acidosis = 0 (0 results returned)

decarboxylase = 0 (0 results returned)

1. **Source: National Institute for Health and Care Excellence (NICE) webpages**

Interface / URL: https://www.nice.org.uk/

Database coverage dates: n/a

Search date: 13/08/2021

Retrieved records: 0

Search strategy:

The following documents were sought: Company Submissions, Final Appraisal Determination Documents, Assessment Reports for multiple technology appraisals, Evidence Review Group (ERG) reports for single technology appraisals.

The site-wide search interface was used at: <https://www.nice.org.uk/>. Separate searches were conducted on each term shown below.

Results were filtered by 'Document Type' to 'Guidance', then by 'Guidance Programme' to 'Technology appraisal guidance.'

Results were scanned to identify technology appraisals on the eligible mitochondrial diseases. For any identified, associated documents under the 'History' tab were viewed to check for relevant documents.

PDFs for relevant documents were retrieved for further assessment. Duplicate documents were not retrieved.

mitochondri* = 0 (1 result returned, excluded)

leigh* = 0 (8 results returned, excluded)

subacute = 0 (1 result returned, excluded)

"sub-acute" = 0 (1 result returned, excluded)

polymerase* AND gamma = 0 (1 result returned, excluded)

polg* = 0 (0 results returned)

alper* OR schilder* OR concentric OR poliodystroph* = 0 (0 results returned)

"diffuse cerebral" OR "encephalitis periaxialis" OR "myelinoclastic diffuse" OR "progressive neuronal" OR "sudanophilic cerebral" = 0 (0 results returned)

ahs = 0 (0 results returned)

myocerebrohepatopath* OR mchs = 0 (0 results returned)

myoclon* AND epilep* AND ataxia* = 0 (0 results returned)

memsa = 0 (0 results returned)

spinocerebellar AND epilep* = 0 (0 results returned)

scae = 0 (0 results returned)

external AND ophthalmoplegi* = 0 (0 results returned)

"ocular muscular" = 0 (0 results returned)

peo OR cpeo* OR arpeo OR adpeo = 0 (0 results returned)

graefe* OR ataxia OR ataxic OR miras OR sando OR melas OR myoencephalopath* OR merrf OR merff OR fukuhara* OR "ragged red" OR rars* OR pontocerebellar OR pontocerebellum OR pch* OR "pyruvate dehydrogenase" OR pdhc OR pdh OR decarboxylase = 0 (1 result returned, excluded)

0 documents were retrieved

1. **Source: Institute for Clinical and Economic Review webpages**

Interface / URL: https://icer-review.org/

Database coverage dates: n/a

Search date: 23/08/2021

Retrieved records: 0

Search strategy:

The following were sought: Final Evidence Report (or Draft Evidence Report if Final was not available) and the Evidence Presentation (if available).

Assessments were located at the following URL: <https://icer.org/explore-our-research/assessments/>.

The drop-down options were used to view all documents with status 'completed' and research type 'assessment'. 84 results were returned.

The descriptive text was checked for relevance to the eligible mitochondrial diseases. No documents were retrieved for further assessment,

1. **Source: Canadian Agency for Drugs and Technologies in Health (CADTH) webpages**

Interface / URL: https://www.cadth.ca/

Database coverage dates: n/a

Search date: 23/08/2021

Retrieved records: 0

Search strategy:

The following documents were sought: economic guidance and final recommendations associated with reimbursement reviews.

The site-wide search interface was used at: <https://www.cadth.ca/>. 'Advanced Search' was selected. Separate searches were conducted on each term shown below.

Results were filtered by 'Project Line' to 'Reimbursement Review'.

Results were assessed for relevance to the eligible mitochondrial diseases. Relevant results were checked for document types of interest.

PDFs of relevant documents were retrieved for further assessment. Duplicate documents were not retrieved.

mitochondri* = 0 (7 results returned, excluded)

leigh* = 0 (13 results returned, excluded)

subacute AND encephal* = 0 (0 results returned)

"sub-acute" AND encephal* = 0 (0 results returned)

polymerase* AND gamma = 0 (2 results returned, excluded)

polg* = 0 (0 results returned)

alper* OR schilder* OR concentric OR poliodystroph* = 0 (2 results returned, excluded)

"diffuse cerebral" OR "encephalitis periaxialis" OR "myelinoclastic diffuse" OR "progressive neuronal" = 0 (0 results returned)

"sudanophilic cerebral" = 0 (0 results returned)

ahs = 0 (1 result returned, excluded)

myocerebrohepatopath* OR mchs = 0 (0 results returned)

myoclon* AND epilep* AND ataxia* = 0 (3 results returned, excluded)

memsa = 0 (0 results returned)

spinocerebellar AND epilep* = 0 (0 results returned)

scae = 0 (0 results returned)

external AND ophthalmoplegi* = 0 (1 result returned, excluded)

"ocular muscular" = 0 (0 results returned)

peo OR cpeo* OR arpeo OR adpeo = 0 (0 results returned)

graefe* OR ataxia OR ataxic OR miras OR sando = 0 (11 results returned, excluded)

melas OR myoencephalopath* OR merrf OR merff OR fukuhara* OR "ragged red" = 0 (0 results returned)

rars* OR pontocerebellar OR pontocerebellum OR pch* = 0 (3 results returned, excluded)

"pyruvate dehydrogenase" OR pdhc OR pdh OR decarboxylase = 0 (0 results returned)

0 documents were retrieved

#### Update searches: July 2022

1. **Source: MEDLINE ALL**

Interface / URL: OvidSP

Database coverage dates: 1946 to July 26, 2022

Search date: 27/07/2022

Retrieved records: 1913

Search strategy:

1 mitochondrial diseases/ (6271)

2 mitochondrial myopathies/ or mitochondrial encephalomyopathies/ (2801)

3 (mitochondri$ adj6 (disease$ or disorder$ or syndrome$)).ti,ab,kf. (19539)

4 (mitochondri$ adj6 (cytopath$ or deficien$ or dysfunction$ or encephalomyopath$ or encephalopath$ or myopath$)).ti,ab,kf. (40895)

5 (mitochondriopath$ or (mdas not modified dental anxiety scale)).ti,ab,kf. (899)

6 (mitochondri$ adj6 (epilep$ or seizure$ or convuls$)).ti,ab,kf. (594)

7 or/1-6 (54693)

8 Leigh Disease/ (1200)

9 ((leigh$ or leigh-feigin$ or leigh-feigin-wolf$) adj6 (disease$ or disorder$ or syndrome$)).ti,ab,kf. (1741)

10 ((leigh$ or leigh-feigin$ or leigh-feigin-wolf$) adj6 (encephalopath$ or encephalomyelopath$)).ti,ab,kf. (317)

11 ((subacute or sub-acute) adj3 (necrot$ encephal$ or necrot$ juvenile encephal$ or necrot$ infantile encephal$)).ti,ab,kf. (265)

12 (leigh$ and mitochondri$).ti,ab,kf. (1262)

13 or/8-12 (2104)

14 DNA Polymerase gamma/ (847)

15 (polymerase$ adj3 gamma).ti,ab,kf. (1343)

16 (polg or polg1).ti,ab,kf. (918)

17 (polgone or polgi).ti,ab,kf. (0)

18 (mitochondri$ adj6 polymerase$).ti,ab,kf. (1912)

19 or/14-18 (3243)

20 "Diffuse Cerebral Sclerosis of Schilder"/ (2376)

21 ((alper$ or alper-huttenlocher$ or alpers-huttenlocher$) adj6 (disease$ or disorder$ or syndrome$)).ti,ab,kf. (250)

22 ((alper$ or alper-huttenlocher$ or alpers-huttenlocher$) adj6 (diffuse cerebral or diffuse degeneration or poliodystroph$)).ti,ab,kf. (19)

23 (schilder$ adj6 (disease$ or disorder$ or syndrome$)).ti,ab,kf. (182)

24 (schilder$ adj6 (diffuse cerebral or diffuse degeneration or poliodystroph$)).ti,ab,kf. (5)

25 (balo$ adj3 concentric scleros$).ti,ab,kf. (191)

26 (progressive adj3 poliodystroph$).ti,ab,kf. (18)

27 (diffuse cerebral degeneration$ or diffuse cerebral scleros$ or encephalitis periaxialis or myelinoclastic diffuse scleros$ or poliodystrophia cerebri or progressive neuronal degeneration$ or sudanophilic cerebral scleros$).ti,ab,kf. (337)

28 ahs.ti,ab,kf. (1684)

29 or/20-28 (4386)

30 myocerebrohepatopath$.ti,ab,kf. (13)

31 mchs.ti,ab,kf. (100)

32 or/30-31 (106)

33 Spinocerebellar Ataxias/ and exp Epilepsy/ (68)

34 (myoclon$ and epilep$ and sensory ataxia$).ti,ab,kf. (11)

35 memsa.ti,ab,kf. (14)

36 (spinocerebellar ataxia$ and epilep$).ti,ab,kf. (181)

37 scae.ti,ab,kf. (58)

38 or/33-37 (292)

39 Ophthalmoplegia, Chronic Progressive External/ (606)

40 external ophthalmoplegi$.ti,ab,kf. (1713)

41 ocular muscular dystroph$.ti,ab,kf. (10)

42 (peo or cpeo$ or arpeo or adpeo).ti,ab,kf. (6436)

43 ((graefe$ or graefe fuch$) adj6 (disease$ or disorder$ or syndrome$)).ti,ab,kf. (11)

44 ((graefe$ or graefe fuch$) adj6 myopath$).ti,ab,kf. (2)

45 or/39-44 (7811)

46 ataxia neuropath$.ti,ab,kf. (146)

47 ataxic neuropath$.ti,ab,kf. (261)

48 miras.ti,ab,kf. (134)

49 sando.ti,ab,kf. (73)

50 or/46-49 (572)

51 MELAS Syndrome/ (1440)

52 melas.ti,ab,kf. (2239)

53 myoencephalopath$.ti,ab,kf. (10)

54 or/51-53 (2566)

55 MERRF Syndrome/ (390)

56 merrf.ti,ab,kf. (537)

57 merff.ti,ab,kf. (13)

58 fukuhara$.ti,ab,kf. (42)

59 (epilep$ and ragged red).ti,ab,kf. (504)

60 or/55-59 (838)

61 Arginine-tRNA Ligase/ (201)

62 (mitochondri$ and arg$ and (tRNA or RNA or ribonucleic acid)).ti,ab,kf. (593)

63 (rars2 or rars-2).ti,ab,kf. (56)

64 (rarstwo or rars-two or rarsii or rars-ii or rars11 or rars-11).ti,ab,kf. (5)

65 or/61-64 (808)

66 Olivopontocerebellar Atrophies/ or (pontocerebellar adj6 hypoplasia$).ti,ab,kf. (940)

67 (pontocerebellum adj6 hypoplasia$).ti,ab,kf. (0)

68 (pch6 or pch-6).ti,ab,kf. (39)

69 (pchsix or pch-six or pchvi or pch-vi).ti,ab,kf. (1)

70 or/66-69 (958)

71 Pyruvate Dehydrogenase Complex Deficiency Disease/ (415)

72 (pyruvate dehydrogenase adj6 deficien$).ti,ab,kf. (522)

73 ((pdhc or pdh or pyruvate decarboxylase) adj6 deficien$).ti,ab,kf. (248)

74 ((ataxia or ataxic) adj6 (lactic acidosis or pyruvate or decarboxylase)).ti,ab,kf. (119)

75 or/71-74 (831)

76 7 or 13 or 19 or 29 or 32 or 38 or 45 or 50 or 54 or 60 or 65 or 70 or 75 (73057)

77 Economics/ (27457)

78 exp "costs and cost analysis"/ (259838)

79 Economics, Dental/ (1920)

80 exp economics, hospital/ (25616)

81 Economics, Medical/ (9229)

82 Economics, Nursing/ (4013)

83 Economics, Pharmaceutical/ (3077)

84 (economic$ or cost or costs or costly or costing or price or prices or pricing or pharmacoeconomic$).ti,ab. (964965)

85 (expenditure$ not energy).ti,ab. (34826)

86 value for money.ti,ab. (1999)

87 budget$.ti,ab. (33632)

88 or/77-87 (1126780)

89 ((energy or oxygen) adj cost).ti,ab. (4586)

90 (metabolic adj cost).ti,ab. (1622)

91 ((energy or oxygen) adj expenditure).ti,ab. (27954)

92 or/89-91 (33122)

93 88 not 92 (1119153)

94 exp Budgets/ (14042)

95 exp models, economic/ (16135)

96 "Value of Life"/ (5794)

97 ec.fs. (442237)

98 Income/ (33346)

99 Remuneration/ (345)

100 "Salaries and Fringe Benefits"/ (16176)

101 exp "Fees and Charges"/ (31185)

102 (earn$ or expens$ or fee or fees or financ$ or fiscal$ or income$1 or money$ or monetary or paid or pay or pays or paying or payment$1 or remunerat$ or salar$ or wage$1).ti,ab,kf. (585663)

103 or/94-102 (946825)

104 93 or 103 (1688243)

105 Health Resources/ or "Supply & Distribution".fs. or exp Resource Allocation/ (100032)

106 (burden$ or resource$1).ti. (90306)

107 (burden$ adj6 (care or caring or disease$ or healthcare or illness$ or sickness$ or therap$ or treatment$)).ti,ab,kf. (87926)

108 (((resource$1 or healthcare or health-care) adj6 (allocat$ or consum$ or ration$ or usage$ or use$1 or utilis$ or utiliz$)) or hcru).ti,ab,kf. (163800)

109 Office Visits/sn, td or "Facilities and Services Utilization"/ or "Equipment and Supplies Utilization"/ or "Procedures and Techniques Utilization"/ (5292)

110 (visit or visits or visited or visiting).ti,ab,kf. (270156)

111 appointment$.ti,ab,kf. (29961)

112 Hospitalization/ (129601)

113 (hospitalization$1 or hospitalisation$1 or hospitalised or hospitalized).ti,ab,kf. (307527)

114 (admission$1 or readmission$1 or admitted or readmitted).ti,ab,kf. (468932)

115 "length of stay"/ (100209)

116 hospital stay$1.ti,ab,kf. (99943)

117 (bed adj3 day$1).ti,ab,kf. (4014)

118 ((days or time or length or duration$1) adj3 hospital$).ti,ab,kf. (111854)

119 ((days or time or length or duration$1) adj3 (stay or stays or stayed)).ti,ab,kf. (125981)

120 ((days or time or length or duration$1) adj3 (discharge or discharged or home or homes)).ti,ab,kf. (29378)

121 or/105-120 (1464376)

122 76 and 104 (1219)

123 76 and 121 (1312)

124 122 or 123 (2404)

125 exp Animals/ not Humans/ (5040469)

126 124 not 125 (2189)

127 limit 126 to english language (1913)

1. **Source: Embase**

Interface / URL: OvidSP

Database coverage dates: 1974 to 2022 July 26

Search date: 27/07/2022

Retrieved records: 3143

Search strategy:

1 "disorders of mitochondrial functions"/ (25430)

2 mitochondrial dna disorder/ or mitochondrial encephalomyopathy/ or mitochondrial encephalopathy/ or mitochondrial myopathy/ (5107)

3 (mitochondri$ adj6 (disease$ or disorder$ or syndrome$)).ti,ab,kw,dq. (24727)

4 (mitochondri$ adj6 (cytopath$ or deficien$ or dysfunction$ or encephalomyopath$ or encephalopath$ or myopath$)).ti,ab,kw,dq. (52611)

5 (mitochondriopath$ or (mdas not modified dental anxiety scale)).ti,ab,kw,dq. (1365)

6 (mitochondri$ adj6 (epilep$ or seizure$ or convuls$)).ti,ab,kw,dq. (1014)

7 or/1-6 (76616)

8 Leigh disease/ (2851)

9 ((leigh$ or leigh-feigin$ or leigh-feigin-wolf$) adj6 (disease$ or disorder$ or syndrome$)).ti,ab,kw,dq. (2333)

10 ((leigh$ or leigh-feigin$ or leigh-feigin-wolf$) adj6 (encephalopath$ or encephalomyelopath$)).ti,ab,kw,dq. (395)

11 ((subacute or sub-acute) adj3 (necrot$ encephal$ or necrot$ juvenile encephal$ or necrot$ infantile encephal$)).ti,ab,kw,dq. (254)

12 (leigh$ and mitochondri$).ti,ab,kw,dq. (1889)

13 or/8-12 (3433)

14 DNA directed DNA polymerase gamma/ (2328)

15 (polymerase$ adj3 gamma).ti,ab,kw,dq. (1611)

16 (polg or polg1).ti,ab,kw,dq. (1647)

17 (polgone or polgi).ti,ab,kw,dq. (4)

18 (mitochondri$ adj6 polymerase$).ti,ab,kw,dq. (2327)

19 or/14-18 (5490)

20 Alpers disease/ or Schilder disease/ (727)

21 ((alper$ or alper-huttenlocher$ or alpers-huttenlocher$) adj6 (disease$ or disorder$ or syndrome$)).ti,ab,kw,dq. (357)

22 ((alper$ or alper-huttenlocher$ or alpers-huttenlocher$) adj6 (diffuse cerebral or diffuse degeneration or poliodystroph$)).ti,ab,kw,dq. (18)

23 (schilder$ adj6 (disease$ or disorder$ or syndrome$)).ti,ab,kw,dq. (168)

24 (schilder$ adj6 (diffuse cerebral or diffuse degeneration or poliodystroph$)).ti,ab,kw,dq. (8)

25 (balo$ adj3 concentric scleros$).ti,ab,kw,dq. (275)

26 (progressive adj3 poliodystroph$).ti,ab,kw,dq. (12)

27 (diffuse cerebral degeneration$ or diffuse cerebral scleros$ or encephalitis periaxialis or myelinoclastic diffuse scleros$ or poliodystrophia cerebri or progressive neuronal degeneration$ or sudanophilic cerebral scleros$).ti,ab,kw,dq. (231)

28 ahs.ti,ab,kw,dq. (2308)

29 or/20-28 (3578)

30 myocerebrohepatopath$.ti,ab,kw,dq. (25)

31 mchs.ti,ab,kw,dq. (131)

32 or/30-31 (148)

33 spinocerebellar degeneration/ and exp epilepsy/ (518)

34 (myoclon$ and epilep$ and sensory ataxia$).ti,ab,kw,dq. (24)

35 memsa.ti,ab,kw,dq. (19)

36 (spinocerebellar ataxia$ and epilep$).ti,ab,kw,dq. (287)

37 scae.ti,ab,kw,dq. (88)

38 or/33-37 (763)

39 chronic progressive external ophthalmoplegia/ (1062)

40 external ophthalmoplegi$.ti,ab,kw,dq. (2136)

41 ocular muscular dystroph$.ti,ab,kw,dq. (6)

42 (peo or cpeo$ or arpeo or adpeo).ti,ab,kw,dq. (5915)

43 ((graefe$ or graefe fuch$) adj6 (disease$ or disorder$ or syndrome$)).ti,ab,kw,dq. (12)

44 ((graefe$ or graefe fuch$) adj6 myopath$).ti,ab,kw,dq. (3)

45 or/39-44 (7749)

46 ataxia neuropath$.ti,ab,kw,dq. (258)

47 ataxic neuropath$.ti,ab,kw,dq. (400)

48 miras.ti,ab,kw,dq. (131)

49 sando.ti,ab,kw,dq. (119)

50 or/46-49 (831)

51 MELAS syndrome/ (3105)

52 melas.ti,ab,kw,dq. (3021)

53 myoencephalopath$.ti,ab,kw,dq. (12)

54 or/51-53 (4140)

55 MERRF syndrome/ (751)

56 merrf.ti,ab,kw,dq. (706)

57 merff.ti,ab,kw,dq. (36)

58 fukuhara$.ti,ab,kw,dq. (72)

59 (epilep$ and ragged red).ti,ab,kw,dq. (624)

60 or/55-59 (1316)

61 arginine transfer RNA ligase/ (236)

62 (mitochondri$ and arg$ and (tRNA or RNA or ribonucleic acid)).ti,ab,kw,dq. (687)

63 (rars2 or rars-2).ti,ab,kw,dq. (136)

64 (rarstwo or rars-two or rarsii or rars-ii or rars11 or rars-11).ti,ab,kw,dq. (7)

65 or/61-64 (999)

66 cerebellum hypoplasia/ or olivopontocerebellar atrophy/ or (pontocerebellar adj6 hypoplasia$).ti,ab,kw,dq. (3484)

67 (pontocerebellum adj6 hypoplasia$).ti,ab,kw,dq. (5)

68 (pch6 or pch-6).ti,ab,kw,dq. (46)

69 (pchsix or pch-six or pchvi or pch-vi).ti,ab,kw,dq. (1)

70 or/66-69 (3500)

71 pyruvate dehydrogenase complex deficiency/ (489)

72 (pyruvate dehydrogenase adj6 deficien$).ti,ab,kw,dq. (696)

73 ((pdhc or pdh or pyruvate decarboxylase) adj6 deficien$).ti,ab,kw,dq. (349)

74 ((ataxia or ataxic) adj6 (lactic acidosis or pyruvate or decarboxylase)).ti,ab,kw,dq. (152)

75 or/71-74 (1122)

76 7 or 13 or 19 or 29 or 32 or 38 or 45 or 50 or 54 or 60 or 65 or 70 or 75 (99198)

77 Health Economics/ (34491)

78 exp Economic Evaluation/ (336797)

79 exp Health Care Cost/ (321749)

80 pharmacoeconomics/ (8856)

81 (econom$ or cost or costs or costly or costing or price or prices or pricing or pharmacoeconomic$).ti,ab. (1270542)

82 (expenditure$ not energy).ti,ab. (46828)

83 (value adj2 money).ti,ab. (2776)

84 budget$.ti,ab. (44000)

85 or/77-84 (1550852)

86 (metabolic adj cost).ti,ab. (1735)

87 ((energy or oxygen) adj cost).ti,ab. (4816)

88 ((energy or oxygen) adj expenditure).ti,ab. (35254)

89 or/86-88 (40635)

90 85 not 89 (1542514)

91 economics/ (243873)

92 cost/ (61049)

93 budget/ (31898)

94 device economics/ (68)

95 exp economic model/ (3161)

96 socioeconomics/ (153825)

97 (pe or de).fs. (88860)

98 income/ (65674)

99 remuneration/ (1802)

100 "salary and fringe benefit"/ or salary/ (20460)

101 exp fee/ (42403)

102 (expens$ or earning$ or salar$ or wage$1 or pay or pays or paid or paying or payment$1 or income$1 or remunerat$ or financ$ or money or monetary or fee or fees or charg$).ti,ab,kw,dq. (1003965)

103 or/91-102 (1466163)

104 90 or 103 (2537926)

105 exp health care utilization/ (85795)

106 drug utilization/ (22031)

107 exp "utilization review"/ (68938)

108 health care planning/ or resource management/ or resource allocation/ (136769)

109 disease burden/ (35293)

110 hospital utilization/ (2582)

111 hospital bed utilization/ (4056)

112 (burden$ or resource$1).ti. (119991)

113 (burden$ adj6 (care or caring or disease$ or healthcare or illness$ or sickness$ or therap$ or treatment$)).ti,ab,kw,dq. (132433)

114 (((resource$1 or healthcare or health-care) adj6 (allocat$ or consum$ or ration$ or usage$ or use$1 or utilis$ or utiliz$)) or hcru).ti,ab,kw,dq. (221299)

115 (visit or visits or visited or visiting).ti,ab,kw,dq. (437651)

116 appointment$.ti,ab,kw,dq. (52605)

117 hospitalization/ or child hospitalization/ or hospital admission/ (684626)

118 (hospitalization$1 or hospitalisation$1 or hospitalised or hospitalized).ti,ab,kw,dq. (494841)

119 (admission$1 or readmission$1 or admitted or readmitted).ti,ab,kw,dq. (798490)

120 "length of stay"/ (239440)

121 hospital stay$1.ti,ab,kw,dq. (163882)

122 (bed adj3 day$1).ti,ab,kw,dq. (6628)

123 ((days or time or length or duration$1) adj3 hospital$).ti,ab,kw,dq. (186699)

124 ((days or time or length or duration$1) adj3 (stay or stays or stayed)).ti,ab,kw,dq. (221464)

125 ((days or time or length or duration$1) adj3 (discharge or discharged or home or homes)).ti,ab,kw,dq. (52818)

126 or/105-125 (2527703)

127 76 and 104 (2753)

128 76 and 126 (3012)

129 127 or 128 (5501)

130 (animal/ or animal experiment/ or animal model/ or animal tissue/ or nonhuman/) not exp human/ (6501033)

131 conference abstract.pt. (4464216)

132 129 not (130 or 131) (3408)

133 limit 132 to english language (3143)

1. **Source: Cochrane Database of Systematic Reviews (CDSR)**

Interface / URL: Cochrane Library / Wiley

Database coverage dates: Information not found. Issue searched: Issue 7 of 12, July 2022

Search date: 26/07/2022

Retrieved records: 8

Search strategy:

#1 MeSH descriptor: [Mitochondrial Diseases] this term only 56

#2 MeSH descriptor: [Mitochondrial Myopathies] this term only 34

#3 MeSH descriptor: [Mitochondrial Encephalomyopathies] this term only 10

#4 (mitochondri* NEAR/6 (disease* OR disorder* OR syndrome*)):ti,ab,kw 358

#5 (mitochondri* NEAR/6 (cytopath* OR deficien* OR dysfunction* OR encephalomyopath* OR encephalopath* OR myopath*)):ti,ab,kw 522

#6 (mitochondriopath* OR (mdas not "modified dental anxiety scale")):ti,ab,kw 91

#7 (mitochondri* NEAR/6 (epilep* OR seizure* OR convuls*)):ti,ab,kw 10

#8 #1 OR #2 OR #3 OR #4 OR #5 OR #6 OR #7 831

#9 MeSH descriptor: [Leigh Disease] this term only 4

#10 ((leigh* OR leigh-feigin* OR leigh-feigin-wolf*) NEAR/6 (disease* OR disorder* OR syndrome*)):ti,ab,kw 11

#11 ((leigh* OR leigh-feigin* OR leigh-feigin-wolf*) NEAR/6 (encephalopath* OR encephalomyelopath*)):ti,ab,kw 0

#12 ((subacute OR sub-acute) NEAR/3 (necrot* NEXT encephal* OR necrot* NEXT juvenile NEXT encephal* OR necrot* NEXT infantile NEXT encephal*)):ti,ab,kw 0

#13 (leigh* AND mitochondri*):ti,ab,kw 8

#14 #9 OR #10 OR #11 OR #12 OR #13 11

#15 MeSH descriptor: [DNA Polymerase gamma] this term only 1

#16 (polymerase* NEAR/3 gamma):ti,ab,kw 12

#17 (polg OR polg1):ti,ab,kw 5

#18 (polgone OR polgi):ti,ab,kw 0

#19 (mitochondri* NEAR/6 polymerase*):ti,ab,kw 21

#20 #15 or #16 or #17 or #18 or #19 26

#21 MeSH descriptor: [Diffuse Cerebral Sclerosis of Schilder] this term only 7

#22 ((alper* OR alper-huttenlocher* OR alpers-huttenlocher*) NEAR/6 (disease* OR disorder* OR syndrome*)):ti,ab,kw 3

#23 ((alper* OR alper-huttenlocher* OR alpers-huttenlocher*) NEAR/6 ("diffuse cerebral" OR "diffuse degeneration" OR poliodystroph*)):ti,ab,kw 0

#24 (schilder* NEAR/6 (disease* OR disorder* OR syndrome*)):ti,ab,kw 1

#25 (schilder* NEAR/6 ("diffuse cerebral" OR "diffuse degeneration" OR poliodystroph*)):ti,ab,kw 7

#26 (balo* NEAR/3 (concentric NEXT scleros*)):ti,ab,kw 1

#27 (progressive NEAR/3 poliodystroph*):ti,ab,kw 0

#28 (diffuse NEXT cerebral NEXT degeneration* OR diffuse NEXT cerebral NEXT scleros* OR "encephalitis periaxialis" OR myelinoclastic NEXT diffuse NEXT scleros* OR "poliodystrophia cerebri" OR progressive NEXT neuronal NEXT degeneration* OR sudanophilic NEXT cerebral NEXT scleros*):ti,ab,kw 10

#29 ahs:ti,ab,kw 112

#30 #21 OR #22 OR #23 OR #24 OR #25 OR #26 OR #27 OR #28 OR #29 127

#31 myocerebrohepatopath*:ti,ab,kw 0

#32 mchs:ti,ab,kw 4

#33 #31 OR #32 4

#34 MeSH descriptor: [Spinocerebellar Ataxias] this term only 59

#35 MeSH descriptor: [Epilepsy] explode all trees 2597

#36 #34 AND #35 0

#37 (myoclon* AND epilep* AND sensory NEXT ataxia*):ti,ab,kw 0

#38 memsa:ti,ab,kw 8

#39 (spinocerebellar NEXT ataxia* AND epilep*):ti,ab,kw 1

#40 scae:ti,ab,kw 5

#41 #36 OR #37 OR #38 OR #39 OR #40 14

#42 MeSH descriptor: [Ophthalmoplegia, Chronic Progressive External] this term only 6

#43 external NEXT ophthalmoplegi*:ti,ab,kw 17

#44 ocular NEXT muscular NEXT dystroph*:ti,ab,kw 0

#45 (peo OR cpeo* OR arpeo OR adpeo):ti,ab,kw 35

#46 ((graefe* OR graefe NEXT fuch*) NEAR/6 (disease* OR disorder* OR syndrome*)):ti,ab,kw 1

#47 ((graefe* OR graefe NEXT fuch*) NEAR/6 myopath*):ti,ab,kw 0

#48 #42 OR #43 OR #44 OR #45 OR #46 OR #47 48

#49 ataxia NEXT neuropath*:ti,ab,kw 2

#50 ataxic NEXT neuropath*:ti,ab,kw 8

#51 miras:ti,ab,kw 11

#52 sando:ti,ab,kw 1

#53 #49 OR #50 OR #51 OR #52 22

#54 MeSH descriptor: [MELAS Syndrome] this term only 12

#55 melas:ti,ab,kw 58

#56 myoencephalopath*:ti,ab,kw 0

#57 #54 OR #55 OR #56 58

#58 MeSH descriptor: [MERRF Syndrome] this term only 0

#59 merrf:ti,ab,kw 2

#60 merff:ti,ab,kw 0

#61 fukuhara*:ti,ab,kw 1

#62 (epilep* AND "ragged red"):ti,ab,kw 1

#63 #58 OR #59 OR #60 OR #61 OR #62 4

#64 MeSH descriptor: [Arginine-tRNA Ligase] this term only 0

#65 (mitochondri* AND arg* AND (tRNA OR RNA OR "ribonucleic acid")):ti,ab,kw 4

#66 (rars2 OR rars-2):ti,ab,kw 5

#67 (rarstwo OR rars-two OR rarsii OR rars-ii OR rars11 OR rars-11):ti,ab,kw 0

#68 #64 OR #65 OR #66 OR #67 9

#69 MeSH descriptor: [Olivopontocerebellar Atrophies] this term only 11

#70 (pontocerebellar NEAR/6 hypoplasia*):ti,ab,kw 0

#71 (pontocerebellum NEAR/6 hypoplasia*):ti,ab,kw 0

#72 (pch6 OR pch-6):ti,ab,kw 0

#73 (pchsix OR pch-six OR pchvi OR pch-vi):ti,ab,kw 0

#74 #69 OR #70 OR #71 OR #72 OR #73 11

#75 MeSH descriptor: [Pyruvate Dehydrogenase Complex Deficiency Disease] this term only 4

#76 ("pyruvate dehydrogenase" NEAR/6 deficien*):ti,ab,kw 8

#77 ((pdhc OR pdh OR "pyruvate decarboxylase") NEAR/6 deficien*):ti,ab,kw 3

#78 ((ataxia OR ataxic) NEAR/6 ("lactic acidosis" OR pyruvate OR decarboxylase)):ti,ab,kw 1

#79 #75 OR #76 OR #77 OR #78 12

#80 #8 OR #14 OR #20 OR #30 OR #33 OR #41 OR #48 OR #53 OR #57 OR #63 OR #68 OR #74 OR #79 1111

#81 #80 in Cochrane Reviews, Cochrane Protocols 8

1. **Source: Cochrane Central Register of Controlled Trials (CENTRAL)**

Interface / URL: Cochrane / Wiley

Database coverage dates: Information not found. Issue searched: Issue Issue 7 of 12, July 2022

Search date: 27/07/2022

Retrieved records: 307

Search strategy:

#1 MeSH descriptor: [Mitochondrial Diseases] this term only 56

#2 MeSH descriptor: [Mitochondrial Myopathies] this term only 34

#3 MeSH descriptor: [Mitochondrial Encephalomyopathies] this term only 10

#4 mitochondri* NEAR/6 (disease* OR disorder* OR syndrome*) 434

#5 mitochondri* NEAR/6 (cytopath* OR deficien* OR dysfunction* OR encephalomyopath* OR encephalopath* OR myopath*) 565

#6 (mitochondriopath* OR (mdas not "modified dental anxiety scale")) 109

#7 mitochondri* NEAR/6 (epilep* OR seizure* OR convuls*) 10

#8 #1 OR #2 OR #3 OR #4 OR #5 OR #6 OR #7 943

#9 MeSH descriptor: [Leigh Disease] this term only 4

#10 (leigh* OR leigh-feigin* OR leigh-feigin-wolf*) NEAR/6 (disease* OR disorder* OR syndrome*) 19

#11 (leigh* OR leigh-feigin* OR leigh-feigin-wolf*) NEAR/6 (encephalopath* OR encephalomyelopath*) 1

#12 (subacute OR sub-acute) NEAR/3 (necrot* NEXT encephal* OR necrot* NEXT juvenile NEXT encephal* OR necrot* NEXT infantile NEXT encephal*) 0

#13 leigh* AND mitochondri* 17

#14 #9 OR #10 OR #11 OR #12 OR #13 22

#15 MeSH descriptor: [DNA Polymerase gamma] this term only 1

#16 polymerase* NEAR/3 gamma 25

#17 polg OR polg1 6

#18 polgone OR polgi 0

#19 mitochondri* NEAR/6 polymerase* 29

#20 #15 or #16 or #17 or #18 or #19 45

#21 MeSH descriptor: [Diffuse Cerebral Sclerosis of Schilder] this term only 7

#22 (alper* OR alper-huttenlocher* OR alpers-huttenlocher*) NEAR/6 (disease* OR disorder* OR syndrome*) 4

#23 (alper* OR alper-huttenlocher* OR alpers-huttenlocher*) NEAR/6 ("diffuse cerebral" OR "diffuse degeneration" OR poliodystroph*) 0

#24 schilder* NEAR/6 (disease* OR disorder* OR syndrome*) 8

#25 schilder* NEAR/6 ("diffuse cerebral" OR "diffuse degeneration" OR poliodystroph*) 7

#26 balo* NEAR/3 (concentric NEXT scleros*) 1

#27 progressive NEAR/3 poliodystroph* 0

#28 diffuse NEXT cerebral NEXT degeneration* OR diffuse NEXT cerebral NEXT scleros* OR "encephalitis periaxialis" OR myelinoclastic NEXT diffuse NEXT scleros* OR "poliodystrophia cerebri" OR progressive NEXT neuronal NEXT degeneration* OR sudanophilic NEXT cerebral NEXT scleros* 10

#29 ahs 393

#30 #21 OR #22 OR #23 OR #24 OR #25 OR #26 OR #27 OR #28 OR #29 416

#31 myocerebrohepatopath* 0

#32 mchs 4

#33 #31 OR #32 4

#34 MeSH descriptor: [Spinocerebellar Ataxias] this term only 59

#35 MeSH descriptor: [Epilepsy] explode all trees 2597

#36 #34 AND #35 0

#37 myoclon* AND epilep* AND sensory NEXT ataxia* 0

#38 memsa 30

#39 spinocerebellar NEXT ataxia* AND epilep* 3

#40 scae 5

#41 #36 OR #37 OR #38 OR #39 OR #40 38

#42 MeSH descriptor: [Ophthalmoplegia, Chronic Progressive External] this term only 6

#43 external NEXT ophthalmoplegi* 19

#44 ocular NEXT muscular NEXT dystroph* 0

#45 peo OR cpeo* OR arpeo OR adpeo 48

#46 (graefe* OR graefe NEXT fuch*) NEAR/6 (disease* OR disorder* OR syndrome*) 1

#47 (graefe* OR graefe NEXT fuch*) NEAR/6 myopath* 0

#48 #42 OR #43 OR #44 OR #45 OR #46 OR #47 62

#49 ataxia NEXT neuropath* 3

#50 ataxic NEXT neuropath* 10

#51 miras 92

#52 sando 90

#53 #49 OR #50 OR #51 OR #52 195

#54 MeSH descriptor: [MELAS Syndrome] this term only 12

#55 melas 100

#56 myoencephalopath* 0

#57 #54 OR #55 OR #56 100

#58 MeSH descriptor: [MERRF Syndrome] this term only 0

#59 merrf 4

#60 merff 1

#61 fukuhara* 227

#62 epilep* AND "ragged red" 2

#63 #58 OR #59 OR #60 OR #61 OR #62 232

#64 MeSH descriptor: [Arginine-tRNA Ligase] this term only 0

#65 mitochondri* AND arg* AND (tRNA OR RNA OR "ribonucleic acid") 10

#66 rars2 OR rars-2 5

#67 rarstwo OR rars-two OR rarsii OR rars-ii OR rars11 OR rars-11 0

#68 #64 OR #65 OR #66 OR #67 15

#69 MeSH descriptor: [Olivopontocerebellar Atrophies] this term only 11

#70 pontocerebellar NEAR/6 hypoplasia* 0

#71 pontocerebellum NEAR/6 hypoplasia* 0

#72 pch6 OR pch-6 0

#73 pchsix OR pch-six OR pchvi OR pch-vi 0

#74 #69 OR #70 OR #71 OR #72 OR #73 11

#75 MeSH descriptor: [Pyruvate Dehydrogenase Complex Deficiency Disease] this term only 4

#76 "pyruvate dehydrogenase" NEAR/6 deficien* 11

#77 (pdhc OR pdh OR "pyruvate decarboxylase") NEAR/6 deficien* 3

#78 (ataxia OR ataxic) NEAR/6 ("lactic acidosis" OR pyruvate OR decarboxylase) 1

#79 #75 OR #76 OR #77 OR #78 15

#80 #8 OR #14 OR #20 OR #30 OR #33 OR #41 OR #48 OR #53 OR #57 OR #63 OR #68 OR #74 OR #79 2011

#81 MeSH descriptor: [Economics] this term only 45

#82 MeSH descriptor: [Costs and Cost Analysis] explode all trees 11515

#83 MeSH descriptor: [Economics, Dental] this term only 2

#84 MeSH descriptor: [Economics, Hospital] explode all trees 736

#85 MeSH descriptor: [Economics, Medical] this term only 26

#86 MeSH descriptor: [Economics, Nursing] this term only 13

#87 MeSH descriptor: [Economics, Pharmaceutical] this term only 65

#88 economic* OR cost OR costs OR costly OR costing OR price OR prices OR pricing OR pharmacoeconomic* 100767

#89 expenditure* NOT energy 2457

#90 "value for money" 326

#91 budget* 1614

#92 #81 OR #82 OR #83 OR #84 OR #85 OR #86 OR #87 OR #88 OR #89 OR #90 OR #91 101948

#93 "energy cost" OR "oxygen cost" 513

#94 "metabolic cost" 143

#95 "energy expenditure" OR "oxygen expenditure" 5143

#96 #93 OR #94 OR #95 5647

#97 #92 NOT #96 100983

#98 MeSH descriptor: [Budgets] explode all trees 32

#99 MeSH descriptor: [Models, Economic] explode all trees 371

#100 MeSH descriptor: [Value of Life] this term only 32

#101 MeSH descriptor: [] explode all trees and with qualifier(s): [economics - EC] 12054

#102 MeSH descriptor: [Income] this term only 346

#103 MeSH descriptor: [Remuneration] this term only 11

#104 MeSH descriptor: [Salaries and Fringe Benefits] this term only 53

#105 MeSH descriptor: [Fees and Charges] explode all trees 259

#106 earn* OR expens* OR fee OR fees OR financ* OR fiscal* OR income* OR money* OR monetary OR paid OR pay OR pays OR paying OR payment* OR remunerat* OR salar* OR wage* 46685

#107 #98 OR #99 OR #100 OR #101 OR #102 OR #103 OR #104 OR #105 OR #106 55411

#108 #97 OR #107 128315

#109 MeSH descriptor: [Health Resources] this term only 430

#110 MeSH descriptor: [] explode all trees and with qualifier(s): [supply & distribution - SD] 396

#111 MeSH descriptor: [Resource Allocation] explode all trees 76

#112 (burden* OR resource*):ti 4193

#113 burden* NEAR/6 (care OR caring OR disease* OR healthcare OR illness* OR sickness* OR therap* OR treatment*) 11165

#114 (resource* OR healthcare OR health-care) NEAR/6 (allocat* OR consum* OR ration* OR usage* OR use* OR utilis* OR utiliz*) 21005

#115 HCRU 90

#116 MeSH descriptor: [Office Visits] this term only and with qualifier(s): [statistics & numerical data - SN] 139

#117 MeSH descriptor: [Office Visits] this term only and with qualifier(s): [trends - TD] 4

#118 MeSH descriptor: [Facilities and Services Utilization] this term only 40

#119 MeSH descriptor: [Equipment and Supplies Utilization] this term only 0

#120 MeSH descriptor: [Procedures and Techniques Utilization] this term only 9

#121 visit OR visits OR visited OR visiting 95567

#122 appointment* 9453

#123 MeSH descriptor: [Hospitalization] this term only 5808

#124 hospitalization* OR hospitalisation* OR hospitalised OR hospitalized 65366

#125 admission* OR readmission* OR admitted OR readmitted 61421

#126 MeSH descriptor: [Length of Stay] this term only 7705

#127 hospital stay* 40087

#128 bed NEAR/3 day* 890

#129 (days OR time OR length OR duration*) NEAR/3 hospital* 32596

#130 (days OR time OR length OR duration*) NEAR/3 (stay OR stays OR stayed) 34251

#131 (days OR time OR length OR duration*) NEAR/3 (discharge OR discharged OR home OR homes) 9900

#132 #109 OR #110 OR #111 OR #112 OR #113 OR #114 OR #115 OR #116 OR #117 OR #118 OR #119 OR #120 OR #121 OR #122 OR #123 OR #124 OR #125 OR #126 OR #127 OR #128 OR #129 OR #130 OR #131 248228

#133 #80 AND #108 313

#134 #80 AND #132 462

#135 #133 OR #134 556

#136 #135 in Trials 307

Search note: It was noticed that line 113 in the CENTRAL strategy for the original searches contained a syntax error (*adj* was used for the proximity operator). This was corrected for the update search.

1. **Source: HTA Database**

Interface / URL: https://database.inahta.org/

Database coverage dates: Information not found. The former database was produced by the CRD until March 2018, at which time the addition of records was stopped as INAHTA was in the process of rebuilding the new database platform. In July 2019, the database records were exported from the CRD platform and imported into the new platform that was developed by INAHTA. The rebuild of the new platform was launched in June 2020.

Search date: 26/07/2022

Retrieved records: 27

Search strategy:

54 #53 OR #52 OR #51 OR #50 OR #49 OR #48 OR #47 OR #46 OR #45 OR #44 OR #43 OR #42 OR #41 OR #40 OR #39 OR #38 OR #37 OR #36 OR #35 OR #34 OR #33 OR #32 OR #31 OR #30 OR #29 OR #28 OR #27 OR #26 OR #25 OR #24 OR #23 OR #22 OR #21 OR #20 OR #19 OR #18 OR #17 OR #16 OR #15 OR #14 OR #13 OR #12 OR #11 OR #10 OR #9 OR #8 OR #7 OR #6 OR #5 OR #4 OR #3 OR #2 OR #1 27

53 (ataxia OR ataxic) AND ("lactic acidosis" OR pyruvate OR decarboxylase) 0

52 (pdhc OR pdh OR "pyruvate decarboxylase") AND deficien* 0

51 ("pyruvate dehydrogenase" AND deficien*) 0

50 "Pyruvate Dehydrogenase Complex Deficiency Disease"[mh] 0

49 (pchsix OR "pch-six" OR pchvi OR "pch-vi") 0

48 (pch6 OR "pch-6") 0

47 (pontocerebellum AND hypoplasia*) 0

46 (pontocerebellar AND hypoplasia*) 0

45 "Olivopontocerebellar Atrophies"[mh] 0

44 (rarstwo OR "rars-two" OR rarsii OR "rars-ii" OR rars11 OR "rars-11") 0

43 (rars2 OR "rars-2") 0

42 "Arginine-tRNA Ligase"[mh] 0

41 epilep* AND "ragged red" 0

40 fukuhara* 0

39 merff 0

38 merrf 0

37 "MERRF Syndrome"[mh] 0

36 myoencephalopath* 0

35 melas 0

34 "MELAS Syndrome"[mh] 0

33 sando 0

32 miras 1

31 ataxic AND neuropath* 0

30 ataxia AND neuropath* 4

29 graefe* 0

28 (peo OR cpeo* OR arpeo OR adpeo) 1

27 "ocular muscular" 0

26 ophthalmoplegi* 2

25 "Ophthalmoplegia, Chronic Progressive External"[mh] 0

24 scae 0

23 spinocerebellar AND ataxia* AND epilep* 1

22 memsa 1

21 myoclon* AND epilep* AND ataxia* 3

20 "Spinocerebellar Ataxias"[mh] AND "Epilepsy"[mhe] 0

19 mchs 1

18 myocerebrohepatopath* 1

17 ahs OR mdas 1

16 ("diffuse cerebral" OR "encephalitis periaxialis" OR "myelinoclastic diffuse" OR "poliodystrophia cerebri" OR "progressive neuronal" OR "sudanophilic cerebral") 0

15 poliodystroph* 0

14 balo* AND concentric 0

13 schilder* 4

12 alper* 1

11 "Diffuse Cerebral Sclerosis of Schilder"[mh] 0

10 (polgone OR polgi) 0

9 (polg OR polg1) 1

8 (polymerase* AND gamma) 1

7 "DNA Polymerase gamma"[mh] 0

6 (subacute OR "sub-acute") AND encephal* 0

5 leigh* 5

4 "Leigh Disease"[mh] 0

3 mitochondri* 11

2 "Mitochondrial Myopathies"[mh] OR "Mitochondrial Encephalomyopathies"[mh] 1

1 "Mitochondrial Diseases"[mh] 1

1. **Source: Cost-Effectiveness Analysis Registry**

Interface / URL: https://cevr.tuftsmedicalcenter.org/databases/cea-registry

Database coverage dates: The webpage at the URL states that the database includes studies published from 1976 to the present

Search date: 26/07/2022

Retrieved records: 0

Search strategy:

The advanced search interface at the following URL was used: <https://cear.tuftsmedicalcenter.org/>

The following searches were conducted separately. Search settings were left at default ('Methods' was left selected, drop-down search options were left at 'Keyword' 'Is'. Where AND or OR is shown, this indicates that Boolean AND / OR was selected using the interface '+AND' / '+OR' options.

Returned results were screened by the information specialist for potential relevance to the populations of interest. Potentially relevant results were cross-checked against results from other search sources to see if already retrieved. Any potentially relevant results not identified via another source were retrieved for further assessment.

mitochondrial OR mitochondria OR mitochondrias OR mitochondriopathy OR mitochondriopathies OR mitochondriopathic OR mdas = 0 (1 result returned, excluded)

leigh OR leighs OR leigh's = 0 (3 results returned, excluded)

necrotizing OR necrotising OR necrotic = 0 (6 results returned, excluded)

polymerase AND gamma = 0 (0 results returned)

polymerases OR polg OR polg1 OR polgone OR polgi = 0 (0 results returned)

alper OR alpers OR alper's = 0 (0 results returned)

schilder OR schilders OR schilder's = 0 (0 results returned)

concentric OR poliodystrophy OR poliodystrophies OR poliodystrophia OR poliodystrophias OR poliodystrophic = 0 (0 results returned)

diffuse AND cerebral = 0 (1 result returned, excluded)

encephalitis AND periaxialis = 0 (0 results returned)

myelinoclastic OR sudanophilic = 0 (0 results returned)

progressive AND neuronal = 0 (0 results returned)

ahs = 0 (1 result returned, excluded)

myocerebrohepatopathy OR myocerebrohepatopathies OR myocerebrohepatopathic OR mchs = 0 (0 results returned)

sensory OR memsa OR spinocerebellar OR scae OR ophthalmoplegia OR ophthalmoplegias OR ophthalmoplegic = 0 (3 results returned, excluded)

ocular AND muscular = 0 (0 results returned)

peo OR cpeo OR arpeo OR adpeo = 0 (0 results returned)

graefe OR graefes OR graefe's = 0 (0 results returned)

ataxia AND neuropathy = 0 (0 results returned)

ataxia AND neuropathies = 0 (0 results returned)

ataxia AND neuropathic = 0 (0 results returned)

ataxic = 0 (0 results returned)

miras OR sando OR melas = 0 (1 result returned, excluded)

myoencephalopathy OR myoencephalopathies OR myoencephalopathic = 0 (0 results returned)

merrf OR merff = 0 (0 results returned)

fukuhara OR fukuharas OR fukuhara's = 0 (0 results returned)

ragged = 0 (0 results returned)

rars2 OR rars OR rarstwo OR rarsii OR rars11 = 0 (0 results returned)

pontocerebellar OR pontocerebellum = 0 (0 results returned)

pch6 OR pch OR pchsix OR pchvi OR pyruvate OR pdhc OR pdh OR decarboxylase = 0 (2 results returned, excluded)

lactic AND acidosis = 0 (0 results returned)

Search note: The CEA Registry search interface had been updated since the date of the original searches. As a result, the search terms used for this search differ from the original search.

1. **Source: NHS Economic Evaluation Database (NHS EED)**

Interface / URL: https://www.crd.york.ac.uk/CRDWeb

Database coverage dates: Information not found. Bibliographic records were published on NHS EED until 31st March 2015. Searches of MEDLINE, Embase, CINAHL, PsycINFO and PubMed were continued until the end of the 2014.

Search date: na – no search conducted – see below

Retrieved records: na

Search strategy: na

Search note:

Although it can still be accessed, NHS EED is a closed database. Records were last added to NHS EED in 2015. This means that no new records will have been added since the date of the original search (13/08/2021). No search of NHS EED was therefore required for this update.

1. **Source: Econlit**

Interface / URL: OvidSP

Database coverage dates: 1886 to July 14, 2022

Search date: 26/07/2022

Retrieved records: 99

Search strategy:

1 (mitochondri$ adj6 (disease$ or disorder$ or syndrome$)).af. (0)

2 (mitochondri$ adj6 (cytopath$ or deficien$ or dysfunction$ or encephalomyopath$ or encephalopath$ or myopath$)).af. (0)

3 (mitochondriopath$ or (mdas not modified dental anxiety scale)).af. (13)

4 (mitochondri$ adj6 (epilep$ or seizure$ or convuls$)).af. (0)

5 or/1-4 (13)

6 ((leigh$ or leigh-feigin$ or leigh-feigin-wolf$) adj6 (disease$ or disorder$ or syndrome$)).af. (0)

7 ((leigh$ or leigh-feigin$ or leigh-feigin-wolf$) adj6 (encephalopath$ or encephalomyelopath$)).af. (0)

8 ((subacute or sub-acute) adj3 (necrot$ encephal$ or necrot$ juvenile encephal$ or necrot$ infantile encephal$)).af. (0)

9 (leigh$ and mitochondri$).af. (0)

10 or/6-9 (0)

11 (polymerase$ adj3 gamma).af. (0)

12 (polg or polg1).af. (0)

13 (polgone or polgi).af. (0)

14 (mitochondri$ adj6 polymerase$).af. (0)

15 or/11-14 (0)

16 ((alper$ or alper-huttenlocher$ or alpers-huttenlocher$) adj6 (disease$ or disorder$ or syndrome$)).af. (1)

17 ((alper$ or alper-huttenlocher$ or alpers-huttenlocher$) adj6 (diffuse cerebral or diffuse degeneration or poliodystroph$)).af. (0)

18 (schilder$ adj6 (disease$ or disorder$ or syndrome$)).af. (0)

19 (schilder$ adj6 (diffuse cerebral or diffuse degeneration or poliodystroph$)).af. (0)

20 (balo$ adj3 concentric scleros$).af. (0)

21 (progressive adj3 poliodystroph$).af. (0)

22 (diffuse cerebral degeneration$ or diffuse cerebral scleros$ or encephalitis periaxialis or myelinoclastic diffuse scleros$ or poliodystrophia cerebri or progressive neuronal degeneration$ or sudanophilic cerebral scleros$).af. (0)

23 ahs.af. (53)

24 or/16-23 (54)

25 myocerebrohepatopath$.af. (0)

26 mchs.af. (0)

27 25 or 26 (0)

28 (myoclon$ and epilep$ and sensory ataxia$).af. (0)

29 memsa.af. (0)

30 (spinocerebellar ataxia$ and epilep$).af. (0)

31 scae.af. (0)

32 or/28-31 (0)

33 external ophthalmoplegi$.af. (0)

34 ocular muscular dystroph$.af. (0)

35 (peo or cpeo$ or arpeo or adpeo).af. (9)

36 ((graefe$ or graefe fuch$) adj6 (disease$ or disorder$ or syndrome$)).af. (0)

37 ((graefe$ or graefe fuch$) adj6 myopath$).af. (0)

38 or/33-37 (9)

39 ataxia neuropath$.af. (0)

40 ataxic neuropath$.af. (0)

41 miras.af. (10)

42 sando.af. (0)

43 or/39-42 (10)

44 melas.af. (3)

45 myoencephalopath$.af. (0)

46 44 or 45 (3)

47 merrf.af. (0)

48 merff.af. (0)

49 fukuhara$.af. (10)

50 (epilep$ and ragged red).af. (0)

51 or/47-50 (10)

52 (mitochondri$ and arg$ and (tRNA or RNA or ribonucleic acid)).af. (0)

53 (rars2 or rars-2).af. (0)

54 (rarstwo or rars-two or rarsii or rars-ii or rars11 or rars-11).af. (0)

55 or/52-54 (0)

56 (pontocerebellar adj6 hypoplasia$).af. (0)

57 (pontocerebellum adj6 hypoplasia$).af. (0)

58 (pch6 or pch-6).af. (0)

59 (pchsix or pch-six or pchvi or pch-vi).af. (0)

60 or/56-59 (0)

61 (pyruvate dehydrogenase adj6 deficien$).af. (0)

62 ((pdhc or pdh or pyruvate decarboxylase) adj6 deficien$).af. (0)

63 ((ataxia or ataxic) adj6 (lactic acidosis or pyruvate or decarboxylase)).af. (0)

64 or/61-63 (0)

65 5 or 10 or 15 or 24 or 27 or 32 or 38 or 43 or 46 or 51 or 55 or 60 or 64 (99)

1. **Source: Paediatric Economic Database Evaluation**

Interface / URL: http://pede.ccb.sickkids.ca/pede/

Database coverage dates: The information at the following URL states that the database contains records for studies published from January 1, 1980 to December 31, 2020 - http://pede.ccb.sickkids.ca/pede/database.jsp

Search date: 26/07/2022

Retrieved records: 0

Search strategy:

The basic search interface at the following URL was used: http://pede.ccb.sickkids.ca/pede/search.jsp. All search settings were left as default. In the default setting, terms are searched across the 'Title, abstract, or Keywords'.

The basic search interface allows the searcher to enter up to three terms combined with AND using the separate search line functionality built into the interface. Where AND is shown between terms in the syntax below, this indicates that this functionality was used. All other terms were searched for individually and not combined with any other term.

Separate searches were conducted on each of the following terms. Returned results were screened by the information specialist for potential relevance to the populations of interest. Potentially relevant results were cross-checked against results from other search sources to see if already retrieved. Any potentially relevant results not identified via another source were retrieved for further assessment.

mitochondri = 0 (0 results returned)

mdas = 0 (0 results returned)

leigh = 0 (0 results returned)

subacute = 0 (0 results returned)

sub-acute = 0 (0 results returned)

polymerase AND gamma = 0 (0 results returned)

polg = 0 (0 results returned)

alper = 0 (0 results returned)

schilder = 0 (0 results returned)

concentric = 0 (0 results returned)

poliodystroph = 0 (0 results returned)

diffuse cerebral = 0 (0 results returned)

encephalitis periaxialis = 0 (0 results returned)

myelinoclastic diffuse = 0 (0 results returned)

progressive neuronal = 0 (0 results returned)

sudanophilic cerebral = 0 (0 results returned)

ahs = 0 (2 results returned, excluded)

myocerebrohepatopath = 0 (0 results returned)

mchs = 0 (0 results returned)

sensory ataxia = 0 (0 results returned)

memsa = 0 (0 results returned)

spinocerebellar ataxia = 0 (0 results returned)

scae = 0 (0 results returned)

external ophthalmoplegi = 0 (0 results returned)

ocular muscular dystroph = 0 (0 results returned)

peo = 0 (102 results returned, excluded)

cpeo = 0 (0 results returned)

arpeo = 0 (0 results returned)

adpeo = 0 (0 results returned)

graefe = 0 (0 results returned)

ataxia neuropath = 0 (0 results returned)

ataxic neuropath = 0 (0 results returned)

miras = 0 (0 result returned)

sando = 0 (1 result returned, excluded)

melas = 0 (0 results returned)

myoencephalopath = 0 (0 results returned)

merrf = 0 (0 results returned)

merff = 0 (0 results returned)

fukuhara = 0 (0 results returned)

ragged red = 0 (0 results returned)

rars = 0 (0 results returned)

pontocerebellar = 0 (0 results returned)

pontocerebellum = 0 (0 results returned)

pch = 0 (1 result returned, excluded)

pdh = 0 (0 results returned)

pyruvate = 0 (0 results returned)

lactic acidosis = 0 (0 results returned)

decarboxylase = 0 (0 results returned)

1. **Source: National Institute for Health and Care Excellence (NICE) webpages**

Interface / URL: https://www.nice.org.uk/

Database coverage dates: n/a

Search date: 26/07/2022

Retrieved records: 0

Search strategy:

The following documents were sought: Company Submissions, Final Appraisal Determination Documents, Assessment Reports for multiple technology appraisals, Evidence Review Group (ERG) reports for single technology appraisals – published or updated since the date of original search (13/08/2021).

The site-wide search interface was used at: https://www.nice.org.uk/. Separate searches were conducted on each term shown below.

Results were filtered by 'Document Type' to 'Guidance', then by 'Guidance Programme' to 'Technology appraisal guidance' (if these were available as options).

Results were scanned to identify published technology appraisals on the eligible mitochondrial diseases. For any identified, associated documents under the 'History' tab were viewed to check for relevant documents. PDFs for relevant documents were retrieved for further assessment. Duplicate documents were not retrieved.

mitochondri* = 0 (1 technology appraisal result returned, excluded)

mdas = 0 (0 results returned)

leigh* = 0 (0 technology appraisal results returned)

subacute = 0 (1 technology appraisal result returned, excluded)

"sub-acute" = 0 (1 technology appraisal result returned, excluded)

polymerase* AND gamma = 0 (0 technology appraisal results returned)

polg* = 0 (0 results returned)

alper* OR schilder* OR concentric OR poliodystroph* = (0 technology appraisal results returned)

"diffuse cerebral" OR "encephalitis periaxialis" OR "myelinoclastic diffuse" OR "progressive neuronal" OR "sudanophilic cerebral" = 0 (0 results returned)

ahs = 0 (0 technology appraisal results returned)

myocerebrohepatopath* OR mchs = 0 (0 results returned)

myoclon* AND epilep* AND ataxia* = 0 (0 results returned)

memsa = 0 (0 results returned)

spinocerebellar AND epilep* = 0 (0 results returned)

scae = 0 (0 results returned)

external AND ophthalmoplegi* = 0 (0 results returned)

"ocular muscular" = 0 (0 results returned)

peo OR cpeo* OR arpeo OR adpeo = 0 (0 results returned)

graefe* OR ataxia OR ataxic OR miras OR sando OR melas OR myoencephalopath* OR merrf OR merff OR fukuhara* OR "ragged red" OR rars* OR pontocerebellar OR pontocerebellum OR pch* OR "pyruvate dehydrogenase" OR pdhc OR pdh OR decarboxylase = 0 (0 technology appraisal results returned)

0 documents were retrieved

1. **Source: Institute for Clinical and Economic Review webpages**

Interface / URL: https://icer-review.org/

Database coverage dates: n/a

Search date: 25/07/2022

Retrieved records: 0

Search strategy:

The following were sought: Final Evidence Report (or Draft Evidence Report if Final was not available) and the Evidence Presentation (if available) – published since the date of the previous search (23/08/2021).

Assessments were located at the following URL: <https://icer.org/explore-our-research/assessments/>.

The drop-down options were used to view all documents with status 'completed' and research type 'assessment'. 92 results were returned.

The descriptive text was checked for relevance to the eligible mitochondrial diseases. No documents were retrieved for further assessment.

1. **Source: Canadian Agency for Drugs and Technologies in Health (CADTH) webpages**

Interface / URL: https://www.cadth.ca/

Database coverage dates: n/a

Search date: 26/07/2022

Retrieved records: 0

Search strategy:

The following documents were sought: economic guidance and final recommendations associated with reimbursement reviews – published or updated from the date of the previous search (23/08/2021).

The site-wide search interface was used at: <https://www.cadth.ca/search?s>=. 'Advanced search was selected'.

Results were filtered by 'Project Line' to 'Reimbursement Review'.

Results were assessed for relevance to the eligible mitochondrial diseases. Relevant results were checked for document types of interest.

PDFs of relevant documents were retrieved for further assessment. Duplicate documents were not retrieved.

mitochondrial = 0 (7 results returned, excluded)

mitochondria = 0 (3 results returned, excluded)

mitochondrias = 0 (3 results returned, excluded)

mitochondriopathy = 0 (0 results returned)

mitochondriopathies = 0 (0 results returned)

mitochondriopathic = 0 (0 results returned)

mdas = 0 (0 results returned)

leigh = 0 (11 results returned, excluded)

leighs = 0 (11 results returned, excluded)

leigh's = 0 (11 results returned, excluded)

necrotizing = 0 (7 results returned, excluded)

necrotising = 0 (7 results returned, excluded)

necrotic = 0 (6 results returned, excluded)

polymerase = 0 (49 results returned, excluded)

polymerases = 0 (49 results returned, excluded)

polg = 0 (0 results returned)

polg1 = 0 (0 results returned

polgone = 0 (0 results returned

polgi = 0 (0 results returned)

alper = 0 (1 result returned, excluded)

alpers = 0 (1 result returned, excluded)

alper's = 0 (1 result returned, excluded)

schilder = 0 (0 results returned)

schilders = 0 (0 results returned)

schilder's = 0 (0 results returned)

balo = 0 (0 results returned)

balos = 0 (0 results returned)

balo's = 0 (0 results returned)

poliodystrophy = 0 (0 results returned)

poliodystrophies = 0 (0 results returned)

poliodystrophia = 0 (0 results returned)

poliodystrophias = 0 (0 results returned)

poliodystrophic = 0 (0 results returned)

diffuse AND cerebral = 0 (8 results returned, excluded)

periaxialis = 0 (0 results returned)

myelinoclastic = 0 (0 results returned)

sudanophilic = 0 (0 results returned)

neuronal = 0 (31 results returned, excluded)

ahs = 0 (93 results returned, excluded)

myocerebrohepatopathy = 0 (0 results returned)

myocerebrohepatopathies = 0 (0 results returned)

myocerebrohepatopathic = 0 (0 results returned)

mchs = 0 (0 results returned)

ataxia = 0 (14 results returned, excluded)

ataxias = 0 (14 results returned, excluded)

memsa = 0 (0 results returned)

scae = 0 (0 results returned)

ophthalmoplegia = 0 (0 results returned)

ophthalmoplegias = 0 (0 results returned)

ophthalmoplegic = 0 (2 results returned, excluded)

ocular AND muscular = 0 (7 results returned, excluded)

peo = 0 (12 results returned, excluded)

cpeo = 0 (0 results returned)

arpeo = 0 (0 results returned)

adpeo = 0 (0 results returned)

graefe = 0 (8 results returned, excluded)

graefes = 0 (8 results returned, excluded)

graefe's = 0 (8 results returned, excluded)

ataxic = 0 (1 result returned, excluded)

miras = 0 (0 results returned)

sando = 0 (1 result returned, excluded)

melas = 0 (0 results returned)

myoencephalopathy = 0 (0 results returned)

myoencephalopathies = 0 (0 results returned)

myoencephalopathic = 0 (0 results returned)

merrf = 0 (0 results returned)

merff = 0 (0 results returned)

fukuhara = 0 (0 results returned)

fukuharas = 0 (0 results returned)

fukuhara's = 0 (0 results returned)

ragged = 0 (3 results returned, excluded)

rars2 = 0 (0 results returned)

rars = 0 (2 results returned, excluded)

rarstwo = 0 (0 results returned)

rarsii = 0 (0 results returned)

rars11 = 0 (0 results returned)

pontocerebellar = 0 (0 results returned)

pontocerebellum = 0 (0 results returned)

pch6 = 0 (0 results returned)

pch = 0 (8 results returned, excluded)

pchsix = 0 (0 results returned)

pchvi = 0 (0 results returned)

pyruvate = 0 (2 results returned, excluded)

pdhc = 0 (0 results returned)

pdh = 0 (1 result returned, excluded)

decarboxylase = 0 (7 results returned, excluded)

lactic AND acidosis = 0 (23 results returned, excluded)

Search note: The CADTH search interface had been updated since the date of the original searches. As a result, the search terms used for this search differ from the original search.

# Search results

**Supplementary Table 1: Literature search results - HRQoL and utilities reviews**

| Resource | Number of records identified in original searches | Number of records identified in update searches |
| --- | --- | --- |
| Databases | | |
| MEDLINE ALL | 483 | 589 |
| Embase | 805 | 914 |
| Cochrane Database of Systematic Reviews (CDSR) | 8 | 8 |
| Cochrane Central Register of Controlled Trials (CENTRAL) | 189 | 225 |
| HTA Database | 23 | 27 |
| NHS EED | 27 | Not searched |
| EconLit | 79 | 99 |
| Cost-Effectiveness Analysis Registry | 0 | 0 |
| Paediatric Economic Database Evaluation | 0 | 0 |
| APA PsycInfo | 90 | 105 |
| ScHARRHud | 0 | 0 |
| Total records identified through database searching | **1704** | **1,967** |
| Other sources | | |
| National Institute for Health and Care Excellence (NICE) webpages | 0 | 0 |
| Canadian Agency for Drugs and Technologies in Health (CADTH) webpages | 0 | 0 |
| Institute for Clinical and Economic Review webpages | 0 | 0 |
| Reference list checking | 0 | 1 |
| Total additional records identified through other sources | **0** | **1** |
| Totals | | |
| Total number of records retrieved | **1,704** | **1,968** |
| Total number of records after deduplication | **1,181** | **244** |

**Supplementary Table 2: Literature search results - costs and healthcare resource use review**

| Resource | Number of records identified in original searches | Number of records identified in update searches |
| --- | --- | --- |
| **Databases** | | |
| MEDLINE ALL | 1,538 | 1,913 |
| Embase | 2,728 | 3,143 |
| Cochrane Database of Systematic Reviews (CDSR) | 8 | 8 |
| Cochrane Central Register of Controlled Trials (CENTRAL) | 230 | 307 |
| HTA Database | 23 | 27 |
| NHS EED | 27 | Not searched |
| EconLit | 79 | 99 |
| Cost-Effectiveness Analysis Registry | 0 | 0 |
| Paediatric Economic Database Evaluation | 0 | 0 |
| **Total records identified through database searching** | **4,633** | **5,497** |
| **Other sources** | | |
| National Institute for Health and Care Excellence (NICE) webpages | 0 | 0 |
| Canadian Agency for Drugs and Technologies in Health (CADTH) webpages | 0 | 0 |
| Institute for Clinical and Economic Review (ICER) webpages | 0 | 0 |
| Reference list checking | 0 | 0 |
| **Total records identified through other sources** | **0** | **0** |
| **Totals** | | |
| **Total number of records retrieved** | **4,633** | **5,497** |
| **Total number of records after deduplication** | **3,333** | **677** |

# Records excluded at full text review

**Supplementary Table 3: Excluded records list (HRQoL and utilities review n=76, healthcare resource use and costs review n=35)**

Records identified from 2021 searches are highlighted in grey.

| Reference | Exclusion reason |
| --- | --- |
| HRQoL and utilities review (n=76) | |
| Aubry E, Aeberhard C, Bally L, Nuoffer J-M, Risch L, Muhlebach S, et al. Are patients affected by mitochondrial disorders at nutritional risk? Nutrition. 2018;47:56-62. | Ineligible patient population |
| Bates MGD, Newman JH, Jakovljevic DG, Hollingsworth KG, Alston CL, Zalewski P, et al. Defining cardiac adaptations and safety of endurance training in patients with m.3243A>G-related mitochondrial disease. Int J Cardiol. 2013;168(4):3599-608. | Ineligible patient population |
| Burow P, Meyer A, Naegel S, Watzke S, Zierz S, Kraya T. Headache and migraine in mitochondrial disease and its impact on life-results from a cross-sectional, questionnaire-based study. Acta Neurol Belg. 2021;121:1151-56. | Ineligible patient population |
| Campolina-Sampaio GP, Lasmar LMdLBF, Ribeiro BSV, Giannetti JG. The Newcastle pediatric mitochondrial disease scale: translation and cultural adaptation for use in Brazil. Arq Neuropsiquiatr. 2016;74(11):909-13. | Ineligible patient population |
| Campolina-Sampaio GP, Lasmar LMdLBF, Ribeiro BSV, Gurgel-Giannetti J. "The Newcastle pediatric mitochondrial disease scale: translation and cultural adaptation for use in Brazil": erratum. Arq Neuropsiquiatr. 2017;75(2):1. | Ineligible patient population |
| Carroll JC, Nelson VS, Hurvitz EA, Priebe M. Home mechanical ventilation in mitochondrial encephalomyopathy syndrome. Arch Phys Med Rehabil. 1995;76(11):1014-16. | Ineligible patient population |
| Chinnery P, Majamaa K, Turnbull D, Thorburn D. Treatment for mitochondrial disorders. Cochrane Database Syst Rev. 2006(1):CD004426.; | Review |
| Choi HS, Lee Y-M. Enteral tube feeding in paediatric mitochondrial diseases. Sci. 2017;7(1):16909. | Ineligible outcomes |
| Cohen B, Karaa A, Haas R, Goldstein A, Vockley G. Effects of elamipretide in adults with primary mitochondrial myopathy: a phase 2 double-blind, randomized, placebo-controlled crossover trial (MMPOWER-2). Neurology. 2018(15); | Abstract |
| Crawford SA, Gong CL, Yieh L, Randolph LM, Hay JW. Diagnosing newborns with suspected mitochondrial disorders: an economic evaluation comparing early exome sequencing to current typical care. Genet Med. 2021;23(10):1854-63. | Ineligible patient population |
| de Laat P, Rodenburg RR, Roeleveld N, Koene S, Smeitink JA, Janssen MC. Six-year prospective follow-up study in 151 carriers of the mitochondrial DNA 3243 A>G variant. J Med Genet. 2021;58(1):48-55. | Ineligible patient population |
| de Laat P, Zweers HEE, Knuijt S, Smeitink JAM, Wanten GJA, Janssen MCH. Dysphagia, malnutrition and gastrointestinal problems in patients with mitochondrial disease caused by the m3243A>G mutation. Neth J Med. 2015;73(1):30-36. | Ineligible patient population |
| DiMatteo M, Aiudi A, Carr J. Mmpower-3 study design: a phase 3, randomized, double-blind, placebo-controlled trial of elamipretide in primary mitochondrial myopathy. Muscle Nerve. 2019(Suppl 1):S55.; | Abstract |
| DiMatteo M, Koenig MK. Baseline demographics of mmpower-3: a clinical trial of elamipretide in primary mitochondrial myopathy. Muscle Nerve. 2019(Suppl 1):S61.; | Abstract |
| Elson JL, Cadogan M, Apabhai S, Whittaker RG, Phillips A, Trennell MI, et al. Initial development and validation of a mitochondrial disease quality of life scale. Neuromuscul Disord. 2013;23(4):324-29. | Ineligible patient population |
| Enns GM, Kinsman SL, Perlman SL, Spicer KM, Abdenur JE, Cohen BH, et al. Initial experience in the treatment of inherited mitochondrial disease with EPI-743. Mol Genet Metab. 2012;105(1):91-102. | Ineligible patient population |
| Europees Fonds voor Regionale Ontwikkeling. The KHENERGYC study: a placebo controlled, double-blind study to explore the safety, efficacy and pharmacokinetics of sonlicromanol in children with a mitochondrial disease. Identifier: EUCTR2020-003124-16-NL. In: http://www.who.int/trialsearch/Trial2.aspx?TrialID=EUCTR2020-003124-16-NL [internet]. London: European Medicines Agency: 2020. Available from http://www.who.int/trialsearch/Trial2.aspx?TrialID=EUCTR2020-003124-16-NL. | Ineligible study design |
| Fattal O, Link J, Quinn K, Cohen BH, Franco K. Psychiatric comorbidity in 36 adults with mitochondrial cytopathies. CNS Spectr. 2007;12(6):429-38. | Ineligible patient population |
| Feeney C, Gorman G, Stefanetti R, McFarland R, Turnbull D, Harding C, et al. Lower urinary tract dysfunction in adult patients with mitochondrial disease. Neurourol Urodyn. 2020;39(8):2253-63. | Ineligible patient population |
| Finsterer J. Overview on visceral manifestations of mitochondrial disorders. Neth J Med. 2006;64(3):61-71. | Ineligible study design |
| Finsterer J. Treatment of central nervous system manifestations in mitochondrial disorders. Eur J Neurol. 2011;18(1):28-38. | Ineligible outcomes |
| Fiuza-Luces C, Diez-Bermejo J, Fernandez-De La Torre M, Rodriguez-Romo G, Sanz-Ayan P, Delmiro A, et al. Health benefits of an innovative exercise program for mitochondrial disorders. Med Sci Sports Exerc. 2018;50(6):1142-51. | Ineligible patient population |
| Fujii T, Nozaki F, Saito K, Hayashi A, Nishigaki Y, Murayama K, et al. Efficacy of pyruvate therapy in patients with mitochondrial disease: a semi-quantitative clinical evaluation study. Mol Genet Metab. 2014;112(2):133-38. | Ineligible patient population |
| Glover EI, Martin J, Maher A, Thornhill RE, Moran GR, Tarnopolsky MA. A randomized trial of coenzyme Q10 in mitochondrial disorders. Muscle Nerve. 2010;42(5):739-48. | Ineligible patient population |
| Hatzmann J, Valstar MJ, Bosch AM, Wijburg FA, Heymans HSA, Grootenhuis MA. Predicting health-related quality of life of parents of children with inherited metabolic diseases. Acta Paediatr. 2009;98(7):1205-10. | Ineligible patient population |
| Hikmat O, Vederhus BJ, Benestad MR, Engeset IME, Klingenberg C, Rasmussen M, et al. Mental health and health related quality of life in mitochondrial POLG disease. Mitochondrion. 2020;55:95-99. | Ineligible patient population |
| Karaa A, Haas R, Goldstein A, Vockley J, Cohen BH. A randomized crossover trial of elamipretide in adults with primary mitochondrial myopathy. J Cachexia Sarcopenia Muscle. 2020;11(4):909-18. | Ineligible patient population |
| Kim KR, Lee E, Namkoong K, Lee YM, Lee JS, Kim HD. Caregiver's burden and quality of life in mitochondrial disease. Pediatr Neurol. 2010;42(4):271-76. | Ineligible patient population |
| Klein I-L, van de Loo KFE, Smeitink JAM, Janssen MCH, Kessels RPC, van Karnebeek CD, et al. Cognitive functioning and mental health in mitochondrial disease: a systematic scoping review. Neurosci Biobehav Rev. 2021;125:57-77. | Ineligible study design |
| Koene S, Kozicz TL, Rodenburg RJT, Verhaak CM, de Vries MC, Wortmann S, et al. Major depression in adolescent children consecutively diagnosed with mitochondrial disorder. J Affect Disord. 2009;114(1-3):327-32. | Ineligible patient population |
| Lagler FB, Koch J, Freisinger P, Mayr J, Moder A, Sperl W. Mitofibrate CT1: a double-blind placebo controlled trial to evaluate efficacy and safety of bezafibrate for patients with mitochondrial myopathies. J Inherit Metab Dis. 2015(1 suppl. 1):S216‐17. | Abstract |
| Leong DY, Chee RY, Lui YS. Psychiatric care for a person with MELAS syndrome: a case report. Clinical Case Reports. 2021;9(5):e04146. | Ineligible outcomes |
| Li M, Zhou S, Chen C, Ma L, Luo D, Tian X, et al. Therapeutic potential of pyruvate therapy for patients with mitochondrial diseases: a systematic review. Ther. 2020;11:1-13. | Ineligible study design |
| Lim AZ, Ng YS, Blain A, Jiminez-Moreno C, Alston CL, Nesbitt V, et al. Natural history of Leigh syndrome: a study of disease burden and progression. Ann Neurol. 2022;91(1):117-30. | Ineligible patient population |
| Loekken N, Khawajazada T, Storgaard J, Raaschou-Pedersen D, Oerngreen M C, J V. No effect of resveratrol supplementation in patients with mitochondrial myopathy - a randomized, double-blind, cross-over study. J Inherit Metab Dis. 2019:22‐23.; | Abstract |
| Lokken N, Khawajazada T, Storgaard JH, Raaschou-Pedersen D, Christensen ME, Hornsyld TM, et al. No effect of resveratrol in patients with mitochondrial myopathy: a cross-over randomized controlled trial. J Inherit Metab Dis. 2021;44(5):1186-98. | Ineligible patient population |
| Mancuso M, Orsucci D, Angelini C, Bertini E, Carelli V, Comi GP, et al. "Mitochondrial neuropathies": a survey from the large cohort of the Italian Network. Neuromuscul Disord. 2016;26(4-5):272-76. | Ineligible outcomes |
| Mancuso M, Orsucci D, Ienco EC, Pini E, Choub A, Siciliano G. Psychiatric involvement in adult patients with mitochondrial disease. Neurol Sci. 2013;34(1):71-74. | Ineligible patient population |
| Mancuso M, Orsucci D, Logerfo A, Rocchi A, Petrozzi L, Nesti C, et al. Oxidative stress biomarkers in mitochondrial myopathies, basally and after cysteine donor supplementation. J Neurol. 2010;257(5):774-81. | Ineligible patient population |
| Martens A-M, Gorter H, Wassink RGV, Rietman H. Physical activity of children with a mitochondrial disease compared to children who are healthy. Pediatr. 2014;26(1):19-26. | Ineligible patient population |
| Martinelli D, Catteruccia M, Piemonte F, Pastore A, Tozzi G, Dionisi-Vici C, et al. EPI-743 reverses the progression of the pediatric mitochondrial disease--genetically defined Leigh Syndrome. Mol Genet Metab. 2012;107(3):383-88. | Ineligible patient population |
| Medical Research Council. Can the drug acipimox relieve muscle symptoms in patients with mitochondrial myopathy? Identifier: ISRCTN12895613. In: ISRCTN Registry [internet]. London: BioMed Central Limited: 2019. Available from http://www.who.int/trialsearch/Trial2.aspx?TrialID=ISRCTN12895613. | Ineligible study design |
| Na J-H, Kim HD, Lee Y-M. Effective application of corpus callosotomy in pediatric intractable epilepsy patients with mitochondrial dysfunction. Ther Adv Neurol Disord. 2022;15:1-10. | Ineligible outcomes |
| Newcastle upon Tyne Hospitals NHS Foundation Trust. Electrical brain stimulation with transcranial direct current (tDCS) to treat focal epilepsy in patients with mitochondrial disease (POLG mutation). Identifier: ISRCTN18241112. In: ISRCTN Registry [internet]. London: BioMed Central Limited: 2021. Available from https://www.isrctn.com/ISRCTN18241112. | Ongoing trial, no results |
| Orsucci D, Calsolaro V, Siciliano G, Mancuso M. Quality of life in adult patients with mitochondrial myopathy. Neuroepidemiology. 2012;38(3):194-95. | Ineligible study design |
| Parikh S, Gupta A. Autonomic dysfunction in epilepsy and mitochondrial diseases. Semin Pediatr Neurol. 2013;20(1):31-34. | Ineligible study design |
| Phoenix C, Schaefer AM, Elson JL, Morava E, Bugiani M, Uziel G, et al. A scale to monitor progression and treatment of mitochondrial disease in children. Neuromuscul Disord. 2006;16(12):814-20. | Ineligible patient population |
| Pizzamiglio C, Machado PM, Thomas RH, Gorman GS, McFarland R, Hanna MG, et al. COVID-19-related outcomes in primary mitochondrial diseases: an international study. Neurology. 2022;98(14):576-82. | Ineligible patient population |
| Radboud University. DINAMITE study nutritional state and effect diet in mitochondrial disease. Identifier: NCT02286856. In: ClinicalTrials.gov [internet]. Bethesda: US National Library of Medicine: 2014. Available from https://clinicaltrials.gov/show/NCT02286856. ; | Ineligible study design |
| Riquin E, Le Nerze T, Pasquini N, Barth M, Prouteau C, Colin E, et al. Psychiatric symptoms of children and adolescents with mitochondrial disorders: a descriptive case series. Front Psychiatr. 2021;12:685532. | Ineligible patient population |
| Saito K, Kimura N, Oda N, Shimomura H, Kumada T, Miyajima T, et al. Pyruvate therapy for mitochondrial DNA depletion syndrome. Biochim Biophys Acta. 2012;1820(5):632-36. | Ineligible patient population |
| Saneto RP, Cohen BH, Copeland WC, Naviaux RK. Alpers-huttenlocher syndrome. Pediatr Neurol. 2013;48(3):167-78. | Ineligible study design |
| Santos LR, Teive HAG, Lopes Neto FDN, Macedo ACBd, Mello NMd, Zonta MB. Quality of life in individuals with spinocerebellar ataxia type 10: a preliminary study. Arq Neuropsiquiatr. 2018;76(8):527-33. | Ineligible patient population |
| Scarpelli M, Zappini F, Filosto M, Russignan A, Tonin P, Tomelleri G. Mitochondrial sensorineural hearing loss: a retrospective study and a description of cochlear implantation in a MELAS patient. Genet Res Int. 2012;2012:287432. | Ineligible outcomes |
| Schaefer AM, Phoenix C, Elson JL, McFarland R, Chinnery PF, Turnbull DM. Mitochondrial disease in adults: a scale to monitor progression and treatment. Neurology. 2006;66(12):1932-34. | Ineligible outcomes |
| Scorza FA, Finsterer J. Sea food consumption for improving cardiac and cerebral manifestations of mitochondrial encephalopathy, lactic acidosis, and stroke-like episodes. Ann. 2017;5(17):369. | Ineligible outcomes |
| Senger BA, Ward LD, Barbosa-Leiker C, Bindler RC. The parent experience of caring for a child with mitochondrial disease. J Pediatr Nurs. 2016;31(1):32-41. | Ineligible patient population |
| Smits BW, Westeneng HJ, van Hal MA, van Engelen BG, Overeem S. Sleep disturbances in chronic progressive external ophthalmoplegia. Eur J Neurol. 2012;19(1):176-78. | Ineligible patient population |
| Sonni A, Kurdziel LBF, Baran B, Spencer RMC. The effects of sleep dysfunction on cognition, affect, and quality of life in individuals with cerebellar ataxia. Journal of Clinical Sleep Medicine. 2014;10(5):535-43. | Ineligible patient population |
| Spiegler J, Stefanova I, Hellenbroich Y, Sperner J. Bowel obstruction in patients with Alpers-Huttenlocher syndrome. Neuropediatrics. 2011;42(5):194-96. | Ineligible study design |
| Taivassalo T, Gardner JL, Taylor RW, Schaefer AM, Newman J, Barron MJ, et al. Endurance training and detraining in mitochondrial myopathies due to single large-scale mtDNA deletions. Brain. 2006;129(Pt 12):3391-401. | Ineligible patient population |
| Taivassalo T, Haller RG. Exercise and training in mitochondrial myopathies. Med Sci Sports Exerc. 2005;37(12):2094-101. | Ineligible patient population |
| Tarnopolsky MA. Exercise as a therapeutic strategy for primary mitochondrial cytopathies. J Child Neurol. 2014;29(9):1225-34. | Ineligible study design |
| Tiehuis LH, Koene S, Saris CGJ, Janssen MCH. Mitochondrial migraine; a prevalence, impact and treatment efficacy cohort study. Mitochondrion. 2020 | Ineligible patient population |
| University of Oxford. Randomised double-blinded control adaptive trial to measure of the effectiveness of acipimox in patients with Mitochondrial Myopathy. Identifier: EUCTR2018-002721-29-GB. In: EU Clinical Trials Register [internet]. London: European Medicines Agency: 2019. Available from http://www.who.int/trialsearch/Trial2.aspx?TrialID=EUCTR2018-002721-29-GB. ; | Ineligible study design |
| van de Loo KFE, Custers JAE, Koene S, Klein I-L, Janssen MCH, Smeitink JAM, et al. Psychological functioning in children suspected for mitochondrial disease: the need for care. Orphanet J Rare Dis. 2020;15(1):76. | Ineligible patient population |
| van de Loo KFE, van Zeijl NT, Custers JAE, Janssen MCH, Verhaak CM. A conceptual disease model for quality of life in mitochondrial disease. Orphanet J Rare Dis. 2022;17(1):263. | Ineligible patient population |
| van den Ameele J, Fuge J, Pitceathly RDS, Berry S, McIntyre Z, Hanna MG, et al. Chronic pain is common in mitochondrial disease. Neuromuscul Disord. 2020;30(5):413-19. | Ineligible patient population |
| Verhaak C, de Laat P, Koene S, Tibosch M, Rodenburg R, de Groot I, et al. Quality of life, fatigue and mental health in patients with the m.3243A > G mutation and its correlates with genetic characteristics and disease manifestation. Orphanet J Rare Dis. 2016;0.475694444444444 | Ineligible patient population |
| Verrotti A, Zara F, Minetti C, Striano P. Novel treatment perspectives from advances in understanding of genetic epilepsy syndromes. Expert Opinion on Orphan Drugs. 2016;4(5):485-90. | Ineligible study design |
| Whittaker R, Blackwood J, Alston C, Blakely E, Elson J, McFarland R, et al. Urine heteroplasmy is the best predictor of clinical outcome in the m.3243A>G mtDNA mutation. Neurology. 2009;72(6):568-69. | Ineligible patient population |
| Wuhan Children's Hospital. Multi-center clinical study for ketogenic diet in the treatment of mitochondrial epilepsy. Identifier: ChiCTR1900020789. In: Chinese Clinical Trial Register [internet]. Chengdu: Chinese University of Hong Kong: 2019. Available from http://www.who.int/trialsearch/Trial2.aspx?TrialID=ChiCTR1900020789. ; | Ineligible study design |
| Yatsugaad S, Povalkoa N, Nishiokaa J, Katayamaa K, Kakimotoa N, Matsuishia T, et al. MELAS: a nationwide prospective cohort study of 96 patients in Japan. Biochim Biophys Acta Gen Subj. 2012;1820(5):619-24. | Ineligible patient population |
| Zia N, Nikookam Y, Muzaffar J, Kullar P, Monksfield P, Bance M. Cochlear implantation outcomes in patients with mitochondrial hearing loss: a systematic review and narrative synthesis. J. 2021;17(1):72-80. | Ineligible study design |
| Zweers H, Smit D, Leij S, Wanten G, Janssen MCH. Individual dietary intervention in adult patients with mitochondrial disease due to the m.3243 A>G mutation. Nutrition. 2020;69:110544. | Ineligible patient population |
| Zweers H, Smit D, Leij S, Wanten G, Janssen MCH. Individual dietary intervention in adult patients with mitochondrial disease due to the m.3243A>G mutation: the DINAMITE study. J Inherit Metab Dis. 2018:S82.; | Abstract |
| Resource use and costs review (n=35) | |
| Abicht A, Scharf F, Kleinle S, Schon U, Holinski-Feder E, Horvath R, et al. Mitochondrial and nuclear disease panel (Mito-aND-Panel): combined sequencing of mitochondrial and nuclear DNA by a cost-effective and sensitive NGS-based method. Mol Genet Genomic Med. 2018;6(6):1188-98. | Ineligible outcomes |
| Buajitti E, Rosella LC, Zabzuni E, Young LT, Andreazza AC. Prevalence and health care costs of mitochondrial disease in Ontario, Canada: a population-based cohort study. PLoS ONE. 2022;17(4):e0265744. | Ineligible patient population |
| Cai W, Yang S, Han X. A case of MELAS with the m.3243A>G variant of the MT-TL1 gene mimicking acute intermittent porphyria. J Clin Neurol. 2022;18(3):361-63. | Ineligible outcomes |
| Cohen B, Balcells C, Hotchkiss B, Aggarwal K, Karaa A. A retrospective analysis of health care utilization for patients with mitochondrial disease in the United States: 2008-2015. Orphanet J Rare Dis. 2018;13(1):210. | Ineligible patient population |
| Cosma J, Russo A, Schino S, Belli M, Mango R, Chiricolo G, et al. Acute myocardial infarction in a patient with MELAS syndrome: a possible link? Minerva Cardiol Angiol. 2022;Online ahead of print | Ineligible patient population |
| Crawford SA, Gong CL, Yieh L, Randolph LM, Hay JW. Diagnosing newborns with suspected mitochondrial disorders: an economic evaluation comparing early exome sequencing to current typical care. Genet Med. 2021;23(10):1854-63. | Ineligible patient population |
| Deverell M, Phu A, Elliott EJ, Teutsch SM, Eslick GD, Stuart C, et al. Health-related out-of-pocket expenses for children living with rare diseases - tuberous sclerosis and mitochondrial disorders: a prospective pilot study in Australian families. J Paediatr Child Health. 2022;58(4):611-17. | Ineligible patient population |
| Diamond T, DiVito D, Savoca M, Mascarenhas M, Goldstein A. Nutrition rehabilitation-related complications in primary mitochondrial disorders. Nutr Clin Pract. 2021;37(2):377-82. | Ineligible patient population |
| Garcia-Perez L, Linertova R, Valcarcel-Nazco C, Serrano-Aguilar P, Posada M, Gorostiza I. Cost-of-illness studies in rare diseases: a scoping review. Orphanet J Rare Dis. 2021;16(1):178. | Ineligible study design |
| Gordon-Lipkin E, Kruk S, Thompson E, Yeske P, Martin L, Hirano M, et al. Risk mitigation behaviors to prevent infection in the mitochondrial disease community during the COVID-19 pandemic. Mol Genet Metab Rep. 2022;30:100837. | Ineligible patient population |
| Hoitzing H, Gammage PA, Haute LV, Minczuk M, Johnston IG, Jones NS. Energetic costs of cellular and therapeutic control of stochastic mitochondrial DNA populations. PLoS Comput Biol. 2019;15(6):e1007023. | Ineligible patient population |
| Howard RS, Russell S, Losseff N, Harding AE, Hughes JM, Wiles CM, et al. Management of mitochondrial disease on an intensive care unit. Qjm. 1995;88(3):197-207. | Ineligible outcomes |
| Inci A, Okur I, Demir E, Biberoglu G, Tumer L, Serdaroglu A, et al. An ultra-rare cause of severe hypotonia mimicking Pompe disease in an infant: RRM2B related mitochon-drial DNA depletion syndrome with a novel mutation. Neurol Asia. 2022;27(1):199-202. | Ineligible patient population |
| Katkevica A, Kreile M, Grinfelde I, Taurina G, Micule I, Dzivite-Krisane I, et al. Two cases of Leigh syndrome in one family: diagnostic challenges and clinical management experience in Latvia. Case Rep Med. 2021:5266820.; | Ineligible patient population |
| Kharbouch H, Boussaadani B, Fellat I, Oukerraj L, Doghmi N, Cherti M. Kearns Sayre syndrome: a rare etiology of complete atrioventricular block in children (case report). Pan Afr Med J. 2021;40:154. | Ineligible patient population |
| Kim NH, Siddiqui M, Vogel J. Melas syndrome and midd unmasked by metformin use: a case report. Ann Intern Med. 2021;174(1):124-25. | Ineligible outcomes |
| Koene S, Wortmann SB, de Vries MC, Jonckheere AI, Morava E, de Groot IJM, et al. Developing outcome measures for pediatric mitochondrial disorders: which complaints and limitations are most burdensome to patients and their parents? Mitochondrion. 2013;13(1):15-24. | Ineligible outcomes |
| Landis TM, Hannah WB, Powers WJ. "Disappearing infarct" is late-onset MELAS. Ann Neurol. 2021;90(6):1001-02. | Ineligible outcomes |
| Leong DY, Chee RY, Lui YS. Psychiatric care for a person with MELAS syndrome: a case report. Clin Case Rep. 2021;9(5):e04146. | Ineligible outcomes |
| Liang J-M, Xin C-J, Wang G-L, Wu X-M. Late-onset Leigh syndrome without delayed development in China: a case report. World J Clin Cases. 2021;9(24):7133-38. | Ineligible patient population |
| Lim AZ, Ng YS, Blain A, Jiminez-Moreno C, Alston CL, Nesbitt V, et al. Natural history of Leigh syndrome: a study of disease burden and progression. Ann Neurol. 2022;91(1):117-30. | Ineligible patient population |
| Long JC, Best S, Hatem S, Theodorou T, Catton T, Murray S, et al. The long and winding road: perspectives of people and parents of children with mitochondrial conditions negotiating management after diagnosis. Orphanet J Rare Dis. 2021;16(1):310. | Ineligible patient population |
| Maruo Y, Ueda Y, Murayama K, Takeda A. A case report of Leigh syndrome diagnosed by endomyocardial biopsy. Eur Heart J Case Rep. 2021;5(2):1-7. | Ineligible outcomes |
| McCormack SE, Xiao R, Kilbaugh TJ, Karlsson M, Ganetzky RD, Cunningham ZZ, et al. Hospitalizations for mitochondrial disease across the lifespan in the U.S. Mol Genet Metab. 2017;121(2):119-26. | Ineligible patient population |
| Meng Y, Clarke PM, Goranitis I. The value of genomic testing: a contingent valuation across six child- and adult-onset genetic conditions. Pharmacoeconomics. 2022;40(2):215-23. | Ineligible patient population |
| Mitsutake T, Tsugawa J, Kimura S, Yamada M, Hanada H, Morinaga Y, et al. Endovascular revascularization for internal carotid artery dissection during stroke-like episode in MELAS: a case report. Neurol Clin Neurosci. 2022;10(2):92-94. | Ineligible patient population |
| Nguyen T, Alzahrani T, Krepp J, Panjrath G. Cardiovascular outcomes in patients with mitochondrial disease in the United States: a propensity score analysis. Tex Heart Inst J. 2021;48(3):1-6. | Ineligible patient population |
| Nishida H, Nawano T, Fukuhara H, Takai S, Narisawa T, Kanno H, et al. Outcomes of living kidney transplantation for mitochondrial disease patients: a case series. Transplant Proc. 2022;54(2):267-71. | Ineligible outcomes |
| Paiva Coelho M, Martins E, Vilarinho L. Diagnosis, management, and follow-up of mitochondrial disorders in childhood: a personalized medicine in the new era of genome sequence. Eur J Pediatr. 2019;178(1):21-32. | Ineligible patient population |
| Papa V, Salfi NCM, Costa R, Bettocchi I, Ricci E, Cordelli DM, et al. Ultrastructural and immunohistochemical diagnosis of a neonatal herpes simplex virus infection presenting as fulminant hepatitis: a case report. In: Advances in Experimental Medicine and Biology. United States: Springer; 2022. p. 93-100. | Ineligible patient population |
| Pizzamiglio C, Machado PM, Thomas RH, Gorman GS, McFarland R, Hanna MG, et al. COVID-19-related outcomes in primary mitochondrial diseases: an international study. Neurology. 2022;98(14):576-82. | Ineligible patient population |
| Sugiura K, Ishimaru S, Fukuda K. Atypical Leber hereditary optic neuropathy with a 34-year interval between vision loss in both eyes. Am J Ophthalmol Case Rep. 2022;25:101263. | Ineligible patient population |
| Wu Y, Balasubramaniam S, Rius R, Thorburn DR, Christodoulou J, Goranitis I. Genomic sequencing for the diagnosis of childhood mitochondrial disorders: a health economic evaluation. Eur J Hum Genet. 2021;30:577-86. | Ineligible patient population |
| Wuhan Children's Hospital. Multi-center clinical study for ketogenic diet in the treatment of mitochondrial epilepsy. Identifier: ChiCTR-1900020789. In: Chinese Clinical Trial Register [internet]. Chengdu: Chinese University of Hong Kong: 2019. Available from http://www.who.int/trialsearch/Trial2.aspx?TrialID=ChiCTR1900020789. ; | Ineligible study design |
| Ye Z, Xue A, Huang Y, Wu Q. Children with cyclic vomiting syndrome: phenotypes, disease burden and mitochondrial DNA analysis. BMC Gastroenterol. 2018;18(1):104. | Ineligible patient population |

# Included studies

**Supplementary Table 4: Included studies (HRQoL n=7, healthcare resource use n=3)**

Records identified from 2021 searches are highlighted in grey.

| HRQoL Studies (n=7) |
| --- |
| Eom S, Lee Y-M. Preliminary study of neurodevelopmental outcomes and parenting stress in Paediatric mitochondrial sisease. Pediatr Neurol. 2017;71(1):43-49.e1. |
| Hendrix CLF, van den Heuvel FMA, Rodwell L, Timmermans J, Nijveldt R, Janssen MCH, et al. Screening and prevalence of cardiac abnormalities on electro- and echocardiography in a large cohort of patients with mitochondrial disease. Mol Genet Metab. 2022;136(3):219-25. |
| Koene S, Hendriks JCM, Dirks I, de Boer L, de Vries MC, Janssen MCH*, et al.* International paediatric mitochondrial disease scale. J Inherit Metab Dis. 2016;39(5):705-12. |
| Koga Y, Povalko N, Inoue E, Nashiki K, Tanaka M. Biomarkers and clinical rating scales for sodium pyruvate therapy in patients with mitochondrial disease. Mitochondrion. 2019;48:11-15. |
| Li Y, Li S, Qiu Y, Zhou M, Chen M, Hu Y, et al. Circulating FGF21 and GDF15 as biomarkers for screening, diagnosis, and severity assessment of primary mitochondrial disorders in children. Front Pediatr. 2022;10:851534. |
| van Kempen CMA, Beynon AJ, Smits JJ, Janssen MCH. A retrospective cohort study exploring the association between different mitochondrial diseases and hearing loss. Mol Genet Metab. 2022;135(4):333-41. |
| Wang J, Liu Z, Xu M, Han X, Ren C, Yang X*, et al.* Cinical, metabolic, and genetic analysis and follow-up of eight patients with HIBCH mutations presenting with Leigh/Leigh-like Syndrome. Front Pharmacol. 2021;12(1):605803. |
| Healthcare Resource Use Studies (n=3) |
| Eom S, Lee HN, Lee S, Kang H-C, Lee JS, Kim HD*, et al.* Cause of death in children with mitochondrial diseases. Pediatr Neurol. 2017;66(1):82-88. |
| Shimizu J, Tabata T, Tsujita Y, Yamane T, Yamamoto Y, Tsukamoto T*, et al.* Propofol infusion syndrome complicated with mitochondrial myopathy, encephalopathy, lactic acidosis, and stroke-like episodes: A case report. Acute med. 2020;7(1):e473. |
| Yesilbas O, Sengenc E, Olbak ME, Bako D, Nizam OG, Seyithanoglu MH, et al. First case of MELAS syndrome presenting with local brain edema requiring decompressive craniectomy. Turk Neurosurg. 2022;32(1):155-59. |

# Quality assessment

The detailed quality assessment for the studies reporting HRQoL data is provided in Supplementary Table 5.

Since NICE does not recommend a specific quality assessment tool for utility studies, and there is no published and validated quality assessment checklist or tool for assessing HRQoL/utility studies, we assessed the quality and relevance of each study by considering the following issues (aligned to NICE Decision Support Unit (DSU) recommendations [6]):

- Selection of participants and sample size – was there clear inclusion and exclusion criteria? Was the sample size and the response rate large enough to be representative of the population?
- Were patients lost at follow up? Why? Could this influence the results and their interpretation?
- Were missing data analysed? How? Was it an appropriate methodology?
- How representative of the target population were the participants in the study, considering age, disease severity, co-morbidities?
- Was a reasonable pilot testing approach used?
- Were the utility elicitation methods reasonable/valid?
- Have the researchers tried to reduce potentials for bias, for example interviewer bias?
- Have the researchers offered assessments of the limitations of the study approach?

**Supplementary Table 5: Quality Assessment HRQoL**

| Study | Selection of participants – was there clear inclusion and exclusion criteria? | Was the sample size and the response rate large enough to be representative of the population? | Were patients lost at follow up? Why? Could this influence the results and their interpretation? | Were missing data analysed? How? Was it an appropriate methodology? | How representative of the target population were the participants in the study, considering age, disease severity, co-morbidities? | Was a reasonable pilot testing approach used? | Were the HRQoL tool/utility elicitation methods reasonable / valid? | Have the researchers tried to reduce potentials for bias, for example interviewer bias? | Have the researchers offered assessments of the limitations of the study approach? |
| --- | --- | --- | --- | --- | --- | --- | --- | --- | --- |
| Eom and Lee (2017) [7] | Yes, inclusion criteria were reported. | Small sample size due to the very rare disease analysed. | No patients appear to have been lost to follow up. | No missing data. | Representative, considering disease severity and neurological condition. | No | Yes | No | Yes. The authors noted some limitations such as sampling bias and the use of prediagnostic profiles of children’s neuropsychology and their mothers’ comorbidity. |
| Hendrix et al. (2022) [8] | Yes, inclusion and exclusion criteria were briefly reported. | Small sample size due to the very rare disease analysed. | No patients appear to have been lost to follow-up. | Several variables had missing data. No details provided regarding dealing with missing data. | Representative, considering disease severity and clinical diagnosis. | No | Yes | No | Yes. The authors noted some limitations including the retrospective design and missing data in several variables as well as the small sample size in some of the disease subgroups. |
| Koene et al. (2016) [9] | Yes, inclusion and exclusion criteria were briefly reported. | Small sample size due to the very rare disease analysed. | No patients appear to have been lost to follow up. | No missing data. | Not representative. Only 3 patients were eligible for the current analysis. | No | Yes | No | Yes. The authors stated that the main limitation of their study was the small sample size. |
| Koga et al. (2019) [10] | Yes, inclusion and exclusion criteria were briefly reported. | Small sample size due to the very rare disease analysed. | No patients appear to have been lost to follow up. | No missing data. | Not representative. Only 3 patients were eligible for the current analysis. | Partly | Yes | No | Yes. The authors pointed out the limited sample size and the need for a more sensitive and quantitative clinical rating scale. |
| Li et al. (2022) [11] | Yes, inclusion criteria were briefly reported. | Small sample size due to the very rare disease analysed. | No patients appear to have been lost to follow-up. | No missing data. | Not representative. Only 2 patients were eligible for the current analysis. | No | Yes | Yes. Two doctors performed the scoring process independently for the IPMDS. | No |
| van Kempen et al. (2022) [12] | Yes, inclusion criteria were briefly reported. | Small sample size due to the very rare disease analysed. | No patients appear to have been lost to follow-up. | Several variables had missing data. No details provided regarding dealing with missing data. | Representative, considering disease severity and clinical diagnosis. | No | Yes | No | Yes. The authors mentioned the small sample size and lack of statistical power in some of the disease subgroups. |
| Wang et al. (2021) [13] | Yes, inclusion and exclusion criteria were briefly reported. | Small sample size due to the very rare disease analysed. | No patients appear to have been lost to follow up. | No missing data. | Not representative. Only 3 patients were eligible for the current analysis. | No | Yes | No | Yes. The authors stated that the main limitations of their study were the small sample size and the absence of formal clinical trials. |

# PRISMA checklist

**Supplementary Table 6: PRISMA checklist**

| **Section and Topic** | **Item #** | **Checklist item** | **Location where item is reported** |
| --- | --- | --- | --- |
| **TITLE** | | |  |
| Title | 1 | Identify the report as a systematic review. | Title |
| **ABSTRACT** | | |  |
| Abstract | 2 | See the PRISMA 2020 for Abstracts checklist. | Completed |
| **INTRODUCTION** | | |  |
| Rationale | 3 | Describe the rationale for the review in the context of existing knowledge. | Background |
| Objectives | 4 | Provide an explicit statement of the objective(s) or question(s) the review addresses. | Background |
| **METHODS** | | |  |
| Eligibility criteria | 5 | Specify the inclusion and exclusion criteria for the review and how studies were grouped for the syntheses. | Methods – eligibility criteria |
| Information sources | 6 | Specify all databases, registers, websites, organisations, reference lists and other sources searched or consulted to identify studies. Specify the date when each source was last searched or consulted. | Methods - searches |
| Search strategy | 7 | Present the full search strategies for all databases, registers and websites, including any filters and limits used. | Additional file |
| Selection process | 8 | Specify the methods used to decide whether a study met the inclusion criteria of the review, including how many reviewers screened each record and each report retrieved, whether they worked independently, and if applicable, details of automation tools used in the process. | Methods – study selection, data extraction and quality assessment |
| Data collection process | 9 | Specify the methods used to collect data from reports, including how many reviewers collected data from each report, whether they worked independently, any processes for obtaining or confirming data from study investigators, and if applicable, details of automation tools used in the process. | Methods – study selection, data extraction and quality assessment |
| Data items | 10a | List and define all outcomes for which data were sought. Specify whether all results that were compatible with each outcome domain in each study were sought (e.g. for all measures, time points, analyses), and if not, the methods used to decide which results to collect. | Methods – study selection, data extraction and quality assessment |
|  | 10b | List and define all other variables for which data were sought (e.g. participant and intervention characteristics, funding sources). Describe any assumptions made about any missing or unclear information. | Methods – study selection, data extraction and quality assessment |
| Study risk of bias assessment | 11 | Specify the methods used to assess risk of bias in the included studies, including details of the tool(s) used, how many reviewers assessed each study and whether they worked independently, and if applicable, details of automation tools used in the process. | Methods – study selection, data extraction and quality assessment |
| Effect measures | 12 | Specify for each outcome the effect measure(s) (e.g. risk ratio, mean difference) used in the synthesis or presentation of results. | N/A |
| Synthesis methods | 13a | Describe the processes used to decide which studies were eligible for each synthesis (e.g. tabulating the study intervention characteristics and comparing against the planned groups for each synthesis (item #5)). | N/A |
|  | 13b | Describe any methods required to prepare the data for presentation or synthesis, such as handling of missing summary statistics, or data conversions. | N/A |
|  | 13c | Describe any methods used to tabulate or visually display results of individual studies and syntheses. | N/A |
|  | 13d | Describe any methods used to synthesize results and provide a rationale for the choice(s). If meta-analysis was performed, describe the model(s), method(s) to identify the presence and extent of statistical heterogeneity, and software package(s) used. | N/A |
|  | 13e | Describe any methods used to explore possible causes of heterogeneity among study results (e.g. subgroup analysis, meta-regression). | N/A |
|  | 13f | Describe any sensitivity analyses conducted to assess robustness of the synthesized results. | N/A |
| Reporting bias assessment | 14 | Describe any methods used to assess risk of bias due to missing results in a synthesis (arising from reporting biases). | N/A |
| Certainty assessment | 15 | Describe any methods used to assess certainty (or confidence) in the body of evidence for an outcome. | N/A |
| **RESULTS** | | |  |
| Study selection | 16a | Describe the results of the search and selection process, from the number of records identified in the search to the number of studies included in the review, ideally using a flow diagram. | Results |
|  | 16b | Cite studies that might appear to meet the inclusion criteria, but which were excluded, and explain why they were excluded. | Additional file |
| Study characteristics | 17 | Cite each included study and present its characteristics. | Results |
| Risk of bias in studies | 18 | Present assessments of risk of bias for each included study. | Additional file |
| Results of individual studies | 19 | For all outcomes, present, for each study: (a) summary statistics for each group (where appropriate) and (b) an effect estimate and its precision (e.g. confidence/credible interval), ideally using structured tables or plots. | Results |
| Results of syntheses | 20a | For each synthesis, briefly summarise the characteristics and risk of bias among contributing studies. | N/A |
|  | 20b | Present results of all statistical syntheses conducted. If meta-analysis was done, present for each the summary estimate and its precision (e.g. confidence/credible interval) and measures of statistical heterogeneity. If comparing groups, describe the direction of the effect. | N/A |
|  | 20c | Present results of all investigations of possible causes of heterogeneity among study results. | N/A |
|  | 20d | Present results of all sensitivity analyses conducted to assess the robustness of the synthesized results. | N/A |
| Reporting biases | 21 | Present assessments of risk of bias due to missing results (arising from reporting biases) for each synthesis assessed. | N/A |
| Certainty of evidence | 22 | Present assessments of certainty (or confidence) in the body of evidence for each outcome assessed. | N/A |
| **DISCUSSION** | | |  |
| Discussion | 23a | Provide a general interpretation of the results in the context of other evidence. | Discussion |
|  | 23b | Discuss any limitations of the evidence included in the review. | Discussion |
|  | 23c | Discuss any limitations of the review processes used. | Discussion |
|  | 23d | Discuss implications of the results for practice, policy, and future research. | Discussion |
| **OTHER INFORMATION** | | |  |
| Registration and protocol | 24a | Provide registration information for the review, including register name and registration number, or state that the review was not registered. | Methods |
|  | 24b | Indicate where the review protocol can be accessed, or state that a protocol was not prepared. | Methods |
|  | 24c | Describe and explain any amendments to information provided at registration or in the protocol. | N/A |
| Support | 25 | Describe sources of financial or non-financial support for the review, and the role of the funders or sponsors in the review. | Funding |
| Competing interests | 26 | Declare any competing interests of review authors. | Competing interests |
| Availability of data, code and other materials | 27 | Report which of the following are publicly available and where they can be found: template data collection forms; data extracted from included studies; data used for all analyses; analytic code; any other materials used in the review. | N/A |

# References

1. Chang X, Wu Y, Zhou J, Meng H, Zhang W, Guo J. A meta-analysis and systematic review of Leigh syndrome: clinical manifestations, respiratory chain enzyme complex deficiency, and gene mutations. Medicine (Baltimore). 2020;99(5):e18634.

2. Anagnostou ME, Ng YS, Taylor RW, McFarland R. Epilepsy due to mutations in the mitochondrial polymerase gamma (POLG) gene: a clinical and molecular genetic review. Epilepsia. 2016;57(10):1531-45.

3. Arber M, Garcia S, Veale T, Edwards M, Shaw A, Glanville JM. Performance of ovid medline search filters to identify health state utility studies. Int J Technol Assess Health Care. 2017;33(4):472-80.

4. Centre for Reviews and Dissemination. Search strategies - NHS EED [online database]. York: CRD; 2021. [cited January 2021]. Available from: <https://www.crd.york.ac.uk/crdweb/searchstrategies.asp#nhseedmedline>.

5. Clarivate. EndNote [X9.3.3 for Windows & Mac]. [program] Philadelphia, USA: Clarivate; 2020. Available from: <http://endnote.com/>.

6. National Institute of Health and Care Excellence Decision Support Unit. Technical support document 9: the identification, review and synthesis of health state utility values from the literature. London: NICE DSU; 2010. Available from: <https://www.ncbi.nlm.nih.gov/books/NBK425822/>.

7. Eom S, Lee Y-M. Preliminary study of neurodevelopmental outcomes and parenting stress in pediatric mitochondrial sisease. Pediatr Neurol. 2017;71(1):43-49.e1.

8. Hendrix CLF, van den Heuvel FMA, Rodwell L, Timmermans J, Nijveldt R, Janssen MCH*, et al.* Screening and prevalence of cardiac abnormalities on electro- and echocardiography in a large cohort of patients with mitochondrial disease. Mol Genet Metab. 2022;136(3):219-25.

9. Koene S, Hendriks JCM, Dirks I, de Boer L, de Vries MC, Janssen MCH*, et al.* International paediatric mitochondrial disease scale. J Inherit Metab Dis. 2016;39(5):705-12.

10. Koga Y, Povalko N, Inoue E, Nashiki K, Tanaka M. Biomarkers and clinical rating scales for sodium pyruvate therapy in patients with mitochondrial disease. Mitochondrion. 2019;48:11-15.

11. Li Y, Li S, Qiu Y, Zhou M, Chen M, Hu Y*, et al.* Circulating FGF21 and GDF15 as biomarkers for screening, diagnosis, and severity assessment of primary mitochondrial disorders in children. Front Pediatr. 2022;10:851534.

12. van Kempen CMA, Beynon AJ, Smits JJ, Janssen MCH. A retrospective cohort study exploring the association between different mitochondrial diseases and hearing loss. Mol Genet Metab. 2022;135(4):333-41.

13. Wang J, Liu Z, Xu M, Han X, Ren C, Yang X*, et al.* Cinical, metabolic, and genetic analysis and follow-up of eight patients with HIBCH mutations presenting with Leigh/Leigh-like Syndrome. Front Pharmacol. 2021;12(1):605803.
